# Public awareness and knowledge of antibiotic use and antimicrobial resistance in Malta

**DOI:** 10.1186/s12889-026-29529-w

**Published:** 2026-09-28

**Authors:** Gavin Schranz, Vanessa Camilleri, Monique Sciortino, Michael A. Borg, Ermira Tartari

**Affiliations:** 1 University of Malta, Msida Campus, Msida, MSD2080 Malta; 2 Department of Infection Prevention and Control, Mater Dei Hospital, Msida, Malta

**Keywords:** Antimicrobial resistance, Antibiotic misuse, Patient education

## Abstract

**Introduction:**

Antimicrobial resistance (AMR) is a major global threat, driven partly by inappropriate antibiotic use. Understanding public knowledge, awareness, and behaviours is critical for designing effective interventions. This study assessed these dimensions within the Maltese population to inform national AMR awareness and stewardship strategies.

**Method:**

A cross-sectional online survey was conducted between 9 and 29 April 2024 among Maltese residents using a questionnaire adapted from the World Health Organization (WHO) Antibiotic Resistance Multi-Country Public Awareness Survey. A convenience sampling approach with active demographic monitoring was used across gender, age, and education groups, using census-derived targets as benchmarks. The survey, available in English and Maltese, assessed knowledge of appropriate antibiotic use, awareness of AMR-related terminology, and attitudes towards antibiotic use and resistance. Data were analysed using descriptive statistics and non-parametric tests (Mann-Whitney U, Kruskal-Wallis H, Chi-Square, Likelihood Ratio) to explore associations between demographic factors and knowledge or behavioural outcomes.

**Results:**

A total of 476 eligible respondents completed the survey and were included in the analysis. Among respondents, 59.9% were female and the largest age group was 25–34 years (31.9%). Awareness of “antibiotic resistance” was high (88%) yet understanding of “antimicrobial resistance” (48%) and its abbreviation “AMR” (21%) was lower. Most participants (97%) agreed that antibiotics should only be used when prescribed, but misconceptions persisted: 16.6% believed antibiotics treat colds and flu, 33.6% for fever, and 39.9% for sore throats. Three-quarters (75%) incorrectly attributed resistance to the body becoming resistant rather than bacteria. Older and more educated respondents showed significantly better knowledge and more appropriate attitudes (*p* < 0.05), whereas younger adults and males were more likely to hold misconceptions. While nearly all respondents (96.7%) agreed that individuals should use antibiotics responsibly, fewer (53.4%) viewed AMR as a major global challenge or expressed personal concern (67.2%) about its impact, and one-third believed they were not at risk if they used antibiotics correctly. Findings indicate general awareness but persistent conceptual and demographic gaps.

**Conclusion:**

Despite high awareness of appropriate antibiotic use, misconceptions about treatment indications and the mechanisms of AMR remain, particularly among younger adults and those with lower education. Improving public understanding of when antibiotics are needed and how resistance develops will be critical to promoting responsible use. These findings provide locally generated evidence to inform targeted public health communication and support AMR prevention efforts in Malta.

**Supplementary Information:**

The online version contains supplementary material available at https://doi.org/10.1186/s12889-026-29529-w.

## Introduction

The discovery of antibiotics revolutionised the treatment of infectious diseases and remains one of the most significant milestones in modern medicine [1]. However, their misuse and overuse have accelerated the development of antimicrobial resistance (AMR), a global threat [2] that undermines the effectiveness of treatments and increases morbidity, mortality, and healthcare costs [3–6] The European Commission reports that resistant infections account for approximately 25,000 deaths annually in Europe, and global estimates suggest this figure could rise to 10 million per year by 2050 if urgent measures are not implemented [3]. The European Antimicrobial Resistance Surveillance System (EARSS) has documented persistently high resistance levels in the Mediterranean region [7] where antibiotic consumption remains high [8].

Beyond clinical and microbiological drivers, public knowledge, awareness, and behaviours play a critical role in influencing antibiotic use and the development of AMR. A systematic review reported that while many individuals are aware of antibiotic resistance, misconceptions are widespread, most notably the belief that resistance develops in the human body rather than in bacteria [9]. Such misunderstandings can lead to inappropriate behaviours, including self-medication, incomplete treatment courses, and reliance on antibiotics for viral infections [10–12]. Understanding these knowledge and awareness gaps is therefore central to shaping effective public health interventions and promoting responsible antibiotic use.

In the European context, several population-based surveys, including the WHO’s Antibiotic Resistance Multi-Country Public Awareness Survey [13] and the European Commission’s Eurobarometer study, have explored public perceptions of antibiotics and AMR. Findings consistently show substantial misuse of antibiotics for common respiratory conditions such as colds, sore throats, and influenza [13, 16]. In Malta, antibiotic consumption is among the highest in Europe, and community prescribing for minor viral illnesses remains common [17,18]. The 2017 the European Centre for Disease Prevention and Control (ECDC) country assessment highlighted the need for stronger community education awareness programmes on appropriate antibiotic use [17]. Malta’s National AMR strategy [18] has prioritised improving understanding of AMR and promoting responsible use as part of the WHO’s Global Action Plan on AMR [19].

Despite these strategic commitments, no published data have assessed the Maltese population’s knowledge, awareness and practices concerning antibiotic use and AMR.

Establishing this evidence base is essential to guide national awareness campaigns and evaluate progress towards the objectives of Malta’s AMR action plan. Understanding how demographic characteristics influence knowledge and behaviour can further support the development of targeted, context-specific interventions. This study aimed to assess public knowledge, awareness and practices related to antibiotic use and AMR in Malta and to examine how these outcomes vary across the demographic groups.

## Methods

### Study design and period

A cross-sectional online survey was conducted in Malta between 9 and 29 April 2024 to assess public knowledge, awareness, and practices related to antibiotic use and AMR.

### Study instrument

The questionnaire was adapted from the WHO Antibiotic Resistance Multi-Country Public Awareness Survey [13]. This tool supports the objectives of the WHO global action plan on AMR to raise awareness and promote responsible antibiotic use [19]. Validated across regions, the survey demonstrates reliability and allows for cross-country comparisons, making it an effective instrument for assessing public knowledge, awareness and practices related to antibiotic use [20, 21]. Minor contextual modifications ensured local relevance: items on residential setting and ethnicity were excluded because of Malta’s small, relatively homogeneous population, and questions on agricultural antibiotic use were removed to maintain focus on human health [22]. The survey, implemented in English and Maltese via Google Forms (Supplementary Material 1), was anonymous and required about ten minutes to complete. It comprised the following sections: (i) demographics; (ii) use of antibiotics, (iii) knowledge about antibiotics, and v) knowledge about antimicrobial resistance.

Content validity was supported through review of the AMR literature and comparison with established survey items. Face validity was assessed through pilot testing with 10 respondents, after which minor wording adjustments were made to improve clarity and comprehension [13].

### Target population and recruitment

The target population comprised Maltese residents aged ≥ 18 years. Participants were recruited using a convenience sampling approach with active demographic monitoring. Demographic targets for age, gender, and education were informed by the NSO 2021 census distributions [22] and the WHO survey benchmarks [13].

Recruitment took place over a three-week period, from 9 to 29 April 2024. The survey was disseminated through social media channels, community networks, institutional mailing lists, and word-of-mouth. During data collection, response distributions were monitored periodically. When specific demographic groups appeared under-represented, additional outreach was directed toward networks and platforms considered more likely to reach those groups. Participation remained open to all eligible individuals throughout the data collection period and was not closed once demographic targets were reached.

The sample size was determined by the number of eligible respondents who completed the questionnaire during the data collection period, within the practical constraints of a non-funded, student-led study relying on voluntary participation.

Participation was voluntary and anonymous, and submission of the online questionnaire indicated informed consent. Before accessing the questionnaire, participants were provided with study information, including the purpose of the research, eligibility criteria, anonymity of responses, and voluntary nature of participation. Further details on the online questionnaire, including adherence to the CHERRIES reporting checklist, are provided in Supplementary Material 2 [23].

### Statistical analysis

Data were exported to IBM SPSS v29.0.2.0 for analysis [19]. Descriptive statistics (such as counts, percentages, medians, and interquartile ranges) were used to summarise demographic characteristics and survey responses. For Likert-scale statements [24–26] agreement categories were coded as 1 (Strongly Disagree) to 5 (Strongly Agree). Statements with three response categories were coded as 1 (Yes/True), 2 (No/False), and 3 (I Don’t Know). Full descriptive statistics for all Likert-scale statements are provided in Supplementary Material 3. Associations between demographic variables (age, gender, education, household income, and region) and knowledge-related statements (with three categories) were examined using Pearson Chi-square or Likelihood ratio test in cases where more than 20% of the expected cells in the contingency table were less than 5 [26]. As data were non-normally distributed (verified by Shapiro-Wilk and Kolmogorov-Smirnov tests), non-parametric tests were applied to test for significant demographic differences on the Likert-scale statements’ agreement: the Mann-Whitney U test compared two independent groups, and the Kruskal-Wallis H test assessed differences across more than two groups [27–29]. Demographic variables were regrouped for analysis (age, education, and household income) where required to ensure adequate cell sizes for the statistical tests, and full pairwise comparison tables and rank-based diagrams are provided in Supplementary Material 3. The level of significance for all statistical tests was set to be 0.05.

### Ethical considerations

Participation was voluntary and anonymous, with no identifiable data collected. Ethical approval was granted by the University of Malta Research Ethics Committee (Reference number ICT-2023-00339). The WHO *Antibiotic Resistance: Multi-Country Public Awareness Survey* questionnaire was used under the Creative Commons BY-NC-SA 3.0 IGO licence, permitting non-commercial adaptation of the original instrument. The questionnaire was available in English and Maltese; the Maltese version was produced through translation and back-translation to ensure conceptual equivalence.

## Results

### Sample demographic information

A total of 476 eligible participants completed the survey and were included in the final analysis. Among the survey respondents, 59.9% (*n* = 285) were female and 40.1% (*n* = 191) male, indicating a higher participation rate among female respondents (see Table 1). There was a higher participation rate among respondents aged 25–34 (31.9%, *n* = 152), followed by those aged 35–44 (25.6%, *n* = 122). The smallest age group was adults aged 65 and over (4.6%, *n* = 22), followed by those aged 55–64 (8.8%, *n* = 42). The respondents had a diverse range of education levels; 47.3% (*n* = 225) of respondents held higher education degrees (Master’s and Doctorate), slightly below the target of 53%. Those with further education (Bachelor’s and Technical/Vocational) made up 31.9% (*n* = 152), exceeding the target of 22%. Basic education respondents (High school and some college) were 19% (*n* = 90), slightly below the 22% target, while only 13.4% (*n* = 64) had no formal education beyond 12th grade, compared to the 3% target (Table 1).

**Table 1:** Demographic distribution of survey respondents by gender, age, region, household income, and education level

Category	Group	*n*	Observed (%)
Gender	Male	191	40.1
	Female	285	59.9
	Prefer not to say	0	0.0
Age	18–24	73	15.3
	25–34	152	31.9
	35–44	122	25.6
	45–54	65	13.7
	55–64	42	8.8
	65+	22	4.6
Region	Southern Harbour	42	8.8
	Northern Harbour	171	35.9
	South Eastern	65	13.7
	Western	87	18.3
	Northern	98	20.6
	Gozo and Comino	13	2.7
Household Income	€0	1	0.2
	€1–9,999	10	2.1
	€10,000–24,999	48	10.1
	€25,000–49,999	164	34.5
	€50,000–74,999	87	18.3
	€75,000–99,999	38	8.0
	€100,000 or more	67	14.1
	Prefer not to answer	61	12.8
Education Level	12th Grade or Less/No Diploma	9	1.9
	Some College Credit/No Degree	35	7.4
	High School Graduate with Diploma	55	11.6
	Technical/Vocational Training or Associate Degree	20	4.2
	Bachelor’s Degree	132	27.7
	Master’s Degree	199	41.8
	Doctorate Degree	26	5.5

### Knowledge on antibiotic use

**Fig. 1:**
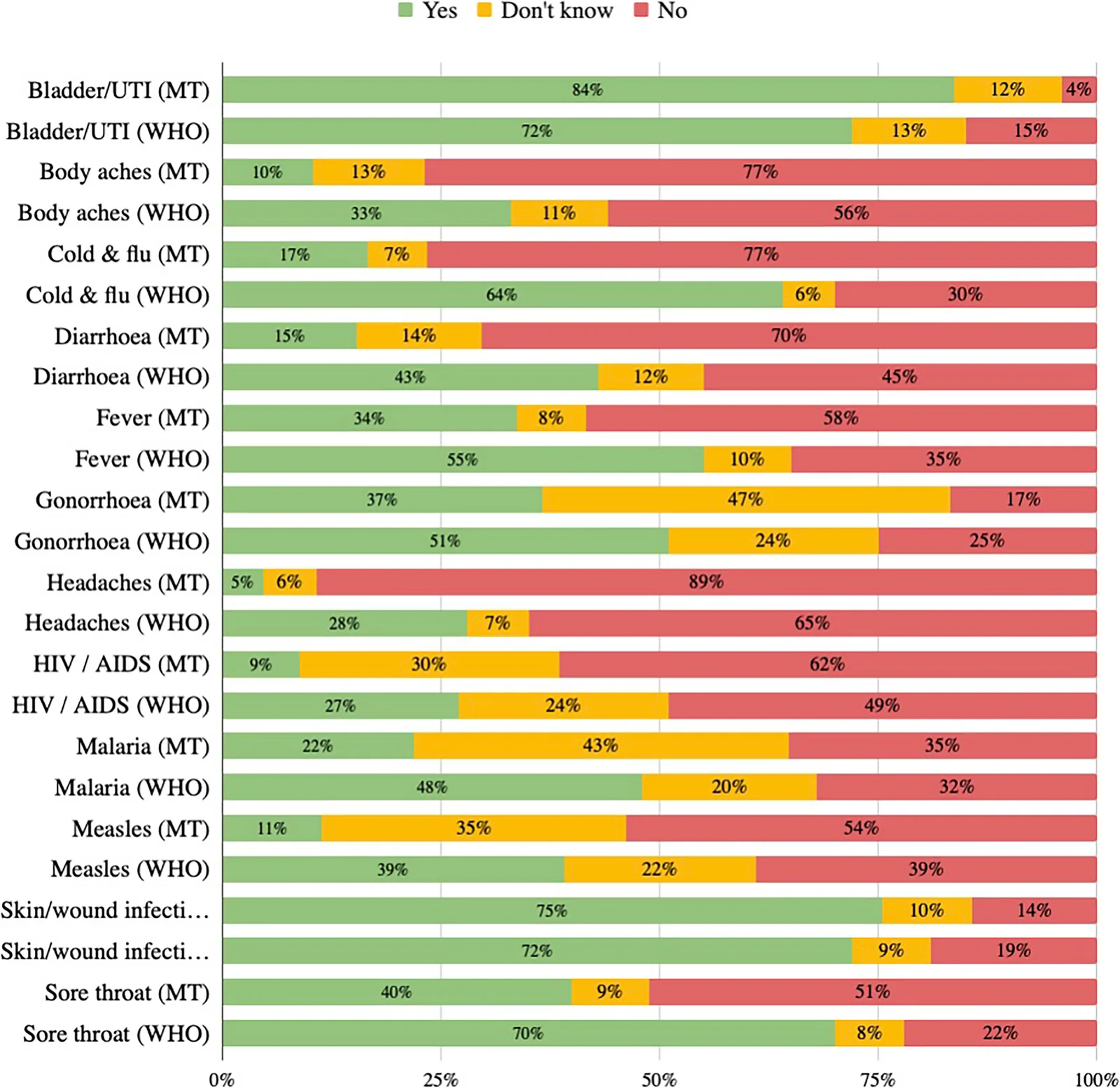
Bar chart showing public perceptions of conditions treatable with antibiotics in Malta with WHO-reported averages

The majority of respondents (95.2%, *n* = 453) correctly indicated that antibiotics should be taken until the full course is completed. A small proportion (4.6%, *n* = 22) selected the option indicating they would stop treatment when they feel better. Although the first reflects knowledge, the latter represents a declared practice and is therefore reported in the practices section. Most respondents (90.8%, *n* = 432) correctly identified as false the statement that it is acceptable to use antibiotics prescribed to others, even for the same illness, while 5.9% (*n* = 28) thought it is acceptable to use someone else’s antibiotics. When asked which conditions they thought could be treated with antibiotics (Fig. 1), 83.6% (*n* = 398) correctly identified urinary tract infections. However, smaller proportions incorrectly believed that antibiotics could be used for colds and flu (16.6%, *n* = 79) and headaches (4.6%, *n* = 22). Misconceptions were also evident for fevers (33.6%, *n* = 160) and sore throats (39.9%, *n* = 190).

### Knowledge on AMR and its implications

Totals in Supplementary Material 3 may not sum to 476 due to item-level missing data, as SPSS automatically excludes respondents with missing values from each individual analysis.

**Fig. 2:**
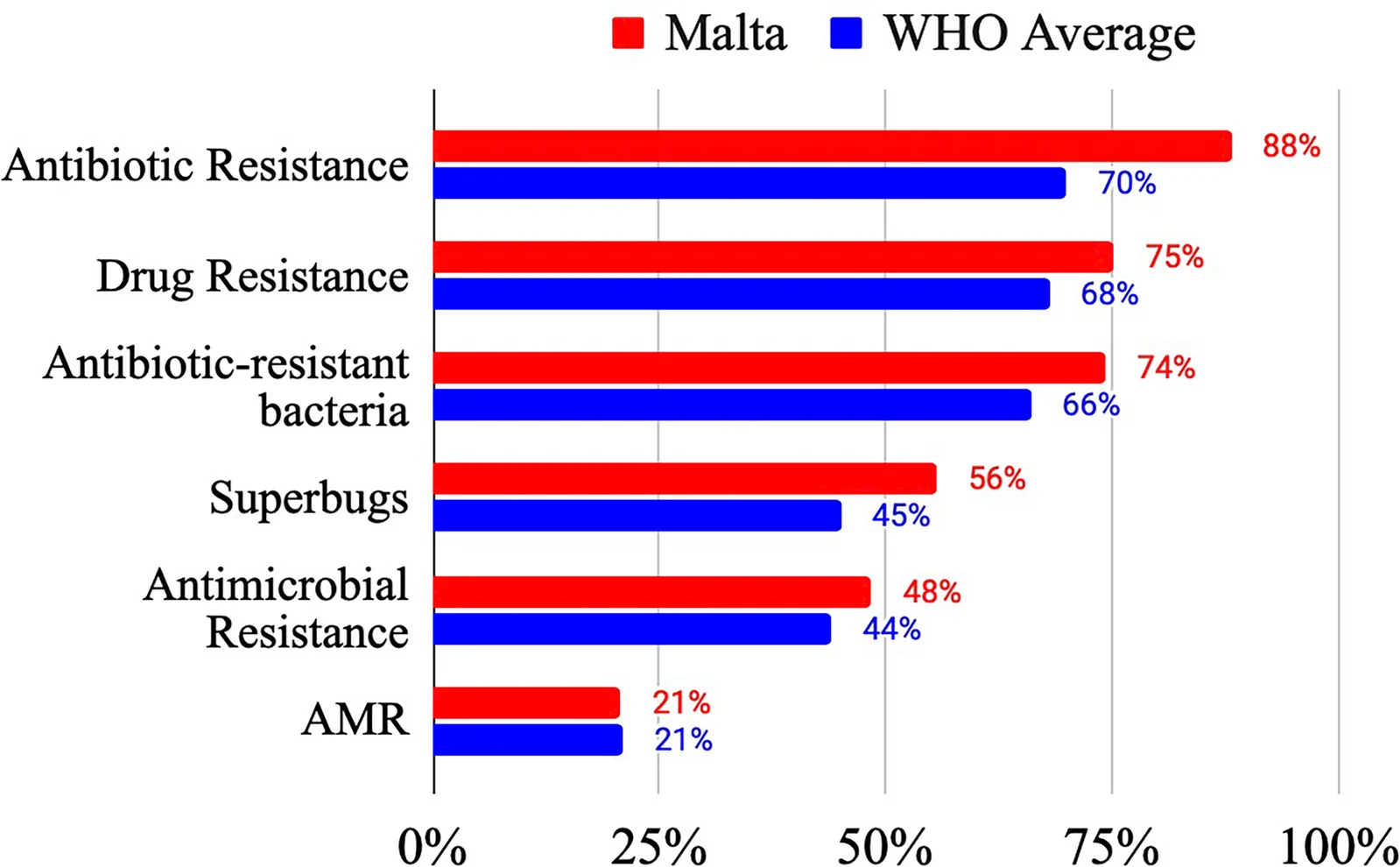
Bar chart showing awareness levels of common AMR terms Malta with WHO-reported averages

With regards to familiarity with key terms associated with AMR (Fig. 2), there was higher awareness of the term “Antibiotic Resistance” (88.0%, *n* = 419) compared to “Drug Resistance” (75.0%, *n* = 357) and “Antibiotic-Resistant Bacteria” (73.9%, *n* = 352). By contrast, many respondents were unfamiliar with “Superbugs” (56.1%, *n* = 267) and “Antimicrobial Resistance” (47.9%, *n* = 228). Awareness of the abbreviation “AMR” was particularly low (21.0%, *n* = 100).

**Fig. 3:**
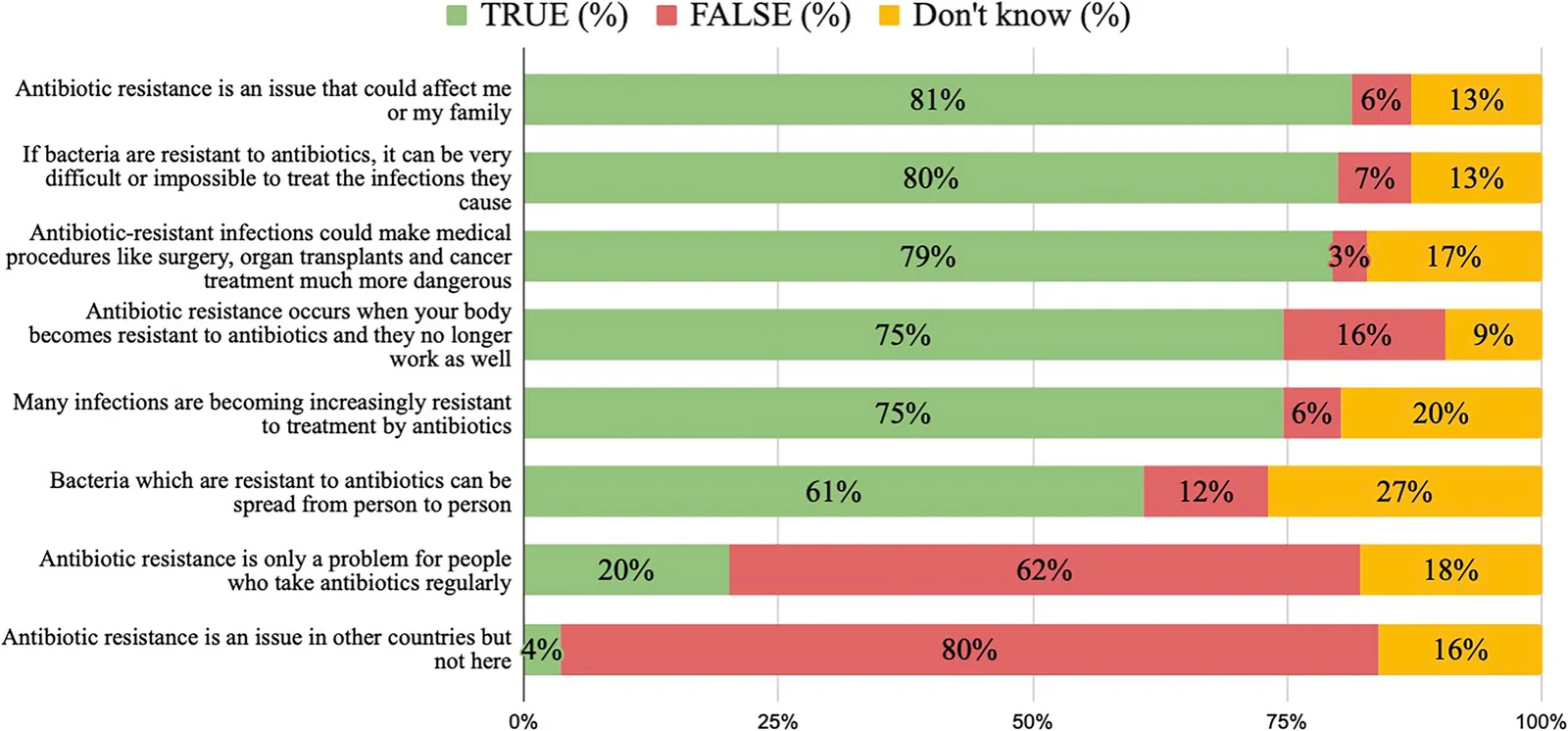
Stacked bar chart showing variations in awareness of AMR implications in Malta

Respondents were then presented with various statements concerning AMR to assess their understanding of its implications. Three quarters (75.0%, *n* = 357) were aware that bacteria are becoming increasingly resistant to antibiotic treatments. A similar proportion recognised that AMR makes infections harder to treat (80.0%, *n* = 381) and complicates medical procedures (79%, *n* = 376). Most respondents (81.1%, *n* = 386) also recognised that AMR could personally affect them or their family (Fig. 3).

**Fig. 4:**
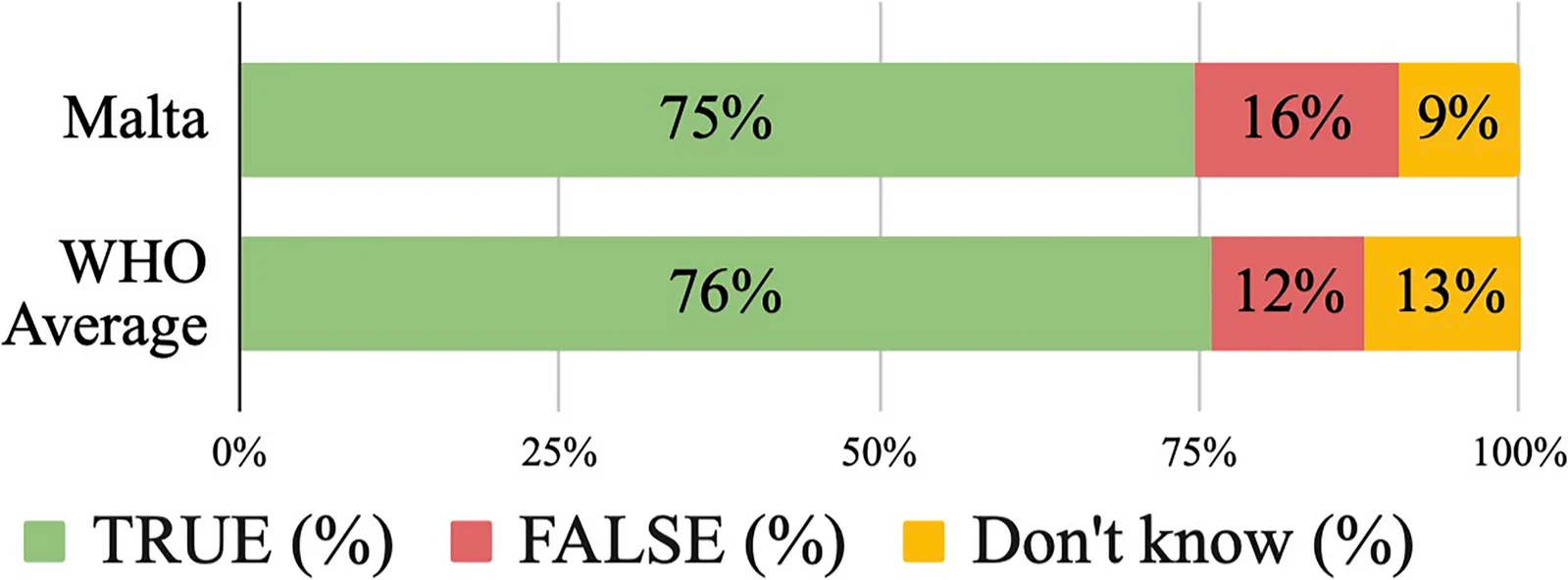
Stacked bar chart showing responses to the statement: “AMR occurs when your body becomes resistant to antibiotics, and they no longer work as well”

Only 4.0% (*n* = 19) thought that AMR is not an issue in Malta, while 60.9% (*n* = 290) recognised that antibiotic-resistant bacteria can be transmitted from person to person. By contrast, three quarters (75.0%, *n* = 357) incorrectly believed that AMR occurs when the body becomes resistant to antibiotics and they no longer work as well (Fig. 4).

### Perceptions on attitudes toward antibiotic use and associated policy measures

Most respondents recognised the importance of regular hand hygiene (95.2%, *n* = 453). A large proportion also opposed the reuse of antibiotics for different illnesses (85.1%, *n* = 405). In addition, most supported keeping children’s vaccinations up to date (95.2%, *n* = 453).

Regarding the acceptability of reusing previously prescribed antibiotics, 73.3% (*n* = 349) disagreed that it is acceptable to do so, while 23.7% (*n* = 113) considered it acceptable.

**Fig. 5:**
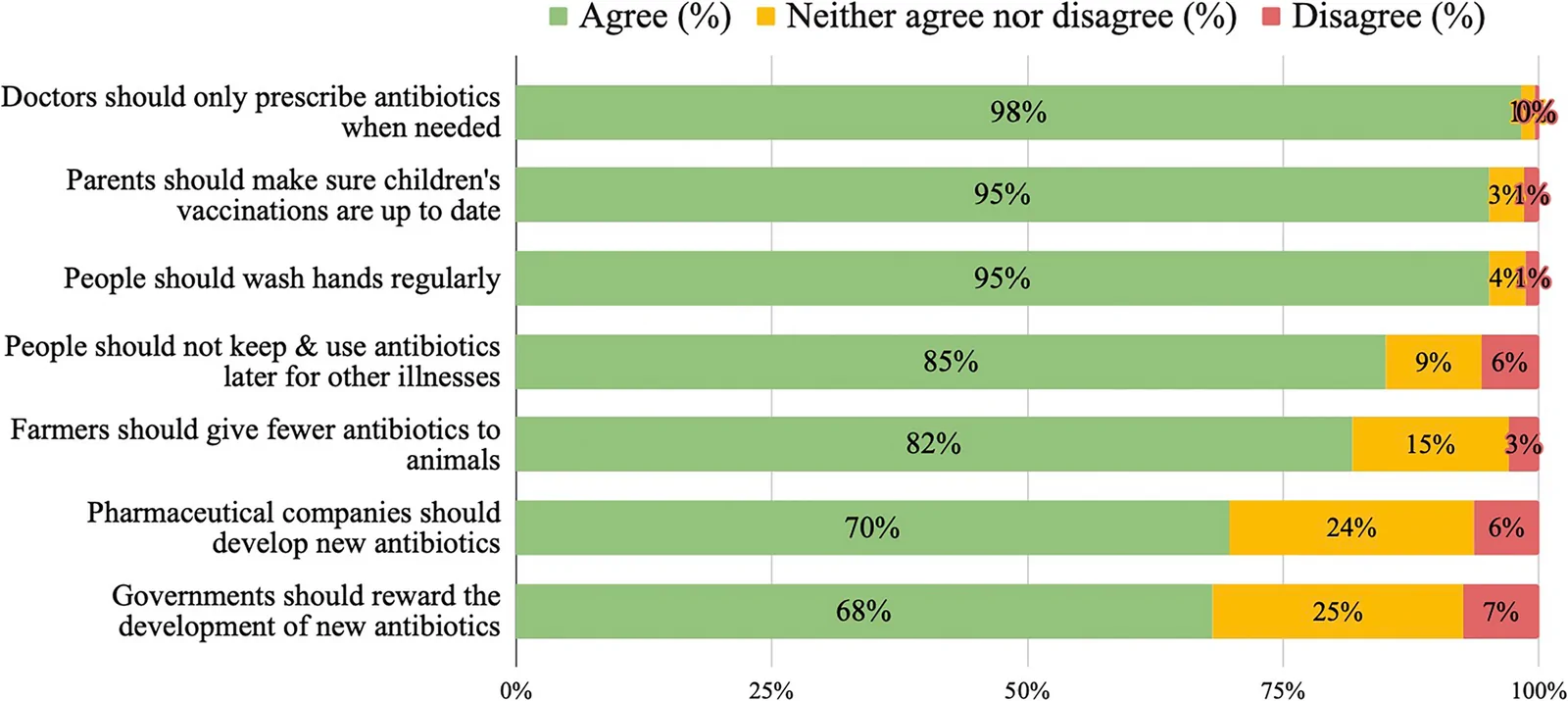
Stacked bar chart showing attitudes toward antibiotic use and associated policy measures

There was agreement that farmers should administer fewer antibiotics to animals (81.7%, *n* = 389), and nearly all respondents agreed that antibiotics should only be used when prescribed by doctors when needed (97.3%, *n* = 463) (Fig. 5).

There were notable neutrality and some disagreement regarding whether governments should reward the development of new antibiotics (24.6% neutral, *n* = 117) and whether pharmaceutical companies should take responsibility for developing new antibiotics (23.9% neutral, *n* = 114).

### Perceptions on responsibility for addressing AMR

**Fig. 6:**
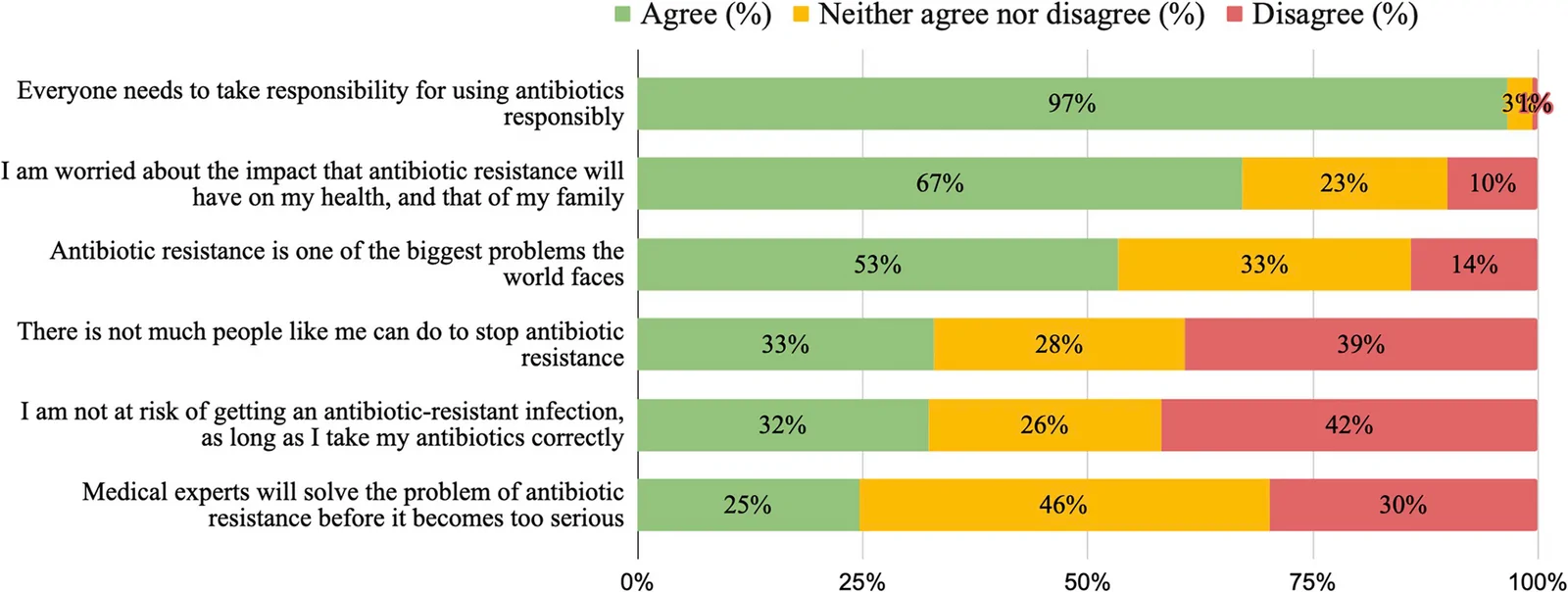
Stacked bar chart showing community perceptions of responsibility for addressing AMR challenges

The majority of respondents (96.7%, *n* = 460) agreed that everyone needs to take responsibility for using antibiotics responsibly (Fig. 6). However, concerns about the impact of AMR on personal and family health were notably lower, with fewer respondents expressing concern (67.2%, *n* = 320). Over half of the respondents view AMR as one of the biggest global challenges (53.4%, *n* = 254).

Opinions are divided on the ability to stop AMR, with 32.9% (*n* = 157) feeling limited in their personal actions to combat resistance. A third of respondents (32.3%, *n* = 154) believed they are not at risk of antibiotic-resistant infections if they use antibiotics correctly. Only 24.5% (*n* = 117) agreed that medical experts will solve the problem of AMR before it becomes too serious. A further 29.9% (*n* = 142) disagreed with this statement, while 45.6% (*n* = 217) were unsure.

### Demographic differences on attitudes toward antibiotic use and associated policy measures

Table 2 presents the results of the Kruskal–Wallis H and Mann–Whitney U tests (in the case of Gender) used to examine demographic differences in attitudes toward antibiotic use and related policy measures. The findings for each demographic variable are discussed separately in Sections "Age", "Education Level", "Gender", "Household Income Level", "Region" .

**Table 2:** Results of Kruskal-Wallis H / Mann-Whitney U tests examining demographic differences on attitudes toward antibiotic use and associated policy measures

Statement	Age Group	Education Level	Gender	Household Income	Region
People should use antibiotics only when they are prescribed by a doctor or nurse	17.750 (**< 0.001**)	6.222 (**0.045)**	27192.5 (0.975)	4.322 (0.364)	3.890 (0.565)
Farmers should give fewer antibiotics to food-producing animals	39.619 (**< 0.001**)	5.080 (0.079)	30419.0 (**0.010**)	6.791 (0.147)	10.583 (0.060)
People should not keep antibiotics and use them later for other illnesses	15.423 (**< 0.001**)	2.161 (0.339)	28756.0 (0.204)	4.893 (0.298)	9.367 (0.095)
Parents should make sure all of their children’s vaccinations are up-to-date	10.624 (**0.005**)	2.881 (0.237)	28456.0 (0.155)	2.819 (0.589)	3.589 (0.610)
People should wash their hands regularly	8.824 (**0.012**)	1.716 (0.424)	27496.5 (0.744)	1.128 (0.890)	1.644 (0.896)
Doctors should only prescribe antibiotics when they are needed	11.751 (**0.003**)	0.287 (0.866)	27646.0 (0.508)	12.233 (**0.016**)	4.593 (0.468)
Governments should reward the development of new antibiotics	5.858 (0.053)	0.591 (0.744)	23684.5 (**0.011**)	1.939 (0.747)	2.758 (0.737)
Pharmaceutical companies should develop new antibiotics	17.249 (**< 0.001**)	0.727 (0.695)	23835.0 (**0.015**)	0.969 (0.915)	6.936 (0.225)

Test Statistic (*p*-value)

Note: Bold values indicate statistically significant differences at *p* < 0.05

#### Age

To examine demographic influences on the Likert-scale statements by age, respondents’ ages were grouped into three categories: 18–34 (Young), 35–54 (Middle-Aged), and 55+ (Senior).

A Kruskal-Wallis H test reveals significantly different views on the statement “People should use antibiotics only when they are prescribed by a doctor or nurse” across age groups.

(H = 17.750, *p* < 0.001). Bonferroni-adjusted pairwise comparison tests show that respondents aged 18–34 years have significantly lower agreement compared to those in the 35–54 age group (Adj. *p* = 0.001, Mean Ranks: 223.48 versus 250.33) as well as those in the 55 + age group (Adj. *p* = 0.005, Mean Ranks: 223.48 versus 256.77). No significant differences were found among the two older age groups, suggesting consistent attitudes towards antibiotic use.

Similarly, older respondents show stronger agreement with reducing antibiotic use in food-producing animals (H = 39.619, *p* < 0.001). Individuals aged 18–34 years have significantly lower agreement compared to those in the 35–54 age group (Adj. *p* < 0.001, Mean Ranks: 204.18 versus 261.94) as well as those in the 55 + age group (Adj. *p* < 0.001, Mean Ranks: 204.18 versus 290.66).

Older respondents are also more likely to agree on not keeping leftover antibiotics for later use (H = 15.423, *p* < 0.001). Individuals aged 18–34 years have significantly lower agreement compared to those in the 35–54 age group (Adj. *p* = 0.003, Mean Ranks: 217.30 versus 254.57) as well as those in the 55 + age group (Adj. *p* = 0.007, Mean Ranks: 217.30 versus 266.09).

There is higher agreement among older respondents on the importance of vaccinations (H = 10.624, *p* = 0.005). Pairwise comparisons only show significant differences between the youngest group (18–34) and the oldest group (55+) (Adj. *p* = 0.003, Mean Ranks: 229.45 versus 266.96).

Older respondents also show greater agreement on the importance of regular hand-washing (H = 8.824, *p* = 0.012), with significant differences being found between the youngest and both other age groups. Individuals aged 18–34 years have significantly lower agreement compared to those in the 35–54 age group (Adj. *p* = 0.046, Mean Ranks: 227.33 versus 246.49) as well as those in the 55 + age group (Adj. *p* = 0.050, Mean Ranks: 227.33 versus 254.41).

Moreover, significant age differences were found on the agreement on prescribing antibiotics only when needed (H = 11.751, *p* = 0.003). Pairwise comparisons revealed significant differences between the 18–34 and 35–54 age groups, with the latter group showing higher agreement (Adj. *p* = 0.003, Mean Ranks: 228.57 versus 248.56).

No significant age differences were found on the statement “Governments should reward the development of new antibiotics” (H = 5.858, *p* = 0.053). The same cannot be said for the statement “Pharmaceutical companies should develop new antibiotics” (H = 17.249, *p* < 0.001), with significant differences being found between the youngest and both other age groups. Individuals aged 18–34 years have significantly lower agreement compared to those in the 35–54 age group (Adj. *p* = 0.041, Mean Ranks: 216.26 versus 248.00) as well as those in the 55 + age group (Adj. *p* < 0.001, Mean Ranks: 216.26 versus 288.93).

#### Education level

To examine demographic influences on the Likert-scale statements by education level, respondents’ levels were grouped into three categories: Low Education (12th grade or less, some college credit, high school graduate with diploma), Medium Education (technical/vocational training, Bachelor’s degree), and High Education (Master’s degree, Doctorate degree).

Individuals with different education levels have significantly different perceptions on whether people should use antibiotics only when they are prescribed (H = 6.222, *p* = 0.045). Pairwise comparison tests indicated that individuals with low education have significantly lower agreement than those with medium education (Adj. *p* = 0.038, Mean Ranks = 223.64 versus 247.59). The Low-High pair (Adj. *p* = 0.267) and the Medium-High pair (Adj. *p* = 0.799) did not yield significant results.

The other results for education level show consistency across groups, indicating uniform attitudes toward antibiotic use and resistance.

#### Gender

Males and females have significantly different agreement levels on the statements “Farmers should give fewer antibiotics to food-producing animals” (U = 30419.0, *p* = 0.010), “Governments should reward the development of new antibiotics” (U = 23684.5, *p* = 0.011), and “Pharmaceutical companies should develop new antibiotics” (U = 23835.0, *p* = 0.015). Specifically, females agree more on the former statement while males agree more on the latter two statements, with the respective mean ranks being as follows: F: 249.73, M: 221.74; F: 226.10, M: 257.00; F: 226.63, M: 256.21.

#### Household income level

To examine demographic influences on the Likert-scale statements by household income level, respondents’ levels were grouped into five categories: Up to EUR24,999, EUR25,000–49,999, EUR50,000–99,999, EUR100,000+, and prefer not to say.

Individuals with different household income brackets have significantly varying views on whether doctors should only prescribe antibiotics when they are needed (H = 12.233, *p* = 0.016). It was found through pairwise comparison tests that those earning up to EUR24,999 have significantly lower agreement than those earning between EUR50,000 and EUR99,999 (Adj. *p* = 0.011, Mean Ranks: 213.98 versus 245.39). The other pairs did not yield significant results.

#### Region

No significant differences in region perceptions were found on the statements regarding attitudes toward antibiotic use and associated policy measures.

### Demographic differences on responsibility perception for addressing AMR

Table 3 presents the results of the Kruskal–Wallis H and Mann–Whitney U tests (in the case of Gender) used to examine demographic differences in perceptions on responsibility for addressing AMR. The findings for each demographic variable are discussed separately in Sections "Age", "Education Level", "Gender", "Household Income Level", "Region" .

**Table 3:** Results of Kruskal-Wallis H / Mann-Whitney U tests examining demographic differences on responsibility for addressing AMR

Statement	Age Group	Education Level	Gender	Household Income	Region
Antibiotic resistance is one of the biggest problems the world faces	15.246 (**< 0.001**)	2.279 (0.320)	29102.5 (0.183)	6.893 (0.142)	8.051 (0.153)
Medical experts will solve the problem of antibiotic resistance before it becomes too serious	9.873 (**0.007**)	9.209 (**0.010**)	26435.5 (0.574)	15.494 (**0.004**)	6.483 (0.262)
Everyone needs to take responsibility for using antibiotics responsibly	5.354 (0.069)	1.165 (0.559)	27660.5 (0.632)	3.786 (0.436)	7.406 (0.192)
There is not much people like me can do to stop antibiotic resistance	4.303 (0.116)	4.133 (0.127)	26072.5 (0.424)	8.540 (0.074)	5.936 (0.313)
I am worried about the impact that antibiotic resistance will have on my health, and that of my family	5.221 (0.074)	1.791 (0.408)	28773.0 (0.268)	7.302 (0.121)	5.561 (0.351)
I am not at risk of getting an antibiotic-resistant infection, as long as I take my antibiotics correctly	2.639 (0.267)	10.421 (**0.005**)	28778.5 (0.277)	12.907 (**0.012**)	11.805 (**0.038**)

Test Statistic (*p*-value)

Bold values indicate statistical significance at *p* < 0.05

#### Age

Older individuals are more likely to perceive antibiotic resistance as one of the biggest problems the world faces (H = 15.246, *p* < 0.001). Bonferroni-adjusted pairwise comparison tests showed that respondents aged 18–34 years have significantly lower agreement compared to those in the 35–54 age group (Adj. *p* = 0.004, Mean Ranks: 214.05 versus 256.37) as well as those in the 55 + age group (Adj. *p* = 0.006, Mean Ranks: 214.05 versus 272.23).

Confidence in medical experts solving the problem of antibiotic resistance before it becomes too serious varied significantly with age, with the senior respondents expressing significantly more trust (H = 9.873, *p* = 0.007). Pairwise comparisons revealed that respondents aged 55 + have significantly higher agreement compared to those in the 18–34 age group (Adj. *p* = 0.009, Mean Ranks: 285.97 versus 231.21) as well as those in the 35–54 age group (Adj. *p* = 0.010, Mean Ranks: 285.97 versus 231.02).

No statistically significant differences were found among age groups on the statements “Everyone needs to take responsibility for using antibiotics responsible” (H = 5.354, *p* = 0.069), “There is not much people like me can do to stop antibiotic resistance” (H = 4.303, *p* = 0.116), “I am worried about the impact that antibiotic resistance will have on my health, and that of my family” (H = 5.221, *p* = 0.074), and “I am not at risk of getting an antibiotic-resistant infection, as long as I take my antibiotics correctly” (H = 2.639, *p* = 0.267).

#### Education level

Individuals with different education levels have significantly different perceptions on whether medical experts will solve the problem of AMR (H = 9.209, *p* = 0.010). In particular, individuals with low education have significantly higher agreement than those with high education (Adj. *p* = 0.009, Mean Ranks: 268.58 versus 221.89). The Low-Medium pair (Adj. *p* = 0.404) and the Medium-High pair (Adj. *p* = 0.340) did not yield significant results.

Additionally, individuals with different education levels do not equally believe they are at risk of getting an antibiotic-resistant infection, as long as they take their antibiotics correctly (H = 10.421, *p* = 0.005). Pairwise comparisons indicated that individuals with low education have significantly higher agreement than those with high education (Adj. *p* = 0.004, Mean Ranks: 273.62 versus 221.46). The Low-Medium pair (Adj. *p* = 0.177) and the Medium-High pair (Adj. *p* = 0.508) did not yield significant results.

#### Gender

No significant differences in gender perceptions were found on the statements regarding the responsibility for addressing the challenge of AMR.

#### Household income level

Individuals with different household income levels have significantly different perceptions on whether medical experts will solve the problem of AMR (H = 15.494, *p* = 0.004) and whether they are not at risk of getting an antibiotic-resistant infection, as long as they take antibiotics correctly (H = 12.907, *p* = 0.012).

For the former statement, individuals earning up to EUR24,999 have significantly higher agreement than those earning EUR25,000–49,999 (Adj. *p* = 0.001, Mean Ranks: 294.62 versus 217.98) and those earning EUR50,000–99,999 (Adj. *p* = 0.027, Mean Ranks: 294.62 versus 232.67). The other pairs did not yield significant results.

For the latter statement, individuals earning up to EUR24,999 have significantly higher agreement than those earning EUR25,000–49,999 (Adj. *p* = 0.010, Mean Ranks: 295.58 versus 227.77) and those earning EUR50,000–99,999 (Adj. *p* = 0.010, Mean Ranks: 295.58 versus 225.51). The other pairs did not yield significant results.

#### Region

Individuals living in different regions do not equally believe they are at risk of getting an antibiotic-resistant infection, as long as they take their antibiotics correctly (H = 11.805, *p* = 0.038). However, after adjustment for multiple comparisons using the Bonferroni correction, none of the pairwise differences remained statistically significant. This is likely due to the use of six regional groups with uneven sample sizes, which substantially reduces statistical power, especially once the Bonferroni correction is applied, making it harder to detect significant pairwise differences even when an overall effect is observed.

### Demographic differences in knowledge on antibiotic use and AMR

Knowledge-related items included categorical responses indicating correct, incorrect, or uncertain behaviours (e.g., whether colds/flu can be treated with antibiotics, whether it is acceptable to use leftover or shared antibiotics, and when to stop an antibiotic course). Tables 4, 5, 6, 7 and 8 summarise how these responses varied across demographic groups of age, education, gender, household income, and region, respectively. In these tables, the correct response for each statement is highlighted in bold font.

**Table 4:** Results examining age differences on knowledge on antibiotic use and AMR

			Age Group (%)			Test Statistic (p-value)
			18–34	35–54	55+	
HIV/AIDS	Yes		12.0	4.8	9.4	8.050 (0.090)
	**No**		58.7	66.8	56.3	
	Don’t Know		29.3	28.3	34.4	
Gonorrhoea	**Yes**		38.2	35.3	34.4	0.729 (0.948)
	No		16.4	17.6	15.6	
	Don’t Know		45.3	47.1	50.0	
Bladder Infection or Urinary Tract Infection (UTI)	**Yes**		80.9	86.6	84.4	4.803 (0.308)
	No		3.6	3.7	6.3	
	Don’t Know		15.6	9.6	9.4	
Diarrhoea	Yes		23.1	8.6	7.8	35.697 (**< 0.001**)
	**No**		57.3	81.3	84.4	
	Don’t Know		19.6	10.2	7.8	
Cold and Flu	Yes		24.9	7.0	15.6	31.285 (**< 0.001**)
	**No**		65.8	88.2	81.3	
	Don’t Know		9.3	4.8	3.1	
Fever	Yes		40.0	29.4	23.4	12.474 (**0.014**)
	**No**		52.0	61.0	73.4	
	Don’t Know		8.0	9.6	3.1	
Malaria	Yes		27.1	15.5	21.9	9.549 (**0.049**)
	**No**		30.7	41.2	34.4	
	Don’t Know		42.2	43.3	43.8	
Measles	Yes		17.8	4.8	7.8	34.375 (**< 0.001**)
	**No**		40.9	66.3	62.5	
	Don’t Know		41.3	28.9	29.7	
Skin or Wound Infection	**Yes**		73.3	78.6	73.4	2.830 (0.587)
	No		14.7	13.9	14.1	
	Don’t Know		12.0	7.5	12.5	
Sore Throat	Yes		45.3	36.4	31.3	8.873 (0.064)
	**No**		44.4	55.6	62.5	
	Don’t Know		10.2	8.0	6.3	
Body Aches	Yes		16.9	4.3	4.7	28.224 (**< 0.001**)
	**No**		66.7	86.1	85.9	
	Don’t Know		16.4	9.6	9.4	
Headaches	Yes		9.3	0.0	1.6	34.240* (**< 0.001**)
	**No**		82.7	96.8	90.6	
	Don’t Know		8.0	3.2	7.8	
When do you think you should stop taking antibiotics once you’ve begun treatment?	**After Taking All Antibiotics**		91.6	97.9	100.0	15.695* (**0.003**)
	When You Feel Better		8.0	2.1	0.0	
	Don’t Know		0.4	0.0	0.0	
It’s okay to use antibiotics that were given to a friend or family member, as long as they were used to treat the same illness	True		8.0	5.3	0.0	11.421* (**0.022**)
	**False**		87.6	92.5	96.9	
	Don’t Know		4.4	2.1	3.1	
It’s okay to buy the same antibiotics, or request these from a doctor, if you’re sick and they helped you get better when you had the same symptoms before	True		28.9	17.1	25.0	10.308 (**0.036**)
	**False**		67.6	79.7	75.0	
	Don’t Know		3.6	3.2	0.0	
Antibiotic resistance occurs when your body becomes resistant to antibiotics and they no longer work as well	True		72.0	75.9	79.7	6.693 (0.153)
	**False**		16.9	17.6	7.8	
	Don’t Know		11.1	6.4	12.5	
Many infections are becoming increasingly resistant to treatment by antibiotics	**True**		68.0	81.8	76.6	13.355 (**0.010**)
	False		6.7	3.2	9.4	
	Don’t Know		25.3	15.0	14.1	
If bacteria are resistant to antibiotics, it can be very difficult or impossible to treat the infections they cause	**True**		76.4	87.7	70.3	15.053 (**0.005**)
	False		7.1	4.8	14.1	
	Don’t Know		16.4	7.5	15.6	
Antibiotic resistance is an issue that could affect me or my family	**True**		78.7	84.5	81.3	5.981 (0.201)
	False		5.8	7.0	3.1	
	Don’t Know		15.6	8.6	15.6	
Antibiotic resistance is an issue in other countries but not here	True		4.9	1.6	4.7	7.386 (0.117)
	**False**		77.3	86.1	75.0	
	Don’t Know		17.8	12.3	20.3	
Antibiotic resistance is only a problem for people who take antibiotics regularly	True		21.3	20.3	15.6	3.164 (0.531)
	**False**		62.7	62.0	59.4	
	Don’t Know		16.0	17.6	25.0	
Bacteria which are resistant to antibiotics can be spread from person to person	**True**		57.3	65.2	60.9	5.057 (0.282)
	False		12.0	10.7	17.2	
	Don’t Know		30.7	24.1	21.9	
Antibiotic-resistant infections could make medical procedures like surgery, organ transplants and cancer treatment much more dangerous	**True**		78.7	81.8	75.0	13.925 (**0.008**)
	False		2.2	2.1	10.9	
	Don’t Know		19.1	16.0	14.1	

*Likelihood Ratio Test instead of Pearson Chi-Square Test Used

**Table 5:** Results examining education level differences on knowledge on antibiotic use and AMR

			Education Level (%)			Test Statistic (p-value)
			Low	Medium	High	
HIV/AIDS	Yes		12.1	9.2	7.1	3.617 (0.460)
	**No**		54.5	62.5	64.0	
	Don’t Know		33.3	28.3	28.9	
Gonorrhoea	**Yes**		31.3	30.3	43.1	9.857 (**0.043**)
	No		14.1	18.4	16.9	
	Don’t Know		54.5	51.3	40.0	
Bladder Infection or Urinary Tract Infection (UTI)	**Yes**		80.8	80.3	87.1	7.191 (0.126)
	No		7.1	5.3	1.8	
	Don’t Know		12.1	14.5	11.1	
Diarrhoea	Yes		10.1	13.2	19.1	5.956 (0.202)
	**No**		76.8	73.0	65.8	
	Don’t Know		13.1	13.8	15.1	
Cold and Flu	Yes		20.2	15.1	16.0	2.125 (0.713)
	**No**		72.7	79.6	76.4	
	Don’t Know		7.1	5.3	7.6	
Fever	Yes		37.4	27.6	36.0	3.703 (0.448)
	**No**		55.6	63.8	56.0	
	Don’t Know		7.1	8.6	8.0	
Malaria	Yes		27.3	19.7	20.9	14.375 (**0.006**)
	**No**		21.2	34.2	42.2	
	Don’t Know		51.5	46.1	36.9	
Measles	Yes		11.1	13.8	9.8	4.134 (0.388)
	**No**		47.5	55.3	55.6	
	Don’t Know		41.4	30.9	34.7	
Skin or Wound Infection	**Yes**		71.7	73.7	78.2	4.148 (0.386)
	No		19.2	15.8	11.1	
	Don’t Know		9.1	10.5	10.7	
Sore Thorat	Yes		46.5	40.8	36.4	3.992 (0.407)
	**No**		46.5	52.0	52.9	
	Don’t Know		7.1	7.2	10.7	
Body Aches	Yes		14.1	12.5	7.1	5.649 (0.227)
	**No**		72.7	77.0	78.7	
	Don’t Know		13.1	10.5	14.2	
Headaches	Yes		9.1	3.9	3.1	6.799 (0.147)
	**No**		83.8	91.4	90.2	
	Don’t Know		7.1	4.6	6.7	
When do you think you should stop taking antibiotics once you’ve begun treatment?	**After Taking All Antibiotics**		91.9	96.7	95.6	4.589* (0.332)
	When You Feel Better		8.1	3.3	4.0	
	Don’t Know		0.0	0.0	0.4	
It’s okay to use antibiotics that were given to a friend or family member, as long as they were used to treat the same illness	True		7.1	7.9	4.0	11.552 (**0.021**)
	**False**		84.8	90.1	93.8	
	Don’t Know		8.1	2.0	2.2	
It’s okay to buy the same antibiotics, or request these from a doctor, if you’re sick and they helped you get better when you had the same symptoms before	True		39.4	23.0	17.3	24.126* (**< 0.001**)
	**False**		55.6	76.3	79.1	
	Don’t Know		5.1	0.7	3.6	
Antibiotic resistance occurs when your body becomes resistant to antibiotics and they no longer work as well	True		85.9	73.0	70.7	18.611 (**< 0.001**)
	**False**		2.0	18.4	20.4	
	Don’t Know		12.1	8.6	8.9	
Many infections are becoming increasingly resistant to treatment by antibiotics	**True**		62.6	73.0	80.9	12.414 (**0.015**)
	False		8.1	5.9	4.4	
	Don’t Know		29.3	21.1	14.7	
If bacteria are resistant to antibiotics, it can be very difficult or impossible to treat the infections they cause	**True**		69.7	80.9	84.0	9.738 (**0.045**)
	False		10.1	5.9	6.7	
	Don’t Know		20.2	13.2	9.3	
Antibiotic resistance is an issue that could affect me or my family	**True**		77.8	79.6	84.0	7.093 (0.131)
	False		6.1	3.9	7.1	
	Don’t Know		16.2	16.4	8.9	
Antibiotic resistance is an issue in other countries but not here	True		7.1	1.3	3.6	21.751 (**< 0.001**)
	**False**		67.7	78.9	87.1	
	Don’t Know		25.3	19.7	9.3	
Antibiotic resistance is only a problem for people who take antibiotics regularly	True		27.3	19.1	17.8	16.356 (**0.003**)
	**False**		46.5	61.2	69.3	
	Don’t Know		26.3	19.7	12.9	
Bacteria which are resistant to antibiotics can be spread from person to person	**True**		49.5	62.5	64.9	7.264 (0.123)
	False		15.2	11.2	11.6	
	Don’t Know		35.4	26.3	23.6	
Antibiotic-resistant infections could make medical procedures like surgery, organ transplants and cancer treatment much more dangerous	**True**		71.7	78.9	83.1	6.572 (0.160)
	False		4.0	4.6	2.2	
	Don’t Know		24.2	16.4	14.7	

*Likelihood Ratio Test instead of Pearson Chi-Square Test Used

**Table 6:** Results examining gender differences on knowledge on antibiotic use and AMR

			Gender (%)		Test Statistic (p-value)
			Males	Females	
HIV/AIDS	Yes		5.8	10.9	4.266 (0.119)
	**No**		61.8	61.4	
	Don’t Know		32.5	27.7	
Gonorrhoea	**Yes**		37.2	36.1	1.068 (0.586)
	No		14.7	18.2	
	Don’t Know		48.2	45.6	
Bladder Infection or Urinary Tract Infection (UTI)	**Yes**		78.5	87.0	16.389 (**< 0.001**)
	No		2.1	5.3	
	Don’t Know		19.4	7.7	
Diarrhoea	Yes		18.3	13.3	25.205 (**< 0.001**)
	**No**		58.6	78.2	
	Don’t Know		23.0	8.4	
Cold and Flu	Yes		21.5	13.3	10.405 (**0.006**)
	**No**		69.1	81.8	
	Don’t Know		9.4	4.9	
Fever	Yes		38.2	30.5	7.236 (**0.027**)
	**No**		51.3	63.2	
	Don’t Know		10.5	6.3	
Malaria	Yes		18.8	23.9	1.682 (0.431)
	**No**		36.6	34.4	
	Don’t Know		44.5	41.8	
Measles	Yes		11.5	11.2	3.011 (0.222)
	**No**		49.2	56.8	
	Don’t Know		39.3	31.9	
Skin or Wound Infection	**Yes**		69.1	79.6	7.619 (**0.022**)
	No		16.8	12.6	
	Don’t Know		14.1	7.7	
Sore Thorat	Yes		38.7	40.7	5.583 (0.061)
	**No**		48.7	53.0	
	Don’t Know		12.6	6.3	
Body Aches	Yes		13.1	8.4	9.563 (**0.008**)
	**No**		69.6	81.8	
	Don’t Know		17.3	9.8	
Headaches	Yes		2.8	7.3	19.714 (**< 0.001**)
	**No**		94.4	81.7	
	Don’t Know		2.8	11.0	
When do you think you should stop taking antibiotics once you’ve begun treatment?	**After Taking All Antibiotics**		91.6	97.5	9.303* (**0.010**)
	When You Feel Better		7.9	2.5	
	Don’t Know		0.5	0.0	
It’s okay to use antibiotics that were given to a friend or family member, as long as they were used to treat the same illness	True		7.9	4.6	14.401 (**< 0.001**)
	**False**		85.3	94.4	
	Don’t Know		6.8	1.1	
It’s okay to buy the same antibiotics, or request these from a doctor, if you’re sick and they helped you get better when you had the same symptoms before	True		21.5	25.3	2.428 (0.297)
	**False**		74.3	72.6	
	Don’t Know		4.2	2.1	
Antibiotic resistance occurs when your body becomes resistant to antibiotics and they no longer work as well	True		68.6	78.6	6.059 (**0.048**)
	**False**		19.9	13.3	
	Don’t Know		11.5	8.1	
Many infections are becoming increasingly resistant to treatment by antibiotics	**True**		73.8	75.1	0.102 (0.951)
	False		5.8	5.6	
	Don’t Know		20.4	19.3	
If bacteria are resistant to antibiotics, it can be very difficult or impossible to treat the infections they cause	**True**		80.1	80.0	1.290 (0.525)
	False		5.8	8.1	
	Don’t Know		14.1	11.9	
Antibiotic resistance is an issue that could affect me or my family	**True**		79.1	82.8	1.132 (0.568)
	False		6.3	5.6	
	Don’t Know		14.7	11.6	
Antibiotic resistance is an issue in other countries but not here	True		4.2	3.2	0.359 (0.836)
	**False**		80.1	80.7	
	Don’t Know		15.7	16.1	
Antibiotic resistance is only a problem for people who take antibiotics regularly	True		18.8	21.1	0.374 (0.830)
	**False**		63.4	61.1	
	Don’t Know		17.8	17.9	
Bacteria which are resistant to antibiotics can be spread from person to person	**True**		62.8	59.6	5.801 (0.055)
	False		7.9	15.1	
	Don’t Know		29.3	25.3	
Antibiotic-resistant infections could make medical procedures like surgery, organ transplants and cancer treatment much more dangerous	**True**		75.9	81.8	4.311 (0.116)
	False		5.2	2.1	
	Don’t Know		18.8	16.1	

*Likelihood Ratio Test instead of Pearson Chi-Square Test Used

**Table 7:** Results examining household income level differences on knowledge on antibiotic use and AMR

			Household Income Level (%)					Test Statistic (p-value)
			<=24,999	25,000–49,999	50,000–99,999	100,000+	Prefer Not to Say	
HIV/AIDS	Yes		17.2	9.1	6.4	3.0	11.3	10.256 (0.248)
	**No**		56.9	59.1	65.6	64.2	61.3	
	Don’t Know		25.9	31.7	28.0	32.8	27.4	
Gonorrhoea	**Yes**		32.8	34.8	42.4	29.9	40.3	6.654 (0.574)
	No		15.5	20.7	12.8	17.9	14.5	
	Don’t Know		51.7	44.5	44.8	52.2	45.2	
Bladder Infection or Urinary Tract Infection (UTI)	**Yes**		74.1	83.5	88.0	82.1	85.5	10.465* (0.234)
	No		8.6	5.5	1.6	1.5	3.2	
	Don’t Know		17.2	11.0	10.4	16.4	11.3	
Diarrhoea	Yes		20.7	12.2	15.2	16.4	17.7	6.545 (0.586)
	**No**		69.0	70.1	72.8	73.1	64.5	
	Don’t Know		10.3	17.7	12.0	10.4	17.7	
Cold and Flu	Yes		27.6	14.6	11.2	19.4	19.4	17.760* (**0.023**)
	**No**		58.6	79.3	85.6	73.1	72.6	
	Don’t Know		13.8	6.1	3.2	7.5	8.1	
Fever	Yes		41.4	30.5	37.6	32.8	27.4	12.778 (0.120)
	**No**		51.7	62.8	58.4	53.7	58.1	
	Don’t Know		6.9	6.7	4.0	13.4	14.5	
Malaria	Yes		39.7	16.5	20.8	19.4	24.2	17.641 (**0.024**)
	**No**		19.0	39.6	39.2	32.8	33.9	
	Don’t Know		41.4	43.9	40.0	47.8	41.9	
Measles	Yes		17.2	11.0	9.6	13.4	8.1	13.108 (0.108)
	**No**		41.4	53.7	64.8	46.3	51.6	
	Don’t Know		41.4	35.4	25.6	40.3	40.3	
Skin or Wound Infection	**Yes**		72.4	74.4	80.0	73.1	74.2	4.138 (0.844)
	No		15.5	15.9	12.8	11.9	14.5	
	Don’t Know		12.1	9.8	7.2	14.9	11.3	
Sore Thorat	Yes		56.9	36.6	40.0	31.3	41.9	13.480 (0.096)
	**No**		37.9	55.5	51.2	59.7	43.5	
	Don’t Know		5.2	7.9	8.8	9.0	14.5	
Body Aches	Yes		19.0	9.8	8.8	10.4	6.5	11.164 (0.193)
	**No**		67.2	79.9	80.8	70.1	77.4	
	Don’t Know		13.8	10.4	10.4	19.4	16.1	
Headaches	Yes		12.1	4.9	2.4	1.5	4.8	10.246* (0.248)
	**No**		79.3	89.0	92.0	94.0	88.7	
	Don’t Know		8.6	6.1	5.6	4.5	6.5	
When do you think you should stop taking antibiotics once you’ve begun treatment?	**After Taking All Antibiotics**		91.4	93.9	97.6	98.5	93.5	14.722* (0.065)
	When You Feel Better		8.6	6.1	2.4	0.0	6.5	
	Don’t Know		0.0	0.0	0.0	1.5	0.0	
t’s okay to use antibiotics that were given to a friend or family member, as long as they were used to treat the same illness	True		6.9	6.1	4.8	7.5	4.8	7.910* (0.442)
	**False**		86.2	90.2	92.8	92.5	90.3	
	Don’t Know		6.9	3.7	2.4	0.0	4.8	
It’s okay to buy the same antibiotics, or request these from a doctor, if you’re sick and they helped you get better when you had the same symptoms before	True		32.8	21.3	20.0	19.4	33.9	13.389* (0.099)
	**False**		63.8	75.6	76.8	80.6	61.3	
	Don’t Know		3.4	3.0	3.2	0.0	4.8	
Antibiotic resistance occurs when your body becomes resistant to antibiotics and they no longer work as well	True		79.3	70.1	75.2	83.6	71.0	15.520 (**0.050**)
	**False**		6.9	22.6	16.0	6.0	17.7	
	Don’t Know		13.8	7.3	8.8	10.4	11.3	
Many infections are becoming increasingly resistant to treatment by antibiotics	**True**		70.7	75.6	76.8	71.6	74.2	5.698* (0.681)
	False		3.4	7.9	4.8	6.0	3.2	
	Don’t Know		25.9	16.5	18.4	22.4	22.6	
If bacteria are resistant to antibiotics, it can be very difficult or impossible to treat the infections they cause	**True**		77.6	81.1	80.8	79.1	79.0	8.976* (0.344)
	False		5.2	9.8	5.6	3.0	9.7	
	Don’t Know		17.2	9.1	13.6	17.9	11.3	
Antibiotic resistance is an issue that could affect me or my family	**True**		79.3	82.3	80.0	83.6	80.6	7.248* (0.510)
	False		1.7	6.1	8.8	3.0	6.5	
	Don’t Know		19.0	11.6	11.2	13.4	12.9	
Antibiotic resistance is an issue in other countries but not here	True		3.4	4.9	2.4	0.0	6.5	19.168* (**0.014**)
	**False**		65.5	78.7	86.4	85.1	82.3	
	Don’t Know		31.0	16.5	11.2	14.9	11.3	
Antibiotic resistance is only a problem for people who take antibiotics regularly	True		31.0	20.1	15.2	16.4	24.2	13.049 (0.110)
	**False**		46.6	62.8	71.2	62.7	54.8	
	Don’t Know		22.4	17.1	13.6	20.9	21.0	
Bacteria which are resistant to antibiotics can be spread from person to person	**True**		56.9	60.4	67.2	56.7	58.1	6.318 (0.612)
	False		19.0	11.6	8.0	13.4	14.5	
	Don’t Know		24.1	28.0	24.8	29.9	27.4	
Antibiotic-resistant infections could make medical procedures like surgery, organ transplants and cancer treatment much more dangerous	**True**		69.0	83.5	80.0	79.1	77.4	9.979* (0.266)
	False		5.2	3.7	1.6	1.5	6.5	
	Don’t Know		25.9	12.8	18.4	19.4	16.1	

*Likelihood Ratio Test instead of Pearson Chi-Square Test Used

**Table 8:** Results examining region differences on knowledge on antibiotic use and AMR

		Region						Test Statistic (*p*-value)
		Northern	Northern Harbour	Southern Harbour	South Western	Western	Gozo and Comino	
HIV/AIDS	Yes	12.2	7.0	11.9	7.7	6.9	15.4	8.117 (0.617)
	**No**	55.1	64.3	52.4	60.0	67.8	69.2	
	Don’t Know	32.7	28.7	35.7	32.3	25.3	15.4	
Gonorrhoea	**Yes**	32.7	40.9	40.5	27.7	36.8	38.5	11.284 (0.336)
	No	10.2	19.3	16.7	18.5	18.4	15.4	
	Don’t Know	57.1	39.8	42.9	53.8	44.8	46.2	
Bladder Infection or Urinary Tract Infection (UTI)	**Yes**	83.7	81.9	83.3	81.5	87.4	92.3	5.323* (0.869)
	No	3.1	4.1	4.8	7.7	2.3	0.0	
	Don’t Know	13.3	14.0	11.9	10.8	10.3	7.7	
Diarrhoea	Yes	13.3	15.2	16.7	16.9	16.1	15.4	2.965 (0.982)
	**No**	73.5	70.2	64.3	69.2	72.4	61.5	
	Don’t Know	13.3	14.6	19.0	13.8	11.5	23.1	
Cold and Flu	Yes	13.3	15.8	26.2	16.9	17.2	15.4	5.613* (0.847)
	**No**	78.6	77.2	69.0	76.9	78.2	69.2	
	Don’t Know	8.2	7.0	4.8	6.2	4.6	15.4	
Fever	Yes	25.5	28.1	38.1	44.6	43.7	30.8	15.606 (0.111)
	**No**	64.3	62.6	59.5	50.8	48.3	61.5	
	Don’t Know	10.2	9.4	2.4	4.6	8.0	7.7	
Malaria	Yes	23.5	24.6	26.2	12.3	18.4	30.8	23.222 (**0.010**)
	**No**	25.5	41.5	21.4	30.8	43.7	38.5	
	Don’t Know	51.0	33.9	52.4	56.9	37.9	30.8	
Measles	Yes	15.3	7.0	14.3	9.2	12.6	30.8	18.533 (**0.047**)
	**No**	42.9	57.3	50.0	52.3	63.2	46.2	
	Don’t Know	41.8	35.7	35.7	38.5	24.1	23.1	
Skin or Wound Infection	**Yes**	74.5	75.4	71.4	70.8	81.6	76.9	7.681 (0.660)
	No	11.2	14.6	19.0	16.9	11.5	23.1	
	Don’t Know	14.3	9.9	9.5	12.3	6.9	0.0	
Sore Thorat	Yes	32.7	36.3	54.8	50.8	41.4	30.8	15.673 (0.109)
	**No**	54.1	55.6	42.9	40.0	49.4	69.2	
	Don’t Know	13.3	8.2	2.4	9.2	9.2	0.0	
Body Aches	Yes	8.2	9.9	16.7	12.3	9.2	7.7	5.367 (0.865)
	**No**	74.5	77.8	71.4	78.5	79.3	76.9	
	Don’t Know	17.3	12.3	11.9	9.2	11.5	15.4	
Headaches	Yes	3.1	5.3	7.1	3.1	4.6	7.7	8.383* (0.591)
	**No**	94.9	88.3	85.7	87.7	89.7	76.9	
	Don’t Know	2.0	6.4	7.1	9.2	5.7	15.4	
When do you think you should stop taking antibiotics once you’ve begun treatment?	**After Taking All Antibiotics**	99.0	94.2	88.1	93.8	96.6	100.0	11.813* (0.298)
	When You Feel Better	1.0	5.3	11.9	6.2	3.4	0.0	
	Don’t Know	0.0	0.6	0.0	0.0	0.0	0.0	
t’s okay to use antibiotics that were given to a friend or family member, as long as they were used to treat the same illness	True	5.1	6.4	11.9	4.6	4.6	0.0	7.763* (0.652)
	**False**	91.8	90.6	81.0	90.8	93.1	100.0	
	Don’t Know	3.1	2.9	7.1	4.6	2.3	0.0	
It’s okay to buy the same antibiotics, or request these from a doctor, if you’re sick and they helped you get better when you had the same symptoms before	True	21.4	18.1	33.3	29.2	27.6	30.8	14.586* (0.148)
	**False**	74.5	79.5	66.7	67.7	70.1	53.8	
	Don’t Know	4.1	2.3	0.0	3.1	2.3	15.4	
Antibiotic resistance occurs when your body becomes resistant to antibiotics and they no longer work as well	True	70.4	72.5	81.0	81.5	73.6	84.6	9.072 (0.525)
	**False**	16.3	19.3	7.1	12.3	16.1	15.4	
	Don’t Know	13.3	8.2	11.9	6.2	10.3	0.0	
Many infections are becoming increasingly resistant to treatment by antibiotics	**True**	72.4	76.0	73.8	66.2	81.6	69.2	12.683* (0.242)
	False	2.0	7.0	7.1	6.2	4.6	15.4	
	Don’t Know	25.5	17.0	19.0	27.7	13.8	15.4	
If bacteria are resistant to antibiotics, it can be very difficult or impossible to treat the infections they cause	**True**	83.7	78.9	73.8	72.3	86.2	84.6	17.451* (0.065)
	False	3.1	10.5	11.9	9.2	1.1	7.7	
	Don’t Know	82.7	84.8	73.8	83.1	75.9	76.9	
Antibiotic resistance is an issue that could affect me or my family	**True**	3.1	4.7	9.5	4.6	8.0	23.1	13.724* (0.186)
	False	14.3	10.5	16.7	12.3	16.1	0.0	
	Don’t Know	82.7	84.8	73.8	83.1	75.9	76.9	
Antibiotic resistance is an issue in other countries but not here	True	2.0	1.8	7.1	3.1	6.9	7.7	20.508* (**0.025**)
	**False**	77.6	85.4	73.8	70.8	82.8	92.3	
	Don’t Know	20.4	12.9	19.0	26.2	10.3	0.0	
Antibiotic resistance is only a problem for people who take antibiotics regularly	True	16.3	15.8	42.9	23.1	18.4	30.8	23.341 (**0.010**)
	**False**	58.2	69.0	45.2	58.5	63.2	61.5	
	Don’t Know	25.5	15.2	11.9	18.5	18.4	7.7	
Bacteria which are resistant to antibiotics can be spread from person to person	**True**	62.2	62.6	57.1	55.4	62.1	61.5	6.498 (0.772)
	False	10.2	15.2	14.3	9.2	10.3	7.7	
	Don’t Know	27.6	22.2	28.6	35.4	27.6	30.8	
Antibiotic-resistant infections could make medical procedures like surgery, organ transplants and cancer treatment much more dangerous	**True**	77.6	81.9	71.4	73.8	81.6	100.0	14.502* (0.151)
	False	1.0	4.1	7.1	3.1	3.4	0.0	
	Don’t Know	21.4	14.0	21.4	23.1	14.9	0.0	

*Likelihood Ratio Test instead of Pearson Chi-Square Test Used

#### Age

Table 4 presents significant differences among the age groups in their responses to treatment of diarrhoea (*p* < 0.001), cold and flu (*p* < 0.001), fever (*p* = 0.014), malaria (*p* = 0.049), measles (*p* < 0.001), body aches (*p* < 0.001), and headaches (*p* < 0.001). For each of these conditions, the 18–34 group consistently reports a higher proportion of “Yes” responses compared to the other groups, indicating greater misconception.

There were significant group differences for several items about antibiotic use and resistance: when to stop antibiotics (*p* = 0.003), the acceptability of using antibiotics given to a friend or family member (*p* = 0.022), the acceptability of buying/requesting the same antibiotics that helped previously (*p* = 0.036), the statement that many infections are becoming increasingly resistant to antibiotics (*p* = 0.010), the statement that if bacteria are resistant to antibiotics it can be very difficult or impossible to treat the infections they cause (*p* = 0.005), and the statement that antibiotic-resistant infections could make procedures like surgery, transplants, and cancer treatment much more dangerous (*p* = 0.008). Overall, the 18–34 group shows higher rates of some inappropriate practices and beliefs (e.g., stopping when feeling better, using others’ antibiotics, requesting previously helpful antibiotics), while the 35–54 group tends to show greater agreement with statements reflecting awareness of the growing problem and its clinical consequences (e.g., infections becoming resistant, difficulty treating resistant infections, increased danger for medical procedures) (Table 4).

#### Education level

Significant differences across the groups were found for gonorrhoea (*p* = 0.043) and malaria (*p* = 0.006) (Table 5). For gonorrhoea, the high education group showed a notably higher proportion of respondents who answered “Yes” (43.1%) compared to the low education (31.3%) and medium education (30.3%) groups. Similarly, for malaria, the high education group showed a notably higher proportion of respondents who answered “No” (42.2%) compared to the low education (21.2%) and medium education (34.2%) groups.

Significant differences across the groups emerged for several key items related to antibiotic use and antibiotic resistance. First, groups differed in the belief that it is acceptable to use antibiotics given to a friend or family member (*p* = 0.021), with this misconception more common in the low education group. There were also strong differences in the belief that one can buy or request the same antibiotics that helped during a previous illness (*p* < 0.001), with inappropriate self-directed reuse substantially higher in the low education group.

Knowledge-related items also showed important disparities: the incorrect belief that antibiotic resistance occurs because the body becomes resistant to antibiotics (*p* < 0.001) was far more common in the middle and high education groups. Awareness that many infections are becoming increasingly resistant to antibiotics (*p* = 0.015) and that infections caused by resistant bacteria can become very difficult or impossible to treat (*p* = 0.045) also differed significantly, with recognition increasing progressively from the low to high education groups (Table 5). Additionally, the misconception that antibiotic resistance is a problem only in other countries (*p* < 0.001) and only for people who take antibiotics regularly (*p* = 0.003) is most common in individuals with low education and least common in individuals with high education.

#### Gender

Females were more likely than males to identify treatment of bladder or UTIs (87.0% versus 78.5%, *p* < 0.001) (Table 6). Similarly, females showed better knowledge about diarrhoea (*p* < 0.001), cold and flu (*p* = 0.006), fever (*p* = 0.027), skin or wound infection (*p* = 0.022), and body aches (*p* = 0.008). For headaches (*p* < 0.001), males showed substantially higher “Yes” responses than females (94.4% versus 81.7%), indicating more misconception about females.

Significant gender differences appeared in attitudes and knowledge related to antibiotic use and resistance. Females were more likely than males to correctly state that you should stop taking antibiotics only after completing the full course (97.5% versus 91.6%, *p* = 0.010). There was also a strong difference in the belief that it’s acceptable to use antibiotics given to a friend or family member (*p* < 0.001): males were more likely to endorse this unsafe practice (7.9%) compared with females (4.6%). A further significant difference was found in understanding what antibiotic resistance is (*p* = 0.048). Females were more likely than males to correctly identify that antibiotic resistance does not occur because the body becomes resistant (78.6% versus 68.6%) (Table 6).

#### Household income level

Two conditions showed statistically significant differences across income groups: cold and flu (*p* = 0.023) and malaria (*p* = 0.024) (Table 7). For cold and flu, the lowest-income group had the highest misconception at 27.6%. For malaria, the lowest-income group again showed the highest “Yes” responses at 39.7%, much higher than middle- and higher-income groups (16.5%–20.8%).

Misconception that “antibiotic resistance occurs because the body becomes resistant” differed by income (*p* = 0.050), with the highest-income group (EUR100k+) reporting this misconception most often (83.6%). There was also a significant difference in the belief that antibiotic resistance is a problem in other countries but not in Malta (*p* = 0.014), with the highest-income group being least likely to hold this incorrect belief (0%).

#### Region

Region was significantly associated with misconceptions about whether malaria and measles can be treated with antibiotics (Table 8). For malaria (*p* = 0.010), incorrect “Yes” responses were highest in Gozo and Comino (30.8%), while the South Western region had the lowest rate of incorrect answers (12.3%). For measles (*p* = 0.047), Gozo and Comino again had the highest proportion of incorrect “Yes” responses (30.8%), whereas the Northern Harbour region showed the lowest (7.0%).

Region was significantly associated with two misconceptions about antibiotic resistance. First, the belief that “antibiotic resistance is an issue in other countries but not here” (*p* = 0.025) varied across regions, with the highest incorrect “True” responses in Gozo and Comino (7.7%), followed by Southern Harbour (7.1%) and Western (6.9%), while other regions had very low rates (1.8–3.1%). Second, the misconception that “antibiotic resistance is only a problem for people who take antibiotics regularly” (*p* = 0.010) was most prevalent in Southern Harbour (42.9%) and Gozo and Comino (30.8%), compared with lower rates in other regions (15.8–23.1%) (Table 8).

## Discussion

This study provides the first Malta-specific evidence on public knowledge, attitudes, and behaviours related to antibiotic use and antimicrobial resistance. The findings report a clear knowledge-understanding gap: although most respondents recognised the need to use antibiotics only when prescribed and to complete the full course, misconceptions remained widespread. Notably, 16.6% believed antibiotics treat colds and flu, 33.6% believed they treat fever, and 39.9% believed they treat sore throats. Awareness of the term “antibiotic resistance” was high yet understanding of “antimicrobial resistance” and “AMR” was markedly lower, with only 21% recognising the abbreviation. The most persistent misconception, shared by 75%, was that resistance develops when the body becomes resistant to antibiotics, mirroring findings from the WHO multi-country survey and international literature. Younger adults and individuals with lower education consistently showed lower knowledge and higher uncertainty, while older respondents and those with higher education demonstrated stronger adherence to correct antibiotic-use behaviours. Together, these findings highlight conceptual gaps that must be addressed to strengthen Malta’s AMR response.

Age and education emerged as important determinants of antibiotic-related knowledge and attitudes. Younger adults demonstrated lower understanding of appropriate antibiotic use and AMR, whereas older respondents showed stronger alignment with evidence-based practices. Similarly, individuals with higher educational levels showed better knowledge and fewer misconceptions than those with lower education. Similar findings from other European surveys have underscored the need to prioritise younger and lower-educated groups in national AMR communication and engagement strategies [9, 14, 15].

### Public knowledge, misconceptions, and sociocultural influences

The persistence of key misconceptions in Malta is consistent with European and global patterns. Comparable surveys in Cyprus and Italy [14, 15] identified similar misunderstandings, particularly the belief that antibiotics are effective for viral infections and that resistance develops in the human body rather than in bacteria. The Cyprus survey [15], for example, reported that 70.7% held the same mechanistic misconception, while one-third believed that colds and flu require antibiotics. These parallels reinforce that Malta’s challenges are not isolated but reflect broader regional gaps in AMR understanding. Muflih et al. [30] reported that better understanding of antibiotics is associated with reduced irrational self-medication, while Pennino et al., [31] found that higher education correlates with more accurate antibiotic knowledge.

Evidence demonstrates that misunderstanding of the mechanism of resistance is one of the most stable and widespread misconceptions globally, persisting even in populations with high awareness of “antibiotic resistance.” McCullough et al., showed that this misconception is repeatedly documented across continents, suggesting that traditional public health messaging has not adequately clarified how resistance develops [9]. In our findings, the high proportion of respondents misunderstanding this mechanism (75%) points to an urgent need for more accurate communication, especially since misunderstandings about cause strongly shape risk perception and behavioural intentions [8, 32].

The belief in antibiotics for viral conditions aligns with what is documented in Malta and other Mediterranean countries, where precautionary prescribing and patient expectations may contribute to high antimicrobial use [33]. Previous local work has noted that Maltese cultural norms, including a strong preference for seeking reassurance when unwell and high uncertainty-avoidance, can influence both expectations and prescribing patterns [34, 35]. While our study did not directly measure cultural constructs, the observed misconceptions and expectations are consistent with this broader cultural context, indicating that improved public messaging should also address expectations during common viral illnesses, such as upper respiratory tract infections.

### Patient expectations and prescription dynamics

Expectations surrounding antibiotic prescribing remain a recognised driver of unnecessary use in Europe. Consistent with findings from Saliba-Gustafsson et al., our results suggest that many Maltese citizens continue to view antibiotic prescriptions as a marker of effective medical care [33, 36].

This dynamic contributes to pressure, perceived or real, on clinicians, particularly in primary care. International evidence shows that clinician beliefs about patient expectations strongly influence prescribing, sometimes even more than patients’ actual requests [33]. According to the OECD [37], countries with lower AMR rates, such as those in Northern Europe [38] have more established stewardship practices and stronger norms against unnecessary antibiotic prescribing. This is supported by Machowska, who reported a correlation between patient demand for antibiotics and inappropriate prescribing [36].

The expectation of antibiotics as a “quick fix” is also consistent with earlier Maltese studies and with patterns observed in other Southern European countries. Wilkinson et al. reported that past prescribing reinforces future demand; this aligns with our finding that 23.7% of respondents considered it acceptable to reuse previous antibiotics [39]. Strengthening shared decision-making models may help shift these expectations. Evidence shows that when patients receive clear explanations about why antibiotics are unnecessary, satisfaction remains high even without a prescription. The Maltese primary care context is shifting toward more collaborative communication, and our findings highlight the importance of accelerating this transition [40].

### Comparisons with EU trends and demographic patterns

A 2022 Eurobarometer survey reported that 42% of Maltese respondents had taken antibiotics in the previous year, substantially higher than the EU average of 23% [8]. Although 90% stated they obtained their last antibiotics from a doctor, this figure was slightly lower than previous years and the EU average. In contrast, our study found that 95.5% obtained antibiotics through a doctor or nurse, and only 3.7% reported accessing them without a prescription, a reduction from the 10% reported in Eurobarometer 2022. This discrepancy likely reflects respondents interpreting telephone-based prescribing during the COVID-19 period as “non-prescription” access.

Recent antibiotic use in Malta (9% in the past month) was markedly lower than WHO multi-country estimates (35%), reflecting broader European post-pandemic reductions in prescribing. However, Malta also reported fewer individuals who had never taken antibiotic (0.6% vs. 4% in WHO countries) indicating high lifetime exposure [8].

Demographic patterns closely mirror trends observed in other European studies. Younger adults (18–34) showed the highest rates of misconceptions and inappropriate attitudes, consistent with findings by Vella [41]. While Borg et al. [42] highlights a significant difference in self-administration behaviours depending on individuals’ levels of education. A higher proportion of those who have completed tertiary education demonstrated a tendency for self-administering antibiotics compared to those who lack any formal educational qualifications. Respondents with lower education also showed greater uncertainty and misconceptions, a pattern widely documented in Italian, Cypriot, and multinational surveys [14, 15]. These findings underscore the need for targeted educational strategies tailored to younger adults and those with lower educational attainment, focusing on correcting common mechanistic and indication-related misconceptions. Gender differences were small but notable: female respondents demonstrated better knowledge on several conditions treatable with antibiotics yet showed higher misunderstanding regarding the cause of AMR. These patterns highlight the complexity of interpreting gender effects and suggest that gender-responsive communication strategies may be warranted.

### Strengths and limitations

A key strength of this study is that it offers the first detailed national profile of public knowledge, attitudes, and behaviours related to antibiotic use and AMR in Malta, providing data beyond what Eurobarometer surveys can capture. The use of a well-established WHO survey instrument facilitates international comparison, and the active monitoring of demographic distributions during recruitment helped to mitigate, though not eliminate, compositional imbalances across age, gender, and education strata; it does not, however, address self-selection into the study.

Limitations include the reliance on non-probability convenience sampling, which may substantially reduce generalisability. As the survey was disseminated via social media platforms, it is likely to have disproportionately attracted individuals with a pre-existing interest in health topics, who may be more knowledgeable or concerned about antibiotic use and AMR than the broader Maltese population. This volunteer effect represents a limitation on the findings. Accordingly, results should not be interpreted as population-representative estimates. The final sample of 476 respondents also falls below the WHO recommendation of approximately 1,000 for online surveys of this type, which may further limit the precision of estimates. The over-representation of females is consistent with typical health survey participation patterns [43, 44], and potential response and social desirability biases cannot be excluded. Although face-to-face and online distribution methods helped broaden reach, online dissemination may have favoured younger participants. The use of non-parametric statistical tests, combined with multiple-comparison corrections, may have reduced power to detect small differences.

### Conclusion

This study highlights persistent misconceptions about antibiotic use and AMR within the Maltese community, despite high awareness of basic principles of responsible antibiotic use. Misunderstandings of key biological mechanisms, uncertainty about appropriate indications, and variable perceptions of personal risk remain common, particularly among younger adults and those with lower educational attainment. Addressing these gaps will require targeted, mechanism-focused public communication and strengthened clinician-patient dialogue to align expectations with evidence-based care. Improving public understanding of AMR is essential to reducing unnecessary antibiotic use and supporting Malta’s broader antimicrobial stewardship efforts.

## Supplementary Information

Supplementary Material 1.

## Data Availability

The datasets used and/or analysed during the current study are available from the corresponding author on reasonable request.

## Abbreviations

AMR: Antimicrobial Resistance
AMS: Antimicrobial Stewardship
CHERRIES: Checklist for Reporting Results of Internet E-Surveys
DPOs: Data Protection Officers
ECDC: European Centre for Disease Prevention and Control
EARSS: European Antimicrobial Resistance Surveillance System
EU: European Union
EU/EEA: European Union/European Economic Area
IPC: Infection Prevention and Control
NAC: National Antibiotic Committee
NSO: National Statistics Office
OECD: Organisation for Economic Co-operation and Development
SPSS: Statistical Package for the Social Sciences
UTI: Urinary Tract Infection
WHO: World Health Organization
