# Supplementary material for: Public awareness and knowledge of antibiotic use and antimicrobial resistance in Malta
Source: BMC Public Health. 2026 Sep 28;26:2893. doi: 10.1186/s12889-026-29529-w (PMC13647623; doi:10.1186/s12889-026-29529-w)
Supplement: Supplementary file 1 [file 12889_2026_29529_MOESM1_ESM.zip › BMC_AMR_Supplementary Material 1.docx]

Supplementary Material 1: Online Questionnaire
