# Supplementary material for: Public awareness and knowledge of antibiotic use and antimicrobial resistance in Malta
Source: BMC Public Health. 2026 Sep 28;26:2893. doi: 10.1186/s12889-026-29529-w (PMC13647623; doi:10.1186/s12889-026-29529-w)
Supplement: Supplementary file 1 [file 12889_2026_29529_MOESM1_ESM.zip › BMC_AMR_Supplementary Material 2.docx]

Supplementary Material 2: CHERRIES Checklist

| **Item Category** | **Checklist Item** | **Explanation** | **Answer** |
| --- | --- | --- | --- |
| **Design** | Describe survey design | Describe target population, sample frame. Is the sample a convenience sample? (In “open” surveys this is most likely.) | The survey was designed using Google Forms and targeted the Maltese population to assess their knowledge and behaviours related to AMR. It was an open survey accessible to Maltese citizens aged 18 and over. |
| **IRB (Institutional Review Board) approval and informed consent process** | IRB approval | Mention whether the paper has been approved by an IRB. | The paper’s proposal and associated documents were initially reviewed and approved by the DPOs of Mater Dei Hospital and Primary Health Care (refer to Supplementary Material A.3.2, A.4). Institutional permissions were granted by Mater Dei Hospital management, including the Chief Executive Officer (refer to Supplementary Material A.3.1). Approval was granted from the research ethics committee (URECA) within the Faculty of ICT at the University of Malta, following the completion of the Mater Dei Hospital Data Protection Clearance Declaration Form (Supplementary Material A.3.3) and an ethical self-assessment form (Supplementary Material A.1). Approvals were then obtained from Mater Dei Hospital department heads in Pathology, Infection Control, Infectious Diseases, and Pharmacy, facilitating access to healthcare professionals (refer to Supplementary Material A.3.4). |
|  | Informed consent | Describe the informed consent process. Where were the participants told the length of time of the survey, which data were stored and where and for how long, who the investigator was, and the purpose of the paper? | The informed consent process is detailed at the beginning of the survey. Participants were informed about the survey’s purpose, confidentiality, estimated time (5-10 minutes), and the investigator’s contact information. No personal data such as names or IP addresses were collected. |
|  | Data protection | If any personal information was collected or stored, describe what mechanisms were used to protect unauthorized access. | No personal information was collected. Data was collected anonymously, and confidentiality was ensured by not storing names or IP addresses. |
| **Development and pre-testing** | Development and testing | State how the survey was developed, including whether the usability and technical functionality of the electronic questionnaire had been tested before fielding the questionnaire. | The survey was adapted from the WHO Antibiotic Resistance Multi-Country Public Awareness Survey and was tested during a pilot phase with 10 respondents to assess clarity, length, functionality, and usability. |
| **Recruitment process and description of the sample having access to the questionnaire** | Open survey versus closed survey | An “open survey” is a survey open for each visitor of a site, while a closed survey is only open to a sample which the investigator knows (password-protected survey). | This was an open survey accessible to all Maltese citizens aged 18 and over. |
|  | Contact mode | Indicate whether or not the initial contact with the potential participants was made on the Internet. (Investigators may also send out questionnaires by mail and allow for Web-based data entry.) | Initial contact was made via the Internet, with the survey shared on LinkedIn, Facebook groups, and the Infection Prevention and Control Department’s Facebook page. |
|  | Advertising the survey | How/where was the survey announced or advertised? Some examples are offline media (newspapers), or online (mailing lists – If yes, which ones?) or banner ads (Where were these banner ads posted and what did they look like?). It is important to know the wording of the announcement as it will heavily influence who chooses to participate. Ideally the survey announcement should be published as an Supplementary Material. | The survey was advertised online through social media platforms like LinkedIn and Facebook, including specific groups related to health and infection prevention. |
| **Survey administration** | Web/E-mail | State the type of e-survey (e.g., one posted on a Web site, or one sent out through e-mail). If it is an e-mail survey, were the responses entered manually into a database, or was there an automatic method for capturing responses? | The survey was a web-based survey hosted on Google Forms. Responses were automatically captured and stored within Google Forms. |
|  | Context | Describe the Web site (for mailing list/newsgroup) in which the survey was posted. What is the Web site about, who is visiting it, what are visitors normally looking for? Discuss to what degree the content of the Web site could pre-select the sample or influence the results. For example, a survey about vaccination on an anti-immunization Web site will have different results from a Web survey conducted on a government Web site. | The survey was posted on social media platforms, which are visited by a diverse population. Specific health-related groups may have attracted individuals more interested in health topics. |
|  | Mandatory/voluntary | Was it a mandatory survey to be filled in by every visitor who wanted to enter the Web site, or was it a voluntary survey? | It was a voluntary survey. |
|  | Incentives | Were any incentives offered (e.g., monetary, prizes, or non-monetary incentives such as an offer to provide the survey results)? | No incentives were offered for participating in the survey. |
|  | Time/Date | In what timeframe were the data collected? | The data were collected over three weeks from April 9, 2024, to April 29, 2024. |
|  | Randomization of items or questionnaires | To prevent biases items can be randomized or alternated. | The methodology does not mention the use of randomization of items or questionnaires. |
|  | Adaptive questioning | Use adaptive questioning (certain items, or only conditionally displayed based on responses to other items) to reduce number and complexity of the questions. | The methodology does not mention the use of adaptive questioning. |
|  | Number of Items | What was the number of questionnaire items per page? The number of items is an important factor for the completion rate. | The survey items were distributed across multiple sections, with an average of 2-5 items per page. |
|  | Number of screens (pages) | Over how many pages was the questionnaire distributed? The number of items is an important factor for the completion rate. | The survey was distributed over 7 pages, each covering different sections such as demographics, use of antibiotics, knowledge about antibiotics, and knowledge about antimicrobial resistance. |
|  | Completeness check | It is technically possible to do consistency or completeness checks before the questionnaire is submitted. Was this done, and if “yes”, how (usually JavaScript)? An alternative is to check for completeness after the questionnaire has been submitted (and highlight mandatory items). If this has been done, it should be reported. All items should provide a non-response option such as “not applicable” or “rather not say”, and selection of one response option should be enforced. | The survey required Google login to ensure single responses and used mandatory fields to ensure completeness. |
|  | Review step | State whether respondents were able to review and change their answers (e.g., through a Back button or a Review step which displays a summary of the responses and asks the respondents if they are correct). | The survey did not explicitly mention a review step allowing respondents to change their answers. |
| **Response rates** | Unique site visitor | If you provide view rates or participation rates, you need to define how you determined a unique visitor. There are different techniques available, based on IP addresses or cookies or both. | Google login was required to ensure unique responses, but no IP address tracking was mentioned. |
|  | View rate (Ratio of unique survey visitors/unique site visitors) | Requires counting unique visitors to the first page of the survey, divided by the number of unique site visitors (not page views!). It is not unusual to have view rates of less than 0.1 % if the survey is voluntary. | The methodology does not provide details on the view rate calculation. |
|  | Participation rate (Ratio of unique visitors who agreed to participate/unique first survey page visitors) | Count the unique number of people who filled in the first survey page (or agreed to participate, for example by checking a checkbox), divided by visitors who visit the first page of the survey (or the informed consents page, if present). This can also be called “recruitment” rate. | The methodology does not provide specific details on the participation rate. |
|  | Completion rate (Ratio of users who finished the survey/users who agreed to participate) | The number of people submitting the last questionnaire page, divided by the number of people who agreed to participate (or submitted the first survey page). This is only relevant if there is a separate “informed consent” page or if the survey goes over several pages. This is a measure for attrition. Note that “completion” can involve leaving questionnaire items blank. This is not a measure for how completely questionnaires were filled in. (If you need a measure for this, use the word “completeness rate”.) | The methodology does not provide specific details on the completion rate. |
| **Preventing multiple entries from the same individual** | Cookies used | Indicate whether cookies were used to assign a unique user identifier to each client computer. If so, mention the page on which the cookie was set and read, and how long the cookie was valid. Were duplicate entries avoided by preventing users access to the survey twice; or were duplicate database entries having the same user ID eliminated before analysis? In the latter case, which entries were kept for analysis (e.g., the first entry or the most recent)? | Google login was used to ensure single responses from each participant. No mention of cookies was made. |
|  | IP check | Indicate whether the IP address of the client computer was used to identify potential duplicate entries from the same user. If so, mention the period of time for which no two entries from the same IP address were allowed (e.g., 24 hours). Were duplicate entries avoided by preventing users with the same IP address access to the survey twice; or were duplicate database entries having the same IP address within a given period of time eliminated before analysis? If the latter, which entries were kept for analysis (e.g., the first entry or the most recent)? | The methodology does not include IP checks. |
|  | Log file analysis | Indicate whether other techniques to analyse the log file for identification of multiple entries were used. If so, please describe. | The methodology does not include log file. |
