# Supplementary material for: Public awareness and knowledge of antibiotic use and antimicrobial resistance in Malta
Source: BMC Public Health. 2026 Sep 28;26:2893. doi: 10.1186/s12889-026-29529-w (PMC13647623; doi:10.1186/s12889-026-29529-w)
Supplement: Supplementary file 1 [file 12889_2026_29529_MOESM1_ESM.zip › BMC_AMR_Supplementary Material 3_SPSS Outputs.docx]

Supplementary Material 3: Knowledge Assessment Results

DESCRIPTIVES AND NORMALITY TESTS

(1) AGE AND LIKERT SCALE

| **Descriptives** | | | | | |
| --- | --- | --- | --- | --- | --- |
|  | AgeGroup | | | Statistic | Std. Error |
| On the scale shown, how much do you agree the following actions would help address the problem of antibiotic resistance? [People should use antibiotics only when they are prescribed by a doctor or nurse] | 18-34 | Mean | | 4.79 | .034 |
|  |  | 95% Confidence Interval for Mean | Lower Bound | 4.72 |  |
|  |  |  | Upper Bound | 4.85 |  |
|  |  | 5% Trimmed Mean | | 4.86 |  |
|  |  | Median | | 5.00 |  |
|  |  | Variance | | .258 |  |
|  |  | Std. Deviation | | .508 |  |
|  |  | Minimum | | 2 |  |
|  |  | Maximum | | 5 |  |
|  |  | Range | | 3 |  |
|  |  | Interquartile Range | | 0 |  |
|  |  | Skewness | | -2.575 | .162 |
|  |  | Kurtosis | | 6.883 | .323 |
|  | 35-54 | Mean | | 4.91 | .028 |
|  |  | 95% Confidence Interval for Mean | Lower Bound | 4.86 |  |
|  |  |  | Upper Bound | 4.97 |  |
|  |  | 5% Trimmed Mean | | 4.99 |  |
|  |  | Median | | 5.00 |  |
|  |  | Variance | | .143 |  |
|  |  | Std. Deviation | | .378 |  |
|  |  | Minimum | | 2 |  |
|  |  | Maximum | | 5 |  |
|  |  | Range | | 3 |  |
|  |  | Interquartile Range | | 0 |  |
|  |  | Skewness | | -5.121 | .178 |
|  |  | Kurtosis | | 28.736 | .354 |
|  | 55+ | Mean | | 4.92 | .064 |
|  |  | 95% Confidence Interval for Mean | Lower Bound | 4.79 |  |
|  |  |  | Upper Bound | 5.05 |  |
|  |  | 5% Trimmed Mean | | 5.00 |  |
|  |  | Median | | 5.00 |  |
|  |  | Variance | | .264 |  |
|  |  | Std. Deviation | | .513 |  |
|  |  | Minimum | | 1 |  |
|  |  | Maximum | | 5 |  |
|  |  | Range | | 4 |  |
|  |  | Interquartile Range | | 0 |  |
|  |  | Skewness | | -7.393 | .299 |
|  |  | Kurtosis | | 56.460 | .590 |
| On the scale shown, how much do you agree the following actions would help address the problem of antibiotic resistance? [Farmers should give fewer antibiotics to food-producing animals] | 18-34 | Mean | | 4.19 | .063 |
|  |  | 95% Confidence Interval for Mean | Lower Bound | 4.07 |  |
|  |  |  | Upper Bound | 4.32 |  |
|  |  | 5% Trimmed Mean | | 4.26 |  |
|  |  | Median | | 5.00 |  |
|  |  | Variance | | .896 |  |
|  |  | Std. Deviation | | .947 |  |
|  |  | Minimum | | 1 |  |
|  |  | Maximum | | 5 |  |
|  |  | Range | | 4 |  |
|  |  | Interquartile Range | | 2 |  |
|  |  | Skewness | | -.901 | .162 |
|  |  | Kurtosis | | .146 | .323 |
|  | 35-54 | Mean | | 4.56 | .063 |
|  |  | 95% Confidence Interval for Mean | Lower Bound | 4.44 |  |
|  |  |  | Upper Bound | 4.69 |  |
|  |  | 5% Trimmed Mean | | 4.68 |  |
|  |  | Median | | 5.00 |  |
|  |  | Variance | | .753 |  |
|  |  | Std. Deviation | | .868 |  |
|  |  | Minimum | | 1 |  |
|  |  | Maximum | | 5 |  |
|  |  | Range | | 4 |  |
|  |  | Interquartile Range | | 1 |  |
|  |  | Skewness | | -2.113 | .178 |
|  |  | Kurtosis | | 4.180 | .354 |
|  | 55+ | Mean | | 4.80 | .074 |
|  |  | 95% Confidence Interval for Mean | Lower Bound | 4.65 |  |
|  |  |  | Upper Bound | 4.95 |  |
|  |  | 5% Trimmed Mean | | 4.88 |  |
|  |  | Median | | 5.00 |  |
|  |  | Variance | | .355 |  |
|  |  | Std. Deviation | | .596 |  |
|  |  | Minimum | | 1 |  |
|  |  | Maximum | | 5 |  |
|  |  | Range | | 4 |  |
|  |  | Interquartile Range | | 0 |  |
|  |  | Skewness | | -4.560 | .299 |
|  |  | Kurtosis | | 26.174 | .590 |
| On the scale shown, how much do you agree the following actions would help address the problem of antibiotic resistance? [People should not keep antibiotics and use them later for other illnesses] | 18-34 | Mean | | 4.32 | .066 |
|  |  | 95% Confidence Interval for Mean | Lower Bound | 4.19 |  |
|  |  |  | Upper Bound | 4.44 |  |
|  |  | 5% Trimmed Mean | | 4.42 |  |
|  |  | Median | | 5.00 |  |
|  |  | Variance | | .967 |  |
|  |  | Std. Deviation | | .983 |  |
|  |  | Minimum | | 1 |  |
|  |  | Maximum | | 5 |  |
|  |  | Range | | 4 |  |
|  |  | Interquartile Range | | 1 |  |
|  |  | Skewness | | -1.379 | .162 |
|  |  | Kurtosis | | 1.112 | .323 |
|  | 35-54 | Mean | | 4.57 | .063 |
|  |  | 95% Confidence Interval for Mean | Lower Bound | 4.45 |  |
|  |  |  | Upper Bound | 4.70 |  |
|  |  | 5% Trimmed Mean | | 4.70 |  |
|  |  | Median | | 5.00 |  |
|  |  | Variance | | .741 |  |
|  |  | Std. Deviation | | .861 |  |
|  |  | Minimum | | 1 |  |
|  |  | Maximum | | 5 |  |
|  |  | Range | | 4 |  |
|  |  | Interquartile Range | | 1 |  |
|  |  | Skewness | | -2.170 | .178 |
|  |  | Kurtosis | | 4.226 | .354 |
|  | 55+ | Mean | | 4.64 | .108 |
|  |  | 95% Confidence Interval for Mean | Lower Bound | 4.43 |  |
|  |  |  | Upper Bound | 4.86 |  |
|  |  | 5% Trimmed Mean | | 4.78 |  |
|  |  | Median | | 5.00 |  |
|  |  | Variance | | .742 |  |
|  |  | Std. Deviation | | .861 |  |
|  |  | Minimum | | 1 |  |
|  |  | Maximum | | 5 |  |
|  |  | Range | | 4 |  |
|  |  | Interquartile Range | | 0 |  |
|  |  | Skewness | | -2.914 | .299 |
|  |  | Kurtosis | | 8.983 | .590 |
| On the scale shown, how much do you agree the following actions would help address the problem of antibiotic resistance? [Parents should make sure all of their children’s vaccinations are up-to-date] | 18-34 | Mean | | 4.76 | .040 |
|  |  | 95% Confidence Interval for Mean | Lower Bound | 4.68 |  |
|  |  |  | Upper Bound | 4.83 |  |
|  |  | 5% Trimmed Mean | | 4.85 |  |
|  |  | Median | | 5.00 |  |
|  |  | Variance | | .355 |  |
|  |  | Std. Deviation | | .596 |  |
|  |  | Minimum | | 2 |  |
|  |  | Maximum | | 5 |  |
|  |  | Range | | 3 |  |
|  |  | Interquartile Range | | 0 |  |
|  |  | Skewness | | -2.668 | .162 |
|  |  | Kurtosis | | 7.032 | .323 |
|  | 35-54 | Mean | | 4.79 | .046 |
|  |  | 95% Confidence Interval for Mean | Lower Bound | 4.70 |  |
|  |  |  | Upper Bound | 4.88 |  |
|  |  | 5% Trimmed Mean | | 4.91 |  |
|  |  | Median | | 5.00 |  |
|  |  | Variance | | .392 |  |
|  |  | Std. Deviation | | .626 |  |
|  |  | Minimum | | 1 |  |
|  |  | Maximum | | 5 |  |
|  |  | Range | | 4 |  |
|  |  | Interquartile Range | | 0 |  |
|  |  | Skewness | | -3.540 | .178 |
|  |  | Kurtosis | | 13.463 | .354 |
|  | 55+ | Mean | | 4.98 | .016 |
|  |  | 95% Confidence Interval for Mean | Lower Bound | 4.95 |  |
|  |  |  | Upper Bound | 5.02 |  |
|  |  | 5% Trimmed Mean | | 5.00 |  |
|  |  | Median | | 5.00 |  |
|  |  | Variance | | .016 |  |
|  |  | Std. Deviation | | .125 |  |
|  |  | Minimum | | 4 |  |
|  |  | Maximum | | 5 |  |
|  |  | Range | | 1 |  |
|  |  | Interquartile Range | | 0 |  |
|  |  | Skewness | | -8.000 | .299 |
|  |  | Kurtosis | | 64.000 | .590 |
| On the scale shown, how much do you agree the following actions would help address the problem of antibiotic resistance? [People should wash their hands regularly] | 18-34 | Mean | | 4.76 | .038 |
|  |  | 95% Confidence Interval for Mean | Lower Bound | 4.69 |  |
|  |  |  | Upper Bound | 4.84 |  |
|  |  | 5% Trimmed Mean | | 4.86 |  |
|  |  | Median | | 5.00 |  |
|  |  | Variance | | .324 |  |
|  |  | Std. Deviation | | .569 |  |
|  |  | Minimum | | 1 |  |
|  |  | Maximum | | 5 |  |
|  |  | Range | | 4 |  |
|  |  | Interquartile Range | | 0 |  |
|  |  | Skewness | | -2.906 | .162 |
|  |  | Kurtosis | | 10.445 | .323 |
|  | 35-54 | Mean | | 4.81 | .049 |
|  |  | 95% Confidence Interval for Mean | Lower Bound | 4.72 |  |
|  |  |  | Upper Bound | 4.91 |  |
|  |  | 5% Trimmed Mean | | 4.94 |  |
|  |  | Median | | 5.00 |  |
|  |  | Variance | | .443 |  |
|  |  | Std. Deviation | | .666 |  |
|  |  | Minimum | | 1 |  |
|  |  | Maximum | | 5 |  |
|  |  | Range | | 4 |  |
|  |  | Interquartile Range | | 0 |  |
|  |  | Skewness | | -3.968 | .178 |
|  |  | Kurtosis | | 16.114 | .354 |
|  | 55+ | Mean | | 4.92 | .040 |
|  |  | 95% Confidence Interval for Mean | Lower Bound | 4.84 |  |
|  |  |  | Upper Bound | 5.00 |  |
|  |  | 5% Trimmed Mean | | 4.99 |  |
|  |  | Median | | 5.00 |  |
|  |  | Variance | | .105 |  |
|  |  | Std. Deviation | | .324 |  |
|  |  | Minimum | | 3 |  |
|  |  | Maximum | | 5 |  |
|  |  | Range | | 2 |  |
|  |  | Interquartile Range | | 0 |  |
|  |  | Skewness | | -4.542 | .299 |
|  |  | Kurtosis | | 21.934 | .590 |
| On the scale shown, how much do you agree the following actions would help address the problem of antibiotic resistance? [Doctors should only prescribe antibiotics when they are needed ] | 18-34 | Mean | | 4.85 | .032 |
|  |  | 95% Confidence Interval for Mean | Lower Bound | 4.79 |  |
|  |  |  | Upper Bound | 4.92 |  |
|  |  | 5% Trimmed Mean | | 4.93 |  |
|  |  | Median | | 5.00 |  |
|  |  | Variance | | .224 |  |
|  |  | Std. Deviation | | .473 |  |
|  |  | Minimum | | 1 |  |
|  |  | Maximum | | 5 |  |
|  |  | Range | | 4 |  |
|  |  | Interquartile Range | | 0 |  |
|  |  | Skewness | | -4.260 | .162 |
|  |  | Kurtosis | | 23.445 | .323 |
|  | 35-54 | Mean | | 4.96 | .021 |
|  |  | 95% Confidence Interval for Mean | Lower Bound | 4.92 |  |
|  |  |  | Upper Bound | 5.00 |  |
|  |  | 5% Trimmed Mean | | 5.00 |  |
|  |  | Median | | 5.00 |  |
|  |  | Variance | | .084 |  |
|  |  | Std. Deviation | | .290 |  |
|  |  | Minimum | | 2 |  |
|  |  | Maximum | | 5 |  |
|  |  | Range | | 3 |  |
|  |  | Interquartile Range | | 0 |  |
|  |  | Skewness | | -8.005 | .178 |
|  |  | Kurtosis | | 70.347 | .354 |
|  | 55+ | Mean | | 4.95 | .027 |
|  |  | 95% Confidence Interval for Mean | Lower Bound | 4.90 |  |
|  |  |  | Upper Bound | 5.01 |  |
|  |  | 5% Trimmed Mean | | 5.00 |  |
|  |  | Median | | 5.00 |  |
|  |  | Variance | | .045 |  |
|  |  | Std. Deviation | | .213 |  |
|  |  | Minimum | | 4 |  |
|  |  | Maximum | | 5 |  |
|  |  | Range | | 1 |  |
|  |  | Interquartile Range | | 0 |  |
|  |  | Skewness | | -4.391 | .299 |
|  |  | Kurtosis | | 17.838 | .590 |
| On the scale shown, how much do you agree the following actions would help address the problem of antibiotic resistance? [Governments should reward the development of new antibiotics] | 18-34 | Mean | | 3.97 | .067 |
|  |  | 95% Confidence Interval for Mean | Lower Bound | 3.84 |  |
|  |  |  | Upper Bound | 4.11 |  |
|  |  | 5% Trimmed Mean | | 4.05 |  |
|  |  | Median | | 4.00 |  |
|  |  | Variance | | 1.017 |  |
|  |  | Std. Deviation | | 1.009 |  |
|  |  | Minimum | | 1 |  |
|  |  | Maximum | | 5 |  |
|  |  | Range | | 4 |  |
|  |  | Interquartile Range | | 2 |  |
|  |  | Skewness | | -.631 | .162 |
|  |  | Kurtosis | | -.294 | .323 |
|  | 35-54 | Mean | | 3.96 | .087 |
|  |  | 95% Confidence Interval for Mean | Lower Bound | 3.78 |  |
|  |  |  | Upper Bound | 4.13 |  |
|  |  | 5% Trimmed Mean | | 4.06 |  |
|  |  | Median | | 4.00 |  |
|  |  | Variance | | 1.428 |  |
|  |  | Std. Deviation | | 1.195 |  |
|  |  | Minimum | | 1 |  |
|  |  | Maximum | | 5 |  |
|  |  | Range | | 4 |  |
|  |  | Interquartile Range | | 2 |  |
|  |  | Skewness | | -.948 | .178 |
|  |  | Kurtosis | | .076 | .354 |
|  | 55+ | Mean | | 4.30 | .119 |
|  |  | 95% Confidence Interval for Mean | Lower Bound | 4.06 |  |
|  |  |  | Upper Bound | 4.54 |  |
|  |  | 5% Trimmed Mean | | 4.42 |  |
|  |  | Median | | 5.00 |  |
|  |  | Variance | | .910 |  |
|  |  | Std. Deviation | | .954 |  |
|  |  | Minimum | | 1 |  |
|  |  | Maximum | | 5 |  |
|  |  | Range | | 4 |  |
|  |  | Interquartile Range | | 1 |  |
|  |  | Skewness | | -1.655 | .299 |
|  |  | Kurtosis | | 3.010 | .590 |
| On the scale shown, how much do you agree the following actions would help address the problem of antibiotic resistance? [Pharmaceutical companies should develop new antibiotics] | 18-34 | Mean | | 3.89 | .064 |
|  |  | 95% Confidence Interval for Mean | Lower Bound | 3.77 |  |
|  |  |  | Upper Bound | 4.02 |  |
|  |  | 5% Trimmed Mean | | 3.95 |  |
|  |  | Median | | 4.00 |  |
|  |  | Variance | | .908 |  |
|  |  | Std. Deviation | | .953 |  |
|  |  | Minimum | | 1 |  |
|  |  | Maximum | | 5 |  |
|  |  | Range | | 4 |  |
|  |  | Interquartile Range | | 2 |  |
|  |  | Skewness | | -.503 | .162 |
|  |  | Kurtosis | | -.267 | .323 |
|  | 35-54 | Mean | | 4.07 | .079 |
|  |  | 95% Confidence Interval for Mean | Lower Bound | 3.91 |  |
|  |  |  | Upper Bound | 4.23 |  |
|  |  | 5% Trimmed Mean | | 4.18 |  |
|  |  | Median | | 4.00 |  |
|  |  | Variance | | 1.173 |  |
|  |  | Std. Deviation | | 1.083 |  |
|  |  | Minimum | | 1 |  |
|  |  | Maximum | | 5 |  |
|  |  | Range | | 4 |  |
|  |  | Interquartile Range | | 2 |  |
|  |  | Skewness | | -1.089 | .178 |
|  |  | Kurtosis | | .664 | .354 |
|  | 55+ | Mean | | 4.38 | .121 |
|  |  | 95% Confidence Interval for Mean | Lower Bound | 4.13 |  |
|  |  |  | Upper Bound | 4.62 |  |
|  |  | 5% Trimmed Mean | | 4.49 |  |
|  |  | Median | | 5.00 |  |
|  |  | Variance | | .937 |  |
|  |  | Std. Deviation | | .968 |  |
|  |  | Minimum | | 1 |  |
|  |  | Maximum | | 5 |  |
|  |  | Range | | 4 |  |
|  |  | Interquartile Range | | 1 |  |
|  |  | Skewness | | -1.478 | .299 |
|  |  | Kurtosis | | 1.541 | .590 |
| On the scale shown, how much do you agree with the following statements? [Antibiotic resistance is one of the biggest problems the world faces] | 18-34 | Mean | | 3.44 | .075 |
|  |  | 95% Confidence Interval for Mean | Lower Bound | 3.29 |  |
|  |  |  | Upper Bound | 3.58 |  |
|  |  | 5% Trimmed Mean | | 3.48 |  |
|  |  | Median | | 3.00 |  |
|  |  | Variance | | 1.274 |  |
|  |  | Std. Deviation | | 1.129 |  |
|  |  | Minimum | | 1 |  |
|  |  | Maximum | | 5 |  |
|  |  | Range | | 4 |  |
|  |  | Interquartile Range | | 1 |  |
|  |  | Skewness | | -.167 | .162 |
|  |  | Kurtosis | | -.756 | .323 |
|  | 35-54 | Mean | | 3.79 | .078 |
|  |  | 95% Confidence Interval for Mean | Lower Bound | 3.64 |  |
|  |  |  | Upper Bound | 3.95 |  |
|  |  | 5% Trimmed Mean | | 3.86 |  |
|  |  | Median | | 4.00 |  |
|  |  | Variance | | 1.144 |  |
|  |  | Std. Deviation | | 1.070 |  |
|  |  | Minimum | | 1 |  |
|  |  | Maximum | | 5 |  |
|  |  | Range | | 4 |  |
|  |  | Interquartile Range | | 2 |  |
|  |  | Skewness | | -.480 | .178 |
|  |  | Kurtosis | | -.391 | .354 |
|  | 55+ | Mean | | 3.91 | .131 |
|  |  | 95% Confidence Interval for Mean | Lower Bound | 3.64 |  |
|  |  |  | Upper Bound | 4.17 |  |
|  |  | 5% Trimmed Mean | | 3.99 |  |
|  |  | Median | | 4.00 |  |
|  |  | Variance | | 1.102 |  |
|  |  | Std. Deviation | | 1.050 |  |
|  |  | Minimum | | 1 |  |
|  |  | Maximum | | 5 |  |
|  |  | Range | | 4 |  |
|  |  | Interquartile Range | | 2 |  |
|  |  | Skewness | | -.827 | .299 |
|  |  | Kurtosis | | .250 | .590 |
| On the scale shown, how much do you agree with the following statements? [Medical experts will solve the problem of antibiotic resistance before it becomes too serious] | 18-34 | Mean | | 2.88 | .062 |
|  |  | 95% Confidence Interval for Mean | Lower Bound | 2.76 |  |
|  |  |  | Upper Bound | 3.00 |  |
|  |  | 5% Trimmed Mean | | 2.87 |  |
|  |  | Median | | 3.00 |  |
|  |  | Variance | | .874 |  |
|  |  | Std. Deviation | | .935 |  |
|  |  | Minimum | | 1 |  |
|  |  | Maximum | | 5 |  |
|  |  | Range | | 4 |  |
|  |  | Interquartile Range | | 1 |  |
|  |  | Skewness | | .076 | .162 |
|  |  | Kurtosis | | .038 | .323 |
|  | 35-54 | Mean | | 2.87 | .081 |
|  |  | 95% Confidence Interval for Mean | Lower Bound | 2.71 |  |
|  |  |  | Upper Bound | 3.03 |  |
|  |  | 5% Trimmed Mean | | 2.85 |  |
|  |  | Median | | 3.00 |  |
|  |  | Variance | | 1.235 |  |
|  |  | Std. Deviation | | 1.111 |  |
|  |  | Minimum | | 1 |  |
|  |  | Maximum | | 5 |  |
|  |  | Range | | 4 |  |
|  |  | Interquartile Range | | 1 |  |
|  |  | Skewness | | .030 | .178 |
|  |  | Kurtosis | | -.353 | .354 |
|  | 55+ | Mean | | 3.38 | .150 |
|  |  | 95% Confidence Interval for Mean | Lower Bound | 3.07 |  |
|  |  |  | Upper Bound | 3.68 |  |
|  |  | 5% Trimmed Mean | | 3.42 |  |
|  |  | Median | | 3.00 |  |
|  |  | Variance | | 1.444 |  |
|  |  | Std. Deviation | | 1.202 |  |
|  |  | Minimum | | 1 |  |
|  |  | Maximum | | 5 |  |
|  |  | Range | | 4 |  |
|  |  | Interquartile Range | | 2 |  |
|  |  | Skewness | | -.092 | .299 |
|  |  | Kurtosis | | -.834 | .590 |
| On the scale shown, how much do you agree with the following statements? [Everyone needs to take responsibility for using antibiotics responsibly] | 18-34 | Mean | | 4.75 | .037 |
|  |  | 95% Confidence Interval for Mean | Lower Bound | 4.68 |  |
|  |  |  | Upper Bound | 4.82 |  |
|  |  | 5% Trimmed Mean | | 4.84 |  |
|  |  | Median | | 5.00 |  |
|  |  | Variance | | .313 |  |
|  |  | Std. Deviation | | .559 |  |
|  |  | Minimum | | 2 |  |
|  |  | Maximum | | 5 |  |
|  |  | Range | | 3 |  |
|  |  | Interquartile Range | | 0 |  |
|  |  | Skewness | | -2.478 | .162 |
|  |  | Kurtosis | | 6.395 | .323 |
|  | 35-54 | Mean | | 4.85 | .031 |
|  |  | 95% Confidence Interval for Mean | Lower Bound | 4.79 |  |
|  |  |  | Upper Bound | 4.91 |  |
|  |  | 5% Trimmed Mean | | 4.92 |  |
|  |  | Median | | 5.00 |  |
|  |  | Variance | | .182 |  |
|  |  | Std. Deviation | | .426 |  |
|  |  | Minimum | | 3 |  |
|  |  | Maximum | | 5 |  |
|  |  | Range | | 2 |  |
|  |  | Interquartile Range | | 0 |  |
|  |  | Skewness | | -2.959 | .178 |
|  |  | Kurtosis | | 8.395 | .354 |
|  | 55+ | Mean | | 4.86 | .058 |
|  |  | 95% Confidence Interval for Mean | Lower Bound | 4.74 |  |
|  |  |  | Upper Bound | 4.98 |  |
|  |  | 5% Trimmed Mean | | 4.93 |  |
|  |  | Median | | 5.00 |  |
|  |  | Variance | | .218 |  |
|  |  | Std. Deviation | | .467 |  |
|  |  | Minimum | | 2 |  |
|  |  | Maximum | | 5 |  |
|  |  | Range | | 3 |  |
|  |  | Interquartile Range | | 0 |  |
|  |  | Skewness | | -4.351 | .299 |
|  |  | Kurtosis | | 22.619 | .590 |
| On the scale shown, how much do you agree with the following statements? [There is not much people like me can do to stop antibiotic resistance] | 18-34 | Mean | | 2.87 | .077 |
|  |  | 95% Confidence Interval for Mean | Lower Bound | 2.72 |  |
|  |  |  | Upper Bound | 3.02 |  |
|  |  | 5% Trimmed Mean | | 2.86 |  |
|  |  | Median | | 3.00 |  |
|  |  | Variance | | 1.327 |  |
|  |  | Std. Deviation | | 1.152 |  |
|  |  | Minimum | | 1 |  |
|  |  | Maximum | | 5 |  |
|  |  | Range | | 4 |  |
|  |  | Interquartile Range | | 2 |  |
|  |  | Skewness | | .007 | .162 |
|  |  | Kurtosis | | -.769 | .323 |
|  | 35-54 | Mean | | 2.74 | .093 |
|  |  | 95% Confidence Interval for Mean | Lower Bound | 2.56 |  |
|  |  |  | Upper Bound | 2.92 |  |
|  |  | 5% Trimmed Mean | | 2.71 |  |
|  |  | Median | | 3.00 |  |
|  |  | Variance | | 1.603 |  |
|  |  | Std. Deviation | | 1.266 |  |
|  |  | Minimum | | 1 |  |
|  |  | Maximum | | 5 |  |
|  |  | Range | | 4 |  |
|  |  | Interquartile Range | | 2 |  |
|  |  | Skewness | | .072 | .178 |
|  |  | Kurtosis | | -1.106 | .354 |
|  | 55+ | Mean | | 3.11 | .167 |
|  |  | 95% Confidence Interval for Mean | Lower Bound | 2.78 |  |
|  |  |  | Upper Bound | 3.44 |  |
|  |  | 5% Trimmed Mean | | 3.12 |  |
|  |  | Median | | 3.00 |  |
|  |  | Variance | | 1.781 |  |
|  |  | Std. Deviation | | 1.335 |  |
|  |  | Minimum | | 1 |  |
|  |  | Maximum | | 5 |  |
|  |  | Range | | 4 |  |
|  |  | Interquartile Range | | 2 |  |
|  |  | Skewness | | -.206 | .299 |
|  |  | Kurtosis | | -1.179 | .590 |
| On the scale shown, how much do you agree with the following statements? [I am worried about the impact that antibiotic resistance will have on my health, and that of my family] | 18-34 | Mean | | 3.80 | .069 |
|  |  | 95% Confidence Interval for Mean | Lower Bound | 3.66 |  |
|  |  |  | Upper Bound | 3.93 |  |
|  |  | 5% Trimmed Mean | | 3.85 |  |
|  |  | Median | | 4.00 |  |
|  |  | Variance | | 1.074 |  |
|  |  | Std. Deviation | | 1.036 |  |
|  |  | Minimum | | 1 |  |
|  |  | Maximum | | 5 |  |
|  |  | Range | | 4 |  |
|  |  | Interquartile Range | | 2 |  |
|  |  | Skewness | | -.528 | .162 |
|  |  | Kurtosis | | -.375 | .323 |
|  | 35-54 | Mean | | 3.93 | .078 |
|  |  | 95% Confidence Interval for Mean | Lower Bound | 3.77 |  |
|  |  |  | Upper Bound | 4.08 |  |
|  |  | 5% Trimmed Mean | | 4.01 |  |
|  |  | Median | | 4.00 |  |
|  |  | Variance | | 1.123 |  |
|  |  | Std. Deviation | | 1.060 |  |
|  |  | Minimum | | 1 |  |
|  |  | Maximum | | 5 |  |
|  |  | Range | | 4 |  |
|  |  | Interquartile Range | | 2 |  |
|  |  | Skewness | | -.863 | .178 |
|  |  | Kurtosis | | .182 | .354 |
|  | 55+ | Mean | | 4.05 | .138 |
|  |  | 95% Confidence Interval for Mean | Lower Bound | 3.77 |  |
|  |  |  | Upper Bound | 4.32 |  |
|  |  | 5% Trimmed Mean | | 4.16 |  |
|  |  | Median | | 4.00 |  |
|  |  | Variance | | 1.220 |  |
|  |  | Std. Deviation | | 1.105 |  |
|  |  | Minimum | | 1 |  |
|  |  | Maximum | | 5 |  |
|  |  | Range | | 4 |  |
|  |  | Interquartile Range | | 1 |  |
|  |  | Skewness | | -1.335 | .299 |
|  |  | Kurtosis | | 1.504 | .590 |
| On the scale shown, how much do you agree with the following statements? [I am not at risk of getting an antibiotic-resistant infection, as long as I take my antibiotics correctly] | 18-34 | Mean | | 2.72 | .083 |
|  |  | 95% Confidence Interval for Mean | Lower Bound | 2.56 |  |
|  |  |  | Upper Bound | 2.88 |  |
|  |  | 5% Trimmed Mean | | 2.69 |  |
|  |  | Median | | 3.00 |  |
|  |  | Variance | | 1.551 |  |
|  |  | Std. Deviation | | 1.245 |  |
|  |  | Minimum | | 1 |  |
|  |  | Maximum | | 5 |  |
|  |  | Range | | 4 |  |
|  |  | Interquartile Range | | 2 |  |
|  |  | Skewness | | .139 | .162 |
|  |  | Kurtosis | | -.998 | .323 |
|  | 35-54 | Mean | | 2.84 | .103 |
|  |  | 95% Confidence Interval for Mean | Lower Bound | 2.64 |  |
|  |  |  | Upper Bound | 3.05 |  |
|  |  | 5% Trimmed Mean | | 2.83 |  |
|  |  | Median | | 3.00 |  |
|  |  | Variance | | 1.981 |  |
|  |  | Std. Deviation | | 1.408 |  |
|  |  | Minimum | | 1 |  |
|  |  | Maximum | | 5 |  |
|  |  | Range | | 4 |  |
|  |  | Interquartile Range | | 2 |  |
|  |  | Skewness | | .150 | .178 |
|  |  | Kurtosis | | -1.222 | .354 |
|  | 55+ | Mean | | 3.02 | .172 |
|  |  | 95% Confidence Interval for Mean | Lower Bound | 2.67 |  |
|  |  |  | Upper Bound | 3.36 |  |
|  |  | 5% Trimmed Mean | | 3.02 |  |
|  |  | Median | | 3.00 |  |
|  |  | Variance | | 1.889 |  |
|  |  | Std. Deviation | | 1.374 |  |
|  |  | Minimum | | 1 |  |
|  |  | Maximum | | 5 |  |
|  |  | Range | | 4 |  |
|  |  | Interquartile Range | | 2 |  |
|  |  | Skewness | | -.256 | .299 |
|  |  | Kurtosis | | -1.145 | .590 |

| **Tests of Normality** | | | | | | | |
| --- | --- | --- | --- | --- | --- | --- | --- |
|  | AgeGroup | Kolmogorov-Smirnov^a^ | | | Shapiro-Wilk | | |
|  |  | Statistic | df | Sig. | Statistic | df | Sig. |
| On the scale shown, how much do you agree the following actions would help address the problem of antibiotic resistance? [People should use antibiotics only when they are prescribed by a doctor or nurse] | 18-34 | .489 | 225 | <.001 | .471 | 225 | <.001 |
|  | 35-54 | .531 | 187 | <.001 | .239 | 187 | <.001 |
|  | 55+ | .529 | 64 | <.001 | .142 | 64 | <.001 |
| On the scale shown, how much do you agree the following actions would help address the problem of antibiotic resistance? [Farmers should give fewer antibiotics to food-producing animals] | 18-34 | .306 | 225 | <.001 | .776 | 225 | <.001 |
|  | 35-54 | .442 | 187 | <.001 | .569 | 187 | <.001 |
|  | 55+ | .477 | 64 | <.001 | .371 | 64 | <.001 |
| On the scale shown, how much do you agree the following actions would help address the problem of antibiotic resistance? [People should not keep antibiotics and use them later for other illnesses] | 18-34 | .348 | 225 | <.001 | .717 | 225 | <.001 |
|  | 35-54 | .439 | 187 | <.001 | .564 | 187 | <.001 |
|  | 55+ | .459 | 64 | <.001 | .481 | 64 | <.001 |
| On the scale shown, how much do you agree the following actions would help address the problem of antibiotic resistance? [Parents should make sure all of their children’s vaccinations are up-to-date] | 18-34 | .486 | 225 | <.001 | .467 | 225 | <.001 |
|  | 35-54 | .502 | 187 | <.001 | .382 | 187 | <.001 |
|  | 55+ | .534 | 64 | <.001 | .105 | 64 | <.001 |
| On the scale shown, how much do you agree the following actions would help address the problem of antibiotic resistance? [People should wash their hands regularly] | 18-34 | .483 | 225 | <.001 | .468 | 225 | <.001 |
|  | 35-54 | .520 | 187 | <.001 | .313 | 187 | <.001 |
|  | 55+ | .533 | 64 | <.001 | .259 | 64 | <.001 |
| On the scale shown, how much do you agree the following actions would help address the problem of antibiotic resistance? [Doctors should only prescribe antibiotics when they are needed ] | 18-34 | .511 | 225 | <.001 | .346 | 225 | <.001 |
|  | 35-54 | .532 | 187 | <.001 | .135 | 187 | <.001 |
|  | 55+ | .540 | 64 | <.001 | .217 | 64 | <.001 |
| On the scale shown, how much do you agree the following actions would help address the problem of antibiotic resistance? [Governments should reward the development of new antibiotics] | 18-34 | .241 | 225 | <.001 | .834 | 225 | <.001 |
|  | 35-54 | .274 | 187 | <.001 | .795 | 187 | <.001 |
|  | 55+ | .301 | 64 | <.001 | .727 | 64 | <.001 |
| On the scale shown, how much do you agree the following actions would help address the problem of antibiotic resistance? [Pharmaceutical companies should develop new antibiotics] | 18-34 | .198 | 225 | <.001 | .859 | 225 | <.001 |
|  | 35-54 | .270 | 187 | <.001 | .791 | 187 | <.001 |
|  | 55+ | .381 | 64 | <.001 | .686 | 64 | <.001 |
| On the scale shown, how much do you agree with the following statements? [Antibiotic resistance is one of the biggest problems the world faces] | 18-34 | .192 | 225 | <.001 | .900 | 225 | <.001 |
|  | 35-54 | .208 | 187 | <.001 | .854 | 187 | <.001 |
|  | 55+ | .223 | 64 | <.001 | .851 | 64 | <.001 |
| On the scale shown, how much do you agree with the following statements? [Medical experts will solve the problem of antibiotic resistance before it becomes too serious] | 18-34 | .240 | 225 | <.001 | .893 | 225 | <.001 |
|  | 35-54 | .238 | 187 | <.001 | .893 | 187 | <.001 |
|  | 55+ | .216 | 64 | <.001 | .889 | 64 | <.001 |
| On the scale shown, how much do you agree with the following statements? [Everyone needs to take responsibility for using antibiotics responsibly] | 18-34 | .476 | 225 | <.001 | .499 | 225 | <.001 |
|  | 35-54 | .514 | 187 | <.001 | .390 | 187 | <.001 |
|  | 55+ | .509 | 64 | <.001 | .334 | 64 | <.001 |
| On the scale shown, how much do you agree with the following statements? [There is not much people like me can do to stop antibiotic resistance] | 18-34 | .176 | 225 | <.001 | .915 | 225 | <.001 |
|  | 35-54 | .156 | 187 | <.001 | .899 | 187 | <.001 |
|  | 55+ | .216 | 64 | <.001 | .892 | 64 | <.001 |
| On the scale shown, how much do you agree with the following statements? [I am worried about the impact that antibiotic resistance will have on my health, and that of my family] | 18-34 | .200 | 225 | <.001 | .872 | 225 | <.001 |
|  | 35-54 | .229 | 187 | <.001 | .843 | 187 | <.001 |
|  | 55+ | .249 | 64 | <.001 | .780 | 64 | <.001 |
| On the scale shown, how much do you agree with the following statements? [I am not at risk of getting an antibiotic-resistant infection, as long as I take my antibiotics correctly] | 18-34 | .158 | 225 | <.001 | .904 | 225 | <.001 |
|  | 35-54 | .154 | 187 | <.001 | .886 | 187 | <.001 |
|  | 55+ | .185 | 64 | <.001 | .875 | 64 | <.001 |
| a. Lilliefors Significance Correction | | | | | | | |

(2) EDUCATION AND LIKERT SCALES

| **Descriptives** | | | | | |
| --- | --- | --- | --- | --- | --- |
|  | EducationLevel | | | Statistic | Std. Error |
| On the scale shown, how much do you agree the following actions would help address the problem of antibiotic resistance? [People should use antibiotics only when they are prescribed by a doctor or nurse] | Low Education | Mean | | 4.77 | .059 |
|  |  | 95% Confidence Interval for Mean | Lower Bound | 4.65 |  |
|  |  |  | Upper Bound | 4.88 |  |
|  |  | 5% Trimmed Mean | | 4.86 |  |
|  |  | Median | | 5.00 |  |
|  |  | Variance | | .343 |  |
|  |  | Std. Deviation | | .586 |  |
|  |  | Minimum | | 2 |  |
|  |  | Maximum | | 5 |  |
|  |  | Range | | 3 |  |
|  |  | Interquartile Range | | 0 |  |
|  |  | Skewness | | -3.017 | .243 |
|  |  | Kurtosis | | 9.933 | .481 |
|  | Medium Education | Mean | | 4.92 | .024 |
|  |  | 95% Confidence Interval for Mean | Lower Bound | 4.87 |  |
|  |  |  | Upper Bound | 4.97 |  |
|  |  | 5% Trimmed Mean | | 4.98 |  |
|  |  | Median | | 5.00 |  |
|  |  | Variance | | .086 |  |
|  |  | Std. Deviation | | .294 |  |
|  |  | Minimum | | 3 |  |
|  |  | Maximum | | 5 |  |
|  |  | Range | | 2 |  |
|  |  | Interquartile Range | | 0 |  |
|  |  | Skewness | | -3.917 | .197 |
|  |  | Kurtosis | | 16.142 | .391 |
|  | High Education | Mean | | 4.85 | .033 |
|  |  | 95% Confidence Interval for Mean | Lower Bound | 4.78 |  |
|  |  |  | Upper Bound | 4.91 |  |
|  |  | 5% Trimmed Mean | | 4.94 |  |
|  |  | Median | | 5.00 |  |
|  |  | Variance | | .245 |  |
|  |  | Std. Deviation | | .495 |  |
|  |  | Minimum | | 1 |  |
|  |  | Maximum | | 5 |  |
|  |  | Range | | 4 |  |
|  |  | Interquartile Range | | 0 |  |
|  |  | Skewness | | -4.101 | .162 |
|  |  | Kurtosis | | 20.631 | .323 |
| On the scale shown, how much do you agree the following actions would help address the problem of antibiotic resistance? [Farmers should give fewer antibiotics to food-producing animals] | Low Education | Mean | | 4.46 | .088 |
|  |  | 95% Confidence Interval for Mean | Lower Bound | 4.29 |  |
|  |  |  | Upper Bound | 4.64 |  |
|  |  | 5% Trimmed Mean | | 4.55 |  |
|  |  | Median | | 5.00 |  |
|  |  | Variance | | .761 |  |
|  |  | Std. Deviation | | .873 |  |
|  |  | Minimum | | 1 |  |
|  |  | Maximum | | 5 |  |
|  |  | Range | | 4 |  |
|  |  | Interquartile Range | | 1 |  |
|  |  | Skewness | | -1.535 | .243 |
|  |  | Kurtosis | | 1.785 | .481 |
|  | Medium Education | Mean | | 4.53 | .065 |
|  |  | 95% Confidence Interval for Mean | Lower Bound | 4.40 |  |
|  |  |  | Upper Bound | 4.66 |  |
|  |  | 5% Trimmed Mean | | 4.62 |  |
|  |  | Median | | 5.00 |  |
|  |  | Variance | | .648 |  |
|  |  | Std. Deviation | | .805 |  |
|  |  | Minimum | | 1 |  |
|  |  | Maximum | | 5 |  |
|  |  | Range | | 4 |  |
|  |  | Interquartile Range | | 1 |  |
|  |  | Skewness | | -1.730 | .197 |
|  |  | Kurtosis | | 2.595 | .391 |
|  | High Education | Mean | | 4.32 | .065 |
|  |  | 95% Confidence Interval for Mean | Lower Bound | 4.19 |  |
|  |  |  | Upper Bound | 4.45 |  |
|  |  | 5% Trimmed Mean | | 4.42 |  |
|  |  | Median | | 5.00 |  |
|  |  | Variance | | .942 |  |
|  |  | Std. Deviation | | .970 |  |
|  |  | Minimum | | 1 |  |
|  |  | Maximum | | 5 |  |
|  |  | Range | | 4 |  |
|  |  | Interquartile Range | | 1 |  |
|  |  | Skewness | | -1.389 | .162 |
|  |  | Kurtosis | | 1.462 | .323 |
| On the scale shown, how much do you agree the following actions would help address the problem of antibiotic resistance? [People should not keep antibiotics and use them later for other illnesses] | Low Education | Mean | | 4.34 | .107 |
|  |  | 95% Confidence Interval for Mean | Lower Bound | 4.13 |  |
|  |  |  | Upper Bound | 4.56 |  |
|  |  | 5% Trimmed Mean | | 4.46 |  |
|  |  | Median | | 5.00 |  |
|  |  | Variance | | 1.126 |  |
|  |  | Std. Deviation | | 1.061 |  |
|  |  | Minimum | | 1 |  |
|  |  | Maximum | | 5 |  |
|  |  | Range | | 4 |  |
|  |  | Interquartile Range | | 1 |  |
|  |  | Skewness | | -1.514 | .243 |
|  |  | Kurtosis | | 1.264 | .481 |
|  | Medium Education | Mean | | 4.57 | .065 |
|  |  | 95% Confidence Interval for Mean | Lower Bound | 4.44 |  |
|  |  |  | Upper Bound | 4.69 |  |
|  |  | 5% Trimmed Mean | | 4.67 |  |
|  |  | Median | | 5.00 |  |
|  |  | Variance | | .645 |  |
|  |  | Std. Deviation | | .803 |  |
|  |  | Minimum | | 1 |  |
|  |  | Maximum | | 5 |  |
|  |  | Range | | 4 |  |
|  |  | Interquartile Range | | 1 |  |
|  |  | Skewness | | -2.006 | .197 |
|  |  | Kurtosis | | 3.799 | .391 |
|  | High Education | Mean | | 4.44 | .063 |
|  |  | 95% Confidence Interval for Mean | Lower Bound | 4.32 |  |
|  |  |  | Upper Bound | 4.56 |  |
|  |  | 5% Trimmed Mean | | 4.56 |  |
|  |  | Median | | 5.00 |  |
|  |  | Variance | | .890 |  |
|  |  | Std. Deviation | | .944 |  |
|  |  | Minimum | | 1 |  |
|  |  | Maximum | | 5 |  |
|  |  | Range | | 4 |  |
|  |  | Interquartile Range | | 1 |  |
|  |  | Skewness | | -1.788 | .162 |
|  |  | Kurtosis | | 2.661 | .323 |
| On the scale shown, how much do you agree the following actions would help address the problem of antibiotic resistance? [Parents should make sure all of their children’s vaccinations are up-to-date] | Low Education | Mean | | 4.76 | .069 |
|  |  | 95% Confidence Interval for Mean | Lower Bound | 4.62 |  |
|  |  |  | Upper Bound | 4.89 |  |
|  |  | 5% Trimmed Mean | | 4.89 |  |
|  |  | Median | | 5.00 |  |
|  |  | Variance | | .471 |  |
|  |  | Std. Deviation | | .686 |  |
|  |  | Minimum | | 1 |  |
|  |  | Maximum | | 5 |  |
|  |  | Range | | 4 |  |
|  |  | Interquartile Range | | 0 |  |
|  |  | Skewness | | -3.509 | .243 |
|  |  | Kurtosis | | 13.402 | .481 |
|  | Medium Education | Mean | | 4.76 | .048 |
|  |  | 95% Confidence Interval for Mean | Lower Bound | 4.67 |  |
|  |  |  | Upper Bound | 4.86 |  |
|  |  | 5% Trimmed Mean | | 4.86 |  |
|  |  | Median | | 5.00 |  |
|  |  | Variance | | .354 |  |
|  |  | Std. Deviation | | .595 |  |
|  |  | Minimum | | 2 |  |
|  |  | Maximum | | 5 |  |
|  |  | Range | | 3 |  |
|  |  | Interquartile Range | | 0 |  |
|  |  | Skewness | | -2.738 | .197 |
|  |  | Kurtosis | | 7.383 | .391 |
|  | High Education | Mean | | 4.84 | .033 |
|  |  | 95% Confidence Interval for Mean | Lower Bound | 4.78 |  |
|  |  |  | Upper Bound | 4.91 |  |
|  |  | 5% Trimmed Mean | | 4.94 |  |
|  |  | Median | | 5.00 |  |
|  |  | Variance | | .248 |  |
|  |  | Std. Deviation | | .498 |  |
|  |  | Minimum | | 2 |  |
|  |  | Maximum | | 5 |  |
|  |  | Range | | 3 |  |
|  |  | Interquartile Range | | 0 |  |
|  |  | Skewness | | -3.582 | .162 |
|  |  | Kurtosis | | 13.336 | .323 |
| On the scale shown, how much do you agree the following actions would help address the problem of antibiotic resistance? [People should wash their hands regularly] | Low Education | Mean | | 4.77 | .067 |
|  |  | 95% Confidence Interval for Mean | Lower Bound | 4.63 |  |
|  |  |  | Upper Bound | 4.90 |  |
|  |  | 5% Trimmed Mean | | 4.89 |  |
|  |  | Median | | 5.00 |  |
|  |  | Variance | | .445 |  |
|  |  | Std. Deviation | | .667 |  |
|  |  | Minimum | | 1 |  |
|  |  | Maximum | | 5 |  |
|  |  | Range | | 4 |  |
|  |  | Interquartile Range | | 0 |  |
|  |  | Skewness | | -3.479 | .243 |
|  |  | Kurtosis | | 13.430 | .481 |
|  | Medium Education | Mean | | 4.86 | .039 |
|  |  | 95% Confidence Interval for Mean | Lower Bound | 4.78 |  |
|  |  |  | Upper Bound | 4.93 |  |
|  |  | 5% Trimmed Mean | | 4.95 |  |
|  |  | Median | | 5.00 |  |
|  |  | Variance | | .231 |  |
|  |  | Std. Deviation | | .480 |  |
|  |  | Minimum | | 2 |  |
|  |  | Maximum | | 5 |  |
|  |  | Range | | 3 |  |
|  |  | Interquartile Range | | 0 |  |
|  |  | Skewness | | -3.662 | .197 |
|  |  | Kurtosis | | 13.865 | .391 |
|  | High Education | Mean | | 4.79 | .041 |
|  |  | 95% Confidence Interval for Mean | Lower Bound | 4.71 |  |
|  |  |  | Upper Bound | 4.87 |  |
|  |  | 5% Trimmed Mean | | 4.90 |  |
|  |  | Median | | 5.00 |  |
|  |  | Variance | | .374 |  |
|  |  | Std. Deviation | | .611 |  |
|  |  | Minimum | | 1 |  |
|  |  | Maximum | | 5 |  |
|  |  | Range | | 4 |  |
|  |  | Interquartile Range | | 0 |  |
|  |  | Skewness | | -3.629 | .162 |
|  |  | Kurtosis | | 15.373 | .323 |
| On the scale shown, how much do you agree the following actions would help address the problem of antibiotic resistance? [Doctors should only prescribe antibiotics when they are needed ] | Low Education | Mean | | 4.88 | .056 |
|  |  | 95% Confidence Interval for Mean | Lower Bound | 4.77 |  |
|  |  |  | Upper Bound | 4.99 |  |
|  |  | 5% Trimmed Mean | | 4.99 |  |
|  |  | Median | | 5.00 |  |
|  |  | Variance | | .312 |  |
|  |  | Std. Deviation | | .558 |  |
|  |  | Minimum | | 1 |  |
|  |  | Maximum | | 5 |  |
|  |  | Range | | 4 |  |
|  |  | Interquartile Range | | 0 |  |
|  |  | Skewness | | -5.429 | .243 |
|  |  | Kurtosis | | 31.445 | .481 |
|  | Medium Education | Mean | | 4.91 | .025 |
|  |  | 95% Confidence Interval for Mean | Lower Bound | 4.87 |  |
|  |  |  | Upper Bound | 4.96 |  |
|  |  | 5% Trimmed Mean | | 4.97 |  |
|  |  | Median | | 5.00 |  |
|  |  | Variance | | .092 |  |
|  |  | Std. Deviation | | .303 |  |
|  |  | Minimum | | 3 |  |
|  |  | Maximum | | 5 |  |
|  |  | Range | | 2 |  |
|  |  | Interquartile Range | | 0 |  |
|  |  | Skewness | | -3.691 | .197 |
|  |  | Kurtosis | | 14.155 | .391 |
|  | High Education | Mean | | 4.92 | .022 |
|  |  | 95% Confidence Interval for Mean | Lower Bound | 4.87 |  |
|  |  |  | Upper Bound | 4.96 |  |
|  |  | 5% Trimmed Mean | | 4.98 |  |
|  |  | Median | | 5.00 |  |
|  |  | Variance | | .113 |  |
|  |  | Std. Deviation | | .337 |  |
|  |  | Minimum | | 3 |  |
|  |  | Maximum | | 5 |  |
|  |  | Range | | 2 |  |
|  |  | Interquartile Range | | 0 |  |
|  |  | Skewness | | -4.299 | .162 |
|  |  | Kurtosis | | 18.934 | .323 |
| On the scale shown, how much do you agree the following actions would help address the problem of antibiotic resistance? [Governments should reward the development of new antibiotics] | Low Education | Mean | | 4.07 | .110 |
|  |  | 95% Confidence Interval for Mean | Lower Bound | 3.85 |  |
|  |  |  | Upper Bound | 4.29 |  |
|  |  | 5% Trimmed Mean | | 4.18 |  |
|  |  | Median | | 4.00 |  |
|  |  | Variance | | 1.189 |  |
|  |  | Std. Deviation | | 1.090 |  |
|  |  | Minimum | | 1 |  |
|  |  | Maximum | | 5 |  |
|  |  | Range | | 4 |  |
|  |  | Interquartile Range | | 2 |  |
|  |  | Skewness | | -1.010 | .243 |
|  |  | Kurtosis | | .445 | .481 |
|  | Medium Education | Mean | | 4.01 | .083 |
|  |  | 95% Confidence Interval for Mean | Lower Bound | 3.84 |  |
|  |  |  | Upper Bound | 4.17 |  |
|  |  | 5% Trimmed Mean | | 4.08 |  |
|  |  | Median | | 4.00 |  |
|  |  | Variance | | 1.040 |  |
|  |  | Std. Deviation | | 1.020 |  |
|  |  | Minimum | | 1 |  |
|  |  | Maximum | | 5 |  |
|  |  | Range | | 4 |  |
|  |  | Interquartile Range | | 2 |  |
|  |  | Skewness | | -.773 | .197 |
|  |  | Kurtosis | | -.050 | .391 |
|  | High Education | Mean | | 3.99 | .075 |
|  |  | 95% Confidence Interval for Mean | Lower Bound | 3.84 |  |
|  |  |  | Upper Bound | 4.13 |  |
|  |  | 5% Trimmed Mean | | 4.10 |  |
|  |  | Median | | 4.00 |  |
|  |  | Variance | | 1.263 |  |
|  |  | Std. Deviation | | 1.124 |  |
|  |  | Minimum | | 1 |  |
|  |  | Maximum | | 5 |  |
|  |  | Range | | 4 |  |
|  |  | Interquartile Range | | 2 |  |
|  |  | Skewness | | -.944 | .162 |
|  |  | Kurtosis | | .243 | .323 |
| On the scale shown, how much do you agree the following actions would help address the problem of antibiotic resistance? [Pharmaceutical companies should develop new antibiotics] | Low Education | Mean | | 3.99 | .110 |
|  |  | 95% Confidence Interval for Mean | Lower Bound | 3.77 |  |
|  |  |  | Upper Bound | 4.21 |  |
|  |  | 5% Trimmed Mean | | 4.09 |  |
|  |  | Median | | 4.00 |  |
|  |  | Variance | | 1.194 |  |
|  |  | Std. Deviation | | 1.093 |  |
|  |  | Minimum | | 1 |  |
|  |  | Maximum | | 5 |  |
|  |  | Range | | 4 |  |
|  |  | Interquartile Range | | 2 |  |
|  |  | Skewness | | -.890 | .243 |
|  |  | Kurtosis | | .228 | .481 |
|  | Medium Education | Mean | | 4.08 | .082 |
|  |  | 95% Confidence Interval for Mean | Lower Bound | 3.92 |  |
|  |  |  | Upper Bound | 4.24 |  |
|  |  | 5% Trimmed Mean | | 4.18 |  |
|  |  | Median | | 4.00 |  |
|  |  | Variance | | 1.027 |  |
|  |  | Std. Deviation | | 1.013 |  |
|  |  | Minimum | | 1 |  |
|  |  | Maximum | | 5 |  |
|  |  | Range | | 4 |  |
|  |  | Interquartile Range | | 2 |  |
|  |  | Skewness | | -1.050 | .197 |
|  |  | Kurtosis | | .845 | .391 |
|  | High Education | Mean | | 4.01 | .066 |
|  |  | 95% Confidence Interval for Mean | Lower Bound | 3.88 |  |
|  |  |  | Upper Bound | 4.14 |  |
|  |  | 5% Trimmed Mean | | 4.08 |  |
|  |  | Median | | 4.00 |  |
|  |  | Variance | | .982 |  |
|  |  | Std. Deviation | | .991 |  |
|  |  | Minimum | | 1 |  |
|  |  | Maximum | | 5 |  |
|  |  | Range | | 4 |  |
|  |  | Interquartile Range | | 2 |  |
|  |  | Skewness | | -.684 | .162 |
|  |  | Kurtosis | | -.258 | .323 |
| On the scale shown, how much do you agree with the following statements? [Antibiotic resistance is one of the biggest problems the world faces] | Low Education | Mean | | 3.56 | .103 |
|  |  | 95% Confidence Interval for Mean | Lower Bound | 3.35 |  |
|  |  |  | Upper Bound | 3.76 |  |
|  |  | 5% Trimmed Mean | | 3.60 |  |
|  |  | Median | | 3.00 |  |
|  |  | Variance | | 1.045 |  |
|  |  | Std. Deviation | | 1.022 |  |
|  |  | Minimum | | 1 |  |
|  |  | Maximum | | 5 |  |
|  |  | Range | | 4 |  |
|  |  | Interquartile Range | | 1 |  |
|  |  | Skewness | | -.182 | .243 |
|  |  | Kurtosis | | -.360 | .481 |
|  | Medium Education | Mean | | 3.61 | .087 |
|  |  | 95% Confidence Interval for Mean | Lower Bound | 3.43 |  |
|  |  |  | Upper Bound | 3.78 |  |
|  |  | 5% Trimmed Mean | | 3.65 |  |
|  |  | Median | | 4.00 |  |
|  |  | Variance | | 1.141 |  |
|  |  | Std. Deviation | | 1.068 |  |
|  |  | Minimum | | 1 |  |
|  |  | Maximum | | 5 |  |
|  |  | Range | | 4 |  |
|  |  | Interquartile Range | | 2 |  |
|  |  | Skewness | | -.245 | .197 |
|  |  | Kurtosis | | -.665 | .391 |
|  | High Education | Mean | | 3.70 | .078 |
|  |  | 95% Confidence Interval for Mean | Lower Bound | 3.54 |  |
|  |  |  | Upper Bound | 3.85 |  |
|  |  | 5% Trimmed Mean | | 3.77 |  |
|  |  | Median | | 4.00 |  |
|  |  | Variance | | 1.381 |  |
|  |  | Std. Deviation | | 1.175 |  |
|  |  | Minimum | | 1 |  |
|  |  | Maximum | | 5 |  |
|  |  | Range | | 4 |  |
|  |  | Interquartile Range | | 2 |  |
|  |  | Skewness | | -.525 | .162 |
|  |  | Kurtosis | | -.614 | .323 |
| On the scale shown, how much do you agree with the following statements? [Medical experts will solve the problem of antibiotic resistance before it becomes too serious] | Low Education | Mean | | 3.21 | .117 |
|  |  | 95% Confidence Interval for Mean | Lower Bound | 2.98 |  |
|  |  |  | Upper Bound | 3.44 |  |
|  |  | 5% Trimmed Mean | | 3.24 |  |
|  |  | Median | | 3.00 |  |
|  |  | Variance | | 1.353 |  |
|  |  | Std. Deviation | | 1.163 |  |
|  |  | Minimum | | 1 |  |
|  |  | Maximum | | 5 |  |
|  |  | Range | | 4 |  |
|  |  | Interquartile Range | | 1 |  |
|  |  | Skewness | | .011 | .243 |
|  |  | Kurtosis | | -.582 | .481 |
|  | Medium Education | Mean | | 2.97 | .085 |
|  |  | 95% Confidence Interval for Mean | Lower Bound | 2.81 |  |
|  |  |  | Upper Bound | 3.14 |  |
|  |  | 5% Trimmed Mean | | 2.97 |  |
|  |  | Median | | 3.00 |  |
|  |  | Variance | | 1.085 |  |
|  |  | Std. Deviation | | 1.042 |  |
|  |  | Minimum | | 1 |  |
|  |  | Maximum | | 5 |  |
|  |  | Range | | 4 |  |
|  |  | Interquartile Range | | 2 |  |
|  |  | Skewness | | .017 | .197 |
|  |  | Kurtosis | | -.208 | .391 |
|  | High Education | Mean | | 2.80 | .066 |
|  |  | 95% Confidence Interval for Mean | Lower Bound | 2.67 |  |
|  |  |  | Upper Bound | 2.93 |  |
|  |  | 5% Trimmed Mean | | 2.78 |  |
|  |  | Median | | 3.00 |  |
|  |  | Variance | | .991 |  |
|  |  | Std. Deviation | | .996 |  |
|  |  | Minimum | | 1 |  |
|  |  | Maximum | | 5 |  |
|  |  | Range | | 4 |  |
|  |  | Interquartile Range | | 1 |  |
|  |  | Skewness | | .055 | .162 |
|  |  | Kurtosis | | -.095 | .323 |
| On the scale shown, how much do you agree with the following statements? [Everyone needs to take responsibility for using antibiotics responsibly] | Low Education | Mean | | 4.75 | .060 |
|  |  | 95% Confidence Interval for Mean | Lower Bound | 4.63 |  |
|  |  |  | Upper Bound | 4.87 |  |
|  |  | 5% Trimmed Mean | | 4.84 |  |
|  |  | Median | | 5.00 |  |
|  |  | Variance | | .354 |  |
|  |  | Std. Deviation | | .595 |  |
|  |  | Minimum | | 2 |  |
|  |  | Maximum | | 5 |  |
|  |  | Range | | 3 |  |
|  |  | Interquartile Range | | 0 |  |
|  |  | Skewness | | -2.529 | .243 |
|  |  | Kurtosis | | 6.231 | .481 |
|  | Medium Education | Mean | | 4.86 | .030 |
|  |  | 95% Confidence Interval for Mean | Lower Bound | 4.80 |  |
|  |  |  | Upper Bound | 4.91 |  |
|  |  | 5% Trimmed Mean | | 4.90 |  |
|  |  | Median | | 5.00 |  |
|  |  | Variance | | .138 |  |
|  |  | Std. Deviation | | .371 |  |
|  |  | Minimum | | 3 |  |
|  |  | Maximum | | 5 |  |
|  |  | Range | | 2 |  |
|  |  | Interquartile Range | | 0 |  |
|  |  | Skewness | | -2.426 | .197 |
|  |  | Kurtosis | | 5.137 | .391 |
|  | High Education | Mean | | 4.80 | .035 |
|  |  | 95% Confidence Interval for Mean | Lower Bound | 4.73 |  |
|  |  |  | Upper Bound | 4.87 |  |
|  |  | 5% Trimmed Mean | | 4.88 |  |
|  |  | Median | | 5.00 |  |
|  |  | Variance | | .279 |  |
|  |  | Std. Deviation | | .529 |  |
|  |  | Minimum | | 2 |  |
|  |  | Maximum | | 5 |  |
|  |  | Range | | 3 |  |
|  |  | Interquartile Range | | 0 |  |
|  |  | Skewness | | -2.918 | .162 |
|  |  | Kurtosis | | 9.036 | .323 |
| On the scale shown, how much do you agree with the following statements? [There is not much people like me can do to stop antibiotic resistance] | Low Education | Mean | | 3.02 | .123 |
|  |  | 95% Confidence Interval for Mean | Lower Bound | 2.78 |  |
|  |  |  | Upper Bound | 3.26 |  |
|  |  | 5% Trimmed Mean | | 3.02 |  |
|  |  | Median | | 3.00 |  |
|  |  | Variance | | 1.489 |  |
|  |  | Std. Deviation | | 1.220 |  |
|  |  | Minimum | | 1 |  |
|  |  | Maximum | | 5 |  |
|  |  | Range | | 4 |  |
|  |  | Interquartile Range | | 2 |  |
|  |  | Skewness | | -.142 | .243 |
|  |  | Kurtosis | | -.787 | .481 |
|  | Medium Education | Mean | | 2.91 | .099 |
|  |  | 95% Confidence Interval for Mean | Lower Bound | 2.71 |  |
|  |  |  | Upper Bound | 3.10 |  |
|  |  | 5% Trimmed Mean | | 2.90 |  |
|  |  | Median | | 3.00 |  |
|  |  | Variance | | 1.488 |  |
|  |  | Std. Deviation | | 1.220 |  |
|  |  | Minimum | | 1 |  |
|  |  | Maximum | | 5 |  |
|  |  | Range | | 4 |  |
|  |  | Interquartile Range | | 2 |  |
|  |  | Skewness | | -.044 | .197 |
|  |  | Kurtosis | | -1.062 | .391 |
|  | High Education | Mean | | 2.74 | .082 |
|  |  | 95% Confidence Interval for Mean | Lower Bound | 2.58 |  |
|  |  |  | Upper Bound | 2.90 |  |
|  |  | 5% Trimmed Mean | | 2.71 |  |
|  |  | Median | | 3.00 |  |
|  |  | Variance | | 1.507 |  |
|  |  | Std. Deviation | | 1.228 |  |
|  |  | Minimum | | 1 |  |
|  |  | Maximum | | 5 |  |
|  |  | Range | | 4 |  |
|  |  | Interquartile Range | | 2 |  |
|  |  | Skewness | | .104 | .162 |
|  |  | Kurtosis | | -.970 | .323 |
| On the scale shown, how much do you agree with the following statements? [I am worried about the impact that antibiotic resistance will have on my health, and that of my family] | Low Education | Mean | | 3.72 | .119 |
|  |  | 95% Confidence Interval for Mean | Lower Bound | 3.48 |  |
|  |  |  | Upper Bound | 3.95 |  |
|  |  | 5% Trimmed Mean | | 3.80 |  |
|  |  | Median | | 4.00 |  |
|  |  | Variance | | 1.409 |  |
|  |  | Std. Deviation | | 1.187 |  |
|  |  | Minimum | | 1 |  |
|  |  | Maximum | | 5 |  |
|  |  | Range | | 4 |  |
|  |  | Interquartile Range | | 2 |  |
|  |  | Skewness | | -.739 | .243 |
|  |  | Kurtosis | | -.182 | .481 |
|  | Medium Education | Mean | | 3.93 | .079 |
|  |  | 95% Confidence Interval for Mean | Lower Bound | 3.77 |  |
|  |  |  | Upper Bound | 4.08 |  |
|  |  | 5% Trimmed Mean | | 3.99 |  |
|  |  | Median | | 4.00 |  |
|  |  | Variance | | .955 |  |
|  |  | Std. Deviation | | .977 |  |
|  |  | Minimum | | 1 |  |
|  |  | Maximum | | 5 |  |
|  |  | Range | | 4 |  |
|  |  | Interquartile Range | | 2 |  |
|  |  | Skewness | | -.630 | .197 |
|  |  | Kurtosis | | -.196 | .391 |
|  | High Education | Mean | | 3.92 | .070 |
|  |  | 95% Confidence Interval for Mean | Lower Bound | 3.78 |  |
|  |  |  | Upper Bound | 4.06 |  |
|  |  | 5% Trimmed Mean | | 4.00 |  |
|  |  | Median | | 4.00 |  |
|  |  | Variance | | 1.092 |  |
|  |  | Std. Deviation | | 1.045 |  |
|  |  | Minimum | | 1 |  |
|  |  | Maximum | | 5 |  |
|  |  | Range | | 4 |  |
|  |  | Interquartile Range | | 2 |  |
|  |  | Skewness | | -.786 | .162 |
|  |  | Kurtosis | | .025 | .323 |
| On the scale shown, how much do you agree with the following statements? [I am not at risk of getting an antibiotic-resistant infection, as long as I take my antibiotics correctly] | Low Education | Mean | | 3.16 | .131 |
|  |  | 95% Confidence Interval for Mean | Lower Bound | 2.90 |  |
|  |  |  | Upper Bound | 3.42 |  |
|  |  | 5% Trimmed Mean | | 3.18 |  |
|  |  | Median | | 3.00 |  |
|  |  | Variance | | 1.708 |  |
|  |  | Std. Deviation | | 1.307 |  |
|  |  | Minimum | | 1 |  |
|  |  | Maximum | | 5 |  |
|  |  | Range | | 4 |  |
|  |  | Interquartile Range | | 2 |  |
|  |  | Skewness | | -.166 | .243 |
|  |  | Kurtosis | | -.913 | .481 |
|  | Medium Education | Mean | | 2.83 | .106 |
|  |  | 95% Confidence Interval for Mean | Lower Bound | 2.62 |  |
|  |  |  | Upper Bound | 3.04 |  |
|  |  | 5% Trimmed Mean | | 2.81 |  |
|  |  | Median | | 3.00 |  |
|  |  | Variance | | 1.706 |  |
|  |  | Std. Deviation | | 1.306 |  |
|  |  | Minimum | | 1 |  |
|  |  | Maximum | | 5 |  |
|  |  | Range | | 4 |  |
|  |  | Interquartile Range | | 2 |  |
|  |  | Skewness | | .069 | .197 |
|  |  | Kurtosis | | -1.089 | .391 |
|  | High Education | Mean | | 2.64 | .089 |
|  |  | 95% Confidence Interval for Mean | Lower Bound | 2.47 |  |
|  |  |  | Upper Bound | 2.81 |  |
|  |  | 5% Trimmed Mean | | 2.60 |  |
|  |  | Median | | 3.00 |  |
|  |  | Variance | | 1.767 |  |
|  |  | Std. Deviation | | 1.329 |  |
|  |  | Minimum | | 1 |  |
|  |  | Maximum | | 5 |  |
|  |  | Range | | 4 |  |
|  |  | Interquartile Range | | 3 |  |
|  |  | Skewness | | .259 | .162 |
|  |  | Kurtosis | | -1.126 | .323 |

| **Tests of Normality** | | | | | | | |
| --- | --- | --- | --- | --- | --- | --- | --- |
|  | EducationLevel | Kolmogorov-Smirnov^a^ | | | Shapiro-Wilk | | |
|  |  | Statistic | df | Sig. | Statistic | df | Sig. |
| On the scale shown, how much do you agree the following actions would help address the problem of antibiotic resistance? [People should use antibiotics only when they are prescribed by a doctor or nurse] | Low Education | .482 | 99 | <.001 | .450 | 99 | <.001 |
|  | Medium Education | .533 | 152 | <.001 | .286 | 152 | <.001 |
|  | High Education | .513 | 225 | <.001 | .342 | 225 | <.001 |
| On the scale shown, how much do you agree the following actions would help address the problem of antibiotic resistance? [Farmers should give fewer antibiotics to food-producing animals] | Low Education | .407 | 99 | <.001 | .650 | 99 | <.001 |
|  | Medium Education | .417 | 152 | <.001 | .630 | 152 | <.001 |
|  | High Education | .354 | 225 | <.001 | .713 | 225 | <.001 |
| On the scale shown, how much do you agree the following actions would help address the problem of antibiotic resistance? [People should not keep antibiotics and use them later for other illnesses] | Low Education | .389 | 99 | <.001 | .668 | 99 | <.001 |
|  | Medium Education | .423 | 152 | <.001 | .602 | 152 | <.001 |
|  | High Education | .390 | 225 | <.001 | .647 | 225 | <.001 |
| On the scale shown, how much do you agree the following actions would help address the problem of antibiotic resistance? [Parents should make sure all of their children’s vaccinations are up-to-date] | Low Education | .486 | 99 | <.001 | .407 | 99 | <.001 |
|  | Medium Education | .490 | 152 | <.001 | .455 | 152 | <.001 |
|  | High Education | .516 | 225 | <.001 | .351 | 225 | <.001 |
| On the scale shown, how much do you agree the following actions would help address the problem of antibiotic resistance? [People should wash their hands regularly] | Low Education | .495 | 99 | <.001 | .403 | 99 | <.001 |
|  | Medium Education | .520 | 152 | <.001 | .338 | 152 | <.001 |
|  | High Education | .494 | 225 | <.001 | .398 | 225 | <.001 |
| On the scale shown, how much do you agree the following actions would help address the problem of antibiotic resistance? [Doctors should only prescribe antibiotics when they are needed ] | Low Education | .525 | 99 | <.001 | .230 | 99 | <.001 |
|  | Medium Education | .532 | 152 | <.001 | .302 | 152 | <.001 |
|  | High Education | .532 | 225 | <.001 | .266 | 225 | <.001 |
| On the scale shown, how much do you agree the following actions would help address the problem of antibiotic resistance? [Governments should reward the development of new antibiotics] | Low Education | .288 | 99 | <.001 | .784 | 99 | <.001 |
|  | Medium Education | .243 | 152 | <.001 | .831 | 152 | <.001 |
|  | High Education | .261 | 225 | <.001 | .806 | 225 | <.001 |
| On the scale shown, how much do you agree the following actions would help address the problem of antibiotic resistance? [Pharmaceutical companies should develop new antibiotics] | Low Education | .257 | 99 | <.001 | .812 | 99 | <.001 |
|  | Medium Education | .253 | 152 | <.001 | .803 | 152 | <.001 |
|  | High Education | .241 | 225 | <.001 | .835 | 225 | <.001 |
| On the scale shown, how much do you agree with the following statements? [Antibiotic resistance is one of the biggest problems the world faces] | Low Education | .232 | 99 | <.001 | .880 | 99 | <.001 |
|  | Medium Education | .201 | 152 | <.001 | .887 | 152 | <.001 |
|  | High Education | .195 | 225 | <.001 | .870 | 225 | <.001 |
| On the scale shown, how much do you agree with the following statements? [Medical experts will solve the problem of antibiotic resistance before it becomes too serious] | Low Education | .239 | 99 | <.001 | .890 | 99 | <.001 |
|  | Medium Education | .227 | 152 | <.001 | .902 | 152 | <.001 |
|  | High Education | .242 | 225 | <.001 | .895 | 225 | <.001 |
| On the scale shown, how much do you agree with the following statements? [Everyone needs to take responsibility for using antibiotics responsibly] | Low Education | .483 | 99 | <.001 | .484 | 99 | <.001 |
|  | Medium Education | .514 | 152 | <.001 | .419 | 152 | <.001 |
|  | High Education | .495 | 225 | <.001 | .439 | 225 | <.001 |
| On the scale shown, how much do you agree with the following statements? [There is not much people like me can do to stop antibiotic resistance] | Low Education | .190 | 99 | <.001 | .908 | 99 | <.001 |
|  | Medium Education | .190 | 152 | <.001 | .905 | 152 | <.001 |
|  | High Education | .158 | 225 | <.001 | .906 | 225 | <.001 |
| On the scale shown, how much do you agree with the following statements? [I am worried about the impact that antibiotic resistance will have on my health, and that of my family] | Low Education | .210 | 99 | <.001 | .861 | 99 | <.001 |
|  | Medium Education | .214 | 152 | <.001 | .857 | 152 | <.001 |
|  | High Education | .219 | 225 | <.001 | .848 | 225 | <.001 |
| On the scale shown, how much do you agree with the following statements? [I am not at risk of getting an antibiotic-resistant infection, as long as I take my antibiotics correctly] | Low Education | .178 | 99 | <.001 | .895 | 99 | <.001 |
|  | Medium Education | .145 | 152 | <.001 | .902 | 152 | <.001 |
|  | High Education | .174 | 225 | <.001 | .888 | 225 | <.001 |
| a. Lilliefors Significance Correction | | | | | | | |

(3) GENDER AND LIKERT SCALE

| **Descriptives** | | | | | |
| --- | --- | --- | --- | --- | --- |
|  | What is your gender? | | | Statistic | Std. Error |
| On the scale shown, how much do you agree the following actions would help address the problem of antibiotic resistance? [People should use antibiotics only when they are prescribed by a doctor or nurse] | Male | Mean | | 4.87 | .030 |
|  |  | 95% Confidence Interval for Mean | Lower Bound | 4.81 |  |
|  |  |  | Upper Bound | 4.93 |  |
|  |  | 5% Trimmed Mean | | 4.93 |  |
|  |  | Median | | 5.00 |  |
|  |  | Variance | | .167 |  |
|  |  | Std. Deviation | | .409 |  |
|  |  | Minimum | | 2 |  |
|  |  | Maximum | | 5 |  |
|  |  | Range | | 3 |  |
|  |  | Interquartile Range | | 0 |  |
|  |  | Skewness | | -3.750 | .176 |
|  |  | Kurtosis | | 16.918 | .350 |
|  | Female | Mean | | 4.85 | .030 |
|  |  | 95% Confidence Interval for Mean | Lower Bound | 4.79 |  |
|  |  |  | Upper Bound | 4.90 |  |
|  |  | 5% Trimmed Mean | | 4.93 |  |
|  |  | Median | | 5.00 |  |
|  |  | Variance | | .251 |  |
|  |  | Std. Deviation | | .501 |  |
|  |  | Minimum | | 1 |  |
|  |  | Maximum | | 5 |  |
|  |  | Range | | 4 |  |
|  |  | Interquartile Range | | 0 |  |
|  |  | Skewness | | -4.010 | .144 |
|  |  | Kurtosis | | 19.163 | .288 |
| On the scale shown, how much do you agree the following actions would help address the problem of antibiotic resistance? [Farmers should give fewer antibiotics to food-producing animals] | Male | Mean | | 4.31 | .067 |
|  |  | 95% Confidence Interval for Mean | Lower Bound | 4.18 |  |
|  |  |  | Upper Bound | 4.44 |  |
|  |  | 5% Trimmed Mean | | 4.39 |  |
|  |  | Median | | 5.00 |  |
|  |  | Variance | | .857 |  |
|  |  | Std. Deviation | | .926 |  |
|  |  | Minimum | | 1 |  |
|  |  | Maximum | | 5 |  |
|  |  | Range | | 4 |  |
|  |  | Interquartile Range | | 1 |  |
|  |  | Skewness | | -1.137 | .176 |
|  |  | Kurtosis | | .560 | .350 |
|  | Female | Mean | | 4.49 | .052 |
|  |  | 95% Confidence Interval for Mean | Lower Bound | 4.39 |  |
|  |  |  | Upper Bound | 4.59 |  |
|  |  | 5% Trimmed Mean | | 4.60 |  |
|  |  | Median | | 5.00 |  |
|  |  | Variance | | .779 |  |
|  |  | Std. Deviation | | .883 |  |
|  |  | Minimum | | 1 |  |
|  |  | Maximum | | 5 |  |
|  |  | Range | | 4 |  |
|  |  | Interquartile Range | | 1 |  |
|  |  | Skewness | | -1.845 | .144 |
|  |  | Kurtosis | | 3.204 | .288 |
| On the scale shown, how much do you agree the following actions would help address the problem of antibiotic resistance? [People should not keep antibiotics and use them later for other illnesses] | Male | Mean | | 4.40 | .070 |
|  |  | 95% Confidence Interval for Mean | Lower Bound | 4.26 |  |
|  |  |  | Upper Bound | 4.54 |  |
|  |  | 5% Trimmed Mean | | 4.52 |  |
|  |  | Median | | 5.00 |  |
|  |  | Variance | | .936 |  |
|  |  | Std. Deviation | | .967 |  |
|  |  | Minimum | | 1 |  |
|  |  | Maximum | | 5 |  |
|  |  | Range | | 4 |  |
|  |  | Interquartile Range | | 1 |  |
|  |  | Skewness | | -1.612 | .176 |
|  |  | Kurtosis | | 1.885 | .350 |
|  | Female | Mean | | 4.50 | .053 |
|  |  | 95% Confidence Interval for Mean | Lower Bound | 4.40 |  |
|  |  |  | Upper Bound | 4.61 |  |
|  |  | 5% Trimmed Mean | | 4.63 |  |
|  |  | Median | | 5.00 |  |
|  |  | Variance | | .814 |  |
|  |  | Std. Deviation | | .902 |  |
|  |  | Minimum | | 1 |  |
|  |  | Maximum | | 5 |  |
|  |  | Range | | 4 |  |
|  |  | Interquartile Range | | 1 |  |
|  |  | Skewness | | -1.945 | .144 |
|  |  | Kurtosis | | 3.285 | .288 |
| On the scale shown, how much do you agree the following actions would help address the problem of antibiotic resistance? [Parents should make sure all of their children’s vaccinations are up-to-date] | Male | Mean | | 4.76 | .044 |
|  |  | 95% Confidence Interval for Mean | Lower Bound | 4.68 |  |
|  |  |  | Upper Bound | 4.85 |  |
|  |  | 5% Trimmed Mean | | 4.87 |  |
|  |  | Median | | 5.00 |  |
|  |  | Variance | | .371 |  |
|  |  | Std. Deviation | | .609 |  |
|  |  | Minimum | | 2 |  |
|  |  | Maximum | | 5 |  |
|  |  | Range | | 3 |  |
|  |  | Interquartile Range | | 0 |  |
|  |  | Skewness | | -2.942 | .176 |
|  |  | Kurtosis | | 8.799 | .350 |
|  | Female | Mean | | 4.82 | .032 |
|  |  | 95% Confidence Interval for Mean | Lower Bound | 4.76 |  |
|  |  |  | Upper Bound | 4.89 |  |
|  |  | 5% Trimmed Mean | | 4.93 |  |
|  |  | Median | | 5.00 |  |
|  |  | Variance | | .300 |  |
|  |  | Std. Deviation | | .548 |  |
|  |  | Minimum | | 1 |  |
|  |  | Maximum | | 5 |  |
|  |  | Range | | 4 |  |
|  |  | Interquartile Range | | 0 |  |
|  |  | Skewness | | -3.701 | .144 |
|  |  | Kurtosis | | 15.400 | .288 |
| On the scale shown, how much do you agree the following actions would help address the problem of antibiotic resistance? [People should wash their hands regularly] | Male | Mean | | 4.81 | .040 |
|  |  | 95% Confidence Interval for Mean | Lower Bound | 4.73 |  |
|  |  |  | Upper Bound | 4.89 |  |
|  |  | 5% Trimmed Mean | | 4.90 |  |
|  |  | Median | | 5.00 |  |
|  |  | Variance | | .301 |  |
|  |  | Std. Deviation | | .549 |  |
|  |  | Minimum | | 1 |  |
|  |  | Maximum | | 5 |  |
|  |  | Range | | 4 |  |
|  |  | Interquartile Range | | 0 |  |
|  |  | Skewness | | -3.746 | .176 |
|  |  | Kurtosis | | 17.069 | .350 |
|  | Female | Mean | | 4.80 | .036 |
|  |  | 95% Confidence Interval for Mean | Lower Bound | 4.73 |  |
|  |  |  | Upper Bound | 4.87 |  |
|  |  | 5% Trimmed Mean | | 4.91 |  |
|  |  | Median | | 5.00 |  |
|  |  | Variance | | .372 |  |
|  |  | Std. Deviation | | .610 |  |
|  |  | Minimum | | 1 |  |
|  |  | Maximum | | 5 |  |
|  |  | Range | | 4 |  |
|  |  | Interquartile Range | | 0 |  |
|  |  | Skewness | | -3.618 | .144 |
|  |  | Kurtosis | | 14.503 | .288 |
| On the scale shown, how much do you agree the following actions would help address the problem of antibiotic resistance? [Doctors should only prescribe antibiotics when they are needed ] | Male | Mean | | 4.88 | .034 |
|  |  | 95% Confidence Interval for Mean | Lower Bound | 4.82 |  |
|  |  |  | Upper Bound | 4.95 |  |
|  |  | 5% Trimmed Mean | | 4.97 |  |
|  |  | Median | | 5.00 |  |
|  |  | Variance | | .218 |  |
|  |  | Std. Deviation | | .467 |  |
|  |  | Minimum | | 1 |  |
|  |  | Maximum | | 5 |  |
|  |  | Range | | 4 |  |
|  |  | Interquartile Range | | 0 |  |
|  |  | Skewness | | -5.393 | .176 |
|  |  | Kurtosis | | 34.478 | .350 |
|  | Female | Mean | | 4.92 | .019 |
|  |  | 95% Confidence Interval for Mean | Lower Bound | 4.89 |  |
|  |  |  | Upper Bound | 4.96 |  |
|  |  | 5% Trimmed Mean | | 4.99 |  |
|  |  | Median | | 5.00 |  |
|  |  | Variance | | .100 |  |
|  |  | Std. Deviation | | .316 |  |
|  |  | Minimum | | 3 |  |
|  |  | Maximum | | 5 |  |
|  |  | Range | | 2 |  |
|  |  | Interquartile Range | | 0 |  |
|  |  | Skewness | | -4.432 | .144 |
|  |  | Kurtosis | | 20.448 | .288 |
| On the scale shown, how much do you agree the following actions would help address the problem of antibiotic resistance? [Governments should reward the development of new antibiotics] | Male | Mean | | 4.18 | .070 |
|  |  | 95% Confidence Interval for Mean | Lower Bound | 4.04 |  |
|  |  |  | Upper Bound | 4.32 |  |
|  |  | 5% Trimmed Mean | | 4.26 |  |
|  |  | Median | | 5.00 |  |
|  |  | Variance | | .947 |  |
|  |  | Std. Deviation | | .973 |  |
|  |  | Minimum | | 1 |  |
|  |  | Maximum | | 5 |  |
|  |  | Range | | 4 |  |
|  |  | Interquartile Range | | 2 |  |
|  |  | Skewness | | -.919 | .176 |
|  |  | Kurtosis | | .010 | .350 |
|  | Female | Mean | | 3.90 | .067 |
|  |  | 95% Confidence Interval for Mean | Lower Bound | 3.77 |  |
|  |  |  | Upper Bound | 4.03 |  |
|  |  | 5% Trimmed Mean | | 4.00 |  |
|  |  | Median | | 4.00 |  |
|  |  | Variance | | 1.296 |  |
|  |  | Std. Deviation | | 1.138 |  |
|  |  | Minimum | | 1 |  |
|  |  | Maximum | | 5 |  |
|  |  | Range | | 4 |  |
|  |  | Interquartile Range | | 2 |  |
|  |  | Skewness | | -.852 | .144 |
|  |  | Kurtosis | | .103 | .288 |
| On the scale shown, how much do you agree the following actions would help address the problem of antibiotic resistance? [Pharmaceutical companies should develop new antibiotics] | Male | Mean | | 4.17 | .068 |
|  |  | 95% Confidence Interval for Mean | Lower Bound | 4.04 |  |
|  |  |  | Upper Bound | 4.31 |  |
|  |  | 5% Trimmed Mean | | 4.26 |  |
|  |  | Median | | 4.00 |  |
|  |  | Variance | | .881 |  |
|  |  | Std. Deviation | | .938 |  |
|  |  | Minimum | | 1 |  |
|  |  | Maximum | | 5 |  |
|  |  | Range | | 4 |  |
|  |  | Interquartile Range | | 1 |  |
|  |  | Skewness | | -.931 | .176 |
|  |  | Kurtosis | | .251 | .350 |
|  | Female | Mean | | 3.93 | .063 |
|  |  | 95% Confidence Interval for Mean | Lower Bound | 3.81 |  |
|  |  |  | Upper Bound | 4.05 |  |
|  |  | 5% Trimmed Mean | | 4.02 |  |
|  |  | Median | | 4.00 |  |
|  |  | Variance | | 1.122 |  |
|  |  | Std. Deviation | | 1.059 |  |
|  |  | Minimum | | 1 |  |
|  |  | Maximum | | 5 |  |
|  |  | Range | | 4 |  |
|  |  | Interquartile Range | | 2 |  |
|  |  | Skewness | | -.772 | .144 |
|  |  | Kurtosis | | .083 | .288 |
| On the scale shown, how much do you agree with the following statements? [Antibiotic resistance is one of the biggest problems the world faces] | Male | Mean | | 3.56 | .079 |
|  |  | 95% Confidence Interval for Mean | Lower Bound | 3.40 |  |
|  |  |  | Upper Bound | 3.72 |  |
|  |  | 5% Trimmed Mean | | 3.61 |  |
|  |  | Median | | 4.00 |  |
|  |  | Variance | | 1.195 |  |
|  |  | Std. Deviation | | 1.093 |  |
|  |  | Minimum | | 1 |  |
|  |  | Maximum | | 5 |  |
|  |  | Range | | 4 |  |
|  |  | Interquartile Range | | 1 |  |
|  |  | Skewness | | -.302 | .176 |
|  |  | Kurtosis | | -.603 | .350 |
|  | Female | Mean | | 3.69 | .066 |
|  |  | 95% Confidence Interval for Mean | Lower Bound | 3.56 |  |
|  |  |  | Upper Bound | 3.82 |  |
|  |  | 5% Trimmed Mean | | 3.76 |  |
|  |  | Median | | 4.00 |  |
|  |  | Variance | | 1.256 |  |
|  |  | Std. Deviation | | 1.121 |  |
|  |  | Minimum | | 1 |  |
|  |  | Maximum | | 5 |  |
|  |  | Range | | 4 |  |
|  |  | Interquartile Range | | 2 |  |
|  |  | Skewness | | -.426 | .144 |
|  |  | Kurtosis | | -.589 | .288 |
| On the scale shown, how much do you agree with the following statements? [Medical experts will solve the problem of antibiotic resistance before it becomes too serious] | Male | Mean | | 2.98 | .072 |
|  |  | 95% Confidence Interval for Mean | Lower Bound | 2.84 |  |
|  |  |  | Upper Bound | 3.12 |  |
|  |  | 5% Trimmed Mean | | 2.98 |  |
|  |  | Median | | 3.00 |  |
|  |  | Variance | | .979 |  |
|  |  | Std. Deviation | | .989 |  |
|  |  | Minimum | | 1 |  |
|  |  | Maximum | | 5 |  |
|  |  | Range | | 4 |  |
|  |  | Interquartile Range | | 2 |  |
|  |  | Skewness | | .141 | .176 |
|  |  | Kurtosis | | -.145 | .350 |
|  | Female | Mean | | 2.92 | .065 |
|  |  | 95% Confidence Interval for Mean | Lower Bound | 2.79 |  |
|  |  |  | Upper Bound | 3.04 |  |
|  |  | 5% Trimmed Mean | | 2.91 |  |
|  |  | Median | | 3.00 |  |
|  |  | Variance | | 1.211 |  |
|  |  | Std. Deviation | | 1.101 |  |
|  |  | Minimum | | 1 |  |
|  |  | Maximum | | 5 |  |
|  |  | Range | | 4 |  |
|  |  | Interquartile Range | | 1 |  |
|  |  | Skewness | | .072 | .144 |
|  |  | Kurtosis | | -.315 | .288 |
| On the scale shown, how much do you agree with the following statements? [Everyone needs to take responsibility for using antibiotics responsibly] | Male | Mean | | 4.81 | .034 |
|  |  | 95% Confidence Interval for Mean | Lower Bound | 4.74 |  |
|  |  |  | Upper Bound | 4.87 |  |
|  |  | 5% Trimmed Mean | | 4.87 |  |
|  |  | Median | | 5.00 |  |
|  |  | Variance | | .220 |  |
|  |  | Std. Deviation | | .469 |  |
|  |  | Minimum | | 2 |  |
|  |  | Maximum | | 5 |  |
|  |  | Range | | 3 |  |
|  |  | Interquartile Range | | 0 |  |
|  |  | Skewness | | -2.744 | .176 |
|  |  | Kurtosis | | 8.831 | .350 |
|  | Female | Mean | | 4.80 | .031 |
|  |  | 95% Confidence Interval for Mean | Lower Bound | 4.74 |  |
|  |  |  | Upper Bound | 4.86 |  |
|  |  | 5% Trimmed Mean | | 4.89 |  |
|  |  | Median | | 5.00 |  |
|  |  | Variance | | .271 |  |
|  |  | Std. Deviation | | .521 |  |
|  |  | Minimum | | 2 |  |
|  |  | Maximum | | 5 |  |
|  |  | Range | | 3 |  |
|  |  | Interquartile Range | | 0 |  |
|  |  | Skewness | | -2.925 | .144 |
|  |  | Kurtosis | | 8.819 | .288 |
| On the scale shown, how much do you agree with the following statements? [There is not much people like me can do to stop antibiotic resistance] | Male | Mean | | 2.91 | .087 |
|  |  | 95% Confidence Interval for Mean | Lower Bound | 2.74 |  |
|  |  |  | Upper Bound | 3.08 |  |
|  |  | 5% Trimmed Mean | | 2.90 |  |
|  |  | Median | | 3.00 |  |
|  |  | Variance | | 1.439 |  |
|  |  | Std. Deviation | | 1.200 |  |
|  |  | Minimum | | 1 |  |
|  |  | Maximum | | 5 |  |
|  |  | Range | | 4 |  |
|  |  | Interquartile Range | | 2 |  |
|  |  | Skewness | | .007 | .176 |
|  |  | Kurtosis | | -.880 | .350 |
|  | Female | Mean | | 2.81 | .074 |
|  |  | 95% Confidence Interval for Mean | Lower Bound | 2.67 |  |
|  |  |  | Upper Bound | 2.96 |  |
|  |  | 5% Trimmed Mean | | 2.79 |  |
|  |  | Median | | 3.00 |  |
|  |  | Variance | | 1.548 |  |
|  |  | Std. Deviation | | 1.244 |  |
|  |  | Minimum | | 1 |  |
|  |  | Maximum | | 5 |  |
|  |  | Range | | 4 |  |
|  |  | Interquartile Range | | 2 |  |
|  |  | Skewness | | .011 | .144 |
|  |  | Kurtosis | | -1.053 | .288 |
| On the scale shown, how much do you agree with the following statements? [I am worried about the impact that antibiotic resistance will have on my health, and that of my family] | Male | Mean | | 3.85 | .071 |
|  |  | 95% Confidence Interval for Mean | Lower Bound | 3.71 |  |
|  |  |  | Upper Bound | 3.99 |  |
|  |  | 5% Trimmed Mean | | 3.90 |  |
|  |  | Median | | 4.00 |  |
|  |  | Variance | | .951 |  |
|  |  | Std. Deviation | | .975 |  |
|  |  | Minimum | | 1 |  |
|  |  | Maximum | | 5 |  |
|  |  | Range | | 4 |  |
|  |  | Interquartile Range | | 2 |  |
|  |  | Skewness | | -.586 | .176 |
|  |  | Kurtosis | | -.150 | .350 |
|  | Female | Mean | | 3.90 | .066 |
|  |  | 95% Confidence Interval for Mean | Lower Bound | 3.77 |  |
|  |  |  | Upper Bound | 4.03 |  |
|  |  | 5% Trimmed Mean | | 3.99 |  |
|  |  | Median | | 4.00 |  |
|  |  | Variance | | 1.230 |  |
|  |  | Std. Deviation | | 1.109 |  |
|  |  | Minimum | | 1 |  |
|  |  | Maximum | | 5 |  |
|  |  | Range | | 4 |  |
|  |  | Interquartile Range | | 2 |  |
|  |  | Skewness | | -.850 | .144 |
|  |  | Kurtosis | | .048 | .288 |
| On the scale shown, how much do you agree with the following statements? [I am not at risk of getting an antibiotic-resistant infection, as long as I take my antibiotics correctly] | Male | Mean | | 2.72 | .092 |
|  |  | 95% Confidence Interval for Mean | Lower Bound | 2.54 |  |
|  |  |  | Upper Bound | 2.90 |  |
|  |  | 5% Trimmed Mean | | 2.69 |  |
|  |  | Median | | 3.00 |  |
|  |  | Variance | | 1.612 |  |
|  |  | Std. Deviation | | 1.270 |  |
|  |  | Minimum | | 1 |  |
|  |  | Maximum | | 5 |  |
|  |  | Range | | 4 |  |
|  |  | Interquartile Range | | 2 |  |
|  |  | Skewness | | .161 | .176 |
|  |  | Kurtosis | | -1.076 | .350 |
|  | Female | Mean | | 2.87 | .081 |
|  |  | 95% Confidence Interval for Mean | Lower Bound | 2.71 |  |
|  |  |  | Upper Bound | 3.03 |  |
|  |  | 5% Trimmed Mean | | 2.85 |  |
|  |  | Median | | 3.00 |  |
|  |  | Variance | | 1.869 |  |
|  |  | Std. Deviation | | 1.367 |  |
|  |  | Minimum | | 1 |  |
|  |  | Maximum | | 5 |  |
|  |  | Range | | 4 |  |
|  |  | Interquartile Range | | 2 |  |
|  |  | Skewness | | .060 | .144 |
|  |  | Kurtosis | | -1.157 | .288 |

| **Tests of Normality** | | | | | | | |
| --- | --- | --- | --- | --- | --- | --- | --- |
|  | What is your gender? | Kolmogorov-Smirnov^a^ | | | Shapiro-Wilk | | |
|  |  | Statistic | df | Sig. | Statistic | df | Sig. |
| On the scale shown, how much do you agree the following actions would help address the problem of antibiotic resistance? [People should use antibiotics only when they are prescribed by a doctor or nurse] | Male | .516 | 191 | <.001 | .356 | 191 | <.001 |
|  | Female | .512 | 285 | <.001 | .347 | 285 | <.001 |
| On the scale shown, how much do you agree the following actions would help address the problem of antibiotic resistance? [Farmers should give fewer antibiotics to food-producing animals] | Male | .348 | 191 | <.001 | .733 | 191 | <.001 |
|  | Female | .409 | 285 | <.001 | .627 | 285 | <.001 |
| On the scale shown, how much do you agree the following actions would help address the problem of antibiotic resistance? [People should not keep antibiotics and use them later for other illnesses] | Male | .382 | 191 | <.001 | .670 | 191 | <.001 |
|  | Female | .411 | 285 | <.001 | .613 | 285 | <.001 |
| On the scale shown, how much do you agree the following actions would help address the problem of antibiotic resistance? [Parents should make sure all of their children’s vaccinations are up-to-date] | Male | .488 | 191 | <.001 | .441 | 191 | <.001 |
|  | Female | .510 | 285 | <.001 | .363 | 285 | <.001 |
| On the scale shown, how much do you agree the following actions would help address the problem of antibiotic resistance? [People should wash their hands regularly] | Male | .498 | 191 | <.001 | .391 | 191 | <.001 |
|  | Female | .506 | 285 | <.001 | .374 | 285 | <.001 |
| On the scale shown, how much do you agree the following actions would help address the problem of antibiotic resistance? [Doctors should only prescribe antibiotics when they are needed ] | Male | .519 | 191 | <.001 | .267 | 191 | <.001 |
|  | Female | .533 | 285 | <.001 | .257 | 285 | <.001 |
| On the scale shown, how much do you agree the following actions would help address the problem of antibiotic resistance? [Governments should reward the development of new antibiotics] | Male | .303 | 191 | <.001 | .784 | 191 | <.001 |
|  | Female | .233 | 285 | <.001 | .825 | 285 | <.001 |
| On the scale shown, how much do you agree the following actions would help address the problem of antibiotic resistance? [Pharmaceutical companies should develop new antibiotics] | Male | .282 | 191 | <.001 | .797 | 191 | <.001 |
|  | Female | .226 | 285 | <.001 | .836 | 285 | <.001 |
| On the scale shown, how much do you agree with the following statements? [Antibiotic resistance is one of the biggest problems the world faces] | Male | .183 | 191 | <.001 | .894 | 191 | <.001 |
|  | Female | .191 | 285 | <.001 | .872 | 285 | <.001 |
| On the scale shown, how much do you agree with the following statements? [Medical experts will solve the problem of antibiotic resistance before it becomes too serious] | Male | .235 | 191 | <.001 | .900 | 191 | <.001 |
|  | Female | .231 | 285 | <.001 | .895 | 285 | <.001 |
| On the scale shown, how much do you agree with the following statements? [Everyone needs to take responsibility for using antibiotics responsibly] | Male | .493 | 191 | <.001 | .458 | 191 | <.001 |
|  | Female | .500 | 285 | <.001 | .428 | 285 | <.001 |
| On the scale shown, how much do you agree with the following statements? [There is not much people like me can do to stop antibiotic resistance] | Male | .158 | 191 | <.001 | .915 | 191 | <.001 |
|  | Female | .160 | 285 | <.001 | .905 | 285 | <.001 |
| On the scale shown, how much do you agree with the following statements? [I am worried about the impact that antibiotic resistance will have on my health, and that of my family] | Male | .227 | 191 | <.001 | .868 | 191 | <.001 |
|  | Female | .214 | 285 | <.001 | .840 | 285 | <.001 |
| On the scale shown, how much do you agree with the following statements? [I am not at risk of getting an antibiotic-resistant infection, as long as I take my antibiotics correctly] | Male | .176 | 191 | <.001 | .901 | 191 | <.001 |
|  | Female | .149 | 285 | <.001 | .892 | 285 | <.001 |
| a. Lilliefors Significance Correction | | | | | | | |

(4) HOUSEHOLD INCOME AND LIKERT SCALE

| **Descriptives** | | | | | |
| --- | --- | --- | --- | --- | --- |
|  | HouseholdIncome | | | Statistic | Std. Error |
| On the scale shown, how much do you agree the following actions would help address the problem of antibiotic resistance? [People should use antibiotics only when they are prescribed by a doctor or nurse] | Up to Eur24999 | Mean | | 4.76 | .075 |
|  |  | 95% Confidence Interval for Mean | Lower Bound | 4.61 |  |
|  |  |  | Upper Bound | 4.91 |  |
|  |  | 5% Trimmed Mean | | 4.84 |  |
|  |  | Median | | 5.00 |  |
|  |  | Variance | | .327 |  |
|  |  | Std. Deviation | | .572 |  |
|  |  | Minimum | | 2 |  |
|  |  | Maximum | | 5 |  |
|  |  | Range | | 3 |  |
|  |  | Interquartile Range | | 0 |  |
|  |  | Skewness | | -2.890 | .314 |
|  |  | Kurtosis | | 9.733 | .618 |
|  | Eur25000-49999 | Mean | | 4.87 | .031 |
|  |  | 95% Confidence Interval for Mean | Lower Bound | 4.81 |  |
|  |  |  | Upper Bound | 4.93 |  |
|  |  | 5% Trimmed Mean | | 4.94 |  |
|  |  | Median | | 5.00 |  |
|  |  | Variance | | .161 |  |
|  |  | Std. Deviation | | .402 |  |
|  |  | Minimum | | 3 |  |
|  |  | Maximum | | 5 |  |
|  |  | Range | | 2 |  |
|  |  | Interquartile Range | | 0 |  |
|  |  | Skewness | | -3.309 | .190 |
|  |  | Kurtosis | | 10.788 | .377 |
|  | Eur50000-99999 | Mean | | 4.86 | .046 |
|  |  | 95% Confidence Interval for Mean | Lower Bound | 4.76 |  |
|  |  |  | Upper Bound | 4.95 |  |
|  |  | 5% Trimmed Mean | | 4.95 |  |
|  |  | Median | | 5.00 |  |
|  |  | Variance | | .269 |  |
|  |  | Std. Deviation | | .519 |  |
|  |  | Minimum | | 1 |  |
|  |  | Maximum | | 5 |  |
|  |  | Range | | 4 |  |
|  |  | Interquartile Range | | 0 |  |
|  |  | Skewness | | -4.758 | .217 |
|  |  | Kurtosis | | 27.364 | .430 |
|  | Eur100000+ | Mean | | 4.85 | .065 |
|  |  | 95% Confidence Interval for Mean | Lower Bound | 4.72 |  |
|  |  |  | Upper Bound | 4.98 |  |
|  |  | 5% Trimmed Mean | | 4.96 |  |
|  |  | Median | | 5.00 |  |
|  |  | Variance | | .280 |  |
|  |  | Std. Deviation | | .530 |  |
|  |  | Minimum | | 2 |  |
|  |  | Maximum | | 5 |  |
|  |  | Range | | 3 |  |
|  |  | Interquartile Range | | 0 |  |
|  |  | Skewness | | -3.943 | .293 |
|  |  | Kurtosis | | 16.086 | .578 |
|  | Prefer Not to Say | Mean | | 4.90 | .038 |
|  |  | 95% Confidence Interval for Mean | Lower Bound | 4.83 |  |
|  |  |  | Upper Bound | 4.98 |  |
|  |  | 5% Trimmed Mean | | 4.95 |  |
|  |  | Median | | 5.00 |  |
|  |  | Variance | | .089 |  |
|  |  | Std. Deviation | | .298 |  |
|  |  | Minimum | | 4 |  |
|  |  | Maximum | | 5 |  |
|  |  | Range | | 1 |  |
|  |  | Interquartile Range | | 0 |  |
|  |  | Skewness | | -2.796 | .304 |
|  |  | Kurtosis | | 6.010 | .599 |
| On the scale shown, how much do you agree the following actions would help address the problem of antibiotic resistance? [Farmers should give fewer antibiotics to food-producing animals] | Up to Eur24999 | Mean | | 4.17 | .130 |
|  |  | 95% Confidence Interval for Mean | Lower Bound | 3.91 |  |
|  |  |  | Upper Bound | 4.43 |  |
|  |  | 5% Trimmed Mean | | 4.25 |  |
|  |  | Median | | 5.00 |  |
|  |  | Variance | | .987 |  |
|  |  | Std. Deviation | | .994 |  |
|  |  | Minimum | | 1 |  |
|  |  | Maximum | | 5 |  |
|  |  | Range | | 4 |  |
|  |  | Interquartile Range | | 2 |  |
|  |  | Skewness | | -.915 | .314 |
|  |  | Kurtosis | | .155 | .618 |
|  | Eur25000-49999 | Mean | | 4.42 | .069 |
|  |  | 95% Confidence Interval for Mean | Lower Bound | 4.28 |  |
|  |  |  | Upper Bound | 4.56 |  |
|  |  | 5% Trimmed Mean | | 4.51 |  |
|  |  | Median | | 5.00 |  |
|  |  | Variance | | .785 |  |
|  |  | Std. Deviation | | .886 |  |
|  |  | Minimum | | 1 |  |
|  |  | Maximum | | 5 |  |
|  |  | Range | | 4 |  |
|  |  | Interquartile Range | | 1 |  |
|  |  | Skewness | | -1.471 | .190 |
|  |  | Kurtosis | | 1.707 | .377 |
|  | Eur50000-99999 | Mean | | 4.43 | .083 |
|  |  | 95% Confidence Interval for Mean | Lower Bound | 4.27 |  |
|  |  |  | Upper Bound | 4.60 |  |
|  |  | 5% Trimmed Mean | | 4.54 |  |
|  |  | Median | | 5.00 |  |
|  |  | Variance | | .860 |  |
|  |  | Std. Deviation | | .928 |  |
|  |  | Minimum | | 1 |  |
|  |  | Maximum | | 5 |  |
|  |  | Range | | 4 |  |
|  |  | Interquartile Range | | 1 |  |
|  |  | Skewness | | -1.647 | .217 |
|  |  | Kurtosis | | 2.224 | .430 |
|  | Eur100000+ | Mean | | 4.54 | .105 |
|  |  | 95% Confidence Interval for Mean | Lower Bound | 4.33 |  |
|  |  |  | Upper Bound | 4.75 |  |
|  |  | 5% Trimmed Mean | | 4.63 |  |
|  |  | Median | | 5.00 |  |
|  |  | Variance | | .737 |  |
|  |  | Std. Deviation | | .859 |  |
|  |  | Minimum | | 1 |  |
|  |  | Maximum | | 5 |  |
|  |  | Range | | 4 |  |
|  |  | Interquartile Range | | 1 |  |
|  |  | Skewness | | -1.895 | .293 |
|  |  | Kurtosis | | 3.430 | .578 |
|  | Prefer Not to Say | Mean | | 4.48 | .107 |
|  |  | 95% Confidence Interval for Mean | Lower Bound | 4.27 |  |
|  |  |  | Upper Bound | 4.70 |  |
|  |  | 5% Trimmed Mean | | 4.59 |  |
|  |  | Median | | 5.00 |  |
|  |  | Variance | | .713 |  |
|  |  | Std. Deviation | | .844 |  |
|  |  | Minimum | | 1 |  |
|  |  | Maximum | | 5 |  |
|  |  | Range | | 4 |  |
|  |  | Interquartile Range | | 1 |  |
|  |  | Skewness | | -1.974 | .304 |
|  |  | Kurtosis | | 4.439 | .599 |
| On the scale shown, how much do you agree the following actions would help address the problem of antibiotic resistance? [People should not keep antibiotics and use them later for other illnesses] | Up to Eur24999 | Mean | | 4.14 | .164 |
|  |  | 95% Confidence Interval for Mean | Lower Bound | 3.81 |  |
|  |  |  | Upper Bound | 4.47 |  |
|  |  | 5% Trimmed Mean | | 4.26 |  |
|  |  | Median | | 5.00 |  |
|  |  | Variance | | 1.560 |  |
|  |  | Std. Deviation | | 1.249 |  |
|  |  | Minimum | | 1 |  |
|  |  | Maximum | | 5 |  |
|  |  | Range | | 4 |  |
|  |  | Interquartile Range | | 1 |  |
|  |  | Skewness | | -1.278 | .314 |
|  |  | Kurtosis | | .389 | .618 |
|  | Eur25000-49999 | Mean | | 4.54 | .060 |
|  |  | 95% Confidence Interval for Mean | Lower Bound | 4.42 |  |
|  |  |  | Upper Bound | 4.66 |  |
|  |  | 5% Trimmed Mean | | 4.62 |  |
|  |  | Median | | 5.00 |  |
|  |  | Variance | | .593 |  |
|  |  | Std. Deviation | | .770 |  |
|  |  | Minimum | | 2 |  |
|  |  | Maximum | | 5 |  |
|  |  | Range | | 3 |  |
|  |  | Interquartile Range | | 1 |  |
|  |  | Skewness | | -1.531 | .190 |
|  |  | Kurtosis | | 1.292 | .377 |
|  | Eur50000-99999 | Mean | | 4.51 | .082 |
|  |  | 95% Confidence Interval for Mean | Lower Bound | 4.35 |  |
|  |  |  | Upper Bound | 4.68 |  |
|  |  | 5% Trimmed Mean | | 4.64 |  |
|  |  | Median | | 5.00 |  |
|  |  | Variance | | .849 |  |
|  |  | Std. Deviation | | .921 |  |
|  |  | Minimum | | 1 |  |
|  |  | Maximum | | 5 |  |
|  |  | Range | | 4 |  |
|  |  | Interquartile Range | | 1 |  |
|  |  | Skewness | | -2.080 | .217 |
|  |  | Kurtosis | | 3.816 | .430 |
|  | Eur100000+ | Mean | | 4.40 | .122 |
|  |  | 95% Confidence Interval for Mean | Lower Bound | 4.16 |  |
|  |  |  | Upper Bound | 4.65 |  |
|  |  | 5% Trimmed Mean | | 4.54 |  |
|  |  | Median | | 5.00 |  |
|  |  | Variance | | 1.002 |  |
|  |  | Std. Deviation | | 1.001 |  |
|  |  | Minimum | | 1 |  |
|  |  | Maximum | | 5 |  |
|  |  | Range | | 4 |  |
|  |  | Interquartile Range | | 1 |  |
|  |  | Skewness | | -1.827 | .293 |
|  |  | Kurtosis | | 2.942 | .578 |
|  | Prefer Not to Say | Mean | | 4.50 | .110 |
|  |  | 95% Confidence Interval for Mean | Lower Bound | 4.28 |  |
|  |  |  | Upper Bound | 4.72 |  |
|  |  | 5% Trimmed Mean | | 4.61 |  |
|  |  | Median | | 5.00 |  |
|  |  | Variance | | .746 |  |
|  |  | Std. Deviation | | .864 |  |
|  |  | Minimum | | 2 |  |
|  |  | Maximum | | 5 |  |
|  |  | Range | | 3 |  |
|  |  | Interquartile Range | | 1 |  |
|  |  | Skewness | | -1.657 | .304 |
|  |  | Kurtosis | | 1.752 | .599 |
| On the scale shown, how much do you agree the following actions would help address the problem of antibiotic resistance? [Parents should make sure all of their children’s vaccinations are up-to-date] | Up to Eur24999 | Mean | | 4.78 | .078 |
|  |  | 95% Confidence Interval for Mean | Lower Bound | 4.62 |  |
|  |  |  | Upper Bound | 4.93 |  |
|  |  | 5% Trimmed Mean | | 4.88 |  |
|  |  | Median | | 5.00 |  |
|  |  | Variance | | .352 |  |
|  |  | Std. Deviation | | .594 |  |
|  |  | Minimum | | 2 |  |
|  |  | Maximum | | 5 |  |
|  |  | Range | | 3 |  |
|  |  | Interquartile Range | | 0 |  |
|  |  | Skewness | | -3.026 | .314 |
|  |  | Kurtosis | | 9.638 | .618 |
|  | Eur25000-49999 | Mean | | 4.77 | .049 |
|  |  | 95% Confidence Interval for Mean | Lower Bound | 4.67 |  |
|  |  |  | Upper Bound | 4.86 |  |
|  |  | 5% Trimmed Mean | | 4.87 |  |
|  |  | Median | | 5.00 |  |
|  |  | Variance | | .388 |  |
|  |  | Std. Deviation | | .623 |  |
|  |  | Minimum | | 2 |  |
|  |  | Maximum | | 5 |  |
|  |  | Range | | 3 |  |
|  |  | Interquartile Range | | 0 |  |
|  |  | Skewness | | -2.882 | .190 |
|  |  | Kurtosis | | 7.926 | .377 |
|  | Eur50000-99999 | Mean | | 4.81 | .045 |
|  |  | 95% Confidence Interval for Mean | Lower Bound | 4.72 |  |
|  |  |  | Upper Bound | 4.90 |  |
|  |  | 5% Trimmed Mean | | 4.89 |  |
|  |  | Median | | 5.00 |  |
|  |  | Variance | | .253 |  |
|  |  | Std. Deviation | | .503 |  |
|  |  | Minimum | | 2 |  |
|  |  | Maximum | | 5 |  |
|  |  | Range | | 3 |  |
|  |  | Interquartile Range | | 0 |  |
|  |  | Skewness | | -3.026 | .217 |
|  |  | Kurtosis | | 10.234 | .430 |
|  | Eur100000+ | Mean | | 4.85 | .074 |
|  |  | 95% Confidence Interval for Mean | Lower Bound | 4.70 |  |
|  |  |  | Upper Bound | 5.00 |  |
|  |  | 5% Trimmed Mean | | 4.97 |  |
|  |  | Median | | 5.00 |  |
|  |  | Variance | | .371 |  |
|  |  | Std. Deviation | | .609 |  |
|  |  | Minimum | | 1 |  |
|  |  | Maximum | | 5 |  |
|  |  | Range | | 4 |  |
|  |  | Interquartile Range | | 0 |  |
|  |  | Skewness | | -4.887 | .293 |
|  |  | Kurtosis | | 26.274 | .578 |
|  | Prefer Not to Say | Mean | | 4.84 | .066 |
|  |  | 95% Confidence Interval for Mean | Lower Bound | 4.71 |  |
|  |  |  | Upper Bound | 4.97 |  |
|  |  | 5% Trimmed Mean | | 4.93 |  |
|  |  | Median | | 5.00 |  |
|  |  | Variance | | .269 |  |
|  |  | Std. Deviation | | .518 |  |
|  |  | Minimum | | 2 |  |
|  |  | Maximum | | 5 |  |
|  |  | Range | | 3 |  |
|  |  | Interquartile Range | | 0 |  |
|  |  | Skewness | | -3.870 | .304 |
|  |  | Kurtosis | | 16.617 | .599 |
| On the scale shown, how much do you agree the following actions would help address the problem of antibiotic resistance? [People should wash their hands regularly] | Up to Eur24999 | Mean | | 4.83 | .070 |
|  |  | 95% Confidence Interval for Mean | Lower Bound | 4.69 |  |
|  |  |  | Upper Bound | 4.97 |  |
|  |  | 5% Trimmed Mean | | 4.92 |  |
|  |  | Median | | 5.00 |  |
|  |  | Variance | | .286 |  |
|  |  | Std. Deviation | | .534 |  |
|  |  | Minimum | | 2 |  |
|  |  | Maximum | | 5 |  |
|  |  | Range | | 3 |  |
|  |  | Interquartile Range | | 0 |  |
|  |  | Skewness | | -3.726 | .314 |
|  |  | Kurtosis | | 15.356 | .618 |
|  | Eur25000-49999 | Mean | | 4.81 | .043 |
|  |  | 95% Confidence Interval for Mean | Lower Bound | 4.73 |  |
|  |  |  | Upper Bound | 4.90 |  |
|  |  | 5% Trimmed Mean | | 4.91 |  |
|  |  | Median | | 5.00 |  |
|  |  | Variance | | .301 |  |
|  |  | Std. Deviation | | .549 |  |
|  |  | Minimum | | 1 |  |
|  |  | Maximum | | 5 |  |
|  |  | Range | | 4 |  |
|  |  | Interquartile Range | | 0 |  |
|  |  | Skewness | | -3.678 | .190 |
|  |  | Kurtosis | | 16.703 | .377 |
|  | Eur50000-99999 | Mean | | 4.84 | .045 |
|  |  | 95% Confidence Interval for Mean | Lower Bound | 4.75 |  |
|  |  |  | Upper Bound | 4.93 |  |
|  |  | 5% Trimmed Mean | | 4.93 |  |
|  |  | Median | | 5.00 |  |
|  |  | Variance | | .248 |  |
|  |  | Std. Deviation | | .498 |  |
|  |  | Minimum | | 2 |  |
|  |  | Maximum | | 5 |  |
|  |  | Range | | 3 |  |
|  |  | Interquartile Range | | 0 |  |
|  |  | Skewness | | -3.489 | .217 |
|  |  | Kurtosis | | 12.766 | .430 |
|  | Eur100000+ | Mean | | 4.79 | .081 |
|  |  | 95% Confidence Interval for Mean | Lower Bound | 4.63 |  |
|  |  |  | Upper Bound | 4.95 |  |
|  |  | 5% Trimmed Mean | | 4.91 |  |
|  |  | Median | | 5.00 |  |
|  |  | Variance | | .441 |  |
|  |  | Std. Deviation | | .664 |  |
|  |  | Minimum | | 1 |  |
|  |  | Maximum | | 5 |  |
|  |  | Range | | 4 |  |
|  |  | Interquartile Range | | 0 |  |
|  |  | Skewness | | -3.908 | .293 |
|  |  | Kurtosis | | 17.304 | .578 |
|  | Prefer Not to Say | Mean | | 4.71 | .099 |
|  |  | 95% Confidence Interval for Mean | Lower Bound | 4.51 |  |
|  |  |  | Upper Bound | 4.91 |  |
|  |  | 5% Trimmed Mean | | 4.84 |  |
|  |  | Median | | 5.00 |  |
|  |  | Variance | | .603 |  |
|  |  | Std. Deviation | | .776 |  |
|  |  | Minimum | | 1 |  |
|  |  | Maximum | | 5 |  |
|  |  | Range | | 4 |  |
|  |  | Interquartile Range | | 0 |  |
|  |  | Skewness | | -3.129 | .304 |
|  |  | Kurtosis | | 10.313 | .599 |
| On the scale shown, how much do you agree the following actions would help address the problem of antibiotic resistance? [Doctors should only prescribe antibiotics when they are needed ] | Up to Eur24999 | Mean | | 4.74 | .094 |
|  |  | 95% Confidence Interval for Mean | Lower Bound | 4.55 |  |
|  |  |  | Upper Bound | 4.93 |  |
|  |  | 5% Trimmed Mean | | 4.86 |  |
|  |  | Median | | 5.00 |  |
|  |  | Variance | | .511 |  |
|  |  | Std. Deviation | | .715 |  |
|  |  | Minimum | | 1 |  |
|  |  | Maximum | | 5 |  |
|  |  | Range | | 4 |  |
|  |  | Interquartile Range | | 0 |  |
|  |  | Skewness | | -3.752 | .314 |
|  |  | Kurtosis | | 15.882 | .618 |
|  | Eur25000-49999 | Mean | | 4.91 | .027 |
|  |  | 95% Confidence Interval for Mean | Lower Bound | 4.86 |  |
|  |  |  | Upper Bound | 4.96 |  |
|  |  | 5% Trimmed Mean | | 4.97 |  |
|  |  | Median | | 5.00 |  |
|  |  | Variance | | .120 |  |
|  |  | Std. Deviation | | .347 |  |
|  |  | Minimum | | 3 |  |
|  |  | Maximum | | 5 |  |
|  |  | Range | | 2 |  |
|  |  | Interquartile Range | | 0 |  |
|  |  | Skewness | | -4.086 | .190 |
|  |  | Kurtosis | | 17.112 | .377 |
|  | Eur50000-99999 | Mean | | 4.94 | .026 |
|  |  | 95% Confidence Interval for Mean | Lower Bound | 4.89 |  |
|  |  |  | Upper Bound | 5.00 |  |
|  |  | 5% Trimmed Mean | | 5.00 |  |
|  |  | Median | | 5.00 |  |
|  |  | Variance | | .086 |  |
|  |  | Std. Deviation | | .292 |  |
|  |  | Minimum | | 3 |  |
|  |  | Maximum | | 5 |  |
|  |  | Range | | 2 |  |
|  |  | Interquartile Range | | 0 |  |
|  |  | Skewness | | -5.632 | .217 |
|  |  | Kurtosis | | 32.719 | .430 |
|  | Eur100000+ | Mean | | 4.94 | .036 |
|  |  | 95% Confidence Interval for Mean | Lower Bound | 4.87 |  |
|  |  |  | Upper Bound | 5.01 |  |
|  |  | 5% Trimmed Mean | | 5.00 |  |
|  |  | Median | | 5.00 |  |
|  |  | Variance | | .087 |  |
|  |  | Std. Deviation | | .295 |  |
|  |  | Minimum | | 3 |  |
|  |  | Maximum | | 5 |  |
|  |  | Range | | 2 |  |
|  |  | Interquartile Range | | 0 |  |
|  |  | Skewness | | -5.422 | .293 |
|  |  | Kurtosis | | 31.132 | .578 |
|  | Prefer Not to Say | Mean | | 4.95 | .027 |
|  |  | 95% Confidence Interval for Mean | Lower Bound | 4.90 |  |
|  |  |  | Upper Bound | 5.01 |  |
|  |  | 5% Trimmed Mean | | 5.00 |  |
|  |  | Median | | 5.00 |  |
|  |  | Variance | | .047 |  |
|  |  | Std. Deviation | | .216 |  |
|  |  | Minimum | | 4 |  |
|  |  | Maximum | | 5 |  |
|  |  | Range | | 1 |  |
|  |  | Interquartile Range | | 0 |  |
|  |  | Skewness | | -4.314 | .304 |
|  |  | Kurtosis | | 17.166 | .599 |
| On the scale shown, how much do you agree the following actions would help address the problem of antibiotic resistance? [Governments should reward the development of new antibiotics] | Up to Eur24999 | Mean | | 3.95 | .133 |
|  |  | 95% Confidence Interval for Mean | Lower Bound | 3.68 |  |
|  |  |  | Upper Bound | 4.22 |  |
|  |  | 5% Trimmed Mean | | 4.02 |  |
|  |  | Median | | 4.00 |  |
|  |  | Variance | | 1.032 |  |
|  |  | Std. Deviation | | 1.016 |  |
|  |  | Minimum | | 1 |  |
|  |  | Maximum | | 5 |  |
|  |  | Range | | 4 |  |
|  |  | Interquartile Range | | 2 |  |
|  |  | Skewness | | -.621 | .314 |
|  |  | Kurtosis | | -.263 | .618 |
|  | Eur25000-49999 | Mean | | 3.98 | .088 |
|  |  | 95% Confidence Interval for Mean | Lower Bound | 3.80 |  |
|  |  |  | Upper Bound | 4.15 |  |
|  |  | 5% Trimmed Mean | | 4.08 |  |
|  |  | Median | | 4.00 |  |
|  |  | Variance | | 1.263 |  |
|  |  | Std. Deviation | | 1.124 |  |
|  |  | Minimum | | 1 |  |
|  |  | Maximum | | 5 |  |
|  |  | Range | | 4 |  |
|  |  | Interquartile Range | | 2 |  |
|  |  | Skewness | | -.870 | .190 |
|  |  | Kurtosis | | .031 | .377 |
|  | Eur50000-99999 | Mean | | 4.04 | .104 |
|  |  | 95% Confidence Interval for Mean | Lower Bound | 3.83 |  |
|  |  |  | Upper Bound | 4.25 |  |
|  |  | 5% Trimmed Mean | | 4.15 |  |
|  |  | Median | | 5.00 |  |
|  |  | Variance | | 1.345 |  |
|  |  | Std. Deviation | | 1.160 |  |
|  |  | Minimum | | 1 |  |
|  |  | Maximum | | 5 |  |
|  |  | Range | | 4 |  |
|  |  | Interquartile Range | | 2 |  |
|  |  | Skewness | | -.993 | .217 |
|  |  | Kurtosis | | .143 | .430 |
|  | Eur100000+ | Mean | | 4.00 | .115 |
|  |  | 95% Confidence Interval for Mean | Lower Bound | 3.77 |  |
|  |  |  | Upper Bound | 4.23 |  |
|  |  | 5% Trimmed Mean | | 4.03 |  |
|  |  | Median | | 4.00 |  |
|  |  | Variance | | .879 |  |
|  |  | Std. Deviation | | .937 |  |
|  |  | Minimum | | 1 |  |
|  |  | Maximum | | 5 |  |
|  |  | Range | | 4 |  |
|  |  | Interquartile Range | | 2 |  |
|  |  | Skewness | | -.455 | .293 |
|  |  | Kurtosis | | -.348 | .578 |
|  | Prefer Not to Say | Mean | | 4.11 | .132 |
|  |  | 95% Confidence Interval for Mean | Lower Bound | 3.85 |  |
|  |  |  | Upper Bound | 4.38 |  |
|  |  | 5% Trimmed Mean | | 4.23 |  |
|  |  | Median | | 4.00 |  |
|  |  | Variance | | 1.085 |  |
|  |  | Std. Deviation | | 1.042 |  |
|  |  | Minimum | | 1 |  |
|  |  | Maximum | | 5 |  |
|  |  | Range | | 4 |  |
|  |  | Interquartile Range | | 1 |  |
|  |  | Skewness | | -1.491 | .304 |
|  |  | Kurtosis | | 2.172 | .599 |
| On the scale shown, how much do you agree the following actions would help address the problem of antibiotic resistance? [Pharmaceutical companies should develop new antibiotics] | Up to Eur24999 | Mean | | 4.03 | .125 |
|  |  | 95% Confidence Interval for Mean | Lower Bound | 3.78 |  |
|  |  |  | Upper Bound | 4.29 |  |
|  |  | 5% Trimmed Mean | | 4.10 |  |
|  |  | Median | | 4.00 |  |
|  |  | Variance | | .911 |  |
|  |  | Std. Deviation | | .955 |  |
|  |  | Minimum | | 1 |  |
|  |  | Maximum | | 5 |  |
|  |  | Range | | 4 |  |
|  |  | Interquartile Range | | 2 |  |
|  |  | Skewness | | -.697 | .314 |
|  |  | Kurtosis | | .128 | .618 |
|  | Eur25000-49999 | Mean | | 3.98 | .079 |
|  |  | 95% Confidence Interval for Mean | Lower Bound | 3.83 |  |
|  |  |  | Upper Bound | 4.14 |  |
|  |  | 5% Trimmed Mean | | 4.07 |  |
|  |  | Median | | 4.00 |  |
|  |  | Variance | | 1.024 |  |
|  |  | Std. Deviation | | 1.012 |  |
|  |  | Minimum | | 1 |  |
|  |  | Maximum | | 5 |  |
|  |  | Range | | 4 |  |
|  |  | Interquartile Range | | 2 |  |
|  |  | Skewness | | -.790 | .190 |
|  |  | Kurtosis | | .300 | .377 |
|  | Eur50000-99999 | Mean | | 4.07 | .092 |
|  |  | 95% Confidence Interval for Mean | Lower Bound | 3.89 |  |
|  |  |  | Upper Bound | 4.25 |  |
|  |  | 5% Trimmed Mean | | 4.15 |  |
|  |  | Median | | 4.00 |  |
|  |  | Variance | | 1.067 |  |
|  |  | Std. Deviation | | 1.033 |  |
|  |  | Minimum | | 1 |  |
|  |  | Maximum | | 5 |  |
|  |  | Range | | 4 |  |
|  |  | Interquartile Range | | 2 |  |
|  |  | Skewness | | -.904 | .217 |
|  |  | Kurtosis | | .016 | .430 |
|  | Eur100000+ | Mean | | 4.06 | .122 |
|  |  | 95% Confidence Interval for Mean | Lower Bound | 3.82 |  |
|  |  |  | Upper Bound | 4.30 |  |
|  |  | 5% Trimmed Mean | | 4.14 |  |
|  |  | Median | | 4.00 |  |
|  |  | Variance | | .996 |  |
|  |  | Std. Deviation | | .998 |  |
|  |  | Minimum | | 1 |  |
|  |  | Maximum | | 5 |  |
|  |  | Range | | 4 |  |
|  |  | Interquartile Range | | 2 |  |
|  |  | Skewness | | -.782 | .293 |
|  |  | Kurtosis | | -.058 | .578 |
|  | Prefer Not to Say | Mean | | 4.02 | .141 |
|  |  | 95% Confidence Interval for Mean | Lower Bound | 3.73 |  |
|  |  |  | Upper Bound | 4.30 |  |
|  |  | 5% Trimmed Mean | | 4.13 |  |
|  |  | Median | | 4.00 |  |
|  |  | Variance | | 1.229 |  |
|  |  | Std. Deviation | | 1.109 |  |
|  |  | Minimum | | 1 |  |
|  |  | Maximum | | 5 |  |
|  |  | Range | | 4 |  |
|  |  | Interquartile Range | | 2 |  |
|  |  | Skewness | | -1.077 | .304 |
|  |  | Kurtosis | | .683 | .599 |
| On the scale shown, how much do you agree with the following statements? [Antibiotic resistance is one of the biggest problems the world faces] | Up to Eur24999 | Mean | | 3.69 | .146 |
|  |  | 95% Confidence Interval for Mean | Lower Bound | 3.40 |  |
|  |  |  | Upper Bound | 3.98 |  |
|  |  | 5% Trimmed Mean | | 3.75 |  |
|  |  | Median | | 4.00 |  |
|  |  | Variance | | 1.235 |  |
|  |  | Std. Deviation | | 1.111 |  |
|  |  | Minimum | | 1 |  |
|  |  | Maximum | | 5 |  |
|  |  | Range | | 4 |  |
|  |  | Interquartile Range | | 2 |  |
|  |  | Skewness | | -.380 | .314 |
|  |  | Kurtosis | | -.570 | .618 |
|  | Eur25000-49999 | Mean | | 3.72 | .084 |
|  |  | 95% Confidence Interval for Mean | Lower Bound | 3.55 |  |
|  |  |  | Upper Bound | 3.88 |  |
|  |  | 5% Trimmed Mean | | 3.76 |  |
|  |  | Median | | 4.00 |  |
|  |  | Variance | | 1.148 |  |
|  |  | Std. Deviation | | 1.071 |  |
|  |  | Minimum | | 1 |  |
|  |  | Maximum | | 5 |  |
|  |  | Range | | 4 |  |
|  |  | Interquartile Range | | 2 |  |
|  |  | Skewness | | -.237 | .190 |
|  |  | Kurtosis | | -.863 | .377 |
|  | Eur50000-99999 | Mean | | 3.69 | .099 |
|  |  | 95% Confidence Interval for Mean | Lower Bound | 3.49 |  |
|  |  |  | Upper Bound | 3.88 |  |
|  |  | 5% Trimmed Mean | | 3.75 |  |
|  |  | Median | | 4.00 |  |
|  |  | Variance | | 1.216 |  |
|  |  | Std. Deviation | | 1.103 |  |
|  |  | Minimum | | 1 |  |
|  |  | Maximum | | 5 |  |
|  |  | Range | | 4 |  |
|  |  | Interquartile Range | | 2 |  |
|  |  | Skewness | | -.562 | .217 |
|  |  | Kurtosis | | -.357 | .430 |
|  | Eur100000+ | Mean | | 3.30 | .144 |
|  |  | 95% Confidence Interval for Mean | Lower Bound | 3.01 |  |
|  |  |  | Upper Bound | 3.59 |  |
|  |  | 5% Trimmed Mean | | 3.33 |  |
|  |  | Median | | 3.00 |  |
|  |  | Variance | | 1.394 |  |
|  |  | Std. Deviation | | 1.181 |  |
|  |  | Minimum | | 1 |  |
|  |  | Maximum | | 5 |  |
|  |  | Range | | 4 |  |
|  |  | Interquartile Range | | 1 |  |
|  |  | Skewness | | -.097 | .293 |
|  |  | Kurtosis | | -.680 | .578 |
|  | Prefer Not to Say | Mean | | 3.65 | .142 |
|  |  | 95% Confidence Interval for Mean | Lower Bound | 3.36 |  |
|  |  |  | Upper Bound | 3.93 |  |
|  |  | 5% Trimmed Mean | | 3.72 |  |
|  |  | Median | | 4.00 |  |
|  |  | Variance | | 1.249 |  |
|  |  | Std. Deviation | | 1.118 |  |
|  |  | Minimum | | 1 |  |
|  |  | Maximum | | 5 |  |
|  |  | Range | | 4 |  |
|  |  | Interquartile Range | | 2 |  |
|  |  | Skewness | | -.560 | .304 |
|  |  | Kurtosis | | -.278 | .599 |
| On the scale shown, how much do you agree with the following statements? [Medical experts will solve the problem of antibiotic resistance before it becomes too serious] | Up to Eur24999 | Mean | | 3.40 | .132 |
|  |  | 95% Confidence Interval for Mean | Lower Bound | 3.13 |  |
|  |  |  | Upper Bound | 3.66 |  |
|  |  | 5% Trimmed Mean | | 3.42 |  |
|  |  | Median | | 3.00 |  |
|  |  | Variance | | 1.015 |  |
|  |  | Std. Deviation | | 1.008 |  |
|  |  | Minimum | | 1 |  |
|  |  | Maximum | | 5 |  |
|  |  | Range | | 4 |  |
|  |  | Interquartile Range | | 1 |  |
|  |  | Skewness | | -.026 | .314 |
|  |  | Kurtosis | | -.191 | .618 |
|  | Eur25000-49999 | Mean | | 2.79 | .086 |
|  |  | 95% Confidence Interval for Mean | Lower Bound | 2.62 |  |
|  |  |  | Upper Bound | 2.96 |  |
|  |  | 5% Trimmed Mean | | 2.76 |  |
|  |  | Median | | 3.00 |  |
|  |  | Variance | | 1.212 |  |
|  |  | Std. Deviation | | 1.101 |  |
|  |  | Minimum | | 1 |  |
|  |  | Maximum | | 5 |  |
|  |  | Range | | 4 |  |
|  |  | Interquartile Range | | 1 |  |
|  |  | Skewness | | .238 | .190 |
|  |  | Kurtosis | | -.290 | .377 |
|  | Eur50000-99999 | Mean | | 2.90 | .092 |
|  |  | 95% Confidence Interval for Mean | Lower Bound | 2.71 |  |
|  |  |  | Upper Bound | 3.08 |  |
|  |  | 5% Trimmed Mean | | 2.88 |  |
|  |  | Median | | 3.00 |  |
|  |  | Variance | | 1.062 |  |
|  |  | Std. Deviation | | 1.030 |  |
|  |  | Minimum | | 1 |  |
|  |  | Maximum | | 5 |  |
|  |  | Range | | 4 |  |
|  |  | Interquartile Range | | 1 |  |
|  |  | Skewness | | .121 | .217 |
|  |  | Kurtosis | | -.106 | .430 |
|  | Eur100000+ | Mean | | 2.97 | .114 |
|  |  | 95% Confidence Interval for Mean | Lower Bound | 2.74 |  |
|  |  |  | Upper Bound | 3.20 |  |
|  |  | 5% Trimmed Mean | | 2.97 |  |
|  |  | Median | | 3.00 |  |
|  |  | Variance | | .878 |  |
|  |  | Std. Deviation | | .937 |  |
|  |  | Minimum | | 1 |  |
|  |  | Maximum | | 5 |  |
|  |  | Range | | 4 |  |
|  |  | Interquartile Range | | 2 |  |
|  |  | Skewness | | -.167 | .293 |
|  |  | Kurtosis | | .145 | .578 |
|  | Prefer Not to Say | Mean | | 2.98 | .135 |
|  |  | 95% Confidence Interval for Mean | Lower Bound | 2.71 |  |
|  |  |  | Upper Bound | 3.25 |  |
|  |  | 5% Trimmed Mean | | 2.98 |  |
|  |  | Median | | 3.00 |  |
|  |  | Variance | | 1.131 |  |
|  |  | Std. Deviation | | 1.063 |  |
|  |  | Minimum | | 1 |  |
|  |  | Maximum | | 5 |  |
|  |  | Range | | 4 |  |
|  |  | Interquartile Range | | 2 |  |
|  |  | Skewness | | .033 | .304 |
|  |  | Kurtosis | | -.140 | .599 |
| On the scale shown, how much do you agree with the following statements? [Everyone needs to take responsibility for using antibiotics responsibly] | Up to Eur24999 | Mean | | 4.81 | .062 |
|  |  | 95% Confidence Interval for Mean | Lower Bound | 4.69 |  |
|  |  |  | Upper Bound | 4.94 |  |
|  |  | 5% Trimmed Mean | | 4.88 |  |
|  |  | Median | | 5.00 |  |
|  |  | Variance | | .227 |  |
|  |  | Std. Deviation | | .476 |  |
|  |  | Minimum | | 3 |  |
|  |  | Maximum | | 5 |  |
|  |  | Range | | 2 |  |
|  |  | Interquartile Range | | 0 |  |
|  |  | Skewness | | -2.571 | .314 |
|  |  | Kurtosis | | 6.163 | .618 |
|  | Eur25000-49999 | Mean | | 4.86 | .032 |
|  |  | 95% Confidence Interval for Mean | Lower Bound | 4.80 |  |
|  |  |  | Upper Bound | 4.92 |  |
|  |  | 5% Trimmed Mean | | 4.93 |  |
|  |  | Median | | 5.00 |  |
|  |  | Variance | | .170 |  |
|  |  | Std. Deviation | | .413 |  |
|  |  | Minimum | | 3 |  |
|  |  | Maximum | | 5 |  |
|  |  | Range | | 2 |  |
|  |  | Interquartile Range | | 0 |  |
|  |  | Skewness | | -3.078 | .190 |
|  |  | Kurtosis | | 9.243 | .377 |
|  | Eur50000-99999 | Mean | | 4.78 | .051 |
|  |  | 95% Confidence Interval for Mean | Lower Bound | 4.68 |  |
|  |  |  | Upper Bound | 4.88 |  |
|  |  | 5% Trimmed Mean | | 4.87 |  |
|  |  | Median | | 5.00 |  |
|  |  | Variance | | .320 |  |
|  |  | Std. Deviation | | .566 |  |
|  |  | Minimum | | 2 |  |
|  |  | Maximum | | 5 |  |
|  |  | Range | | 3 |  |
|  |  | Interquartile Range | | 0 |  |
|  |  | Skewness | | -2.978 | .217 |
|  |  | Kurtosis | | 9.681 | .430 |
|  | Eur100000+ | Mean | | 4.75 | .065 |
|  |  | 95% Confidence Interval for Mean | Lower Bound | 4.62 |  |
|  |  |  | Upper Bound | 4.88 |  |
|  |  | 5% Trimmed Mean | | 4.82 |  |
|  |  | Median | | 5.00 |  |
|  |  | Variance | | .283 |  |
|  |  | Std. Deviation | | .532 |  |
|  |  | Minimum | | 3 |  |
|  |  | Maximum | | 5 |  |
|  |  | Range | | 2 |  |
|  |  | Interquartile Range | | 0 |  |
|  |  | Skewness | | -2.040 | .293 |
|  |  | Kurtosis | | 3.385 | .578 |
|  | Prefer Not to Say | Mean | | 4.77 | .071 |
|  |  | 95% Confidence Interval for Mean | Lower Bound | 4.63 |  |
|  |  |  | Upper Bound | 4.92 |  |
|  |  | 5% Trimmed Mean | | 4.86 |  |
|  |  | Median | | 5.00 |  |
|  |  | Variance | | .309 |  |
|  |  | Std. Deviation | | .556 |  |
|  |  | Minimum | | 2 |  |
|  |  | Maximum | | 5 |  |
|  |  | Range | | 3 |  |
|  |  | Interquartile Range | | 0 |  |
|  |  | Skewness | | -3.013 | .304 |
|  |  | Kurtosis | | 10.597 | .599 |
| On the scale shown, how much do you agree with the following statements? [There is not much people like me can do to stop antibiotic resistance] | Up to Eur24999 | Mean | | 3.22 | .133 |
|  |  | 95% Confidence Interval for Mean | Lower Bound | 2.96 |  |
|  |  |  | Upper Bound | 3.49 |  |
|  |  | 5% Trimmed Mean | | 3.23 |  |
|  |  | Median | | 3.00 |  |
|  |  | Variance | | 1.019 |  |
|  |  | Std. Deviation | | 1.009 |  |
|  |  | Minimum | | 1 |  |
|  |  | Maximum | | 5 |  |
|  |  | Range | | 4 |  |
|  |  | Interquartile Range | | 1 |  |
|  |  | Skewness | | -.048 | .314 |
|  |  | Kurtosis | | -.504 | .618 |
|  | Eur25000-49999 | Mean | | 2.71 | .097 |
|  |  | 95% Confidence Interval for Mean | Lower Bound | 2.52 |  |
|  |  |  | Upper Bound | 2.91 |  |
|  |  | 5% Trimmed Mean | | 2.68 |  |
|  |  | Median | | 3.00 |  |
|  |  | Variance | | 1.555 |  |
|  |  | Std. Deviation | | 1.247 |  |
|  |  | Minimum | | 1 |  |
|  |  | Maximum | | 5 |  |
|  |  | Range | | 4 |  |
|  |  | Interquartile Range | | 2 |  |
|  |  | Skewness | | .118 | .190 |
|  |  | Kurtosis | | -1.027 | .377 |
|  | Eur50000-99999 | Mean | | 2.77 | .108 |
|  |  | 95% Confidence Interval for Mean | Lower Bound | 2.55 |  |
|  |  |  | Upper Bound | 2.98 |  |
|  |  | 5% Trimmed Mean | | 2.74 |  |
|  |  | Median | | 3.00 |  |
|  |  | Variance | | 1.454 |  |
|  |  | Std. Deviation | | 1.206 |  |
|  |  | Minimum | | 1 |  |
|  |  | Maximum | | 5 |  |
|  |  | Range | | 4 |  |
|  |  | Interquartile Range | | 2 |  |
|  |  | Skewness | | .094 | .217 |
|  |  | Kurtosis | | -.995 | .430 |
|  | Eur100000+ | Mean | | 2.91 | .151 |
|  |  | 95% Confidence Interval for Mean | Lower Bound | 2.61 |  |
|  |  |  | Upper Bound | 3.21 |  |
|  |  | 5% Trimmed Mean | | 2.90 |  |
|  |  | Median | | 3.00 |  |
|  |  | Variance | | 1.537 |  |
|  |  | Std. Deviation | | 1.240 |  |
|  |  | Minimum | | 1 |  |
|  |  | Maximum | | 5 |  |
|  |  | Range | | 4 |  |
|  |  | Interquartile Range | | 2 |  |
|  |  | Skewness | | -.219 | .293 |
|  |  | Kurtosis | | -1.010 | .578 |
|  | Prefer Not to Say | Mean | | 2.97 | .169 |
|  |  | 95% Confidence Interval for Mean | Lower Bound | 2.63 |  |
|  |  |  | Upper Bound | 3.31 |  |
|  |  | 5% Trimmed Mean | | 2.96 |  |
|  |  | Median | | 3.00 |  |
|  |  | Variance | | 1.769 |  |
|  |  | Std. Deviation | | 1.330 |  |
|  |  | Minimum | | 1 |  |
|  |  | Maximum | | 5 |  |
|  |  | Range | | 4 |  |
|  |  | Interquartile Range | | 2 |  |
|  |  | Skewness | | .018 | .304 |
|  |  | Kurtosis | | -1.076 | .599 |
| On the scale shown, how much do you agree with the following statements? [I am worried about the impact that antibiotic resistance will have on my health, and that of my family] | Up to Eur24999 | Mean | | 3.79 | .145 |
|  |  | 95% Confidence Interval for Mean | Lower Bound | 3.50 |  |
|  |  |  | Upper Bound | 4.08 |  |
|  |  | 5% Trimmed Mean | | 3.86 |  |
|  |  | Median | | 4.00 |  |
|  |  | Variance | | 1.220 |  |
|  |  | Std. Deviation | | 1.104 |  |
|  |  | Minimum | | 1 |  |
|  |  | Maximum | | 5 |  |
|  |  | Range | | 4 |  |
|  |  | Interquartile Range | | 2 |  |
|  |  | Skewness | | -.706 | .314 |
|  |  | Kurtosis | | -.193 | .618 |
|  | Eur25000-49999 | Mean | | 4.05 | .075 |
|  |  | 95% Confidence Interval for Mean | Lower Bound | 3.90 |  |
|  |  |  | Upper Bound | 4.20 |  |
|  |  | 5% Trimmed Mean | | 4.12 |  |
|  |  | Median | | 4.00 |  |
|  |  | Variance | | .918 |  |
|  |  | Std. Deviation | | .958 |  |
|  |  | Minimum | | 1 |  |
|  |  | Maximum | | 5 |  |
|  |  | Range | | 4 |  |
|  |  | Interquartile Range | | 2 |  |
|  |  | Skewness | | -.734 | .190 |
|  |  | Kurtosis | | -.063 | .377 |
|  | Eur50000-99999 | Mean | | 3.83 | .097 |
|  |  | 95% Confidence Interval for Mean | Lower Bound | 3.64 |  |
|  |  |  | Upper Bound | 4.02 |  |
|  |  | 5% Trimmed Mean | | 3.91 |  |
|  |  | Median | | 4.00 |  |
|  |  | Variance | | 1.173 |  |
|  |  | Std. Deviation | | 1.083 |  |
|  |  | Minimum | | 1 |  |
|  |  | Maximum | | 5 |  |
|  |  | Range | | 4 |  |
|  |  | Interquartile Range | | 2 |  |
|  |  | Skewness | | -.858 | .217 |
|  |  | Kurtosis | | .166 | .430 |
|  | Eur100000+ | Mean | | 3.66 | .134 |
|  |  | 95% Confidence Interval for Mean | Lower Bound | 3.39 |  |
|  |  |  | Upper Bound | 3.92 |  |
|  |  | 5% Trimmed Mean | | 3.71 |  |
|  |  | Median | | 4.00 |  |
|  |  | Variance | | 1.199 |  |
|  |  | Std. Deviation | | 1.095 |  |
|  |  | Minimum | | 1 |  |
|  |  | Maximum | | 5 |  |
|  |  | Range | | 4 |  |
|  |  | Interquartile Range | | 2 |  |
|  |  | Skewness | | -.343 | .293 |
|  |  | Kurtosis | | -.617 | .578 |
|  | Prefer Not to Say | Mean | | 3.85 | .143 |
|  |  | 95% Confidence Interval for Mean | Lower Bound | 3.57 |  |
|  |  |  | Upper Bound | 4.14 |  |
|  |  | 5% Trimmed Mean | | 3.95 |  |
|  |  | Median | | 4.00 |  |
|  |  | Variance | | 1.274 |  |
|  |  | Std. Deviation | | 1.129 |  |
|  |  | Minimum | | 1 |  |
|  |  | Maximum | | 5 |  |
|  |  | Range | | 4 |  |
|  |  | Interquartile Range | | 2 |  |
|  |  | Skewness | | -.978 | .304 |
|  |  | Kurtosis | | .559 | .599 |
| On the scale shown, how much do you agree with the following statements? [I am not at risk of getting an antibiotic-resistant infection, as long as I take my antibiotics correctly] | Up to Eur24999 | Mean | | 3.36 | .149 |
|  |  | 95% Confidence Interval for Mean | Lower Bound | 3.06 |  |
|  |  |  | Upper Bound | 3.66 |  |
|  |  | 5% Trimmed Mean | | 3.40 |  |
|  |  | Median | | 3.00 |  |
|  |  | Variance | | 1.288 |  |
|  |  | Std. Deviation | | 1.135 |  |
|  |  | Minimum | | 1 |  |
|  |  | Maximum | | 5 |  |
|  |  | Range | | 4 |  |
|  |  | Interquartile Range | | 1 |  |
|  |  | Skewness | | -.391 | .314 |
|  |  | Kurtosis | | -.251 | .618 |
|  | Eur25000-49999 | Mean | | 2.71 | .105 |
|  |  | 95% Confidence Interval for Mean | Lower Bound | 2.50 |  |
|  |  |  | Upper Bound | 2.91 |  |
|  |  | 5% Trimmed Mean | | 2.67 |  |
|  |  | Median | | 3.00 |  |
|  |  | Variance | | 1.803 |  |
|  |  | Std. Deviation | | 1.343 |  |
|  |  | Minimum | | 1 |  |
|  |  | Maximum | | 5 |  |
|  |  | Range | | 4 |  |
|  |  | Interquartile Range | | 3 |  |
|  |  | Skewness | | .242 | .190 |
|  |  | Kurtosis | | -1.079 | .377 |
|  | Eur50000-99999 | Mean | | 2.68 | .121 |
|  |  | 95% Confidence Interval for Mean | Lower Bound | 2.44 |  |
|  |  |  | Upper Bound | 2.92 |  |
|  |  | 5% Trimmed Mean | | 2.64 |  |
|  |  | Median | | 3.00 |  |
|  |  | Variance | | 1.832 |  |
|  |  | Std. Deviation | | 1.354 |  |
|  |  | Minimum | | 1 |  |
|  |  | Maximum | | 5 |  |
|  |  | Range | | 4 |  |
|  |  | Interquartile Range | | 3 |  |
|  |  | Skewness | | .206 | .217 |
|  |  | Kurtosis | | -1.171 | .430 |
|  | Eur100000+ | Mean | | 2.76 | .147 |
|  |  | 95% Confidence Interval for Mean | Lower Bound | 2.47 |  |
|  |  |  | Upper Bound | 3.06 |  |
|  |  | 5% Trimmed Mean | | 2.73 |  |
|  |  | Median | | 3.00 |  |
|  |  | Variance | | 1.457 |  |
|  |  | Std. Deviation | | 1.207 |  |
|  |  | Minimum | | 1 |  |
|  |  | Maximum | | 5 |  |
|  |  | Range | | 4 |  |
|  |  | Interquartile Range | | 2 |  |
|  |  | Skewness | | .265 | .293 |
|  |  | Kurtosis | | -.708 | .578 |
|  | Prefer Not to Say | Mean | | 2.87 | .183 |
|  |  | 95% Confidence Interval for Mean | Lower Bound | 2.50 |  |
|  |  |  | Upper Bound | 3.24 |  |
|  |  | 5% Trimmed Mean | | 2.86 |  |
|  |  | Median | | 3.00 |  |
|  |  | Variance | | 2.081 |  |
|  |  | Std. Deviation | | 1.443 |  |
|  |  | Minimum | | 1 |  |
|  |  | Maximum | | 5 |  |
|  |  | Range | | 4 |  |
|  |  | Interquartile Range | | 3 |  |
|  |  | Skewness | | -.004 | .304 |
|  |  | Kurtosis | | -1.421 | .599 |

| **Tests of Normality** | | | | | | | |
| --- | --- | --- | --- | --- | --- | --- | --- |
|  | HouseholdIncome | Kolmogorov-Smirnov^a^ | | | Shapiro-Wilk | | |
|  |  | Statistic | df | Sig. | Statistic | df | Sig. |
| On the scale shown, how much do you agree the following actions would help address the problem of antibiotic resistance? [People should use antibiotics only when they are prescribed by a doctor or nurse] | Up to Eur24999 | .474 | 58 | <.001 | .479 | 58 | <.001 |
|  | Eur25000-49999 | .521 | 164 | <.001 | .352 | 164 | <.001 |
|  | Eur50000-99999 | .513 | 125 | <.001 | .309 | 125 | <.001 |
|  | Eur100000+ | .521 | 67 | <.001 | .316 | 67 | <.001 |
|  | Prefer Not to Say | .531 | 62 | <.001 | .337 | 62 | <.001 |
| On the scale shown, how much do you agree the following actions would help address the problem of antibiotic resistance? [Farmers should give fewer antibiotics to food-producing animals] | Up to Eur24999 | .315 | 58 | <.001 | .769 | 58 | <.001 |
|  | Eur25000-49999 | .384 | 164 | <.001 | .680 | 164 | <.001 |
|  | Eur50000-99999 | .394 | 125 | <.001 | .660 | 125 | <.001 |
|  | Eur100000+ | .436 | 67 | <.001 | .590 | 67 | <.001 |
|  | Prefer Not to Say | .375 | 62 | <.001 | .656 | 62 | <.001 |
| On the scale shown, how much do you agree the following actions would help address the problem of antibiotic resistance? [People should not keep antibiotics and use them later for other illnesses] | Up to Eur24999 | .341 | 58 | <.001 | .715 | 58 | <.001 |
|  | Eur25000-49999 | .419 | 164 | <.001 | .634 | 164 | <.001 |
|  | Eur50000-99999 | .414 | 125 | <.001 | .594 | 125 | <.001 |
|  | Eur100000+ | .381 | 67 | <.001 | .653 | 67 | <.001 |
|  | Prefer Not to Say | .412 | 62 | <.001 | .631 | 62 | <.001 |
| On the scale shown, how much do you agree the following actions would help address the problem of antibiotic resistance? [Parents should make sure all of their children’s vaccinations are up-to-date] | Up to Eur24999 | .492 | 58 | <.001 | .437 | 58 | <.001 |
|  | Eur25000-49999 | .499 | 164 | <.001 | .424 | 164 | <.001 |
|  | Eur50000-99999 | .497 | 125 | <.001 | .433 | 125 | <.001 |
|  | Eur100000+ | .522 | 67 | <.001 | .269 | 67 | <.001 |
|  | Prefer Not to Say | .509 | 62 | <.001 | .355 | 62 | <.001 |
| On the scale shown, how much do you agree the following actions would help address the problem of antibiotic resistance? [People should wash their hands regularly] | Up to Eur24999 | .506 | 58 | <.001 | .370 | 58 | <.001 |
|  | Eur25000-49999 | .501 | 164 | <.001 | .391 | 164 | <.001 |
|  | Eur50000-99999 | .514 | 125 | <.001 | .364 | 125 | <.001 |
|  | Eur100000+ | .504 | 67 | <.001 | .361 | 67 | <.001 |
|  | Prefer Not to Say | .484 | 62 | <.001 | .437 | 62 | <.001 |
| On the scale shown, how much do you agree the following actions would help address the problem of antibiotic resistance? [Doctors should only prescribe antibiotics when they are needed ] | Up to Eur24999 | .469 | 58 | <.001 | .410 | 58 | <.001 |
|  | Eur25000-49999 | .531 | 164 | <.001 | .283 | 164 | <.001 |
|  | Eur50000-99999 | .536 | 125 | <.001 | .190 | 125 | <.001 |
|  | Eur100000+ | .535 | 67 | <.001 | .208 | 67 | <.001 |
|  | Prefer Not to Say | .540 | 62 | <.001 | .222 | 62 | <.001 |
| On the scale shown, how much do you agree the following actions would help address the problem of antibiotic resistance? [Governments should reward the development of new antibiotics] | Up to Eur24999 | .229 | 58 | <.001 | .845 | 58 | <.001 |
|  | Eur25000-49999 | .264 | 164 | <.001 | .811 | 164 | <.001 |
|  | Eur50000-99999 | .300 | 125 | <.001 | .779 | 125 | <.001 |
|  | Eur100000+ | .245 | 67 | <.001 | .803 | 67 | <.001 |
|  | Prefer Not to Say | .279 | 62 | <.001 | .762 | 62 | <.001 |
| On the scale shown, how much do you agree the following actions would help address the problem of antibiotic resistance? [Pharmaceutical companies should develop new antibiotics] | Up to Eur24999 | .241 | 58 | <.001 | .825 | 58 | <.001 |
|  | Eur25000-49999 | .233 | 164 | <.001 | .824 | 164 | <.001 |
|  | Eur50000-99999 | .263 | 125 | <.001 | .813 | 125 | <.001 |
|  | Eur100000+ | .260 | 67 | <.001 | .821 | 67 | <.001 |
|  | Prefer Not to Say | .248 | 62 | <.001 | .806 | 62 | <.001 |
| On the scale shown, how much do you agree with the following statements? [Antibiotic resistance is one of the biggest problems the world faces] | Up to Eur24999 | .198 | 58 | <.001 | .872 | 58 | <.001 |
|  | Eur25000-49999 | .225 | 164 | <.001 | .859 | 164 | <.001 |
|  | Eur50000-99999 | .211 | 125 | <.001 | .881 | 125 | <.001 |
|  | Eur100000+ | .212 | 67 | <.001 | .898 | 67 | <.001 |
|  | Prefer Not to Say | .205 | 62 | <.001 | .886 | 62 | <.001 |
| On the scale shown, how much do you agree with the following statements? [Medical experts will solve the problem of antibiotic resistance before it becomes too serious] | Up to Eur24999 | .256 | 58 | <.001 | .883 | 58 | <.001 |
|  | Eur25000-49999 | .222 | 164 | <.001 | .897 | 164 | <.001 |
|  | Eur50000-99999 | .236 | 125 | <.001 | .897 | 125 | <.001 |
|  | Eur100000+ | .259 | 67 | <.001 | .889 | 67 | <.001 |
|  | Prefer Not to Say | .236 | 62 | <.001 | .898 | 62 | <.001 |
| On the scale shown, how much do you agree with the following statements? [Everyone needs to take responsibility for using antibiotics responsibly] | Up to Eur24999 | .500 | 58 | <.001 | .448 | 58 | <.001 |
|  | Eur25000-49999 | .517 | 164 | <.001 | .377 | 164 | <.001 |
|  | Eur50000-99999 | .486 | 125 | <.001 | .450 | 125 | <.001 |
|  | Eur100000+ | .474 | 67 | <.001 | .524 | 67 | <.001 |
|  | Prefer Not to Say | .480 | 62 | <.001 | .462 | 62 | <.001 |
| On the scale shown, how much do you agree with the following statements? [There is not much people like me can do to stop antibiotic resistance] | Up to Eur24999 | .191 | 58 | <.001 | .910 | 58 | <.001 |
|  | Eur25000-49999 | .155 | 164 | <.001 | .903 | 164 | <.001 |
|  | Eur50000-99999 | .178 | 125 | <.001 | .907 | 125 | <.001 |
|  | Eur100000+ | .186 | 67 | <.001 | .892 | 67 | <.001 |
|  | Prefer Not to Say | .139 | 62 | .005 | .905 | 62 | <.001 |
| On the scale shown, how much do you agree with the following statements? [I am worried about the impact that antibiotic resistance will have on my health, and that of my family] | Up to Eur24999 | .229 | 58 | <.001 | .867 | 58 | <.001 |
|  | Eur25000-49999 | .242 | 164 | <.001 | .830 | 164 | <.001 |
|  | Eur50000-99999 | .258 | 125 | <.001 | .851 | 125 | <.001 |
|  | Eur100000+ | .188 | 67 | <.001 | .883 | 67 | <.001 |
|  | Prefer Not to Say | .229 | 62 | <.001 | .835 | 62 | <.001 |
| On the scale shown, how much do you agree with the following statements? [I am not at risk of getting an antibiotic-resistant infection, as long as I take my antibiotics correctly] | Up to Eur24999 | .202 | 58 | <.001 | .895 | 58 | <.001 |
|  | Eur25000-49999 | .158 | 164 | <.001 | .891 | 164 | <.001 |
|  | Eur50000-99999 | .165 | 125 | <.001 | .886 | 125 | <.001 |
|  | Eur100000+ | .169 | 67 | <.001 | .909 | 67 | <.001 |
|  | Prefer Not to Say | .202 | 62 | <.001 | .871 | 62 | <.001 |
| a. Lilliefors Significance Correction | | | | | | | |

(5) REGION AND LIKERT SCALE

| **Descriptives** | | | | | |
| --- | --- | --- | --- | --- | --- |
|  | Which region of Malta do you live in? | | | Statistic | Std. Error |
| On the scale shown, how much do you agree the following actions would help address the problem of antibiotic resistance? [People should use antibiotics only when they are prescribed by a doctor or nurse] | Northern Region | Mean | | 4.88 | .042 |
|  |  | 95% Confidence Interval for Mean | Lower Bound | 4.79 |  |
|  |  |  | Upper Bound | 4.96 |  |
|  |  | 5% Trimmed Mean | | 4.94 |  |
|  |  | Median | | 5.00 |  |
|  |  | Variance | | .170 |  |
|  |  | Std. Deviation | | .413 |  |
|  |  | Minimum | | 2 |  |
|  |  | Maximum | | 5 |  |
|  |  | Range | | 3 |  |
|  |  | Interquartile Range | | 0 |  |
|  |  | Skewness | | -4.450 | .244 |
|  |  | Kurtosis | | 24.819 | .483 |
|  | Northern Harbour Region | Mean | | 4.87 | .031 |
|  |  | 95% Confidence Interval for Mean | Lower Bound | 4.80 |  |
|  |  |  | Upper Bound | 4.93 |  |
|  |  | 5% Trimmed Mean | | 4.93 |  |
|  |  | Median | | 5.00 |  |
|  |  | Variance | | .164 |  |
|  |  | Std. Deviation | | .405 |  |
|  |  | Minimum | | 3 |  |
|  |  | Maximum | | 5 |  |
|  |  | Range | | 2 |  |
|  |  | Interquartile Range | | 0 |  |
|  |  | Skewness | | -3.161 | .186 |
|  |  | Kurtosis | | 9.816 | .369 |
|  | Southern Harbour Region | Mean | | 4.69 | .110 |
|  |  | 95% Confidence Interval for Mean | Lower Bound | 4.47 |  |
|  |  |  | Upper Bound | 4.91 |  |
|  |  | 5% Trimmed Mean | | 4.79 |  |
|  |  | Median | | 5.00 |  |
|  |  | Variance | | .512 |  |
|  |  | Std. Deviation | | .715 |  |
|  |  | Minimum | | 2 |  |
|  |  | Maximum | | 5 |  |
|  |  | Range | | 3 |  |
|  |  | Interquartile Range | | 0 |  |
|  |  | Skewness | | -2.399 | .365 |
|  |  | Kurtosis | | 5.194 | .717 |
|  | South Western Region | Mean | | 4.91 | .036 |
|  |  | 95% Confidence Interval for Mean | Lower Bound | 4.84 |  |
|  |  |  | Upper Bound | 4.98 |  |
|  |  | 5% Trimmed Mean | | 4.95 |  |
|  |  | Median | | 5.00 |  |
|  |  | Variance | | .085 |  |
|  |  | Std. Deviation | | .292 |  |
|  |  | Minimum | | 4 |  |
|  |  | Maximum | | 5 |  |
|  |  | Range | | 1 |  |
|  |  | Interquartile Range | | 0 |  |
|  |  | Skewness | | -2.884 | .297 |
|  |  | Kurtosis | | 6.517 | .586 |
|  | Western Region | Mean | | 4.84 | .063 |
|  |  | 95% Confidence Interval for Mean | Lower Bound | 4.71 |  |
|  |  |  | Upper Bound | 4.96 |  |
|  |  | 5% Trimmed Mean | | 4.95 |  |
|  |  | Median | | 5.00 |  |
|  |  | Variance | | .346 |  |
|  |  | Std. Deviation | | .588 |  |
|  |  | Minimum | | 1 |  |
|  |  | Maximum | | 5 |  |
|  |  | Range | | 4 |  |
|  |  | Interquartile Range | | 0 |  |
|  |  | Skewness | | -4.521 | .258 |
|  |  | Kurtosis | | 23.318 | .511 |
|  | Gozo and Comino | Mean | | 4.92 | .077 |
|  |  | 95% Confidence Interval for Mean | Lower Bound | 4.76 |  |
|  |  |  | Upper Bound | 5.09 |  |
|  |  | 5% Trimmed Mean | | 4.97 |  |
|  |  | Median | | 5.00 |  |
|  |  | Variance | | .077 |  |
|  |  | Std. Deviation | | .277 |  |
|  |  | Minimum | | 4 |  |
|  |  | Maximum | | 5 |  |
|  |  | Range | | 1 |  |
|  |  | Interquartile Range | | 0 |  |
|  |  | Skewness | | -3.606 | .616 |
|  |  | Kurtosis | | 13.000 | 1.191 |
| On the scale shown, how much do you agree the following actions would help address the problem of antibiotic resistance? [Farmers should give fewer antibiotics to food-producing animals] | Northern Region | Mean | | 4.47 | .085 |
|  |  | 95% Confidence Interval for Mean | Lower Bound | 4.30 |  |
|  |  |  | Upper Bound | 4.64 |  |
|  |  | 5% Trimmed Mean | | 4.56 |  |
|  |  | Median | | 5.00 |  |
|  |  | Variance | | .705 |  |
|  |  | Std. Deviation | | .840 |  |
|  |  | Minimum | | 2 |  |
|  |  | Maximum | | 5 |  |
|  |  | Range | | 3 |  |
|  |  | Interquartile Range | | 1 |  |
|  |  | Skewness | | -1.394 | .244 |
|  |  | Kurtosis | | .833 | .483 |
|  | Northern Harbour Region | Mean | | 4.51 | .065 |
|  |  | 95% Confidence Interval for Mean | Lower Bound | 4.39 |  |
|  |  |  | Upper Bound | 4.64 |  |
|  |  | 5% Trimmed Mean | | 4.61 |  |
|  |  | Median | | 5.00 |  |
|  |  | Variance | | .722 |  |
|  |  | Std. Deviation | | .850 |  |
|  |  | Minimum | | 1 |  |
|  |  | Maximum | | 5 |  |
|  |  | Range | | 4 |  |
|  |  | Interquartile Range | | 1 |  |
|  |  | Skewness | | -1.793 | .186 |
|  |  | Kurtosis | | 2.903 | .369 |
|  | Southern Harbour Region | Mean | | 4.21 | .147 |
|  |  | 95% Confidence Interval for Mean | Lower Bound | 3.92 |  |
|  |  |  | Upper Bound | 4.51 |  |
|  |  | 5% Trimmed Mean | | 4.29 |  |
|  |  | Median | | 4.50 |  |
|  |  | Variance | | .904 |  |
|  |  | Std. Deviation | | .951 |  |
|  |  | Minimum | | 1 |  |
|  |  | Maximum | | 5 |  |
|  |  | Range | | 4 |  |
|  |  | Interquartile Range | | 1 |  |
|  |  | Skewness | | -1.169 | .365 |
|  |  | Kurtosis | | 1.407 | .717 |
|  | South Western Region | Mean | | 4.52 | .103 |
|  |  | 95% Confidence Interval for Mean | Lower Bound | 4.32 |  |
|  |  |  | Upper Bound | 4.73 |  |
|  |  | 5% Trimmed Mean | | 4.62 |  |
|  |  | Median | | 5.00 |  |
|  |  | Variance | | .691 |  |
|  |  | Std. Deviation | | .831 |  |
|  |  | Minimum | | 1 |  |
|  |  | Maximum | | 5 |  |
|  |  | Range | | 4 |  |
|  |  | Interquartile Range | | 1 |  |
|  |  | Skewness | | -1.928 | .297 |
|  |  | Kurtosis | | 4.054 | .586 |
|  | Western Region | Mean | | 4.21 | .115 |
|  |  | 95% Confidence Interval for Mean | Lower Bound | 3.98 |  |
|  |  |  | Upper Bound | 4.43 |  |
|  |  | 5% Trimmed Mean | | 4.32 |  |
|  |  | Median | | 5.00 |  |
|  |  | Variance | | 1.143 |  |
|  |  | Std. Deviation | | 1.069 |  |
|  |  | Minimum | | 1 |  |
|  |  | Maximum | | 5 |  |
|  |  | Range | | 4 |  |
|  |  | Interquartile Range | | 2 |  |
|  |  | Skewness | | -1.245 | .258 |
|  |  | Kurtosis | | .925 | .511 |
|  | Gozo and Comino | Mean | | 4.31 | .237 |
|  |  | 95% Confidence Interval for Mean | Lower Bound | 3.79 |  |
|  |  |  | Upper Bound | 4.82 |  |
|  |  | 5% Trimmed Mean | | 4.34 |  |
|  |  | Median | | 5.00 |  |
|  |  | Variance | | .731 |  |
|  |  | Std. Deviation | | .855 |  |
|  |  | Minimum | | 3 |  |
|  |  | Maximum | | 5 |  |
|  |  | Range | | 2 |  |
|  |  | Interquartile Range | | 2 |  |
|  |  | Skewness | | -.705 | .616 |
|  |  | Kurtosis | | -1.240 | 1.191 |
| On the scale shown, how much do you agree the following actions would help address the problem of antibiotic resistance? [People should not keep antibiotics and use them later for other illnesses] | Northern Region | Mean | | 4.42 | .099 |
|  |  | 95% Confidence Interval for Mean | Lower Bound | 4.22 |  |
|  |  |  | Upper Bound | 4.62 |  |
|  |  | 5% Trimmed Mean | | 4.53 |  |
|  |  | Median | | 5.00 |  |
|  |  | Variance | | .967 |  |
|  |  | Std. Deviation | | .984 |  |
|  |  | Minimum | | 1 |  |
|  |  | Maximum | | 5 |  |
|  |  | Range | | 4 |  |
|  |  | Interquartile Range | | 1 |  |
|  |  | Skewness | | -1.658 | .244 |
|  |  | Kurtosis | | 1.784 | .483 |
|  | Northern Harbour Region | Mean | | 4.61 | .056 |
|  |  | 95% Confidence Interval for Mean | Lower Bound | 4.50 |  |
|  |  |  | Upper Bound | 4.73 |  |
|  |  | 5% Trimmed Mean | | 4.71 |  |
|  |  | Median | | 5.00 |  |
|  |  | Variance | | .544 |  |
|  |  | Std. Deviation | | .738 |  |
|  |  | Minimum | | 1 |  |
|  |  | Maximum | | 5 |  |
|  |  | Range | | 4 |  |
|  |  | Interquartile Range | | 1 |  |
|  |  | Skewness | | -2.091 | .186 |
|  |  | Kurtosis | | 4.470 | .369 |
|  | Southern Harbour Region | Mean | | 4.14 | .179 |
|  |  | 95% Confidence Interval for Mean | Lower Bound | 3.78 |  |
|  |  |  | Upper Bound | 4.50 |  |
|  |  | 5% Trimmed Mean | | 4.27 |  |
|  |  | Median | | 5.00 |  |
|  |  | Variance | | 1.345 |  |
|  |  | Std. Deviation | | 1.160 |  |
|  |  | Minimum | | 1 |  |
|  |  | Maximum | | 5 |  |
|  |  | Range | | 4 |  |
|  |  | Interquartile Range | | 2 |  |
|  |  | Skewness | | -1.277 | .365 |
|  |  | Kurtosis | | .852 | .717 |
|  | South Western Region | Mean | | 4.48 | .116 |
|  |  | 95% Confidence Interval for Mean | Lower Bound | 4.24 |  |
|  |  |  | Upper Bound | 4.71 |  |
|  |  | 5% Trimmed Mean | | 4.59 |  |
|  |  | Median | | 5.00 |  |
|  |  | Variance | | .878 |  |
|  |  | Std. Deviation | | .937 |  |
|  |  | Minimum | | 2 |  |
|  |  | Maximum | | 5 |  |
|  |  | Range | | 3 |  |
|  |  | Interquartile Range | | 1 |  |
|  |  | Skewness | | -1.694 | .297 |
|  |  | Kurtosis | | 1.656 | .586 |
|  | Western Region | Mean | | 4.32 | .114 |
|  |  | 95% Confidence Interval for Mean | Lower Bound | 4.10 |  |
|  |  |  | Upper Bound | 4.55 |  |
|  |  | 5% Trimmed Mean | | 4.45 |  |
|  |  | Median | | 5.00 |  |
|  |  | Variance | | 1.128 |  |
|  |  | Std. Deviation | | 1.062 |  |
|  |  | Minimum | | 1 |  |
|  |  | Maximum | | 5 |  |
|  |  | Range | | 4 |  |
|  |  | Interquartile Range | | 1 |  |
|  |  | Skewness | | -1.635 | .258 |
|  |  | Kurtosis | | 1.996 | .511 |
|  | Gozo and Comino | Mean | | 4.62 | .180 |
|  |  | 95% Confidence Interval for Mean | Lower Bound | 4.22 |  |
|  |  |  | Upper Bound | 5.01 |  |
|  |  | 5% Trimmed Mean | | 4.68 |  |
|  |  | Median | | 5.00 |  |
|  |  | Variance | | .423 |  |
|  |  | Std. Deviation | | .650 |  |
|  |  | Minimum | | 3 |  |
|  |  | Maximum | | 5 |  |
|  |  | Range | | 2 |  |
|  |  | Interquartile Range | | 1 |  |
|  |  | Skewness | | -1.576 | .616 |
|  |  | Kurtosis | | 1.801 | 1.191 |
| On the scale shown, how much do you agree the following actions would help address the problem of antibiotic resistance? [Parents should make sure all of their children’s vaccinations are up-to-date] | Northern Region | Mean | | 4.86 | .046 |
|  |  | 95% Confidence Interval for Mean | Lower Bound | 4.77 |  |
|  |  |  | Upper Bound | 4.95 |  |
|  |  | 5% Trimmed Mean | | 4.93 |  |
|  |  | Median | | 5.00 |  |
|  |  | Variance | | .206 |  |
|  |  | Std. Deviation | | .454 |  |
|  |  | Minimum | | 2 |  |
|  |  | Maximum | | 5 |  |
|  |  | Range | | 3 |  |
|  |  | Interquartile Range | | 0 |  |
|  |  | Skewness | | -3.950 | .244 |
|  |  | Kurtosis | | 18.427 | .483 |
|  | Northern Harbour Region | Mean | | 4.78 | .048 |
|  |  | 95% Confidence Interval for Mean | Lower Bound | 4.69 |  |
|  |  |  | Upper Bound | 4.88 |  |
|  |  | 5% Trimmed Mean | | 4.90 |  |
|  |  | Median | | 5.00 |  |
|  |  | Variance | | .394 |  |
|  |  | Std. Deviation | | .628 |  |
|  |  | Minimum | | 1 |  |
|  |  | Maximum | | 5 |  |
|  |  | Range | | 4 |  |
|  |  | Interquartile Range | | 0 |  |
|  |  | Skewness | | -3.411 | .186 |
|  |  | Kurtosis | | 12.684 | .369 |
|  | Southern Harbour Region | Mean | | 4.69 | .130 |
|  |  | 95% Confidence Interval for Mean | Lower Bound | 4.43 |  |
|  |  |  | Upper Bound | 4.95 |  |
|  |  | 5% Trimmed Mean | | 4.82 |  |
|  |  | Median | | 5.00 |  |
|  |  | Variance | | .707 |  |
|  |  | Std. Deviation | | .841 |  |
|  |  | Minimum | | 2 |  |
|  |  | Maximum | | 5 |  |
|  |  | Range | | 3 |  |
|  |  | Interquartile Range | | 0 |  |
|  |  | Skewness | | -2.709 | .365 |
|  |  | Kurtosis | | 6.160 | .717 |
|  | South Western Region | Mean | | 4.89 | .045 |
|  |  | 95% Confidence Interval for Mean | Lower Bound | 4.80 |  |
|  |  |  | Upper Bound | 4.98 |  |
|  |  | 5% Trimmed Mean | | 4.95 |  |
|  |  | Median | | 5.00 |  |
|  |  | Variance | | .129 |  |
|  |  | Std. Deviation | | .359 |  |
|  |  | Minimum | | 3 |  |
|  |  | Maximum | | 5 |  |
|  |  | Range | | 2 |  |
|  |  | Interquartile Range | | 0 |  |
|  |  | Skewness | | -3.574 | .297 |
|  |  | Kurtosis | | 13.394 | .586 |
|  | Western Region | Mean | | 4.76 | .061 |
|  |  | 95% Confidence Interval for Mean | Lower Bound | 4.64 |  |
|  |  |  | Upper Bound | 4.88 |  |
|  |  | 5% Trimmed Mean | | 4.84 |  |
|  |  | Median | | 5.00 |  |
|  |  | Variance | | .325 |  |
|  |  | Std. Deviation | | .570 |  |
|  |  | Minimum | | 3 |  |
|  |  | Maximum | | 5 |  |
|  |  | Range | | 2 |  |
|  |  | Interquartile Range | | 0 |  |
|  |  | Skewness | | -2.286 | .258 |
|  |  | Kurtosis | | 4.057 | .511 |
|  | Gozo and Comino | Mean | | 4.77 | .122 |
|  |  | 95% Confidence Interval for Mean | Lower Bound | 4.50 |  |
|  |  |  | Upper Bound | 5.03 |  |
|  |  | 5% Trimmed Mean | | 4.80 |  |
|  |  | Median | | 5.00 |  |
|  |  | Variance | | .192 |  |
|  |  | Std. Deviation | | .439 |  |
|  |  | Minimum | | 4 |  |
|  |  | Maximum | | 5 |  |
|  |  | Range | | 1 |  |
|  |  | Interquartile Range | | 1 |  |
|  |  | Skewness | | -1.451 | .616 |
|  |  | Kurtosis | | .095 | 1.191 |
| On the scale shown, how much do you agree the following actions would help address the problem of antibiotic resistance? [People should wash their hands regularly] | Northern Region | Mean | | 4.77 | .069 |
|  |  | 95% Confidence Interval for Mean | Lower Bound | 4.63 |  |
|  |  |  | Upper Bound | 4.90 |  |
|  |  | 5% Trimmed Mean | | 4.88 |  |
|  |  | Median | | 5.00 |  |
|  |  | Variance | | .470 |  |
|  |  | Std. Deviation | | .686 |  |
|  |  | Minimum | | 1 |  |
|  |  | Maximum | | 5 |  |
|  |  | Range | | 4 |  |
|  |  | Interquartile Range | | 0 |  |
|  |  | Skewness | | -3.383 | .244 |
|  |  | Kurtosis | | 12.305 | .483 |
|  | Northern Harbour Region | Mean | | 4.81 | .047 |
|  |  | 95% Confidence Interval for Mean | Lower Bound | 4.71 |  |
|  |  |  | Upper Bound | 4.90 |  |
|  |  | 5% Trimmed Mean | | 4.92 |  |
|  |  | Median | | 5.00 |  |
|  |  | Variance | | .380 |  |
|  |  | Std. Deviation | | .617 |  |
|  |  | Minimum | | 1 |  |
|  |  | Maximum | | 5 |  |
|  |  | Range | | 4 |  |
|  |  | Interquartile Range | | 0 |  |
|  |  | Skewness | | -4.122 | .186 |
|  |  | Kurtosis | | 19.332 | .369 |
|  | Southern Harbour Region | Mean | | 4.86 | .073 |
|  |  | 95% Confidence Interval for Mean | Lower Bound | 4.71 |  |
|  |  |  | Upper Bound | 5.00 |  |
|  |  | 5% Trimmed Mean | | 4.95 |  |
|  |  | Median | | 5.00 |  |
|  |  | Variance | | .223 |  |
|  |  | Std. Deviation | | .472 |  |
|  |  | Minimum | | 3 |  |
|  |  | Maximum | | 5 |  |
|  |  | Range | | 2 |  |
|  |  | Interquartile Range | | 0 |  |
|  |  | Skewness | | -3.395 | .365 |
|  |  | Kurtosis | | 10.898 | .717 |
|  | South Western Region | Mean | | 4.88 | .041 |
|  |  | 95% Confidence Interval for Mean | Lower Bound | 4.79 |  |
|  |  |  | Upper Bound | 4.96 |  |
|  |  | 5% Trimmed Mean | | 4.92 |  |
|  |  | Median | | 5.00 |  |
|  |  | Variance | | .110 |  |
|  |  | Std. Deviation | | .331 |  |
|  |  | Minimum | | 4 |  |
|  |  | Maximum | | 5 |  |
|  |  | Range | | 1 |  |
|  |  | Interquartile Range | | 0 |  |
|  |  | Skewness | | -2.349 | .297 |
|  |  | Kurtosis | | 3.630 | .586 |
|  | Western Region | Mean | | 4.77 | .067 |
|  |  | 95% Confidence Interval for Mean | Lower Bound | 4.64 |  |
|  |  |  | Upper Bound | 4.90 |  |
|  |  | 5% Trimmed Mean | | 4.87 |  |
|  |  | Median | | 5.00 |  |
|  |  | Variance | | .388 |  |
|  |  | Std. Deviation | | .623 |  |
|  |  | Minimum | | 2 |  |
|  |  | Maximum | | 5 |  |
|  |  | Range | | 3 |  |
|  |  | Interquartile Range | | 0 |  |
|  |  | Skewness | | -2.749 | .258 |
|  |  | Kurtosis | | 6.791 | .511 |
|  | Gozo and Comino | Mean | | 4.77 | .122 |
|  |  | 95% Confidence Interval for Mean | Lower Bound | 4.50 |  |
|  |  |  | Upper Bound | 5.03 |  |
|  |  | 5% Trimmed Mean | | 4.80 |  |
|  |  | Median | | 5.00 |  |
|  |  | Variance | | .192 |  |
|  |  | Std. Deviation | | .439 |  |
|  |  | Minimum | | 4 |  |
|  |  | Maximum | | 5 |  |
|  |  | Range | | 1 |  |
|  |  | Interquartile Range | | 1 |  |
|  |  | Skewness | | -1.451 | .616 |
|  |  | Kurtosis | | .095 | 1.191 |
| On the scale shown, how much do you agree the following actions would help address the problem of antibiotic resistance? [Doctors should only prescribe antibiotics when they are needed ] | Northern Region | Mean | | 4.92 | .037 |
|  |  | 95% Confidence Interval for Mean | Lower Bound | 4.84 |  |
|  |  |  | Upper Bound | 4.99 |  |
|  |  | 5% Trimmed Mean | | 4.99 |  |
|  |  | Median | | 5.00 |  |
|  |  | Variance | | .138 |  |
|  |  | Std. Deviation | | .371 |  |
|  |  | Minimum | | 2 |  |
|  |  | Maximum | | 5 |  |
|  |  | Range | | 3 |  |
|  |  | Interquartile Range | | 0 |  |
|  |  | Skewness | | -5.913 | .244 |
|  |  | Kurtosis | | 40.985 | .483 |
|  | Northern Harbour Region | Mean | | 4.94 | .022 |
|  |  | 95% Confidence Interval for Mean | Lower Bound | 4.89 |  |
|  |  |  | Upper Bound | 4.98 |  |
|  |  | 5% Trimmed Mean | | 5.00 |  |
|  |  | Median | | 5.00 |  |
|  |  | Variance | | .084 |  |
|  |  | Std. Deviation | | .290 |  |
|  |  | Minimum | | 3 |  |
|  |  | Maximum | | 5 |  |
|  |  | Range | | 2 |  |
|  |  | Interquartile Range | | 0 |  |
|  |  | Skewness | | -4.931 | .186 |
|  |  | Kurtosis | | 25.780 | .369 |
|  | Southern Harbour Region | Mean | | 4.83 | .067 |
|  |  | 95% Confidence Interval for Mean | Lower Bound | 4.70 |  |
|  |  |  | Upper Bound | 4.97 |  |
|  |  | 5% Trimmed Mean | | 4.90 |  |
|  |  | Median | | 5.00 |  |
|  |  | Variance | | .191 |  |
|  |  | Std. Deviation | | .437 |  |
|  |  | Minimum | | 3 |  |
|  |  | Maximum | | 5 |  |
|  |  | Range | | 2 |  |
|  |  | Interquartile Range | | 0 |  |
|  |  | Skewness | | -2.726 | .365 |
|  |  | Kurtosis | | 7.393 | .717 |
|  | South Western Region | Mean | | 4.94 | .030 |
|  |  | 95% Confidence Interval for Mean | Lower Bound | 4.88 |  |
|  |  |  | Upper Bound | 5.00 |  |
|  |  | 5% Trimmed Mean | | 4.99 |  |
|  |  | Median | | 5.00 |  |
|  |  | Variance | | .059 |  |
|  |  | Std. Deviation | | .242 |  |
|  |  | Minimum | | 4 |  |
|  |  | Maximum | | 5 |  |
|  |  | Range | | 1 |  |
|  |  | Interquartile Range | | 0 |  |
|  |  | Skewness | | -3.736 | .297 |
|  |  | Kurtosis | | 12.335 | .586 |
|  | Western Region | Mean | | 4.85 | .062 |
|  |  | 95% Confidence Interval for Mean | Lower Bound | 4.73 |  |
|  |  |  | Upper Bound | 4.97 |  |
|  |  | 5% Trimmed Mean | | 4.97 |  |
|  |  | Median | | 5.00 |  |
|  |  | Variance | | .338 |  |
|  |  | Std. Deviation | | .581 |  |
|  |  | Minimum | | 1 |  |
|  |  | Maximum | | 5 |  |
|  |  | Range | | 4 |  |
|  |  | Interquartile Range | | 0 |  |
|  |  | Skewness | | -4.708 | .258 |
|  |  | Kurtosis | | 24.921 | .511 |
|  | Gozo and Comino | Mean | | 4.92 | .077 |
|  |  | 95% Confidence Interval for Mean | Lower Bound | 4.76 |  |
|  |  |  | Upper Bound | 5.09 |  |
|  |  | 5% Trimmed Mean | | 4.97 |  |
|  |  | Median | | 5.00 |  |
|  |  | Variance | | .077 |  |
|  |  | Std. Deviation | | .277 |  |
|  |  | Minimum | | 4 |  |
|  |  | Maximum | | 5 |  |
|  |  | Range | | 1 |  |
|  |  | Interquartile Range | | 0 |  |
|  |  | Skewness | | -3.606 | .616 |
|  |  | Kurtosis | | 13.000 | 1.191 |
| On the scale shown, how much do you agree the following actions would help address the problem of antibiotic resistance? [Governments should reward the development of new antibiotics] | Northern Region | Mean | | 3.92 | .115 |
|  |  | 95% Confidence Interval for Mean | Lower Bound | 3.69 |  |
|  |  |  | Upper Bound | 4.15 |  |
|  |  | 5% Trimmed Mean | | 4.00 |  |
|  |  | Median | | 4.00 |  |
|  |  | Variance | | 1.292 |  |
|  |  | Std. Deviation | | 1.137 |  |
|  |  | Minimum | | 1 |  |
|  |  | Maximum | | 5 |  |
|  |  | Range | | 4 |  |
|  |  | Interquartile Range | | 2 |  |
|  |  | Skewness | | -.654 | .244 |
|  |  | Kurtosis | | -.526 | .483 |
|  | Northern Harbour Region | Mean | | 4.06 | .083 |
|  |  | 95% Confidence Interval for Mean | Lower Bound | 3.90 |  |
|  |  |  | Upper Bound | 4.23 |  |
|  |  | 5% Trimmed Mean | | 4.17 |  |
|  |  | Median | | 4.00 |  |
|  |  | Variance | | 1.166 |  |
|  |  | Std. Deviation | | 1.080 |  |
|  |  | Minimum | | 1 |  |
|  |  | Maximum | | 5 |  |
|  |  | Range | | 4 |  |
|  |  | Interquartile Range | | 2 |  |
|  |  | Skewness | | -1.065 | .186 |
|  |  | Kurtosis | | .601 | .369 |
|  | Southern Harbour Region | Mean | | 3.90 | .195 |
|  |  | 95% Confidence Interval for Mean | Lower Bound | 3.51 |  |
|  |  |  | Upper Bound | 4.30 |  |
|  |  | 5% Trimmed Mean | | 4.01 |  |
|  |  | Median | | 4.00 |  |
|  |  | Variance | | 1.600 |  |
|  |  | Std. Deviation | | 1.265 |  |
|  |  | Minimum | | 1 |  |
|  |  | Maximum | | 5 |  |
|  |  | Range | | 4 |  |
|  |  | Interquartile Range | | 2 |  |
|  |  | Skewness | | -.875 | .365 |
|  |  | Kurtosis | | -.190 | .717 |
|  | South Western Region | Mean | | 3.94 | .126 |
|  |  | 95% Confidence Interval for Mean | Lower Bound | 3.69 |  |
|  |  |  | Upper Bound | 4.19 |  |
|  |  | 5% Trimmed Mean | | 4.03 |  |
|  |  | Median | | 4.00 |  |
|  |  | Variance | | 1.027 |  |
|  |  | Std. Deviation | | 1.014 |  |
|  |  | Minimum | | 1 |  |
|  |  | Maximum | | 5 |  |
|  |  | Range | | 4 |  |
|  |  | Interquartile Range | | 2 |  |
|  |  | Skewness | | -.988 | .297 |
|  |  | Kurtosis | | 1.159 | .586 |
|  | Western Region | Mean | | 4.08 | .106 |
|  |  | 95% Confidence Interval for Mean | Lower Bound | 3.87 |  |
|  |  |  | Upper Bound | 4.29 |  |
|  |  | 5% Trimmed Mean | | 4.17 |  |
|  |  | Median | | 4.00 |  |
|  |  | Variance | | .982 |  |
|  |  | Std. Deviation | | .991 |  |
|  |  | Minimum | | 1 |  |
|  |  | Maximum | | 5 |  |
|  |  | Range | | 4 |  |
|  |  | Interquartile Range | | 2 |  |
|  |  | Skewness | | -.898 | .258 |
|  |  | Kurtosis | | .428 | .511 |
|  | Gozo and Comino | Mean | | 4.23 | .303 |
|  |  | 95% Confidence Interval for Mean | Lower Bound | 3.57 |  |
|  |  |  | Upper Bound | 4.89 |  |
|  |  | 5% Trimmed Mean | | 4.31 |  |
|  |  | Median | | 5.00 |  |
|  |  | Variance | | 1.192 |  |
|  |  | Std. Deviation | | 1.092 |  |
|  |  | Minimum | | 2 |  |
|  |  | Maximum | | 5 |  |
|  |  | Range | | 3 |  |
|  |  | Interquartile Range | | 2 |  |
|  |  | Skewness | | -.988 | .616 |
|  |  | Kurtosis | | -.586 | 1.191 |
| On the scale shown, how much do you agree the following actions would help address the problem of antibiotic resistance? [Pharmaceutical companies should develop new antibiotics] | Northern Region | Mean | | 3.84 | .106 |
|  |  | 95% Confidence Interval for Mean | Lower Bound | 3.63 |  |
|  |  |  | Upper Bound | 4.05 |  |
|  |  | 5% Trimmed Mean | | 3.92 |  |
|  |  | Median | | 4.00 |  |
|  |  | Variance | | 1.107 |  |
|  |  | Std. Deviation | | 1.052 |  |
|  |  | Minimum | | 1 |  |
|  |  | Maximum | | 5 |  |
|  |  | Range | | 4 |  |
|  |  | Interquartile Range | | 2 |  |
|  |  | Skewness | | -.695 | .244 |
|  |  | Kurtosis | | .184 | .483 |
|  | Northern Harbour Region | Mean | | 4.04 | .081 |
|  |  | 95% Confidence Interval for Mean | Lower Bound | 3.88 |  |
|  |  |  | Upper Bound | 4.20 |  |
|  |  | 5% Trimmed Mean | | 4.13 |  |
|  |  | Median | | 4.00 |  |
|  |  | Variance | | 1.122 |  |
|  |  | Std. Deviation | | 1.059 |  |
|  |  | Minimum | | 1 |  |
|  |  | Maximum | | 5 |  |
|  |  | Range | | 4 |  |
|  |  | Interquartile Range | | 2 |  |
|  |  | Skewness | | -.864 | .186 |
|  |  | Kurtosis | | -.026 | .369 |
|  | Southern Harbour Region | Mean | | 4.10 | .144 |
|  |  | 95% Confidence Interval for Mean | Lower Bound | 3.80 |  |
|  |  |  | Upper Bound | 4.39 |  |
|  |  | 5% Trimmed Mean | | 4.16 |  |
|  |  | Median | | 4.00 |  |
|  |  | Variance | | .869 |  |
|  |  | Std. Deviation | | .932 |  |
|  |  | Minimum | | 1 |  |
|  |  | Maximum | | 5 |  |
|  |  | Range | | 4 |  |
|  |  | Interquartile Range | | 2 |  |
|  |  | Skewness | | -.956 | .365 |
|  |  | Kurtosis | | 1.166 | .717 |
|  | South Western Region | Mean | | 3.98 | .121 |
|  |  | 95% Confidence Interval for Mean | Lower Bound | 3.74 |  |
|  |  |  | Upper Bound | 4.23 |  |
|  |  | 5% Trimmed Mean | | 4.06 |  |
|  |  | Median | | 4.00 |  |
|  |  | Variance | | .953 |  |
|  |  | Std. Deviation | | .976 |  |
|  |  | Minimum | | 1 |  |
|  |  | Maximum | | 5 |  |
|  |  | Range | | 4 |  |
|  |  | Interquartile Range | | 2 |  |
|  |  | Skewness | | -.696 | .297 |
|  |  | Kurtosis | | .000 | .586 |
|  | Western Region | Mean | | 4.21 | .100 |
|  |  | 95% Confidence Interval for Mean | Lower Bound | 4.01 |  |
|  |  |  | Upper Bound | 4.40 |  |
|  |  | 5% Trimmed Mean | | 4.28 |  |
|  |  | Median | | 4.00 |  |
|  |  | Variance | | .864 |  |
|  |  | Std. Deviation | | .929 |  |
|  |  | Minimum | | 1 |  |
|  |  | Maximum | | 5 |  |
|  |  | Range | | 4 |  |
|  |  | Interquartile Range | | 1 |  |
|  |  | Skewness | | -.962 | .258 |
|  |  | Kurtosis | | .377 | .511 |
|  | Gozo and Comino | Mean | | 4.08 | .329 |
|  |  | 95% Confidence Interval for Mean | Lower Bound | 3.36 |  |
|  |  |  | Upper Bound | 4.79 |  |
|  |  | 5% Trimmed Mean | | 4.20 |  |
|  |  | Median | | 4.00 |  |
|  |  | Variance | | 1.410 |  |
|  |  | Std. Deviation | | 1.188 |  |
|  |  | Minimum | | 1 |  |
|  |  | Maximum | | 5 |  |
|  |  | Range | | 4 |  |
|  |  | Interquartile Range | | 2 |  |
|  |  | Skewness | | -1.583 | .616 |
|  |  | Kurtosis | | 2.775 | 1.191 |
| On the scale shown, how much do you agree with the following statements? [Antibiotic resistance is one of the biggest problems the world faces] | Northern Region | Mean | | 3.60 | .105 |
|  |  | 95% Confidence Interval for Mean | Lower Bound | 3.39 |  |
|  |  |  | Upper Bound | 3.81 |  |
|  |  | 5% Trimmed Mean | | 3.64 |  |
|  |  | Median | | 4.00 |  |
|  |  | Variance | | 1.087 |  |
|  |  | Std. Deviation | | 1.043 |  |
|  |  | Minimum | | 1 |  |
|  |  | Maximum | | 5 |  |
|  |  | Range | | 4 |  |
|  |  | Interquartile Range | | 1 |  |
|  |  | Skewness | | -.194 | .244 |
|  |  | Kurtosis | | -.674 | .483 |
|  | Northern Harbour Region | Mean | | 3.65 | .088 |
|  |  | 95% Confidence Interval for Mean | Lower Bound | 3.48 |  |
|  |  |  | Upper Bound | 3.83 |  |
|  |  | 5% Trimmed Mean | | 3.72 |  |
|  |  | Median | | 4.00 |  |
|  |  | Variance | | 1.333 |  |
|  |  | Std. Deviation | | 1.155 |  |
|  |  | Minimum | | 1 |  |
|  |  | Maximum | | 5 |  |
|  |  | Range | | 4 |  |
|  |  | Interquartile Range | | 2 |  |
|  |  | Skewness | | -.454 | .186 |
|  |  | Kurtosis | | -.693 | .369 |
|  | Southern Harbour Region | Mean | | 3.55 | .171 |
|  |  | 95% Confidence Interval for Mean | Lower Bound | 3.20 |  |
|  |  |  | Upper Bound | 3.89 |  |
|  |  | 5% Trimmed Mean | | 3.58 |  |
|  |  | Median | | 4.00 |  |
|  |  | Variance | | 1.229 |  |
|  |  | Std. Deviation | | 1.109 |  |
|  |  | Minimum | | 1 |  |
|  |  | Maximum | | 5 |  |
|  |  | Range | | 4 |  |
|  |  | Interquartile Range | | 1 |  |
|  |  | Skewness | | -.239 | .365 |
|  |  | Kurtosis | | -.835 | .717 |
|  | South Western Region | Mean | | 3.75 | .140 |
|  |  | 95% Confidence Interval for Mean | Lower Bound | 3.47 |  |
|  |  |  | Upper Bound | 4.03 |  |
|  |  | 5% Trimmed Mean | | 3.83 |  |
|  |  | Median | | 4.00 |  |
|  |  | Variance | | 1.282 |  |
|  |  | Std. Deviation | | 1.132 |  |
|  |  | Minimum | | 1 |  |
|  |  | Maximum | | 5 |  |
|  |  | Range | | 4 |  |
|  |  | Interquartile Range | | 2 |  |
|  |  | Skewness | | -.427 | .297 |
|  |  | Kurtosis | | -.452 | .586 |
|  | Western Region | Mean | | 3.74 | .110 |
|  |  | 95% Confidence Interval for Mean | Lower Bound | 3.52 |  |
|  |  |  | Upper Bound | 3.95 |  |
|  |  | 5% Trimmed Mean | | 3.79 |  |
|  |  | Median | | 4.00 |  |
|  |  | Variance | | 1.057 |  |
|  |  | Std. Deviation | | 1.028 |  |
|  |  | Minimum | | 1 |  |
|  |  | Maximum | | 5 |  |
|  |  | Range | | 4 |  |
|  |  | Interquartile Range | | 2 |  |
|  |  | Skewness | | -.364 | .258 |
|  |  | Kurtosis | | -.439 | .511 |
|  | Gozo and Comino | Mean | | 2.77 | .343 |
|  |  | 95% Confidence Interval for Mean | Lower Bound | 2.02 |  |
|  |  |  | Upper Bound | 3.52 |  |
|  |  | 5% Trimmed Mean | | 2.74 |  |
|  |  | Median | | 3.00 |  |
|  |  | Variance | | 1.526 |  |
|  |  | Std. Deviation | | 1.235 |  |
|  |  | Minimum | | 1 |  |
|  |  | Maximum | | 5 |  |
|  |  | Range | | 4 |  |
|  |  | Interquartile Range | | 2 |  |
|  |  | Skewness | | -.113 | .616 |
|  |  | Kurtosis | | -.426 | 1.191 |
| On the scale shown, how much do you agree with the following statements? [Medical experts will solve the problem of antibiotic resistance before it becomes too serious] | Northern Region | Mean | | 2.86 | .104 |
|  |  | 95% Confidence Interval for Mean | Lower Bound | 2.65 |  |
|  |  |  | Upper Bound | 3.06 |  |
|  |  | 5% Trimmed Mean | | 2.84 |  |
|  |  | Median | | 3.00 |  |
|  |  | Variance | | 1.052 |  |
|  |  | Std. Deviation | | 1.025 |  |
|  |  | Minimum | | 1 |  |
|  |  | Maximum | | 5 |  |
|  |  | Range | | 4 |  |
|  |  | Interquartile Range | | 1 |  |
|  |  | Skewness | | .117 | .244 |
|  |  | Kurtosis | | .142 | .483 |
|  | Northern Harbour Region | Mean | | 2.84 | .078 |
|  |  | 95% Confidence Interval for Mean | Lower Bound | 2.68 |  |
|  |  |  | Upper Bound | 2.99 |  |
|  |  | 5% Trimmed Mean | | 2.82 |  |
|  |  | Median | | 3.00 |  |
|  |  | Variance | | 1.032 |  |
|  |  | Std. Deviation | | 1.016 |  |
|  |  | Minimum | | 1 |  |
|  |  | Maximum | | 5 |  |
|  |  | Range | | 4 |  |
|  |  | Interquartile Range | | 1 |  |
|  |  | Skewness | | .198 | .186 |
|  |  | Kurtosis | | -.132 | .369 |
|  | Southern Harbour Region | Mean | | 3.07 | .165 |
|  |  | 95% Confidence Interval for Mean | Lower Bound | 2.74 |  |
|  |  |  | Upper Bound | 3.40 |  |
|  |  | 5% Trimmed Mean | | 3.08 |  |
|  |  | Median | | 3.00 |  |
|  |  | Variance | | 1.141 |  |
|  |  | Std. Deviation | | 1.068 |  |
|  |  | Minimum | | 1 |  |
|  |  | Maximum | | 5 |  |
|  |  | Range | | 4 |  |
|  |  | Interquartile Range | | 1 |  |
|  |  | Skewness | | -.148 | .365 |
|  |  | Kurtosis | | -.119 | .717 |
|  | South Western Region | Mean | | 3.06 | .142 |
|  |  | 95% Confidence Interval for Mean | Lower Bound | 2.78 |  |
|  |  |  | Upper Bound | 3.34 |  |
|  |  | 5% Trimmed Mean | | 3.07 |  |
|  |  | Median | | 3.00 |  |
|  |  | Variance | | 1.309 |  |
|  |  | Std. Deviation | | 1.144 |  |
|  |  | Minimum | | 1 |  |
|  |  | Maximum | | 5 |  |
|  |  | Range | | 4 |  |
|  |  | Interquartile Range | | 2 |  |
|  |  | Skewness | | -.124 | .297 |
|  |  | Kurtosis | | -.371 | .586 |
|  | Western Region | Mean | | 3.05 | .117 |
|  |  | 95% Confidence Interval for Mean | Lower Bound | 2.81 |  |
|  |  |  | Upper Bound | 3.28 |  |
|  |  | 5% Trimmed Mean | | 3.05 |  |
|  |  | Median | | 3.00 |  |
|  |  | Variance | | 1.184 |  |
|  |  | Std. Deviation | | 1.088 |  |
|  |  | Minimum | | 1 |  |
|  |  | Maximum | | 5 |  |
|  |  | Range | | 4 |  |
|  |  | Interquartile Range | | 2 |  |
|  |  | Skewness | | .073 | .258 |
|  |  | Kurtosis | | -.410 | .511 |
|  | Gozo and Comino | Mean | | 3.23 | .303 |
|  |  | 95% Confidence Interval for Mean | Lower Bound | 2.57 |  |
|  |  |  | Upper Bound | 3.89 |  |
|  |  | 5% Trimmed Mean | | 3.26 |  |
|  |  | Median | | 3.00 |  |
|  |  | Variance | | 1.192 |  |
|  |  | Std. Deviation | | 1.092 |  |
|  |  | Minimum | | 1 |  |
|  |  | Maximum | | 5 |  |
|  |  | Range | | 4 |  |
|  |  | Interquartile Range | | 1 |  |
|  |  | Skewness | | -.081 | .616 |
|  |  | Kurtosis | | .668 | 1.191 |
| On the scale shown, how much do you agree with the following statements? [Everyone needs to take responsibility for using antibiotics responsibly] | Northern Region | Mean | | 4.78 | .049 |
|  |  | 95% Confidence Interval for Mean | Lower Bound | 4.68 |  |
|  |  |  | Upper Bound | 4.87 |  |
|  |  | 5% Trimmed Mean | | 4.84 |  |
|  |  | Median | | 5.00 |  |
|  |  | Variance | | .238 |  |
|  |  | Std. Deviation | | .488 |  |
|  |  | Minimum | | 3 |  |
|  |  | Maximum | | 5 |  |
|  |  | Range | | 2 |  |
|  |  | Interquartile Range | | 0 |  |
|  |  | Skewness | | -2.121 | .244 |
|  |  | Kurtosis | | 3.852 | .483 |
|  | Northern Harbour Region | Mean | | 4.84 | .035 |
|  |  | 95% Confidence Interval for Mean | Lower Bound | 4.77 |  |
|  |  |  | Upper Bound | 4.91 |  |
|  |  | 5% Trimmed Mean | | 4.91 |  |
|  |  | Median | | 5.00 |  |
|  |  | Variance | | .204 |  |
|  |  | Std. Deviation | | .452 |  |
|  |  | Minimum | | 2 |  |
|  |  | Maximum | | 5 |  |
|  |  | Range | | 3 |  |
|  |  | Interquartile Range | | 0 |  |
|  |  | Skewness | | -3.343 | .186 |
|  |  | Kurtosis | | 12.845 | .369 |
|  | Southern Harbour Region | Mean | | 4.67 | .100 |
|  |  | 95% Confidence Interval for Mean | Lower Bound | 4.46 |  |
|  |  |  | Upper Bound | 4.87 |  |
|  |  | 5% Trimmed Mean | | 4.76 |  |
|  |  | Median | | 5.00 |  |
|  |  | Variance | | .423 |  |
|  |  | Std. Deviation | | .650 |  |
|  |  | Minimum | | 2 |  |
|  |  | Maximum | | 5 |  |
|  |  | Range | | 3 |  |
|  |  | Interquartile Range | | 1 |  |
|  |  | Skewness | | -2.340 | .365 |
|  |  | Kurtosis | | 6.317 | .717 |
|  | South Western Region | Mean | | 4.88 | .047 |
|  |  | 95% Confidence Interval for Mean | Lower Bound | 4.78 |  |
|  |  |  | Upper Bound | 4.97 |  |
|  |  | 5% Trimmed Mean | | 4.94 |  |
|  |  | Median | | 5.00 |  |
|  |  | Variance | | .141 |  |
|  |  | Std. Deviation | | .375 |  |
|  |  | Minimum | | 3 |  |
|  |  | Maximum | | 5 |  |
|  |  | Range | | 2 |  |
|  |  | Interquartile Range | | 0 |  |
|  |  | Skewness | | -3.217 | .297 |
|  |  | Kurtosis | | 10.667 | .586 |
|  | Western Region | Mean | | 4.76 | .065 |
|  |  | 95% Confidence Interval for Mean | Lower Bound | 4.63 |  |
|  |  |  | Upper Bound | 4.89 |  |
|  |  | 5% Trimmed Mean | | 4.86 |  |
|  |  | Median | | 5.00 |  |
|  |  | Variance | | .371 |  |
|  |  | Std. Deviation | | .609 |  |
|  |  | Minimum | | 2 |  |
|  |  | Maximum | | 5 |  |
|  |  | Range | | 3 |  |
|  |  | Interquartile Range | | 0 |  |
|  |  | Skewness | | -2.665 | .258 |
|  |  | Kurtosis | | 6.718 | .511 |
|  | Gozo and Comino | Mean | | 4.92 | .077 |
|  |  | 95% Confidence Interval for Mean | Lower Bound | 4.76 |  |
|  |  |  | Upper Bound | 5.09 |  |
|  |  | 5% Trimmed Mean | | 4.97 |  |
|  |  | Median | | 5.00 |  |
|  |  | Variance | | .077 |  |
|  |  | Std. Deviation | | .277 |  |
|  |  | Minimum | | 4 |  |
|  |  | Maximum | | 5 |  |
|  |  | Range | | 1 |  |
|  |  | Interquartile Range | | 0 |  |
|  |  | Skewness | | -3.606 | .616 |
|  |  | Kurtosis | | 13.000 | 1.191 |
| On the scale shown, how much do you agree with the following statements? [There is not much people like me can do to stop antibiotic resistance] | Northern Region | Mean | | 2.68 | .123 |
|  |  | 95% Confidence Interval for Mean | Lower Bound | 2.44 |  |
|  |  |  | Upper Bound | 2.93 |  |
|  |  | 5% Trimmed Mean | | 2.65 |  |
|  |  | Median | | 3.00 |  |
|  |  | Variance | | 1.476 |  |
|  |  | Std. Deviation | | 1.215 |  |
|  |  | Minimum | | 1 |  |
|  |  | Maximum | | 5 |  |
|  |  | Range | | 4 |  |
|  |  | Interquartile Range | | 2 |  |
|  |  | Skewness | | .035 | .244 |
|  |  | Kurtosis | | -.973 | .483 |
|  | Northern Harbour Region | Mean | | 2.87 | .091 |
|  |  | 95% Confidence Interval for Mean | Lower Bound | 2.69 |  |
|  |  |  | Upper Bound | 3.04 |  |
|  |  | 5% Trimmed Mean | | 2.85 |  |
|  |  | Median | | 3.00 |  |
|  |  | Variance | | 1.411 |  |
|  |  | Std. Deviation | | 1.188 |  |
|  |  | Minimum | | 1 |  |
|  |  | Maximum | | 5 |  |
|  |  | Range | | 4 |  |
|  |  | Interquartile Range | | 2 |  |
|  |  | Skewness | | -.120 | .186 |
|  |  | Kurtosis | | -.903 | .369 |
|  | Southern Harbour Region | Mean | | 3.19 | .211 |
|  |  | 95% Confidence Interval for Mean | Lower Bound | 2.76 |  |
|  |  |  | Upper Bound | 3.62 |  |
|  |  | 5% Trimmed Mean | | 3.21 |  |
|  |  | Median | | 3.00 |  |
|  |  | Variance | | 1.865 |  |
|  |  | Std. Deviation | | 1.366 |  |
|  |  | Minimum | | 1 |  |
|  |  | Maximum | | 5 |  |
|  |  | Range | | 4 |  |
|  |  | Interquartile Range | | 2 |  |
|  |  | Skewness | | -.181 | .365 |
|  |  | Kurtosis | | -1.163 | .717 |
|  | South Western Region | Mean | | 2.83 | .148 |
|  |  | 95% Confidence Interval for Mean | Lower Bound | 2.54 |  |
|  |  |  | Upper Bound | 3.13 |  |
|  |  | 5% Trimmed Mean | | 2.81 |  |
|  |  | Median | | 3.00 |  |
|  |  | Variance | | 1.424 |  |
|  |  | Std. Deviation | | 1.193 |  |
|  |  | Minimum | | 1 |  |
|  |  | Maximum | | 5 |  |
|  |  | Range | | 4 |  |
|  |  | Interquartile Range | | 2 |  |
|  |  | Skewness | | .110 | .297 |
|  |  | Kurtosis | | -.801 | .586 |
|  | Western Region | Mean | | 2.80 | .136 |
|  |  | 95% Confidence Interval for Mean | Lower Bound | 2.53 |  |
|  |  |  | Upper Bound | 3.07 |  |
|  |  | 5% Trimmed Mean | | 2.78 |  |
|  |  | Median | | 3.00 |  |
|  |  | Variance | | 1.601 |  |
|  |  | Std. Deviation | | 1.265 |  |
|  |  | Minimum | | 1 |  |
|  |  | Maximum | | 5 |  |
|  |  | Range | | 4 |  |
|  |  | Interquartile Range | | 2 |  |
|  |  | Skewness | | .167 | .258 |
|  |  | Kurtosis | | -1.184 | .511 |
|  | Gozo and Comino | Mean | | 3.23 | .323 |
|  |  | 95% Confidence Interval for Mean | Lower Bound | 2.53 |  |
|  |  |  | Upper Bound | 3.94 |  |
|  |  | 5% Trimmed Mean | | 3.26 |  |
|  |  | Median | | 3.00 |  |
|  |  | Variance | | 1.359 |  |
|  |  | Std. Deviation | | 1.166 |  |
|  |  | Minimum | | 1 |  |
|  |  | Maximum | | 5 |  |
|  |  | Range | | 4 |  |
|  |  | Interquartile Range | | 2 |  |
|  |  | Skewness | | -.152 | .616 |
|  |  | Kurtosis | | -.193 | 1.191 |
| On the scale shown, how much do you agree with the following statements? [I am worried about the impact that antibiotic resistance will have on my health, and that of my family] | Northern Region | Mean | | 3.82 | .100 |
|  |  | 95% Confidence Interval for Mean | Lower Bound | 3.62 |  |
|  |  |  | Upper Bound | 4.01 |  |
|  |  | 5% Trimmed Mean | | 3.86 |  |
|  |  | Median | | 4.00 |  |
|  |  | Variance | | .976 |  |
|  |  | Std. Deviation | | .988 |  |
|  |  | Minimum | | 1 |  |
|  |  | Maximum | | 5 |  |
|  |  | Range | | 4 |  |
|  |  | Interquartile Range | | 2 |  |
|  |  | Skewness | | -.406 | .244 |
|  |  | Kurtosis | | -.570 | .483 |
|  | Northern Harbour Region | Mean | | 3.90 | .080 |
|  |  | 95% Confidence Interval for Mean | Lower Bound | 3.74 |  |
|  |  |  | Upper Bound | 4.06 |  |
|  |  | 5% Trimmed Mean | | 3.98 |  |
|  |  | Median | | 4.00 |  |
|  |  | Variance | | 1.102 |  |
|  |  | Std. Deviation | | 1.050 |  |
|  |  | Minimum | | 1 |  |
|  |  | Maximum | | 5 |  |
|  |  | Range | | 4 |  |
|  |  | Interquartile Range | | 2 |  |
|  |  | Skewness | | -.818 | .186 |
|  |  | Kurtosis | | .112 | .369 |
|  | Southern Harbour Region | Mean | | 3.98 | .147 |
|  |  | 95% Confidence Interval for Mean | Lower Bound | 3.68 |  |
|  |  |  | Upper Bound | 4.27 |  |
|  |  | 5% Trimmed Mean | | 4.03 |  |
|  |  | Median | | 4.00 |  |
|  |  | Variance | | .902 |  |
|  |  | Std. Deviation | | .950 |  |
|  |  | Minimum | | 2 |  |
|  |  | Maximum | | 5 |  |
|  |  | Range | | 3 |  |
|  |  | Interquartile Range | | 2 |  |
|  |  | Skewness | | -.489 | .365 |
|  |  | Kurtosis | | -.757 | .717 |
|  | South Western Region | Mean | | 3.69 | .158 |
|  |  | 95% Confidence Interval for Mean | Lower Bound | 3.38 |  |
|  |  |  | Upper Bound | 4.01 |  |
|  |  | 5% Trimmed Mean | | 3.77 |  |
|  |  | Median | | 4.00 |  |
|  |  | Variance | | 1.623 |  |
|  |  | Std. Deviation | | 1.274 |  |
|  |  | Minimum | | 1 |  |
|  |  | Maximum | | 5 |  |
|  |  | Range | | 4 |  |
|  |  | Interquartile Range | | 2 |  |
|  |  | Skewness | | -.798 | .297 |
|  |  | Kurtosis | | -.352 | .586 |
|  | Western Region | Mean | | 4.06 | .105 |
|  |  | 95% Confidence Interval for Mean | Lower Bound | 3.85 |  |
|  |  |  | Upper Bound | 4.27 |  |
|  |  | 5% Trimmed Mean | | 4.14 |  |
|  |  | Median | | 4.00 |  |
|  |  | Variance | | .962 |  |
|  |  | Std. Deviation | | .981 |  |
|  |  | Minimum | | 1 |  |
|  |  | Maximum | | 5 |  |
|  |  | Range | | 4 |  |
|  |  | Interquartile Range | | 2 |  |
|  |  | Skewness | | -.950 | .258 |
|  |  | Kurtosis | | .649 | .511 |
|  | Gozo and Comino | Mean | | 3.54 | .332 |
|  |  | 95% Confidence Interval for Mean | Lower Bound | 2.81 |  |
|  |  |  | Upper Bound | 4.26 |  |
|  |  | 5% Trimmed Mean | | 3.60 |  |
|  |  | Median | | 3.00 |  |
|  |  | Variance | | 1.436 |  |
|  |  | Std. Deviation | | 1.198 |  |
|  |  | Minimum | | 1 |  |
|  |  | Maximum | | 5 |  |
|  |  | Range | | 4 |  |
|  |  | Interquartile Range | | 2 |  |
|  |  | Skewness | | -.278 | .616 |
|  |  | Kurtosis | | .112 | 1.191 |
| On the scale shown, how much do you agree with the following statements? [I am not at risk of getting an antibiotic-resistant infection, as long as I take my antibiotics correctly] | Northern Region | Mean | | 2.81 | .136 |
|  |  | 95% Confidence Interval for Mean | Lower Bound | 2.54 |  |
|  |  |  | Upper Bound | 3.08 |  |
|  |  | 5% Trimmed Mean | | 2.78 |  |
|  |  | Median | | 3.00 |  |
|  |  | Variance | | 1.807 |  |
|  |  | Std. Deviation | | 1.344 |  |
|  |  | Minimum | | 1 |  |
|  |  | Maximum | | 5 |  |
|  |  | Range | | 4 |  |
|  |  | Interquartile Range | | 2 |  |
|  |  | Skewness | | .077 | .244 |
|  |  | Kurtosis | | -1.171 | .483 |
|  | Northern Harbour Region | Mean | | 2.61 | .099 |
|  |  | 95% Confidence Interval for Mean | Lower Bound | 2.41 |  |
|  |  |  | Upper Bound | 2.80 |  |
|  |  | 5% Trimmed Mean | | 2.56 |  |
|  |  | Median | | 3.00 |  |
|  |  | Variance | | 1.675 |  |
|  |  | Std. Deviation | | 1.294 |  |
|  |  | Minimum | | 1 |  |
|  |  | Maximum | | 5 |  |
|  |  | Range | | 4 |  |
|  |  | Interquartile Range | | 2 |  |
|  |  | Skewness | | .335 | .186 |
|  |  | Kurtosis | | -.925 | .369 |
|  | Southern Harbour Region | Mean | | 3.24 | .212 |
|  |  | 95% Confidence Interval for Mean | Lower Bound | 2.81 |  |
|  |  |  | Upper Bound | 3.67 |  |
|  |  | 5% Trimmed Mean | | 3.26 |  |
|  |  | Median | | 4.00 |  |
|  |  | Variance | | 1.893 |  |
|  |  | Std. Deviation | | 1.376 |  |
|  |  | Minimum | | 1 |  |
|  |  | Maximum | | 5 |  |
|  |  | Range | | 4 |  |
|  |  | Interquartile Range | | 2 |  |
|  |  | Skewness | | -.511 | .365 |
|  |  | Kurtosis | | -1.003 | .717 |
|  | South Western Region | Mean | | 3.09 | .165 |
|  |  | 95% Confidence Interval for Mean | Lower Bound | 2.76 |  |
|  |  |  | Upper Bound | 3.42 |  |
|  |  | 5% Trimmed Mean | | 3.10 |  |
|  |  | Median | | 3.00 |  |
|  |  | Variance | | 1.773 |  |
|  |  | Std. Deviation | | 1.331 |  |
|  |  | Minimum | | 1 |  |
|  |  | Maximum | | 5 |  |
|  |  | Range | | 4 |  |
|  |  | Interquartile Range | | 2 |  |
|  |  | Skewness | | -.174 | .297 |
|  |  | Kurtosis | | -1.062 | .586 |
|  | Western Region | Mean | | 2.80 | .143 |
|  |  | 95% Confidence Interval for Mean | Lower Bound | 2.52 |  |
|  |  |  | Upper Bound | 3.09 |  |
|  |  | 5% Trimmed Mean | | 2.78 |  |
|  |  | Median | | 3.00 |  |
|  |  | Variance | | 1.787 |  |
|  |  | Std. Deviation | | 1.337 |  |
|  |  | Minimum | | 1 |  |
|  |  | Maximum | | 5 |  |
|  |  | Range | | 4 |  |
|  |  | Interquartile Range | | 2 |  |
|  |  | Skewness | | .128 | .258 |
|  |  | Kurtosis | | -1.055 | .511 |
|  | Gozo and Comino | Mean | | 2.69 | .308 |
|  |  | 95% Confidence Interval for Mean | Lower Bound | 2.02 |  |
|  |  |  | Upper Bound | 3.36 |  |
|  |  | 5% Trimmed Mean | | 2.66 |  |
|  |  | Median | | 2.00 |  |
|  |  | Variance | | 1.231 |  |
|  |  | Std. Deviation | | 1.109 |  |
|  |  | Minimum | | 1 |  |
|  |  | Maximum | | 5 |  |
|  |  | Range | | 4 |  |
|  |  | Interquartile Range | | 2 |  |
|  |  | Skewness | | .722 | .616 |
|  |  | Kurtosis | | .061 | 1.191 |

| **Tests of Normality** | | | | | | | |
| --- | --- | --- | --- | --- | --- | --- | --- |
|  | Which region of Malta do you live in? | Kolmogorov-Smirnov^a^ | | | Shapiro-Wilk | | |
|  |  | Statistic | df | Sig. | Statistic | df | Sig. |
| On the scale shown, how much do you agree the following actions would help address the problem of antibiotic resistance? [People should use antibiotics only when they are prescribed by a doctor or nurse] | Northern Region | .515 | 98 | <.001 | .324 | 98 | <.001 |
|  | Northern Harbour Region | .519 | 171 | <.001 | .367 | 171 | <.001 |
|  | Southern Harbour Region | .477 | 42 | <.001 | .500 | 42 | <.001 |
|  | South Western Region | .532 | 65 | <.001 | .328 | 65 | <.001 |
|  | Western Region | .516 | 87 | <.001 | .306 | 87 | <.001 |
|  | Gozo and Comino | .532 | 13 | <.001 | .311 | 13 | <.001 |
| On the scale shown, how much do you agree the following actions would help address the problem of antibiotic resistance? [Farmers should give fewer antibiotics to food-producing animals] | Northern Region | .400 | 98 | <.001 | .665 | 98 | <.001 |
|  | Northern Harbour Region | .418 | 171 | <.001 | .622 | 171 | <.001 |
|  | Southern Harbour Region | .296 | 42 | <.001 | .769 | 42 | <.001 |
|  | South Western Region | .409 | 65 | <.001 | .625 | 65 | <.001 |
|  | Western Region | .334 | 87 | <.001 | .738 | 87 | <.001 |
|  | Gozo and Comino | .329 | 13 | <.001 | .744 | 13 | .002 |
| On the scale shown, how much do you agree the following actions would help address the problem of antibiotic resistance? [People should not keep antibiotics and use them later for other illnesses] | Northern Region | .396 | 98 | <.001 | .645 | 98 | <.001 |
|  | Northern Harbour Region | .436 | 171 | <.001 | .583 | 171 | <.001 |
|  | Southern Harbour Region | .318 | 42 | <.001 | .750 | 42 | <.001 |
|  | South Western Region | .419 | 65 | <.001 | .608 | 65 | <.001 |
|  | Western Region | .359 | 87 | <.001 | .683 | 87 | <.001 |
|  | Gozo and Comino | .415 | 13 | <.001 | .650 | 13 | <.001 |
| On the scale shown, how much do you agree the following actions would help address the problem of antibiotic resistance? [Parents should make sure all of their children’s vaccinations are up-to-date] | Northern Region | .511 | 98 | <.001 | .353 | 98 | <.001 |
|  | Northern Harbour Region | .500 | 171 | <.001 | .396 | 171 | <.001 |
|  | Southern Harbour Region | .501 | 42 | <.001 | .412 | 42 | <.001 |
|  | South Western Region | .526 | 65 | <.001 | .332 | 65 | <.001 |
|  | Western Region | .492 | 87 | <.001 | .470 | 87 | <.001 |
|  | Gozo and Comino | .470 | 13 | <.001 | .533 | 13 | <.001 |
| On the scale shown, how much do you agree the following actions would help address the problem of antibiotic resistance? [People should wash their hands regularly] | Northern Region | .501 | 98 | <.001 | .395 | 98 | <.001 |
|  | Northern Harbour Region | .500 | 171 | <.001 | .354 | 171 | <.001 |
|  | Southern Harbour Region | .524 | 42 | <.001 | .336 | 42 | <.001 |
|  | South Western Region | .522 | 65 | <.001 | .385 | 65 | <.001 |
|  | Western Region | .506 | 87 | <.001 | .418 | 87 | <.001 |
|  | Gozo and Comino | .470 | 13 | <.001 | .533 | 13 | <.001 |
| On the scale shown, how much do you agree the following actions would help address the problem of antibiotic resistance? [Doctors should only prescribe antibiotics when they are needed ] | Northern Region | .526 | 98 | <.001 | .229 | 98 | <.001 |
|  | Northern Harbour Region | .535 | 171 | <.001 | .229 | 171 | <.001 |
|  | Southern Harbour Region | .506 | 42 | <.001 | .431 | 42 | <.001 |
|  | South Western Region | .539 | 65 | <.001 | .258 | 65 | <.001 |
|  | Western Region | .521 | 87 | <.001 | .284 | 87 | <.001 |
|  | Gozo and Comino | .532 | 13 | <.001 | .311 | 13 | <.001 |
| On the scale shown, how much do you agree the following actions would help address the problem of antibiotic resistance? [Governments should reward the development of new antibiotics] | Northern Region | .268 | 98 | <.001 | .821 | 98 | <.001 |
|  | Northern Harbour Region | .269 | 171 | <.001 | .794 | 171 | <.001 |
|  | Southern Harbour Region | .283 | 42 | <.001 | .798 | 42 | <.001 |
|  | South Western Region | .217 | 65 | <.001 | .822 | 65 | <.001 |
|  | Western Region | .260 | 87 | <.001 | .810 | 87 | <.001 |
|  | Gozo and Comino | .375 | 13 | <.001 | .719 | 13 | <.001 |
| On the scale shown, how much do you agree the following actions would help address the problem of antibiotic resistance? [Pharmaceutical companies should develop new antibiotics] | Northern Region | .192 | 98 | <.001 | .850 | 98 | <.001 |
|  | Northern Harbour Region | .268 | 171 | <.001 | .813 | 171 | <.001 |
|  | Southern Harbour Region | .239 | 42 | <.001 | .809 | 42 | <.001 |
|  | South Western Region | .220 | 65 | <.001 | .845 | 65 | <.001 |
|  | Western Region | .298 | 87 | <.001 | .784 | 87 | <.001 |
|  | Gozo and Comino | .243 | 13 | .034 | .777 | 13 | .004 |
| On the scale shown, how much do you agree with the following statements? [Antibiotic resistance is one of the biggest problems the world faces] | Northern Region | .208 | 98 | <.001 | .888 | 98 | <.001 |
|  | Northern Harbour Region | .185 | 171 | <.001 | .881 | 171 | <.001 |
|  | Southern Harbour Region | .182 | 42 | .001 | .897 | 42 | .001 |
|  | South Western Region | .240 | 65 | <.001 | .821 | 65 | <.001 |
|  | Western Region | .200 | 87 | <.001 | .873 | 87 | <.001 |
|  | Gozo and Comino | .266 | 13 | .012 | .889 | 13 | .095 |
| On the scale shown, how much do you agree with the following statements? [Medical experts will solve the problem of antibiotic resistance before it becomes too serious] | Northern Region | .261 | 98 | <.001 | .873 | 98 | <.001 |
|  | Northern Harbour Region | .225 | 171 | <.001 | .900 | 171 | <.001 |
|  | Southern Harbour Region | .235 | 42 | <.001 | .902 | 42 | .002 |
|  | South Western Region | .232 | 65 | <.001 | .896 | 65 | <.001 |
|  | Western Region | .218 | 87 | <.001 | .909 | 87 | <.001 |
|  | Gozo and Comino | .276 | 13 | .008 | .882 | 13 | .077 |
| On the scale shown, how much do you agree with the following statements? [Everyone needs to take responsibility for using antibiotics responsibly] | Northern Region | .484 | 98 | <.001 | .504 | 98 | <.001 |
|  | Northern Harbour Region | .508 | 171 | <.001 | .393 | 171 | <.001 |
|  | Southern Harbour Region | .434 | 42 | <.001 | .567 | 42 | <.001 |
|  | South Western Region | .521 | 65 | <.001 | .365 | 65 | <.001 |
|  | Western Region | .493 | 87 | <.001 | .453 | 87 | <.001 |
|  | Gozo and Comino | .532 | 13 | <.001 | .311 | 13 | <.001 |
| On the scale shown, how much do you agree with the following statements? [There is not much people like me can do to stop antibiotic resistance] | Northern Region | .195 | 98 | <.001 | .896 | 98 | <.001 |
|  | Northern Harbour Region | .188 | 171 | <.001 | .904 | 171 | <.001 |
|  | Southern Harbour Region | .176 | 42 | .002 | .897 | 42 | .001 |
|  | South Western Region | .157 | 65 | <.001 | .915 | 65 | <.001 |
|  | Western Region | .232 | 87 | <.001 | .887 | 87 | <.001 |
|  | Gozo and Comino | .194 | 13 | .195 | .935 | 13 | .390 |
| On the scale shown, how much do you agree with the following statements? [I am worried about the impact that antibiotic resistance will have on my health, and that of my family] | Northern Region | .196 | 98 | <.001 | .872 | 98 | <.001 |
|  | Northern Harbour Region | .234 | 171 | <.001 | .850 | 171 | <.001 |
|  | Southern Harbour Region | .217 | 42 | <.001 | .846 | 42 | <.001 |
|  | South Western Region | .242 | 65 | <.001 | .847 | 65 | <.001 |
|  | Western Region | .234 | 87 | <.001 | .823 | 87 | <.001 |
|  | Gozo and Comino | .289 | 13 | .004 | .813 | 13 | .010 |
| On the scale shown, how much do you agree with the following statements? [I am not at risk of getting an antibiotic-resistant infection, as long as I take my antibiotics correctly] | Northern Region | .149 | 98 | <.001 | .895 | 98 | <.001 |
|  | Northern Harbour Region | .166 | 171 | <.001 | .891 | 171 | <.001 |
|  | Southern Harbour Region | .258 | 42 | <.001 | .857 | 42 | <.001 |
|  | South Western Region | .168 | 65 | <.001 | .901 | 65 | <.001 |
|  | Western Region | .156 | 87 | <.001 | .896 | 87 | <.001 |
|  | Gozo and Comino | .272 | 13 | .009 | .890 | 13 | .097 |
| a. Lilliefors Significance Correction | | | | | | | |

(6) AGE DIFFERENCES ON LIKERT SCALE

**On the scale shown, how much do you agree the following actions would help address the problem of antibiotic resistance? [People should use antibiotics only when they are prescribed by a doctor or nurse] across AgeGroup**

| **Independent-Samples Kruskal-Wallis Test Summary** | |
| --- | --- |
| Total N | 476 |
| Test Statistic | 17.750^a^ |
| Degree Of Freedom | 2 |
| Asymptotic Sig.(2-sided test) | <.001 |
| a. The test statistic is adjusted for ties. | |

| **Pairwise Comparisons of AgeGroup** | | | | | |
| --- | --- | --- | --- | --- | --- |
| Sample 1-Sample 2 | Test Statistic | Std. Error | Std. Test Statistic | Sig. | Adj. Sig.^a^ |
| 18-34-35-54 | -26.851 | 7.364 | -3.646 | <.001 | .001 |
| 18-34-55+ | -33.290 | 10.542 | -3.158 | .002 | .005 |
| 35-54-55+ | -6.439 | 10.777 | -.598 | .550 | 1.000 |
| Each row tests the null hypothesis that the Sample 1 and Sample 2 distributions are the same.  Asymptotic significances (2-sided tests) are displayed. The significance level is .050. | | | | | |
| a. Significance values have been adjusted by the Bonferroni correction for multiple tests. | | | | | |

**On the scale shown, how much do you agree the following actions would help address the problem of antibiotic resistance? [Farmers should give fewer antibiotics to food-producing animals] across AgeGroup**

| **Independent-Samples Kruskal-Wallis Test Summary** | |
| --- | --- |
| Total N | 476 |
| Test Statistic | 39.619^a^ |
| Degree Of Freedom | 2 |
| Asymptotic Sig.(2-sided test) | <.001 |
| a. The test statistic is adjusted for ties. | |

| **Pairwise Comparisons of AgeGroup** | | | | | |
| --- | --- | --- | --- | --- | --- |
| Sample 1-Sample 2 | Test Statistic | Std. Error | Std. Test Statistic | Sig. | Adj. Sig.^a^ |
| 18-34-35-54 | -57.766 | 11.574 | -4.991 | <.001 | .000 |
| 18-34-55+ | -86.486 | 16.569 | -5.220 | <.001 | .000 |
| 35-54-55+ | -28.720 | 16.938 | -1.696 | .090 | .270 |
| Each row tests the null hypothesis that the Sample 1 and Sample 2 distributions are the same.  Asymptotic significances (2-sided tests) are displayed. The significance level is .050. | | | | | |
| a. Significance values have been adjusted by the Bonferroni correction for multiple tests. | | | | | |

**On the scale shown, how much do you agree the following actions would help address the problem of antibiotic resistance? [People should not keep antibiotics and use them later for other illnesses] across AgeGroup**

| **Independent-Samples Kruskal-Wallis Test Summary** | |
| --- | --- |
| Total N | 476 |
| Test Statistic | 15.423^a^ |
| Degree Of Freedom | 2 |
| Asymptotic Sig.(2-sided test) | <.001 |
| a. The test statistic is adjusted for ties. | |

| **Pairwise Comparisons of AgeGroup** | | | | | |
| --- | --- | --- | --- | --- | --- |
| Sample 1-Sample 2 | Test Statistic | Std. Error | Std. Test Statistic | Sig. | Adj. Sig.^a^ |
| 18-34-35-54 | -37.267 | 11.215 | -3.323 | <.001 | .003 |
| 18-34-55+ | -48.786 | 16.056 | -3.039 | .002 | .007 |
| 35-54-55+ | -11.519 | 16.413 | -.702 | .483 | 1.000 |
| Each row tests the null hypothesis that the Sample 1 and Sample 2 distributions are the same.  Asymptotic significances (2-sided tests) are displayed. The significance level is .050. | | | | | |
| a. Significance values have been adjusted by the Bonferroni correction for multiple tests. | | | | | |

**On the scale shown, how much do you agree the following actions would help address the problem of antibiotic resistance? [Parents should make sure all of their children’s vaccinations are up-to-date] across AgeGroup**

| **Independent-Samples Kruskal-Wallis Test Summary** | |
| --- | --- |
| Total N | 476 |
| Test Statistic | 10.624^a^ |
| Degree Of Freedom | 2 |
| Asymptotic Sig.(2-sided test) | .005 |
| a. The test statistic is adjusted for ties. | |

| **Pairwise Comparisons of AgeGroup** | | | | | |
| --- | --- | --- | --- | --- | --- |
| Sample 1-Sample 2 | Test Statistic | Std. Error | Std. Test Statistic | Sig. | Adj. Sig.^a^ |
| 18-34-35-54 | -10.206 | 8.063 | -1.266 | .206 | .617 |
| 18-34-55+ | -37.514 | 11.543 | -3.250 | .001 | .003 |
| 35-54-55+ | -27.309 | 11.800 | -2.314 | .021 | .062 |
| Each row tests the null hypothesis that the Sample 1 and Sample 2 distributions are the same.  Asymptotic significances (2-sided tests) are displayed. The significance level is .050. | | | | | |
| a. Significance values have been adjusted by the Bonferroni correction for multiple tests. | | | | | |

**On the scale shown, how much do you agree the following actions would help address the problem of antibiotic resistance? [People should wash their hands regularly] across AgeGroup**

| **Independent-Samples Kruskal-Wallis Test Summary** | |
| --- | --- |
| Total N | 476 |
| Test Statistic | 8.824^a^ |
| Degree Of Freedom | 2 |
| Asymptotic Sig.(2-sided test) | .012 |
| a. The test statistic is adjusted for ties. | |

| **Pairwise Comparisons of AgeGroup** | | | | | |
| --- | --- | --- | --- | --- | --- |
| Sample 1-Sample 2 | Test Statistic | Std. Error | Std. Test Statistic | Sig. | Adj. Sig.^a^ |
| 18-34-35-54 | -19.161 | 7.899 | -2.426 | .015 | .046 |
| 18-34-55+ | -27.083 | 11.308 | -2.395 | .017 | .050 |
| 35-54-55+ | -7.922 | 11.560 | -.685 | .493 | 1.000 |
| Each row tests the null hypothesis that the Sample 1 and Sample 2 distributions are the same.  Asymptotic significances (2-sided tests) are displayed. The significance level is .050. | | | | | |
| a. Significance values have been adjusted by the Bonferroni correction for multiple tests. | | | | | |

**On the scale shown, how much do you agree the following actions would help address the problem of antibiotic resistance? [Doctors should only prescribe antibiotics when they are needed ] across AgeGroup**

| **Independent-Samples Kruskal-Wallis Test Summary** | |
| --- | --- |
| Total N | 476 |
| Test Statistic | 11.751^a^ |
| Degree Of Freedom | 2 |
| Asymptotic Sig.(2-sided test) | .003 |
| a. The test statistic is adjusted for ties. | |

| **Pairwise Comparisons of AgeGroup** | | | | | |
| --- | --- | --- | --- | --- | --- |
| Sample 1-Sample 2 | Test Statistic | Std. Error | Std. Test Statistic | Sig. | Adj. Sig.^a^ |
| 18-34-55+ | -15.465 | 8.578 | -1.803 | .071 | .214 |
| 18-34-35-54 | -19.992 | 5.991 | -3.337 | <.001 | .003 |
| 55+-35-54 | 4.528 | 8.768 | .516 | .606 | 1.000 |
| Each row tests the null hypothesis that the Sample 1 and Sample 2 distributions are the same.  Asymptotic significances (2-sided tests) are displayed. The significance level is .050. | | | | | |
| a. Significance values have been adjusted by the Bonferroni correction for multiple tests. | | | | | |

**On the scale shown, how much do you agree the following actions would help address the problem of antibiotic resistance? [Governments should reward the development of new antibiotics] across AgeGroup**

| **Independent-Samples Kruskal-Wallis Test Summary** | |
| --- | --- |
| Total N | 476 |
| Test Statistic | 5.858^a^ |
| Degree Of Freedom | 2 |
| Asymptotic Sig.(2-sided test) | .053 |
| a. The test statistic is adjusted for ties. | |

| **Pairwise Comparisons of AgeGroup** | | | | | |
| --- | --- | --- | --- | --- | --- |
| Sample 1-Sample 2 | Test Statistic | Std. Error | Std. Test Statistic | Sig. | Adj. Sig.^a^ |
| 18-34-35-54 | -7.576 | 12.808 | -.592 | .554 | 1.000 |
| 18-34-55+ | -44.255 | 18.337 | -2.413 | .016 | .047 |
| 35-54-55+ | -36.678 | 18.745 | -1.957 | .050 | .151 |
| Each row tests the null hypothesis that the Sample 1 and Sample 2 distributions are the same.  Asymptotic significances (2-sided tests) are displayed. The significance level is .050. | | | | | |
| a. Significance values have been adjusted by the Bonferroni correction for multiple tests. | | | | | |

**On the scale shown, how much do you agree the following actions would help address the problem of antibiotic resistance? [Pharmaceutical companies should develop new antibiotics] across AgeGroup**

| **Independent-Samples Kruskal-Wallis Test Summary** | |
| --- | --- |
| Total N | 476 |
| Test Statistic | 17.249^a^ |
| Degree Of Freedom | 2 |
| Asymptotic Sig.(2-sided test) | <.001 |
| a. The test statistic is adjusted for ties. | |

| **Pairwise Comparisons of AgeGroup** | | | | | |
| --- | --- | --- | --- | --- | --- |
| Sample 1-Sample 2 | Test Statistic | Std. Error | Std. Test Statistic | Sig. | Adj. Sig.^a^ |
| 18-34-35-54 | -31.735 | 12.851 | -2.470 | .014 | .041 |
| 18-34-55+ | -72.667 | 18.397 | -3.950 | <.001 | .000 |
| 35-54-55+ | -40.932 | 18.807 | -2.176 | .030 | .089 |
| Each row tests the null hypothesis that the Sample 1 and Sample 2 distributions are the same.  Asymptotic significances (2-sided tests) are displayed. The significance level is .050. | | | | | |
| a. Significance values have been adjusted by the Bonferroni correction for multiple tests. | | | | | |

**On the scale shown, how much do you agree with the following statements? [Antibiotic resistance is one of the biggest problems the world faces] across AgeGroup**

| **Independent-Samples Kruskal-Wallis Test Summary** | |
| --- | --- |
| Total N | 476 |
| Test Statistic | 15.246^a^ |
| Degree Of Freedom | 2 |
| Asymptotic Sig.(2-sided test) | <.001 |
| a. The test statistic is adjusted for ties. | |

| **Pairwise Comparisons of AgeGroup** | | | | | |
| --- | --- | --- | --- | --- | --- |
| Sample 1-Sample 2 | Test Statistic | Std. Error | Std. Test Statistic | Sig. | Adj. Sig.^a^ |
| 18-34-35-54 | -42.325 | 13.098 | -3.232 | .001 | .004 |
| 18-34-55+ | -58.185 | 18.751 | -3.103 | .002 | .006 |
| 35-54-55+ | -15.860 | 19.168 | -.827 | .408 | 1.000 |
| Each row tests the null hypothesis that the Sample 1 and Sample 2 distributions are the same.  Asymptotic significances (2-sided tests) are displayed. The significance level is .050. | | | | | |
| a. Significance values have been adjusted by the Bonferroni correction for multiple tests. | | | | | |

**On the scale shown, how much do you agree with the following statements? [Medical experts will solve the problem of antibiotic resistance before it becomes too serious] across AgeGroup**

| **Independent-Samples Kruskal-Wallis Test Summary** | |
| --- | --- |
| Total N | 476 |
| Test Statistic | 9.873^a^ |
| Degree Of Freedom | 2 |
| Asymptotic Sig.(2-sided test) | .007 |
| a. The test statistic is adjusted for ties. | |

| **Pairwise Comparisons of AgeGroup** | | | | | |
| --- | --- | --- | --- | --- | --- |
| Sample 1-Sample 2 | Test Statistic | Std. Error | Std. Test Statistic | Sig. | Adj. Sig.^a^ |
| 35-54-18-34 | .187 | 12.855 | .015 | .988 | 1.000 |
| 35-54-55+ | -54.945 | 18.813 | -2.921 | .003 | .010 |
| 18-34-55+ | -54.758 | 18.404 | -2.975 | .003 | .009 |
| Each row tests the null hypothesis that the Sample 1 and Sample 2 distributions are the same.  Asymptotic significances (2-sided tests) are displayed. The significance level is .050. | | | | | |
| a. Significance values have been adjusted by the Bonferroni correction for multiple tests. | | | | | |

**On the scale shown, how much do you agree with the following statements? [Everyone needs to take responsibility for using antibiotics responsibly] across AgeGroup**

| **Independent-Samples Kruskal-Wallis Test Summary** | |
| --- | --- |
| Total N | 476 |
| Test Statistic | 5.354^a^ |
| Degree Of Freedom | 2 |
| Asymptotic Sig.(2-sided test) | .069 |
| a. The test statistic is adjusted for ties. | |

| **Pairwise Comparisons of AgeGroup** | | | | | |
| --- | --- | --- | --- | --- | --- |
| Sample 1-Sample 2 | Test Statistic | Std. Error | Std. Test Statistic | Sig. | Adj. Sig.^a^ |
| 18-34-35-54 | -17.389 | 8.564 | -2.031 | .042 | .127 |
| 18-34-55+ | -20.791 | 12.260 | -1.696 | .090 | .270 |
| 35-54-55+ | -3.402 | 12.533 | -.271 | .786 | 1.000 |
| Each row tests the null hypothesis that the Sample 1 and Sample 2 distributions are the same.  Asymptotic significances (2-sided tests) are displayed. The significance level is .050. | | | | | |
| a. Significance values have been adjusted by the Bonferroni correction for multiple tests. | | | | | |

**On the scale shown, how much do you agree with the following statements? [There is not much people like me can do to stop antibiotic resistance] across AgeGroup**

| **Independent-Samples Kruskal-Wallis Test Summary** | |
| --- | --- |
| Total N | 476 |
| Test Statistic | 4.303^a^ |
| Degree Of Freedom | 2 |
| Asymptotic Sig.(2-sided test) | .116 |
| a. The test statistic is adjusted for ties. | |

| **Pairwise Comparisons of AgeGroup** | | | | | |
| --- | --- | --- | --- | --- | --- |
| Sample 1-Sample 2 | Test Statistic | Std. Error | Std. Test Statistic | Sig. | Adj. Sig.^a^ |
| 35-54-18-34 | 13.603 | 13.257 | 1.026 | .305 | .915 |
| 35-54-55+ | -39.877 | 19.401 | -2.055 | .040 | .120 |
| 18-34-55+ | -26.275 | 18.979 | -1.384 | .166 | .499 |
| Each row tests the null hypothesis that the Sample 1 and Sample 2 distributions are the same.  Asymptotic significances (2-sided tests) are displayed. The significance level is .050. | | | | | |
| a. Significance values have been adjusted by the Bonferroni correction for multiple tests. | | | | | |

**On the scale shown, how much do you agree with the following statements? [I am worried about the impact that antibiotic resistance will have on my health, and that of my family] across AgeGroup**

| **Independent-Samples Kruskal-Wallis Test Summary** | |
| --- | --- |
| Total N | 476 |
| Test Statistic | 5.221^a^ |
| Degree Of Freedom | 2 |
| Asymptotic Sig.(2-sided test) | .074 |
| a. The test statistic is adjusted for ties. | |

| **Pairwise Comparisons of AgeGroup** | | | | | |
| --- | --- | --- | --- | --- | --- |
| Sample 1-Sample 2 | Test Statistic | Std. Error | Std. Test Statistic | Sig. | Adj. Sig.^a^ |
| 18-34-35-54 | -19.506 | 12.999 | -1.501 | .133 | .400 |
| 18-34-55+ | -39.263 | 18.609 | -2.110 | .035 | .105 |
| 35-54-55+ | -19.756 | 19.023 | -1.039 | .299 | .897 |
| Each row tests the null hypothesis that the Sample 1 and Sample 2 distributions are the same.  Asymptotic significances (2-sided tests) are displayed. The significance level is .050. | | | | | |
| a. Significance values have been adjusted by the Bonferroni correction for multiple tests. | | | | | |

**On the scale shown, how much do you agree with the following statements? [I am not at risk of getting an antibiotic-resistant infection, as long as I take my antibiotics correctly] across AgeGroup**

| **Independent-Samples Kruskal-Wallis Test Summary** | |
| --- | --- |
| Total N | 476 |
| Test Statistic | 2.639^a^ |
| Degree Of Freedom | 2 |
| Asymptotic Sig.(2-sided test) | .267 |
| a. The test statistic is adjusted for ties. | |

| **Pairwise Comparisons of AgeGroup** | | | | | |
| --- | --- | --- | --- | --- | --- |
| Sample 1-Sample 2 | Test Statistic | Std. Error | Std. Test Statistic | Sig. | Adj. Sig.^a^ |
| 18-34-35-54 | -10.819 | 13.299 | -.814 | .416 | 1.000 |
| 18-34-55+ | -30.301 | 19.039 | -1.591 | .111 | .334 |
| 35-54-55+ | -19.482 | 19.463 | -1.001 | .317 | .951 |
| Each row tests the null hypothesis that the Sample 1 and Sample 2 distributions are the same.  Asymptotic significances (2-sided tests) are displayed. The significance level is .050. | | | | | |
| a. Significance values have been adjusted by the Bonferroni correction for multiple tests. | | | | | |

(7) EDUCATION DIFFERENCES ON LIKERT SCALE

**On the scale shown, how much do you agree the following actions would help address the problem of antibiotic resistance? [People should use antibiotics only when they are prescribed by a doctor or nurse] across EducationLevel**

| **Independent-Samples Kruskal-Wallis Test Summary** | |
| --- | --- |
| Total N | 476 |
| Test Statistic | 6.222^a^ |
| Degree Of Freedom | 2 |
| Asymptotic Sig.(2-sided test) | .045 |
| a. The test statistic is adjusted for ties. | |

| **Pairwise Comparisons of EducationLevel** | | | | | |
| --- | --- | --- | --- | --- | --- |
| Sample 1-Sample 2 | Test Statistic | Std. Error | Std. Test Statistic | Sig. | Adj. Sig.^a^ |
| Low Education-High Education | -15.266 | 8.975 | -1.701 | .089 | .267 |
| Low Education-Medium Education | -23.949 | 9.611 | -2.492 | .013 | .038 |
| High Education-Medium Education | 8.683 | 7.813 | 1.111 | .266 | .799 |
| Each row tests the null hypothesis that the Sample 1 and Sample 2 distributions are the same.  Asymptotic significances (2-sided tests) are displayed. The significance level is .050. | | | | | |
| a. Significance values have been adjusted by the Bonferroni correction for multiple tests. | | | | | |

**On the scale shown, how much do you agree the following actions would help address the problem of antibiotic resistance? [Farmers should give fewer antibiotics to food-producing animals] across EducationLevel**

| **Independent-Samples Kruskal-Wallis Test Summary** | |
| --- | --- |
| Total N | 476 |
| Test Statistic | 5.080^a^ |
| Degree Of Freedom | 2 |
| Asymptotic Sig.(2-sided test) | .079 |
| a. The test statistic is adjusted for ties. | |

| **Pairwise Comparisons of EducationLevel** | | | | | |
| --- | --- | --- | --- | --- | --- |
| Sample 1-Sample 2 | Test Statistic | Std. Error | Std. Test Statistic | Sig. | Adj. Sig.^a^ |
| High Education-Low Education | 19.115 | 14.106 | 1.355 | .175 | .526 |
| High Education-Medium Education | 26.549 | 12.280 | 2.162 | .031 | .092 |
| Low Education-Medium Education | -7.434 | 15.106 | -.492 | .623 | 1.000 |
| Each row tests the null hypothesis that the Sample 1 and Sample 2 distributions are the same.  Asymptotic significances (2-sided tests) are displayed. The significance level is .050. | | | | | |
| a. Significance values have been adjusted by the Bonferroni correction for multiple tests. | | | | | |

**On the scale shown, how much do you agree the following actions would help address the problem of antibiotic resistance? [People should not keep antibiotics and use them later for other illnesses] across EducationLevel**

| **Independent-Samples Kruskal-Wallis Test Summary** | |
| --- | --- |
| Total N | 476 |
| Test Statistic | 2.161^a^ |
| Degree Of Freedom | 2 |
| Asymptotic Sig.(2-sided test) | .339 |
| a. The test statistic is adjusted for ties. | |

| **Pairwise Comparisons of EducationLevel** | | | | | |
| --- | --- | --- | --- | --- | --- |
| Sample 1-Sample 2 | Test Statistic | Std. Error | Std. Test Statistic | Sig. | Adj. Sig.^a^ |
| Low Education-High Education | -6.320 | 13.668 | -.462 | .644 | 1.000 |
| Low Education-Medium Education | -19.936 | 14.637 | -1.362 | .173 | .520 |
| High Education-Medium Education | 13.616 | 11.899 | 1.144 | .253 | .758 |
| Each row tests the null hypothesis that the Sample 1 and Sample 2 distributions are the same.  Asymptotic significances (2-sided tests) are displayed. The significance level is .050. | | | | | |
| a. Significance values have been adjusted by the Bonferroni correction for multiple tests. | | | | | |

**On the scale shown, how much do you agree the following actions would help address the problem of antibiotic resistance? [Parents should make sure all of their children’s vaccinations are up-to-date] across EducationLevel**

| **Independent-Samples Kruskal-Wallis Test Summary** | |
| --- | --- |
| Total N | 476 |
| Test Statistic | 2.881^a^ |
| Degree Of Freedom | 2 |
| Asymptotic Sig.(2-sided test) | .237 |
| a. The test statistic is adjusted for ties. | |

| **Pairwise Comparisons of EducationLevel** | | | | | |
| --- | --- | --- | --- | --- | --- |
| Sample 1-Sample 2 | Test Statistic | Std. Error | Std. Test Statistic | Sig. | Adj. Sig.^a^ |
| Medium Education-Low Education | 2.959 | 10.523 | .281 | .779 | 1.000 |
| Medium Education-High Education | -13.688 | 8.555 | -1.600 | .110 | .329 |
| Low Education-High Education | -10.728 | 9.827 | -1.092 | .275 | .825 |
| Each row tests the null hypothesis that the Sample 1 and Sample 2 distributions are the same.  Asymptotic significances (2-sided tests) are displayed. The significance level is .050. | | | | | |
| a. Significance values have been adjusted by the Bonferroni correction for multiple tests. | | | | | |

**On the scale shown, how much do you agree the following actions would help address the problem of antibiotic resistance? [People should wash their hands regularly] across EducationLevel**

| **Independent-Samples Kruskal-Wallis Test Summary** | |
| --- | --- |
| Total N | 476 |
| Test Statistic | 1.716^a^ |
| Degree Of Freedom | 2 |
| Asymptotic Sig.(2-sided test) | .424 |
| a. The test statistic is adjusted for ties. | |

| **Pairwise Comparisons of EducationLevel** | | | | | |
| --- | --- | --- | --- | --- | --- |
| Sample 1-Sample 2 | Test Statistic | Std. Error | Std. Test Statistic | Sig. | Adj. Sig.^a^ |
| Low Education-High Education | -.212 | 9.627 | -.022 | .982 | 1.000 |
| Low Education-Medium Education | -10.426 | 10.309 | -1.011 | .312 | .936 |
| High Education-Medium Education | 10.214 | 8.381 | 1.219 | .223 | .669 |
| Each row tests the null hypothesis that the Sample 1 and Sample 2 distributions are the same.  Asymptotic significances (2-sided tests) are displayed. The significance level is .050. | | | | | |
| a. Significance values have been adjusted by the Bonferroni correction for multiple tests. | | | | | |

**On the scale shown, how much do you agree the following actions would help address the problem of antibiotic resistance? [Doctors should only prescribe antibiotics when they are needed ] across EducationLevel**

| **Independent-Samples Kruskal-Wallis Test Summary** | |
| --- | --- |
| Total N | 476 |
| Test Statistic | .287^a^ |
| Degree Of Freedom | 2 |
| Asymptotic Sig.(2-sided test) | .866 |
| a. The test statistic is adjusted for ties. | |

| **Pairwise Comparisons of EducationLevel** | | | | | |
| --- | --- | --- | --- | --- | --- |
| Sample 1-Sample 2 | Test Statistic | Std. Error | Std. Test Statistic | Sig. | Adj. Sig.^a^ |
| Medium Education-High Education | -2.700 | 6.357 | -.425 | .671 | 1.000 |
| Medium Education-Low Education | 3.843 | 7.820 | .491 | .623 | 1.000 |
| High Education-Low Education | 1.143 | 7.302 | .157 | .876 | 1.000 |
| Each row tests the null hypothesis that the Sample 1 and Sample 2 distributions are the same.  Asymptotic significances (2-sided tests) are displayed. The significance level is .050. | | | | | |
| a. Significance values have been adjusted by the Bonferroni correction for multiple tests. | | | | | |

**On the scale shown, how much do you agree the following actions would help address the problem of antibiotic resistance? [Governments should reward the development of new antibiotics] across EducationLevel**

| **Independent-Samples Kruskal-Wallis Test Summary** | |
| --- | --- |
| Total N | 476 |
| Test Statistic | .591^a^ |
| Degree Of Freedom | 2 |
| Asymptotic Sig.(2-sided test) | .744 |
| a. The test statistic is adjusted for ties. | |

| **Pairwise Comparisons of EducationLevel** | | | | | |
| --- | --- | --- | --- | --- | --- |
| Sample 1-Sample 2 | Test Statistic | Std. Error | Std. Test Statistic | Sig. | Adj. Sig.^a^ |
| Medium Education-High Education | -2.627 | 13.590 | -.193 | .847 | 1.000 |
| Medium Education-Low Education | 12.440 | 16.717 | .744 | .457 | 1.000 |
| High Education-Low Education | 9.813 | 15.611 | .629 | .530 | 1.000 |
| Each row tests the null hypothesis that the Sample 1 and Sample 2 distributions are the same.  Asymptotic significances (2-sided tests) are displayed. The significance level is .050. | | | | | |
| a. Significance values have been adjusted by the Bonferroni correction for multiple tests. | | | | | |

**On the scale shown, how much do you agree the following actions would help address the problem of antibiotic resistance? [Pharmaceutical companies should develop new antibiotics] across EducationLevel**

| **Independent-Samples Kruskal-Wallis Test Summary** | |
| --- | --- |
| Total N | 476 |
| Test Statistic | .727^a^ |
| Degree Of Freedom | 2 |
| Asymptotic Sig.(2-sided test) | .695 |
| a. The test statistic is adjusted for ties. | |

| **Pairwise Comparisons of EducationLevel** | | | | | |
| --- | --- | --- | --- | --- | --- |
| Sample 1-Sample 2 | Test Statistic | Std. Error | Std. Test Statistic | Sig. | Adj. Sig.^a^ |
| High Education-Low Education | 2.284 | 15.662 | .146 | .884 | 1.000 |
| High Education-Medium Education | 11.422 | 13.635 | .838 | .402 | 1.000 |
| Low Education-Medium Education | -9.138 | 16.772 | -.545 | .586 | 1.000 |
| Each row tests the null hypothesis that the Sample 1 and Sample 2 distributions are the same.  Asymptotic significances (2-sided tests) are displayed. The significance level is .050. | | | | | |
| a. Significance values have been adjusted by the Bonferroni correction for multiple tests. | | | | | |

**On the scale shown, how much do you agree with the following statements? [Antibiotic resistance is one of the biggest problems the world faces] across EducationLevel**

| **Independent-Samples Kruskal-Wallis Test Summary** | |
| --- | --- |
| Total N | 476 |
| Test Statistic | 2.279^a^ |
| Degree Of Freedom | 2 |
| Asymptotic Sig.(2-sided test) | .320 |
| a. The test statistic is adjusted for ties. | |

| **Pairwise Comparisons of EducationLevel** | | | | | |
| --- | --- | --- | --- | --- | --- |
| Sample 1-Sample 2 | Test Statistic | Std. Error | Std. Test Statistic | Sig. | Adj. Sig.^a^ |
| Low Education-Medium Education | -7.000 | 17.094 | -.410 | .682 | 1.000 |
| Low Education-High Education | -21.897 | 15.963 | -1.372 | .170 | .510 |
| Medium Education-High Education | -14.897 | 13.897 | -1.072 | .284 | .851 |
| Each row tests the null hypothesis that the Sample 1 and Sample 2 distributions are the same.  Asymptotic significances (2-sided tests) are displayed. The significance level is .050. | | | | | |
| a. Significance values have been adjusted by the Bonferroni correction for multiple tests. | | | | | |

**On the scale shown, how much do you agree with the following statements? [Medical experts will solve the problem of antibiotic resistance before it becomes too serious] across EducationLevel**

| **Independent-Samples Kruskal-Wallis Test Summary** | |
| --- | --- |
| Total N | 476 |
| Test Statistic | 9.209^a^ |
| Degree Of Freedom | 2 |
| Asymptotic Sig.(2-sided test) | .010 |
| a. The test statistic is adjusted for ties. | |

| **Pairwise Comparisons of EducationLevel** | | | | | |
| --- | --- | --- | --- | --- | --- |
| Sample 1-Sample 2 | Test Statistic | Std. Error | Std. Test Statistic | Sig. | Adj. Sig.^a^ |
| High Education-Medium Education | 21.597 | 13.640 | 1.583 | .113 | .340 |
| High Education-Low Education | 46.687 | 15.668 | 2.980 | .003 | .009 |
| Medium Education-Low Education | 25.091 | 16.778 | 1.495 | .135 | .404 |
| Each row tests the null hypothesis that the Sample 1 and Sample 2 distributions are the same.  Asymptotic significances (2-sided tests) are displayed. The significance level is .050. | | | | | |
| a. Significance values have been adjusted by the Bonferroni correction for multiple tests. | | | | | |

**On the scale shown, how much do you agree with the following statements? [Everyone needs to take responsibility for using antibiotics responsibly] across EducationLevel**

| **Independent-Samples Kruskal-Wallis Test Summary** | |
| --- | --- |
| Total N | 476 |
| Test Statistic | 1.165^a^ |
| Degree Of Freedom | 2 |
| Asymptotic Sig.(2-sided test) | .559 |
| a. The test statistic is adjusted for ties. | |

| **Pairwise Comparisons of EducationLevel** | | | | | |
| --- | --- | --- | --- | --- | --- |
| Sample 1-Sample 2 | Test Statistic | Std. Error | Std. Test Statistic | Sig. | Adj. Sig.^a^ |
| Low Education-High Education | -6.782 | 10.437 | -.650 | .516 | 1.000 |
| Low Education-Medium Education | -12.041 | 11.177 | -1.077 | .281 | .844 |
| High Education-Medium Education | 5.259 | 9.086 | .579 | .563 | 1.000 |
| Each row tests the null hypothesis that the Sample 1 and Sample 2 distributions are the same.  Asymptotic significances (2-sided tests) are displayed. The significance level is .050. | | | | | |
| a. Significance values have been adjusted by the Bonferroni correction for multiple tests. | | | | | |

**On the scale shown, how much do you agree with the following statements? [There is not much people like me can do to stop antibiotic resistance] across EducationLevel**

| **Independent-Samples Kruskal-Wallis Test Summary** | |
| --- | --- |
| Total N | 476 |
| Test Statistic | 4.133^a^ |
| Degree Of Freedom | 2 |
| Asymptotic Sig.(2-sided test) | .127 |
| a. The test statistic is adjusted for ties. | |

| **Pairwise Comparisons of EducationLevel** | | | | | |
| --- | --- | --- | --- | --- | --- |
| Sample 1-Sample 2 | Test Statistic | Std. Error | Std. Test Statistic | Sig. | Adj. Sig.^a^ |
| High Education-Medium Education | 18.972 | 14.066 | 1.349 | .177 | .532 |
| High Education-Low Education | 30.663 | 16.157 | 1.898 | .058 | .173 |
| Medium Education-Low Education | 11.691 | 17.302 | .676 | .499 | 1.000 |
| Each row tests the null hypothesis that the Sample 1 and Sample 2 distributions are the same.  Asymptotic significances (2-sided tests) are displayed. The significance level is .050. | | | | | |
| a. Significance values have been adjusted by the Bonferroni correction for multiple tests. | | | | | |

**On the scale shown, how much do you agree with the following statements? [I am worried about the impact that antibiotic resistance will have on my health, and that of my family] across EducationLevel**

| **Independent-Samples Kruskal-Wallis Test Summary** | |
| --- | --- |
| Total N | 476 |
| Test Statistic | 1.791^a^ |
| Degree Of Freedom | 2 |
| Asymptotic Sig.(2-sided test) | .408 |
| a. The test statistic is adjusted for ties. | |

| **Pairwise Comparisons of EducationLevel** | | | | | |
| --- | --- | --- | --- | --- | --- |
| Sample 1-Sample 2 | Test Statistic | Std. Error | Std. Test Statistic | Sig. | Adj. Sig.^a^ |
| Low Education-Medium Education | -18.419 | 16.965 | -1.086 | .278 | .833 |
| Low Education-High Education | -20.594 | 15.843 | -1.300 | .194 | .581 |
| Medium Education-High Education | -2.175 | 13.792 | -.158 | .875 | 1.000 |
| Each row tests the null hypothesis that the Sample 1 and Sample 2 distributions are the same.  Asymptotic significances (2-sided tests) are displayed. The significance level is .050. | | | | | |
| a. Significance values have been adjusted by the Bonferroni correction for multiple tests. | | | | | |

**On the scale shown, how much do you agree with the following statements? [I am not at risk of getting an antibiotic-resistant infection, as long as I take my antibiotics correctly] across EducationLevel**

| **Independent-Samples Kruskal-Wallis Test Summary** | |
| --- | --- |
| Total N | 476 |
| Test Statistic | 10.421^a^ |
| Degree Of Freedom | 2 |
| Asymptotic Sig.(2-sided test) | .005 |
| a. The test statistic is adjusted for ties. | |

| **Pairwise Comparisons of EducationLevel** | | | | | |
| --- | --- | --- | --- | --- | --- |
| Sample 1-Sample 2 | Test Statistic | Std. Error | Std. Test Statistic | Sig. | Adj. Sig.^a^ |
| High Education-Medium Education | 19.386 | 14.110 | 1.374 | .169 | .508 |
| High Education-Low Education | 52.154 | 16.209 | 3.218 | .001 | .004 |
| Medium Education-Low Education | 32.767 | 17.357 | 1.888 | .059 | .177 |
| Each row tests the null hypothesis that the Sample 1 and Sample 2 distributions are the same.  Asymptotic significances (2-sided tests) are displayed. The significance level is .050. | | | | | |
| a. Significance values have been adjusted by the Bonferroni correction for multiple tests. | | | | | |

(8) GENDER DIFFERENCES ON LIKERT SCALE

**On the scale shown, how much do you agree the following actions would help address the problem of antibiotic resistance? [People should use antibiotics only when they are prescribed by a doctor or nurse] across What is your gender?**

| **Independent-Samples Mann-Whitney U Test Summary** | |
| --- | --- |
| Total N | 476 |
| Mann-Whitney U | 27192.500 |
| Wilcoxon W | 67947.500 |
| Test Statistic | 27192.500 |
| Standard Error | 795.793 |
| Standardized Test Statistic | -.031 |
| Asymptotic Sig.(2-sided test) | .975 |

**On the scale shown, how much do you agree the following actions would help address the problem of antibiotic resistance? [Farmers should give fewer antibiotics to food-producing animals] across What is your gender?**

| **Independent-Samples Mann-Whitney U Test Summary** | |
| --- | --- |
| Total N | 476 |
| Mann-Whitney U | 30419.000 |
| Wilcoxon W | 71174.000 |
| Test Statistic | 30419.000 |
| Standard Error | 1250.763 |
| Standardized Test Statistic | 2.560 |
| Asymptotic Sig.(2-sided test) | .010 |

**On the scale shown, how much do you agree the following actions would help address the problem of antibiotic resistance? [People should not keep antibiotics and use them later for other illnesses] across What is your gender?**

| **Independent-Samples Mann-Whitney U Test Summary** | |
| --- | --- |
| Total N | 476 |
| Mann-Whitney U | 28756.000 |
| Wilcoxon W | 69511.000 |
| Test Statistic | 28756.000 |
| Standard Error | 1211.971 |
| Standardized Test Statistic | 1.269 |
| Asymptotic Sig.(2-sided test) | .204 |

**On the scale shown, how much do you agree the following actions would help address the problem of antibiotic resistance? [Parents should make sure all of their children’s vaccinations are up-to-date] across What is your gender?**

| **Independent-Samples Mann-Whitney U Test Summary** | |
| --- | --- |
| Total N | 476 |
| Mann-Whitney U | 28456.000 |
| Wilcoxon W | 69211.000 |
| Test Statistic | 28456.000 |
| Standard Error | 871.340 |
| Standardized Test Statistic | 1.421 |
| Asymptotic Sig.(2-sided test) | .155 |

**On the scale shown, how much do you agree the following actions would help address the problem of antibiotic resistance? [People should wash their hands regularly] across What is your gender?**

| **Independent-Samples Mann-Whitney U Test Summary** | |
| --- | --- |
| Total N | 476 |
| Mann-Whitney U | 27496.500 |
| Wilcoxon W | 68251.500 |
| Test Statistic | 27496.500 |
| Standard Error | 853.597 |
| Standardized Test Statistic | .327 |
| Asymptotic Sig.(2-sided test) | .744 |

**On the scale shown, how much do you agree the following actions would help address the problem of antibiotic resistance? [Doctors should only prescribe antibiotics when they are needed ] across What is your gender?**

| **Independent-Samples Mann-Whitney U Test Summary** | |
| --- | --- |
| Total N | 476 |
| Mann-Whitney U | 27646.000 |
| Wilcoxon W | 68401.000 |
| Test Statistic | 27646.000 |
| Standard Error | 647.486 |
| Standardized Test Statistic | .662 |
| Asymptotic Sig.(2-sided test) | .508 |

**On the scale shown, how much do you agree the following actions would help address the problem of antibiotic resistance? [Governments should reward the development of new antibiotics] across What is your gender?**

| **Independent-Samples Mann-Whitney U Test Summary** | |
| --- | --- |
| Total N | 476 |
| Mann-Whitney U | 23684.500 |
| Wilcoxon W | 64439.500 |
| Test Statistic | 23684.500 |
| Standard Error | 1384.169 |
| Standardized Test Statistic | -2.552 |
| Asymptotic Sig.(2-sided test) | .011 |

**On the scale shown, how much do you agree the following actions would help address the problem of antibiotic resistance? [Pharmaceutical companies should develop new antibiotics] across What is your gender?**

| **Independent-Samples Mann-Whitney U Test Summary** | |
| --- | --- |
| Total N | 476 |
| Mann-Whitney U | 23835.000 |
| Wilcoxon W | 64590.000 |
| Test Statistic | 23835.000 |
| Standard Error | 1388.746 |
| Standardized Test Statistic | -2.436 |
| Asymptotic Sig.(2-sided test) | .015 |

**On the scale shown, how much do you agree with the following statements? [Antibiotic resistance is one of the biggest problems the world faces] across What is your gender?**

| **Independent-Samples Mann-Whitney U Test Summary** | |
| --- | --- |
| Total N | 476 |
| Mann-Whitney U | 29102.500 |
| Wilcoxon W | 69857.500 |
| Test Statistic | 29102.500 |
| Standard Error | 1415.429 |
| Standardized Test Statistic | 1.332 |
| Asymptotic Sig.(2-sided test) | .183 |

**On the scale shown, how much do you agree with the following statements? [Medical experts will solve the problem of antibiotic resistance before it becomes too serious] across What is your gender?**

| **Independent-Samples Mann-Whitney U Test Summary** | |
| --- | --- |
| Total N | 476 |
| Mann-Whitney U | 26435.500 |
| Wilcoxon W | 67190.500 |
| Test Statistic | 26435.500 |
| Standard Error | 1389.242 |
| Standardized Test Statistic | -.563 |
| Asymptotic Sig.(2-sided test) | .574 |

**On the scale shown, how much do you agree with the following statements? [Everyone needs to take responsibility for using antibiotics responsibly] across What is your gender?**

| **Independent-Samples Mann-Whitney U Test Summary** | |
| --- | --- |
| Total N | 476 |
| Mann-Whitney U | 27660.500 |
| Wilcoxon W | 68415.500 |
| Test Statistic | 27660.500 |
| Standard Error | 925.446 |
| Standardized Test Statistic | .479 |
| Asymptotic Sig.(2-sided test) | .632 |

**On the scale shown, how much do you agree with the following statements? [There is not much people like me can do to stop antibiotic resistance] across What is your gender?**

| **Independent-Samples Mann-Whitney U Test Summary** | |
| --- | --- |
| Total N | 476 |
| Mann-Whitney U | 26072.500 |
| Wilcoxon W | 66827.500 |
| Test Statistic | 26072.500 |
| Standard Error | 1432.663 |
| Standardized Test Statistic | -.799 |
| Asymptotic Sig.(2-sided test) | .424 |

**On the scale shown, how much do you agree with the following statements? [I am worried about the impact that antibiotic resistance will have on my health, and that of my family] across What is your gender?**

| **Independent-Samples Mann-Whitney U Test Summary** | |
| --- | --- |
| Total N | 476 |
| Mann-Whitney U | 28773.000 |
| Wilcoxon W | 69528.000 |
| Test Statistic | 28773.000 |
| Standard Error | 1404.744 |
| Standardized Test Statistic | 1.107 |
| Asymptotic Sig.(2-sided test) | .268 |

**On the scale shown, how much do you agree with the following statements? [I am not at risk of getting an antibiotic-resistant infection, as long as I take my antibiotics correctly] across What is your gender?**

| **Independent-Samples Mann-Whitney U Test Summary** | |
| --- | --- |
| Total N | 476 |
| Mann-Whitney U | 28778.500 |
| Wilcoxon W | 69533.500 |
| Test Statistic | 28778.500 |
| Standard Error | 1437.210 |
| Standardized Test Statistic | 1.086 |
| Asymptotic Sig.(2-sided test) | .277 |

(9) HOUSEHOLD INCOME DIFFERENCES ON LIKERT SCALE

**On the scale shown, how much do you agree the following actions would help address the problem of antibiotic resistance? [People should use antibiotics only when they are prescribed by a doctor or nurse] across What is your gender?**

| **Independent-Samples Mann-Whitney U Test Summary** | |
| --- | --- |
| Total N | 476 |
| Mann-Whitney U | 27192.500 |
| Wilcoxon W | 67947.500 |
| Test Statistic | 27192.500 |
| Standard Error | 795.793 |
| Standardized Test Statistic | -.031 |
| Asymptotic Sig.(2-sided test) | .975 |

**On the scale shown, how much do you agree the following actions would help address the problem of antibiotic resistance? [Farmers should give fewer antibiotics to food-producing animals] across What is your gender?**

| **Independent-Samples Mann-Whitney U Test Summary** | |
| --- | --- |
| Total N | 476 |
| Mann-Whitney U | 30419.000 |
| Wilcoxon W | 71174.000 |
| Test Statistic | 30419.000 |
| Standard Error | 1250.763 |
| Standardized Test Statistic | 2.560 |
| Asymptotic Sig.(2-sided test) | .010 |

**On the scale shown, how much do you agree the following actions would help address the problem of antibiotic resistance? [People should not keep antibiotics and use them later for other illnesses] across What is your gender?**

| **Independent-Samples Mann-Whitney U Test Summary** | |
| --- | --- |
| Total N | 476 |
| Mann-Whitney U | 28756.000 |
| Wilcoxon W | 69511.000 |
| Test Statistic | 28756.000 |
| Standard Error | 1211.971 |
| Standardized Test Statistic | 1.269 |
| Asymptotic Sig.(2-sided test) | .204 |

**On the scale shown, how much do you agree the following actions would help address the problem of antibiotic resistance? [Parents should make sure all of their children’s vaccinations are up-to-date] across What is your gender?**

| **Independent-Samples Mann-Whitney U Test Summary** | |
| --- | --- |
| Total N | 476 |
| Mann-Whitney U | 28456.000 |
| Wilcoxon W | 69211.000 |
| Test Statistic | 28456.000 |
| Standard Error | 871.340 |
| Standardized Test Statistic | 1.421 |
| Asymptotic Sig.(2-sided test) | .155 |

**On the scale shown, how much do you agree the following actions would help address the problem of antibiotic resistance? [People should wash their hands regularly] across What is your gender?**

| **Independent-Samples Mann-Whitney U Test Summary** | |
| --- | --- |
| Total N | 476 |
| Mann-Whitney U | 27496.500 |
| Wilcoxon W | 68251.500 |
| Test Statistic | 27496.500 |
| Standard Error | 853.597 |
| Standardized Test Statistic | .327 |
| Asymptotic Sig.(2-sided test) | .744 |

**On the scale shown, how much do you agree the following actions would help address the problem of antibiotic resistance? [Doctors should only prescribe antibiotics when they are needed ] across What is your gender?**

| **Independent-Samples Mann-Whitney U Test Summary** | |
| --- | --- |
| Total N | 476 |
| Mann-Whitney U | 27646.000 |
| Wilcoxon W | 68401.000 |
| Test Statistic | 27646.000 |
| Standard Error | 647.486 |
| Standardized Test Statistic | .662 |
| Asymptotic Sig.(2-sided test) | .508 |

**On the scale shown, how much do you agree the following actions would help address the problem of antibiotic resistance? [Governments should reward the development of new antibiotics] across What is your gender?**

| **Independent-Samples Mann-Whitney U Test Summary** | |
| --- | --- |
| Total N | 476 |
| Mann-Whitney U | 23684.500 |
| Wilcoxon W | 64439.500 |
| Test Statistic | 23684.500 |
| Standard Error | 1384.169 |
| Standardized Test Statistic | -2.552 |
| Asymptotic Sig.(2-sided test) | .011 |

**On the scale shown, how much do you agree the following actions would help address the problem of antibiotic resistance? [Pharmaceutical companies should develop new antibiotics] across What is your gender?**

| **Independent-Samples Mann-Whitney U Test Summary** | |
| --- | --- |
| Total N | 476 |
| Mann-Whitney U | 23835.000 |
| Wilcoxon W | 64590.000 |
| Test Statistic | 23835.000 |
| Standard Error | 1388.746 |
| Standardized Test Statistic | -2.436 |
| Asymptotic Sig.(2-sided test) | .015 |

**On the scale shown, how much do you agree with the following statements? [Antibiotic resistance is one of the biggest problems the world faces] across What is your gender?**

| **Independent-Samples Mann-Whitney U Test Summary** | |
| --- | --- |
| Total N | 476 |
| Mann-Whitney U | 29102.500 |
| Wilcoxon W | 69857.500 |
| Test Statistic | 29102.500 |
| Standard Error | 1415.429 |
| Standardized Test Statistic | 1.332 |
| Asymptotic Sig.(2-sided test) | .183 |

**On the scale shown, how much do you agree with the following statements? [Medical experts will solve the problem of antibiotic resistance before it becomes too serious] across What is your gender?**

| **Independent-Samples Mann-Whitney U Test Summary** | |
| --- | --- |
| Total N | 476 |
| Mann-Whitney U | 26435.500 |
| Wilcoxon W | 67190.500 |
| Test Statistic | 26435.500 |
| Standard Error | 1389.242 |
| Standardized Test Statistic | -.563 |
| Asymptotic Sig.(2-sided test) | .574 |

**On the scale shown, how much do you agree with the following statements? [Everyone needs to take responsibility for using antibiotics responsibly] across What is your gender?**

| **Independent-Samples Mann-Whitney U Test Summary** | |
| --- | --- |
| Total N | 476 |
| Mann-Whitney U | 27660.500 |
| Wilcoxon W | 68415.500 |
| Test Statistic | 27660.500 |
| Standard Error | 925.446 |
| Standardized Test Statistic | .479 |
| Asymptotic Sig.(2-sided test) | .632 |

**On the scale shown, how much do you agree with the following statements? [There is not much people like me can do to stop antibiotic resistance] across What is your gender?**

| **Independent-Samples Mann-Whitney U Test Summary** | |
| --- | --- |
| Total N | 476 |
| Mann-Whitney U | 26072.500 |
| Wilcoxon W | 66827.500 |
| Test Statistic | 26072.500 |
| Standard Error | 1432.663 |
| Standardized Test Statistic | -.799 |
| Asymptotic Sig.(2-sided test) | .424 |

**On the scale shown, how much do you agree with the following statements? [I am worried about the impact that antibiotic resistance will have on my health, and that of my family] across What is your gender?**

| **Independent-Samples Mann-Whitney U Test Summary** | |
| --- | --- |
| Total N | 476 |
| Mann-Whitney U | 28773.000 |
| Wilcoxon W | 69528.000 |
| Test Statistic | 28773.000 |
| Standard Error | 1404.744 |
| Standardized Test Statistic | 1.107 |
| Asymptotic Sig.(2-sided test) | .268 |

**On the scale shown, how much do you agree with the following statements? [I am not at risk of getting an antibiotic-resistant infection, as long as I take my antibiotics correctly] across What is your gender?**

| **Independent-Samples Mann-Whitney U Test Summary** | |
| --- | --- |
| Total N | 476 |
| Mann-Whitney U | 28778.500 |
| Wilcoxon W | 69533.500 |
| Test Statistic | 28778.500 |
| Standard Error | 1437.210 |
| Standardized Test Statistic | 1.086 |
| Asymptotic Sig.(2-sided test) | .277 |

(10) REGION DIFFERENCES ON LIKERT SCALE

**On the scale shown, how much do you agree the following actions would help address the problem of antibiotic resistance? [People should use antibiotics only when they are prescribed by a doctor or nurse] across Which region of Malta do you live in?**

| **Independent-Samples Kruskal-Wallis Test Summary** | |
| --- | --- |
| Total N | 476 |
| Test Statistic | 3.890^a^ |
| Degree Of Freedom | 5 |
| Asymptotic Sig.(2-sided test) | .565 |
| a. The test statistic is adjusted for ties. | |

| **Pairwise Comparisons of Which region of Malta do you live in?** | | | | | |
| --- | --- | --- | --- | --- | --- |
| Sample 1-Sample 2 | Test Statistic | Std. Error | Std. Test Statistic | Sig. | Adj. Sig.^a^ |
| Southern Harbour Region-Northern Harbour Region | 20.276 | 12.815 | 1.582 | .114 | 1.000 |
| Southern Harbour Region-Northern Region | 22.638 | 13.724 | 1.649 | .099 | 1.000 |
| Southern Harbour Region-Western Region | -24.071 | 13.982 | -1.722 | .085 | 1.000 |
| Southern Harbour Region-South Western Region | -25.202 | 14.732 | -1.711 | .087 | 1.000 |
| Southern Harbour Region-Gozo and Comino | -28.764 | 23.618 | -1.218 | .223 | 1.000 |
| Northern Harbour Region-Northern Region | 2.362 | 9.428 | .250 | .802 | 1.000 |
| Northern Harbour Region-Western Region | -3.795 | 9.800 | -.387 | .699 | 1.000 |
| Northern Harbour Region-South Western Region | -4.926 | 10.843 | -.454 | .650 | 1.000 |
| Northern Harbour Region-Gozo and Comino | -8.488 | 21.409 | -.396 | .692 | 1.000 |
| Northern Region-Western Region | -1.434 | 10.962 | -.131 | .896 | 1.000 |
| Northern Region-South Western Region | -2.564 | 11.904 | -.215 | .829 | 1.000 |
| Northern Region-Gozo and Comino | -6.126 | 21.965 | -.279 | .780 | 1.000 |
| Western Region-South Western Region | 1.131 | 12.200 | .093 | .926 | 1.000 |
| Western Region-Gozo and Comino | -4.692 | 22.128 | -.212 | .832 | 1.000 |
| South Western Region-Gozo and Comino | -3.562 | 22.609 | -.158 | .875 | 1.000 |
| Each row tests the null hypothesis that the Sample 1 and Sample 2 distributions are the same.  Asymptotic significances (2-sided tests) are displayed. The significance level is .050. | | | | | |
| a. Significance values have been adjusted by the Bonferroni correction for multiple tests. | | | | | |

**On the scale shown, how much do you agree the following actions would help address the problem of antibiotic resistance? [Farmers should give fewer antibiotics to food-producing animals] across Which region of Malta do you live in?**

| **Independent-Samples Kruskal-Wallis Test Summary** | |
| --- | --- |
| Total N | 476 |
| Test Statistic | 10.583^a^ |
| Degree Of Freedom | 5 |
| Asymptotic Sig.(2-sided test) | .060 |
| a. The test statistic is adjusted for ties. | |

| **Pairwise Comparisons of Which region of Malta do you live in?** | | | | | |
| --- | --- | --- | --- | --- | --- |
| Sample 1-Sample 2 | Test Statistic | Std. Error | Std. Test Statistic | Sig. | Adj. Sig.^a^ |
| Southern Harbour Region-Western Region | -8.839 | 21.976 | -.402 | .688 | 1.000 |
| Southern Harbour Region-Gozo and Comino | -9.168 | 37.121 | -.247 | .805 | 1.000 |
| Southern Harbour Region-Northern Region | 37.449 | 21.571 | 1.736 | .083 | 1.000 |
| Southern Harbour Region-South Western Region | -45.521 | 23.155 | -1.966 | .049 | .740 |
| Southern Harbour Region-Northern Harbour Region | 46.011 | 20.142 | 2.284 | .022 | .335 |
| Western Region-Gozo and Comino | -.329 | 34.778 | -.009 | .992 | 1.000 |
| Western Region-Northern Region | 28.610 | 17.229 | 1.661 | .097 | 1.000 |
| Western Region-South Western Region | 36.683 | 19.175 | 1.913 | .056 | .836 |
| Western Region-Northern Harbour Region | 37.173 | 15.403 | 2.413 | .016 | .237 |
| Gozo and Comino-Northern Region | 28.281 | 34.524 | .819 | .413 | 1.000 |
| Gozo and Comino-South Western Region | 36.354 | 35.535 | 1.023 | .306 | 1.000 |
| Gozo and Comino-Northern Harbour Region | 36.844 | 33.649 | 1.095 | .274 | 1.000 |
| Northern Region-South Western Region | -8.072 | 18.710 | -.431 | .666 | 1.000 |
| Northern Region-Northern Harbour Region | -8.563 | 14.819 | -.578 | .563 | 1.000 |
| South Western Region-Northern Harbour Region | .490 | 17.043 | .029 | .977 | 1.000 |
| Each row tests the null hypothesis that the Sample 1 and Sample 2 distributions are the same.  Asymptotic significances (2-sided tests) are displayed. The significance level is .050. | | | | | |
| a. Significance values have been adjusted by the Bonferroni correction for multiple tests. | | | | | |

**On the scale shown, how much do you agree the following actions would help address the problem of antibiotic resistance? [People should not keep antibiotics and use them later for other illnesses] across Which region of Malta do you live in?**

| **Independent-Samples Kruskal-Wallis Test Summary** | |
| --- | --- |
| Total N | 476 |
| Test Statistic | 9.367^a^ |
| Degree Of Freedom | 5 |
| Asymptotic Sig.(2-sided test) | .095 |
| a. The test statistic is adjusted for ties. | |

| **Pairwise Comparisons of Which region of Malta do you live in?** | | | | | |
| --- | --- | --- | --- | --- | --- |
| Sample 1-Sample 2 | Test Statistic | Std. Error | Std. Test Statistic | Sig. | Adj. Sig.^a^ |
| Southern Harbour Region-Western Region | -20.322 | 21.294 | -.954 | .340 | 1.000 |
| Southern Harbour Region-Northern Region | 32.590 | 20.902 | 1.559 | .119 | 1.000 |
| Southern Harbour Region-South Western Region | -40.397 | 22.437 | -1.800 | .072 | 1.000 |
| Southern Harbour Region-Gozo and Comino | -44.551 | 35.970 | -1.239 | .216 | 1.000 |
| Southern Harbour Region-Northern Harbour Region | 51.523 | 19.517 | 2.640 | .008 | .124 |
| Western Region-Northern Region | 12.268 | 16.694 | .735 | .462 | 1.000 |
| Western Region-South Western Region | 20.076 | 18.581 | 1.080 | .280 | 1.000 |
| Western Region-Gozo and Comino | -24.229 | 33.700 | -.719 | .472 | 1.000 |
| Western Region-Northern Harbour Region | 31.202 | 14.925 | 2.091 | .037 | .548 |
| Northern Region-South Western Region | -7.807 | 18.129 | -.431 | .667 | 1.000 |
| Northern Region-Gozo and Comino | -11.961 | 33.453 | -.358 | .721 | 1.000 |
| Northern Region-Northern Harbour Region | -18.933 | 14.359 | -1.319 | .187 | 1.000 |
| South Western Region-Gozo and Comino | -4.154 | 34.433 | -.121 | .904 | 1.000 |
| South Western Region-Northern Harbour Region | 11.126 | 16.514 | .674 | .500 | 1.000 |
| Gozo and Comino-Northern Harbour Region | 6.972 | 32.606 | .214 | .831 | 1.000 |
| Each row tests the null hypothesis that the Sample 1 and Sample 2 distributions are the same.  Asymptotic significances (2-sided tests) are displayed. The significance level is .050. | | | | | |
| a. Significance values have been adjusted by the Bonferroni correction for multiple tests. | | | | | |

**On the scale shown, how much do you agree the following actions would help address the problem of antibiotic resistance? [Parents should make sure all of their children’s vaccinations are up-to-date] across Which region of Malta do you live in?**

| **Independent-Samples Kruskal-Wallis Test Summary** | |
| --- | --- |
| Total N | 476 |
| Test Statistic | 3.589^a^ |
| Degree Of Freedom | 5 |
| Asymptotic Sig.(2-sided test) | .610 |
| a. The test statistic is adjusted for ties. | |

| **Pairwise Comparisons of Which region of Malta do you live in?** | | | | | |
| --- | --- | --- | --- | --- | --- |
| Sample 1-Sample 2 | Test Statistic | Std. Error | Std. Test Statistic | Sig. | Adj. Sig.^a^ |
| Gozo and Comino-Western Region | 11.252 | 24.228 | .464 | .642 | 1.000 |
| Gozo and Comino-Southern Harbour Region | 16.412 | 25.861 | .635 | .526 | 1.000 |
| Gozo and Comino-Northern Harbour Region | 19.924 | 23.442 | .850 | .395 | 1.000 |
| Gozo and Comino-Northern Region | 26.152 | 24.051 | 1.087 | .277 | 1.000 |
| Gozo and Comino-South Western Region | 30.923 | 24.755 | 1.249 | .212 | 1.000 |
| Western Region-Southern Harbour Region | 5.160 | 15.310 | .337 | .736 | 1.000 |
| Western Region-Northern Harbour Region | 8.672 | 10.730 | .808 | .419 | 1.000 |
| Western Region-Northern Region | 14.900 | 12.002 | 1.241 | .214 | 1.000 |
| Western Region-South Western Region | 19.671 | 13.358 | 1.473 | .141 | 1.000 |
| Southern Harbour Region-Northern Harbour Region | 3.512 | 14.032 | .250 | .802 | 1.000 |
| Southern Harbour Region-Northern Region | 9.740 | 15.027 | .648 | .517 | 1.000 |
| Southern Harbour Region-South Western Region | -14.511 | 16.131 | -.900 | .368 | 1.000 |
| Northern Harbour Region-Northern Region | 6.228 | 10.323 | .603 | .546 | 1.000 |
| Northern Harbour Region-South Western Region | -10.999 | 11.873 | -.926 | .354 | 1.000 |
| Northern Region-South Western Region | -4.771 | 13.034 | -.366 | .714 | 1.000 |
| Each row tests the null hypothesis that the Sample 1 and Sample 2 distributions are the same.  Asymptotic significances (2-sided tests) are displayed. The significance level is .050. | | | | | |
| a. Significance values have been adjusted by the Bonferroni correction for multiple tests. | | | | | |

**On the scale shown, how much do you agree the following actions would help address the problem of antibiotic resistance? [People should wash their hands regularly] across Which region of Malta do you live in?**

| **Independent-Samples Kruskal-Wallis Test Summary** | |
| --- | --- |
| Total N | 476 |
| Test Statistic | 1.644^a^ |
| Degree Of Freedom | 5 |
| Asymptotic Sig.(2-sided test) | .896 |
| a. The test statistic is adjusted for ties. | |

| **Pairwise Comparisons of Which region of Malta do you live in?** | | | | | |
| --- | --- | --- | --- | --- | --- |
| Sample 1-Sample 2 | Test Statistic | Std. Error | Std. Test Statistic | Sig. | Adj. Sig.^a^ |
| Gozo and Comino-Western Region | 18.700 | 23.735 | .788 | .431 | 1.000 |
| Gozo and Comino-Northern Region | 20.024 | 23.561 | .850 | .395 | 1.000 |
| Gozo and Comino-Northern Harbour Region | 23.117 | 22.964 | 1.007 | .314 | 1.000 |
| Gozo and Comino-South Western Region | 24.392 | 24.251 | 1.006 | .315 | 1.000 |
| Gozo and Comino-Southern Harbour Region | 29.388 | 25.334 | 1.160 | .246 | 1.000 |
| Western Region-Northern Region | 1.324 | 11.758 | .113 | .910 | 1.000 |
| Western Region-Northern Harbour Region | 4.417 | 10.512 | .420 | .674 | 1.000 |
| Western Region-South Western Region | 5.692 | 13.086 | .435 | .664 | 1.000 |
| Western Region-Southern Harbour Region | 10.688 | 14.998 | .713 | .476 | 1.000 |
| Northern Region-Northern Harbour Region | -3.093 | 10.113 | -.306 | .760 | 1.000 |
| Northern Region-South Western Region | -4.368 | 12.769 | -.342 | .732 | 1.000 |
| Northern Region-Southern Harbour Region | -9.364 | 14.721 | -.636 | .525 | 1.000 |
| Northern Harbour Region-South Western Region | -1.275 | 11.631 | -.110 | .913 | 1.000 |
| Northern Harbour Region-Southern Harbour Region | -6.271 | 13.746 | -.456 | .648 | 1.000 |
| South Western Region-Southern Harbour Region | 4.996 | 15.803 | .316 | .752 | 1.000 |
| Each row tests the null hypothesis that the Sample 1 and Sample 2 distributions are the same.  Asymptotic significances (2-sided tests) are displayed. The significance level is .050. | | | | | |
| a. Significance values have been adjusted by the Bonferroni correction for multiple tests. | | | | | |

**On the scale shown, how much do you agree the following actions would help address the problem of antibiotic resistance? [Doctors should only prescribe antibiotics when they are needed ] across Which region of Malta do you live in?**

| **Independent-Samples Kruskal-Wallis Test Summary** | |
| --- | --- |
| Total N | 476 |
| Test Statistic | 4.593^a^ |
| Degree Of Freedom | 5 |
| Asymptotic Sig.(2-sided test) | .468 |
| a. The test statistic is adjusted for ties. | |

| **Pairwise Comparisons of Which region of Malta do you live in?** | | | | | |
| --- | --- | --- | --- | --- | --- |
| Sample 1-Sample 2 | Test Statistic | Std. Error | Std. Test Statistic | Sig. | Adj. Sig.^a^ |
| Southern Harbour Region-Western Region | -14.206 | 11.376 | -1.249 | .212 | 1.000 |
| Southern Harbour Region-Gozo and Comino | -15.798 | 19.217 | -.822 | .411 | 1.000 |
| Southern Harbour Region-Northern Region | 19.277 | 11.167 | 1.726 | .084 | 1.000 |
| Southern Harbour Region-South Western Region | -19.398 | 11.987 | -1.618 | .106 | 1.000 |
| Southern Harbour Region-Northern Harbour Region | 21.301 | 10.427 | 2.043 | .041 | .616 |
| Western Region-Gozo and Comino | -1.592 | 18.004 | -.088 | .930 | 1.000 |
| Western Region-Northern Region | 5.072 | 8.919 | .569 | .570 | 1.000 |
| Western Region-South Western Region | 5.192 | 9.927 | .523 | .601 | 1.000 |
| Western Region-Northern Harbour Region | 7.095 | 7.973 | .890 | .374 | 1.000 |
| Gozo and Comino-Northern Region | 3.480 | 17.872 | .195 | .846 | 1.000 |
| Gozo and Comino-South Western Region | 3.600 | 18.396 | .196 | .845 | 1.000 |
| Gozo and Comino-Northern Harbour Region | 5.503 | 17.419 | .316 | .752 | 1.000 |
| Northern Region-South Western Region | -.120 | 9.685 | -.012 | .990 | 1.000 |
| Northern Region-Northern Harbour Region | -2.023 | 7.671 | -.264 | .792 | 1.000 |
| South Western Region-Northern Harbour Region | 1.903 | 8.823 | .216 | .829 | 1.000 |
| Each row tests the null hypothesis that the Sample 1 and Sample 2 distributions are the same.  Asymptotic significances (2-sided tests) are displayed. The significance level is .050. | | | | | |
| a. Significance values have been adjusted by the Bonferroni correction for multiple tests. | | | | | |

**On the scale shown, how much do you agree the following actions would help address the problem of antibiotic resistance? [Governments should reward the development of new antibiotics] across Which region of Malta do you live in?**

| **Independent-Samples Kruskal-Wallis Test Summary** | |
| --- | --- |
| Total N | 476 |
| Test Statistic | 2.758^a^ |
| Degree Of Freedom | 5 |
| Asymptotic Sig.(2-sided test) | .737 |
| a. The test statistic is adjusted for ties. | |

| **Pairwise Comparisons of Which region of Malta do you live in?** | | | | | |
| --- | --- | --- | --- | --- | --- |
| Sample 1-Sample 2 | Test Statistic | Std. Error | Std. Test Statistic | Sig. | Adj. Sig.^a^ |
| South Western Region-Northern Region | 3.531 | 20.705 | .171 | .865 | 1.000 |
| South Western Region-Southern Harbour Region | 8.524 | 25.625 | .333 | .739 | 1.000 |
| South Western Region-Western Region | -18.694 | 21.221 | -.881 | .378 | 1.000 |
| South Western Region-Northern Harbour Region | 20.935 | 18.861 | 1.110 | .267 | 1.000 |
| South Western Region-Gozo and Comino | -43.608 | 39.325 | -1.109 | .267 | 1.000 |
| Northern Region-Southern Harbour Region | -4.993 | 23.872 | -.209 | .834 | 1.000 |
| Northern Region-Western Region | -15.163 | 19.066 | -.795 | .426 | 1.000 |
| Northern Region-Northern Harbour Region | -17.404 | 16.399 | -1.061 | .289 | 1.000 |
| Northern Region-Gozo and Comino | -40.077 | 38.206 | -1.049 | .294 | 1.000 |
| Southern Harbour Region-Western Region | -10.170 | 24.320 | -.418 | .676 | 1.000 |
| Southern Harbour Region-Northern Harbour Region | 12.411 | 22.291 | .557 | .578 | 1.000 |
| Southern Harbour Region-Gozo and Comino | -35.083 | 41.081 | -.854 | .393 | 1.000 |
| Western Region-Northern Harbour Region | 2.241 | 17.045 | .131 | .895 | 1.000 |
| Western Region-Gozo and Comino | -24.914 | 38.488 | -.647 | .517 | 1.000 |
| Northern Harbour Region-Gozo and Comino | -22.673 | 37.239 | -.609 | .543 | 1.000 |
| Each row tests the null hypothesis that the Sample 1 and Sample 2 distributions are the same.  Asymptotic significances (2-sided tests) are displayed. The significance level is .050. | | | | | |
| a. Significance values have been adjusted by the Bonferroni correction for multiple tests. | | | | | |

**On the scale shown, how much do you agree the following actions would help address the problem of antibiotic resistance? [Pharmaceutical companies should develop new antibiotics] across Which region of Malta do you live in?**

| **Independent-Samples Kruskal-Wallis Test Summary** | |
| --- | --- |
| Total N | 476 |
| Test Statistic | 6.936^a^ |
| Degree Of Freedom | 5 |
| Asymptotic Sig.(2-sided test) | .225 |
| a. The test statistic is adjusted for ties. | |

| **Pairwise Comparisons of Which region of Malta do you live in?** | | | | | |
| --- | --- | --- | --- | --- | --- |
| Sample 1-Sample 2 | Test Statistic | Std. Error | Std. Test Statistic | Sig. | Adj. Sig.^a^ |
| Northern Region-South Western Region | -16.750 | 20.774 | -.806 | .420 | 1.000 |
| Northern Region-Northern Harbour Region | -29.189 | 16.453 | -1.774 | .076 | 1.000 |
| Northern Region-Southern Harbour Region | -30.667 | 23.950 | -1.280 | .200 | 1.000 |
| Northern Region-Gozo and Comino | -39.635 | 38.332 | -1.034 | .301 | 1.000 |
| Northern Region-Western Region | -47.537 | 19.129 | -2.485 | .013 | .194 |
| South Western Region-Northern Harbour Region | 12.439 | 18.923 | .657 | .511 | 1.000 |
| South Western Region-Southern Harbour Region | 13.917 | 25.710 | .541 | .588 | 1.000 |
| South Western Region-Gozo and Comino | -22.885 | 39.455 | -.580 | .562 | 1.000 |
| South Western Region-Western Region | -30.787 | 21.291 | -1.446 | .148 | 1.000 |
| Northern Harbour Region-Southern Harbour Region | -1.478 | 22.364 | -.066 | .947 | 1.000 |
| Northern Harbour Region-Gozo and Comino | -10.446 | 37.362 | -.280 | .780 | 1.000 |
| Northern Harbour Region-Western Region | -18.349 | 17.102 | -1.073 | .283 | 1.000 |
| Southern Harbour Region-Gozo and Comino | -8.968 | 41.217 | -.218 | .828 | 1.000 |
| Southern Harbour Region-Western Region | -16.871 | 24.400 | -.691 | .489 | 1.000 |
| Gozo and Comino-Western Region | 7.903 | 38.615 | .205 | .838 | 1.000 |
| Each row tests the null hypothesis that the Sample 1 and Sample 2 distributions are the same.  Asymptotic significances (2-sided tests) are displayed. The significance level is .050. | | | | | |
| a. Significance values have been adjusted by the Bonferroni correction for multiple tests. | | | | | |

**On the scale shown, how much do you agree with the following statements? [Antibiotic resistance is one of the biggest problems the world faces] across Which region of Malta do you live in?**

| **Independent-Samples Kruskal-Wallis Test Summary** | |
| --- | --- |
| Total N | 476 |
| Test Statistic | 8.051^a^ |
| Degree Of Freedom | 5 |
| Asymptotic Sig.(2-sided test) | .153 |
| a. The test statistic is adjusted for ties. | |

| **Pairwise Comparisons of Which region of Malta do you live in?** | | | | | |
| --- | --- | --- | --- | --- | --- |
| Sample 1-Sample 2 | Test Statistic | Std. Error | Std. Test Statistic | Sig. | Adj. Sig.^a^ |
| Gozo and Comino-Southern Harbour Region | 79.505 | 42.009 | 1.893 | .058 | .876 |
| Gozo and Comino-Northern Region | 84.516 | 39.069 | 2.163 | .031 | .458 |
| Gozo and Comino-Northern Harbour Region | 94.652 | 38.080 | 2.486 | .013 | .194 |
| Gozo and Comino-Western Region | 101.237 | 39.357 | 2.572 | .010 | .152 |
| Gozo and Comino-South Western Region | 104.785 | 40.213 | 2.606 | .009 | .138 |
| Southern Harbour Region-Northern Region | 5.012 | 24.411 | .205 | .837 | 1.000 |
| Southern Harbour Region-Northern Harbour Region | 15.147 | 22.794 | .665 | .506 | 1.000 |
| Southern Harbour Region-Western Region | -21.732 | 24.869 | -.874 | .382 | 1.000 |
| Southern Harbour Region-South Western Region | -25.280 | 26.204 | -.965 | .335 | 1.000 |
| Northern Region-Northern Harbour Region | -10.135 | 16.769 | -.604 | .546 | 1.000 |
| Northern Region-Western Region | -16.720 | 19.497 | -.858 | .391 | 1.000 |
| Northern Region-South Western Region | -20.268 | 21.173 | -.957 | .338 | 1.000 |
| Northern Harbour Region-Western Region | -6.585 | 17.430 | -.378 | .706 | 1.000 |
| Northern Harbour Region-South Western Region | -10.133 | 19.287 | -.525 | .599 | 1.000 |
| Western Region-South Western Region | 3.548 | 21.700 | .164 | .870 | 1.000 |
| Each row tests the null hypothesis that the Sample 1 and Sample 2 distributions are the same.  Asymptotic significances (2-sided tests) are displayed. The significance level is .050. | | | | | |
| a. Significance values have been adjusted by the Bonferroni correction for multiple tests. | | | | | |

**On the scale shown, how much do you agree with the following statements? [Medical experts will solve the problem of antibiotic resistance before it becomes too serious] across Which region of Malta do you live in?**

| **Independent-Samples Kruskal-Wallis Test Summary** | |
| --- | --- |
| Total N | 476 |
| Test Statistic | 6.483^a^ |
| Degree Of Freedom | 5 |
| Asymptotic Sig.(2-sided test) | .262 |
| a. The test statistic is adjusted for ties. | |

| **Pairwise Comparisons of Which region of Malta do you live in?** | | | | | |
| --- | --- | --- | --- | --- | --- |
| Sample 1-Sample 2 | Test Statistic | Std. Error | Std. Test Statistic | Sig. | Adj. Sig.^a^ |
| Northern Harbour Region-Northern Region | 4.024 | 16.459 | .244 | .807 | 1.000 |
| Northern Harbour Region-Western Region | -26.266 | 17.108 | -1.535 | .125 | 1.000 |
| Northern Harbour Region-South Western Region | -31.425 | 18.930 | -1.660 | .097 | 1.000 |
| Northern Harbour Region-Southern Harbour Region | -33.296 | 22.372 | -1.488 | .137 | 1.000 |
| Northern Harbour Region-Gozo and Comino | -50.902 | 37.375 | -1.362 | .173 | 1.000 |
| Northern Region-Western Region | -22.242 | 19.136 | -1.162 | .245 | 1.000 |
| Northern Region-South Western Region | -27.401 | 20.781 | -1.319 | .187 | 1.000 |
| Northern Region-Southern Harbour Region | -29.272 | 23.959 | -1.222 | .222 | 1.000 |
| Northern Region-Gozo and Comino | -46.878 | 38.346 | -1.223 | .222 | 1.000 |
| Western Region-South Western Region | 5.160 | 21.298 | .242 | .809 | 1.000 |
| Western Region-Southern Harbour Region | 7.030 | 24.409 | .288 | .773 | 1.000 |
| Western Region-Gozo and Comino | -24.637 | 38.629 | -.638 | .524 | 1.000 |
| South Western Region-Southern Harbour Region | 1.871 | 25.719 | .073 | .942 | 1.000 |
| South Western Region-Gozo and Comino | -19.477 | 39.469 | -.493 | .622 | 1.000 |
| Southern Harbour Region-Gozo and Comino | -17.606 | 41.231 | -.427 | .669 | 1.000 |
| Each row tests the null hypothesis that the Sample 1 and Sample 2 distributions are the same.  Asymptotic significances (2-sided tests) are displayed. The significance level is .050. | | | | | |
| a. Significance values have been adjusted by the Bonferroni correction for multiple tests. | | | | | |

**On the scale shown, how much do you agree with the following statements? [Everyone needs to take responsibility for using antibiotics responsibly] across Which region of Malta do you live in?**

| **Independent-Samples Kruskal-Wallis Test Summary** | |
| --- | --- |
| Total N | 476 |
| Test Statistic | 7.406^a^ |
| Degree Of Freedom | 5 |
| Asymptotic Sig.(2-sided test) | .192 |
| a. The test statistic is adjusted for ties. | |

| **Pairwise Comparisons of Which region of Malta do you live in?** | | | | | |
| --- | --- | --- | --- | --- | --- |
| Sample 1-Sample 2 | Test Statistic | Std. Error | Std. Test Statistic | Sig. | Adj. Sig.^a^ |
| Southern Harbour Region-Northern Region | 16.440 | 15.960 | 1.030 | .303 | 1.000 |
| Southern Harbour Region-Western Region | -22.567 | 16.260 | -1.388 | .165 | 1.000 |
| Southern Harbour Region-Northern Harbour Region | 31.651 | 14.903 | 2.124 | .034 | .505 |
| Southern Harbour Region-South Western Region | -36.804 | 17.133 | -2.148 | .032 | .476 |
| Southern Harbour Region-Gozo and Comino | -44.427 | 27.466 | -1.617 | .106 | 1.000 |
| Northern Region-Western Region | -6.127 | 12.748 | -.481 | .631 | 1.000 |
| Northern Region-Northern Harbour Region | -15.211 | 10.964 | -1.387 | .165 | 1.000 |
| Northern Region-South Western Region | -20.363 | 13.843 | -1.471 | .141 | 1.000 |
| Northern Region-Gozo and Comino | -27.986 | 25.544 | -1.096 | .273 | 1.000 |
| Western Region-Northern Harbour Region | 9.084 | 11.396 | .797 | .425 | 1.000 |
| Western Region-South Western Region | 14.236 | 14.188 | 1.003 | .316 | 1.000 |
| Western Region-Gozo and Comino | -21.859 | 25.733 | -.849 | .396 | 1.000 |
| Northern Harbour Region-South Western Region | -5.152 | 12.610 | -.409 | .683 | 1.000 |
| Northern Harbour Region-Gozo and Comino | -12.776 | 24.897 | -.513 | .608 | 1.000 |
| South Western Region-Gozo and Comino | -7.623 | 26.293 | -.290 | .772 | 1.000 |
| Each row tests the null hypothesis that the Sample 1 and Sample 2 distributions are the same.  Asymptotic significances (2-sided tests) are displayed. The significance level is .050. | | | | | |
| a. Significance values have been adjusted by the Bonferroni correction for multiple tests. | | | | | |

**On the scale shown, how much do you agree with the following statements? [There is not much people like me can do to stop antibiotic resistance] across Which region of Malta do you live in?**

| **Independent-Samples Kruskal-Wallis Test Summary** | |
| --- | --- |
| Total N | 476 |
| Test Statistic | 5.936^a^ |
| Degree Of Freedom | 5 |
| Asymptotic Sig.(2-sided test) | .313 |
| a. The test statistic is adjusted for ties. | |

| **Pairwise Comparisons of Which region of Malta do you live in?** | | | | | |
| --- | --- | --- | --- | --- | --- |
| Sample 1-Sample 2 | Test Statistic | Std. Error | Std. Test Statistic | Sig. | Adj. Sig.^a^ |
| Northern Region-Western Region | -11.803 | 19.734 | -.598 | .550 | 1.000 |
| Northern Region-South Western Region | -14.280 | 21.431 | -.666 | .505 | 1.000 |
| Northern Region-Northern Harbour Region | -19.935 | 16.974 | -1.174 | .240 | 1.000 |
| Northern Region-Southern Harbour Region | -52.405 | 24.708 | -2.121 | .034 | .509 |
| Northern Region-Gozo and Comino | -57.541 | 39.544 | -1.455 | .146 | 1.000 |
| Western Region-South Western Region | 2.476 | 21.964 | .113 | .910 | 1.000 |
| Western Region-Northern Harbour Region | 8.132 | 17.643 | .461 | .645 | 1.000 |
| Western Region-Southern Harbour Region | 40.601 | 25.172 | 1.613 | .107 | 1.000 |
| Western Region-Gozo and Comino | -45.738 | 39.836 | -1.148 | .251 | 1.000 |
| South Western Region-Northern Harbour Region | 5.655 | 19.521 | .290 | .772 | 1.000 |
| South Western Region-Southern Harbour Region | 38.125 | 26.523 | 1.437 | .151 | 1.000 |
| South Western Region-Gozo and Comino | -43.262 | 40.703 | -1.063 | .288 | 1.000 |
| Northern Harbour Region-Southern Harbour Region | -32.470 | 23.071 | -1.407 | .159 | 1.000 |
| Northern Harbour Region-Gozo and Comino | -37.606 | 38.543 | -.976 | .329 | 1.000 |
| Southern Harbour Region-Gozo and Comino | -5.136 | 42.520 | -.121 | .904 | 1.000 |
| Each row tests the null hypothesis that the Sample 1 and Sample 2 distributions are the same.  Asymptotic significances (2-sided tests) are displayed. The significance level is .050. | | | | | |
| a. Significance values have been adjusted by the Bonferroni correction for multiple tests. | | | | | |

**On the scale shown, how much do you agree with the following statements? [I am worried about the impact that antibiotic resistance will have on my health, and that of my family] across Which region of Malta do you live in?**

| **Independent-Samples Kruskal-Wallis Test Summary** | |
| --- | --- |
| Total N | 476 |
| Test Statistic | 5.561^a^ |
| Degree Of Freedom | 5 |
| Asymptotic Sig.(2-sided test) | .351 |
| a. The test statistic is adjusted for ties. | |

| **Pairwise Comparisons of Which region of Malta do you live in?** | | | | | |
| --- | --- | --- | --- | --- | --- |
| Sample 1-Sample 2 | Test Statistic | Std. Error | Std. Test Statistic | Sig. | Adj. Sig.^a^ |
| Gozo and Comino-South Western Region | 29.285 | 39.910 | .734 | .463 | 1.000 |
| Gozo and Comino-Northern Region | 30.298 | 38.774 | .781 | .435 | 1.000 |
| Gozo and Comino-Northern Harbour Region | 45.586 | 37.792 | 1.206 | .228 | 1.000 |
| Gozo and Comino-Southern Harbour Region | 50.825 | 41.691 | 1.219 | .223 | 1.000 |
| Gozo and Comino-Western Region | 64.488 | 39.060 | 1.651 | .099 | 1.000 |
| South Western Region-Northern Region | 1.013 | 21.013 | .048 | .962 | 1.000 |
| South Western Region-Northern Harbour Region | 16.302 | 19.141 | .852 | .394 | 1.000 |
| South Western Region-Southern Harbour Region | 21.540 | 26.006 | .828 | .408 | 1.000 |
| South Western Region-Western Region | -35.203 | 21.536 | -1.635 | .102 | 1.000 |
| Northern Region-Northern Harbour Region | -15.288 | 16.643 | -.919 | .358 | 1.000 |
| Northern Region-Southern Harbour Region | -20.527 | 24.226 | -.847 | .397 | 1.000 |
| Northern Region-Western Region | -34.190 | 19.350 | -1.767 | .077 | 1.000 |
| Northern Harbour Region-Southern Harbour Region | -5.239 | 22.622 | -.232 | .817 | 1.000 |
| Northern Harbour Region-Western Region | -18.902 | 17.299 | -1.093 | .275 | 1.000 |
| Southern Harbour Region-Western Region | -13.663 | 24.682 | -.554 | .580 | 1.000 |
| Each row tests the null hypothesis that the Sample 1 and Sample 2 distributions are the same.  Asymptotic significances (2-sided tests) are displayed. The significance level is .050. | | | | | |
| a. Significance values have been adjusted by the Bonferroni correction for multiple tests. | | | | | |

**On the scale shown, how much do you agree with the following statements? [I am not at risk of getting an antibiotic-resistant infection, as long as I take my antibiotics correctly] across Which region of Malta do you live in?**

| **Independent-Samples Kruskal-Wallis Test Summary** | |
| --- | --- |
| Total N | 476 |
| Test Statistic | 11.805^a^ |
| Degree Of Freedom | 5 |
| Asymptotic Sig.(2-sided test) | .038 |
| a. The test statistic is adjusted for ties. | |

| **Pairwise Comparisons of Which region of Malta do you live in?** | | | | | |
| --- | --- | --- | --- | --- | --- |
| Sample 1-Sample 2 | Test Statistic | Std. Error | Std. Test Statistic | Sig. | Adj. Sig.^a^ |
| Northern Harbour Region-Gozo and Comino | -8.457 | 38.666 | -.219 | .827 | 1.000 |
| Northern Harbour Region-Western Region | -19.925 | 17.699 | -1.126 | .260 | 1.000 |
| Northern Harbour Region-Northern Region | 20.619 | 17.027 | 1.211 | .226 | 1.000 |
| Northern Harbour Region-South Western Region | -49.472 | 19.583 | -2.526 | .012 | .173 |
| Northern Harbour Region-Southern Harbour Region | -65.459 | 23.145 | -2.828 | .005 | .070 |
| Gozo and Comino-Western Region | 11.468 | 39.963 | .287 | .774 | 1.000 |
| Gozo and Comino-Northern Region | 12.162 | 39.670 | .307 | .759 | 1.000 |
| Gozo and Comino-South Western Region | 41.015 | 40.832 | 1.004 | .315 | 1.000 |
| Gozo and Comino-Southern Harbour Region | 57.002 | 42.655 | 1.336 | .181 | 1.000 |
| Western Region-Northern Region | .694 | 19.797 | .035 | .972 | 1.000 |
| Western Region-South Western Region | 29.547 | 22.034 | 1.341 | .180 | 1.000 |
| Western Region-Southern Harbour Region | 45.534 | 25.252 | 1.803 | .071 | 1.000 |
| Northern Region-South Western Region | -28.854 | 21.499 | -1.342 | .180 | 1.000 |
| Northern Region-Southern Harbour Region | -44.840 | 24.786 | -1.809 | .070 | 1.000 |
| South Western Region-Southern Harbour Region | 15.986 | 26.607 | .601 | .548 | 1.000 |
| Each row tests the null hypothesis that the Sample 1 and Sample 2 distributions are the same.  Asymptotic significances (2-sided tests) are displayed. The significance level is .050. | | | | | |
| a. Significance values have been adjusted by the Bonferroni correction for multiple tests. | | | | | |

(11) INCOME DIFFERENCES ON LIKERT SCALE

**On the scale shown, how much do you agree the following actions would help address the problem of antibiotic resistance? [People should use antibiotics only when they are prescribed by a doctor or nurse] across HouseholdIncome**

| **Independent-Samples Kruskal-Wallis Test Summary** | |
| --- | --- |
| Total N | 476 |
| Test Statistic | 4.322^a^ |
| Degree Of Freedom | 4 |
| Asymptotic Sig.(2-sided test) | .364 |
| a. The test statistic is adjusted for ties. | |

| **Pairwise Comparisons of HouseholdIncome** | | | | | |
| --- | --- | --- | --- | --- | --- |
| Sample 1-Sample 2 | Test Statistic | Std. Error | Std. Test Statistic | Sig. | Adj. Sig.^a^ |
| Up to Eur24999-Eur25000-49999 | -20.259 | 11.369 | -1.782 | .075 | .747 |
| Up to Eur24999-Eur50000-99999 | -21.785 | 11.823 | -1.843 | .065 | .654 |
| Up to Eur24999-Prefer Not to Say | -22.450 | 13.594 | -1.651 | .099 | .986 |
| Up to Eur24999-Eur100000+ | -22.936 | 13.347 | -1.718 | .086 | .857 |
| Eur25000-49999-Eur50000-99999 | -1.527 | 8.836 | -.173 | .863 | 1.000 |
| Eur25000-49999-Prefer Not to Say | -2.191 | 11.094 | -.198 | .843 | 1.000 |
| Eur25000-49999-Eur100000+ | -2.677 | 10.790 | -.248 | .804 | 1.000 |
| Eur50000-99999-Prefer Not to Say | -.665 | 11.559 | -.058 | .954 | 1.000 |
| Eur50000-99999-Eur100000+ | -1.150 | 11.267 | -.102 | .919 | 1.000 |
| Prefer Not to Say-Eur100000+ | .485 | 13.114 | .037 | .970 | 1.000 |
| Each row tests the null hypothesis that the Sample 1 and Sample 2 distributions are the same.  Asymptotic significances (2-sided tests) are displayed. The significance level is .050. | | | | | |
| a. Significance values have been adjusted by the Bonferroni correction for multiple tests. | | | | | |

**On the scale shown, how much do you agree the following actions would help address the problem of antibiotic resistance? [Farmers should give fewer antibiotics to food-producing animals] across HouseholdIncome**

| **Independent-Samples Kruskal-Wallis Test Summary** | |
| --- | --- |
| Total N | 476 |
| Test Statistic | 6.791^a^ |
| Degree Of Freedom | 4 |
| Asymptotic Sig.(2-sided test) | .147 |
| a. The test statistic is adjusted for ties. | |

| **Pairwise Comparisons of HouseholdIncome** | | | | | |
| --- | --- | --- | --- | --- | --- |
| Sample 1-Sample 2 | Test Statistic | Std. Error | Std. Test Statistic | Sig. | Adj. Sig.^a^ |
| Up to Eur24999-Eur25000-49999 | -32.902 | 17.868 | -1.841 | .066 | .656 |
| Up to Eur24999-Eur50000-99999 | -37.408 | 18.582 | -2.013 | .044 | .441 |
| Up to Eur24999-Prefer Not to Say | -38.943 | 21.366 | -1.823 | .068 | .684 |
| Up to Eur24999-Eur100000+ | -52.433 | 20.977 | -2.500 | .012 | .124 |
| Eur25000-49999-Eur50000-99999 | -4.506 | 13.887 | -.325 | .746 | 1.000 |
| Eur25000-49999-Prefer Not to Say | -6.041 | 17.437 | -.346 | .729 | 1.000 |
| Eur25000-49999-Eur100000+ | -19.531 | 16.958 | -1.152 | .249 | 1.000 |
| Eur50000-99999-Prefer Not to Say | -1.535 | 18.168 | -.084 | .933 | 1.000 |
| Eur50000-99999-Eur100000+ | -15.025 | 17.709 | -.848 | .396 | 1.000 |
| Prefer Not to Say-Eur100000+ | 13.490 | 20.611 | .655 | .513 | 1.000 |
| Each row tests the null hypothesis that the Sample 1 and Sample 2 distributions are the same.  Asymptotic significances (2-sided tests) are displayed. The significance level is .050. | | | | | |
| a. Significance values have been adjusted by the Bonferroni correction for multiple tests. | | | | | |

**On the scale shown, how much do you agree the following actions would help address the problem of antibiotic resistance? [People should not keep antibiotics and use them later for other illnesses] across HouseholdIncome**

| **Independent-Samples Kruskal-Wallis Test Summary** | |
| --- | --- |
| Total N | 476 |
| Test Statistic | 4.893^a^ |
| Degree Of Freedom | 4 |
| Asymptotic Sig.(2-sided test) | .298 |
| a. The test statistic is adjusted for ties. | |

| **Pairwise Comparisons of HouseholdIncome** | | | | | |
| --- | --- | --- | --- | --- | --- |
| Sample 1-Sample 2 | Test Statistic | Std. Error | Std. Test Statistic | Sig. | Adj. Sig.^a^ |
| Up to Eur24999-Eur100000+ | -22.454 | 20.326 | -1.105 | .269 | 1.000 |
| Up to Eur24999-Prefer Not to Say | -31.866 | 20.703 | -1.539 | .124 | 1.000 |
| Up to Eur24999-Eur25000-49999 | -33.836 | 17.314 | -1.954 | .051 | .507 |
| Up to Eur24999-Eur50000-99999 | -36.459 | 18.006 | -2.025 | .043 | .429 |
| Eur100000+-Prefer Not to Say | -9.412 | 19.972 | -.471 | .637 | 1.000 |
| Eur100000+-Eur25000-49999 | 11.382 | 16.432 | .693 | .489 | 1.000 |
| Eur100000+-Eur50000-99999 | 14.006 | 17.160 | .816 | .414 | 1.000 |
| Prefer Not to Say-Eur25000-49999 | 1.970 | 16.896 | .117 | .907 | 1.000 |
| Prefer Not to Say-Eur50000-99999 | 4.593 | 17.605 | .261 | .794 | 1.000 |
| Eur25000-49999-Eur50000-99999 | -2.623 | 13.456 | -.195 | .845 | 1.000 |
| Each row tests the null hypothesis that the Sample 1 and Sample 2 distributions are the same.  Asymptotic significances (2-sided tests) are displayed. The significance level is .050. | | | | | |
| a. Significance values have been adjusted by the Bonferroni correction for multiple tests. | | | | | |

**On the scale shown, how much do you agree the following actions would help address the problem of antibiotic resistance? [Parents should make sure all of their children’s vaccinations are up-to-date] across HouseholdIncome**

| **Independent-Samples Kruskal-Wallis Test Summary** | |
| --- | --- |
| Total N | 476 |
| Test Statistic | 2.819^a^ |
| Degree Of Freedom | 4 |
| Asymptotic Sig.(2-sided test) | .589 |
| a. The test statistic is adjusted for ties. | |

| **Pairwise Comparisons of HouseholdIncome** | | | | | |
| --- | --- | --- | --- | --- | --- |
| Sample 1-Sample 2 | Test Statistic | Std. Error | Std. Test Statistic | Sig. | Adj. Sig.^a^ |
| Up to Eur24999-Eur50000-99999 | -1.382 | 12.945 | -.107 | .915 | 1.000 |
| Up to Eur24999-Eur25000-49999 | -1.551 | 12.448 | -.125 | .901 | 1.000 |
| Up to Eur24999-Prefer Not to Say | -10.141 | 14.884 | -.681 | .496 | 1.000 |
| Up to Eur24999-Eur100000+ | -18.415 | 14.614 | -1.260 | .208 | 1.000 |
| Eur50000-99999-Eur25000-49999 | .169 | 9.674 | .017 | .986 | 1.000 |
| Eur50000-99999-Prefer Not to Say | -8.759 | 12.657 | -.692 | .489 | 1.000 |
| Eur50000-99999-Eur100000+ | -17.032 | 12.337 | -1.381 | .167 | 1.000 |
| Eur25000-49999-Prefer Not to Say | -8.590 | 12.148 | -.707 | .479 | 1.000 |
| Eur25000-49999-Eur100000+ | -16.864 | 11.814 | -1.427 | .153 | 1.000 |
| Prefer Not to Say-Eur100000+ | 8.274 | 14.359 | .576 | .564 | 1.000 |
| Each row tests the null hypothesis that the Sample 1 and Sample 2 distributions are the same.  Asymptotic significances (2-sided tests) are displayed. The significance level is .050. | | | | | |
| a. Significance values have been adjusted by the Bonferroni correction for multiple tests. | | | | | |

**On the scale shown, how much do you agree the following actions would help address the problem of antibiotic resistance? [People should wash their hands regularly] across HouseholdIncome**

| **Independent-Samples Kruskal-Wallis Test Summary** | |
| --- | --- |
| Total N | 476 |
| Test Statistic | 1.128^a^ |
| Degree Of Freedom | 4 |
| Asymptotic Sig.(2-sided test) | .890 |
| a. The test statistic is adjusted for ties. | |

| **Pairwise Comparisons of HouseholdIncome** | | | | | |
| --- | --- | --- | --- | --- | --- |
| Sample 1-Sample 2 | Test Statistic | Std. Error | Std. Test Statistic | Sig. | Adj. Sig.^a^ |
| Prefer Not to Say-Eur25000-49999 | 7.484 | 11.900 | .629 | .529 | 1.000 |
| Prefer Not to Say-Eur100000+ | 10.240 | 14.066 | .728 | .467 | 1.000 |
| Prefer Not to Say-Up to Eur24999 | 10.664 | 14.581 | .731 | .465 | 1.000 |
| Prefer Not to Say-Eur50000-99999 | 12.573 | 12.399 | 1.014 | .311 | 1.000 |
| Eur25000-49999-Eur100000+ | -2.757 | 11.573 | -.238 | .812 | 1.000 |
| Eur25000-49999-Up to Eur24999 | 3.180 | 12.194 | .261 | .794 | 1.000 |
| Eur25000-49999-Eur50000-99999 | -5.089 | 9.477 | -.537 | .591 | 1.000 |
| Eur100000+-Up to Eur24999 | .424 | 14.316 | .030 | .976 | 1.000 |
| Eur100000+-Eur50000-99999 | 2.333 | 12.086 | .193 | .847 | 1.000 |
| Up to Eur24999-Eur50000-99999 | -1.909 | 12.682 | -.151 | .880 | 1.000 |
| Each row tests the null hypothesis that the Sample 1 and Sample 2 distributions are the same.  Asymptotic significances (2-sided tests) are displayed. The significance level is .050. | | | | | |
| a. Significance values have been adjusted by the Bonferroni correction for multiple tests. | | | | | |

**On the scale shown, how much do you agree the following actions would help address the problem of antibiotic resistance? [Doctors should only prescribe antibiotics when they are needed ] across HouseholdIncome**

| **Independent-Samples Kruskal-Wallis Test Summary** | |
| --- | --- |
| Total N | 476 |
| Test Statistic | 12.233^a^ |
| Degree Of Freedom | 4 |
| Asymptotic Sig.(2-sided test) | .016 |
| a. The test statistic is adjusted for ties. | |

| **Pairwise Comparisons of HouseholdIncome** | | | | | |
| --- | --- | --- | --- | --- | --- |
| Sample 1-Sample 2 | Test Statistic | Std. Error | Std. Test Statistic | Sig. | Adj. Sig.^a^ |
| Up to Eur24999-Eur25000-49999 | -23.612 | 9.250 | -2.553 | .011 | .107 |
| Up to Eur24999-Prefer Not to Say | -29.695 | 11.061 | -2.685 | .007 | .073 |
| Up to Eur24999-Eur100000+ | -30.308 | 10.859 | -2.791 | .005 | .053 |
| Up to Eur24999-Eur50000-99999 | -31.409 | 9.619 | -3.265 | .001 | .011 |
| Eur25000-49999-Prefer Not to Say | -6.083 | 9.027 | -.674 | .500 | 1.000 |
| Eur25000-49999-Eur100000+ | -6.697 | 8.779 | -.763 | .446 | 1.000 |
| Eur25000-49999-Eur50000-99999 | -7.797 | 7.189 | -1.085 | .278 | 1.000 |
| Prefer Not to Say-Eur100000+ | .614 | 10.670 | .058 | .954 | 1.000 |
| Prefer Not to Say-Eur50000-99999 | 1.715 | 9.405 | .182 | .855 | 1.000 |
| Eur100000+-Eur50000-99999 | 1.101 | 9.168 | .120 | .904 | 1.000 |
| Each row tests the null hypothesis that the Sample 1 and Sample 2 distributions are the same.  Asymptotic significances (2-sided tests) are displayed. The significance level is .050. | | | | | |
| a. Significance values have been adjusted by the Bonferroni correction for multiple tests. | | | | | |

**On the scale shown, how much do you agree the following actions would help address the problem of antibiotic resistance? [Governments should reward the development of new antibiotics] across HouseholdIncome**

| **Independent-Samples Kruskal-Wallis Test Summary** | |
| --- | --- |
| Total N | 476 |
| Test Statistic | 1.939^a^ |
| Degree Of Freedom | 4 |
| Asymptotic Sig.(2-sided test) | .747 |
| a. The test statistic is adjusted for ties. | |

| **Pairwise Comparisons of HouseholdIncome** | | | | | |
| --- | --- | --- | --- | --- | --- |
| Sample 1-Sample 2 | Test Statistic | Std. Error | Std. Test Statistic | Sig. | Adj. Sig.^a^ |
| Up to Eur24999-Eur100000+ | -3.533 | 23.214 | -.152 | .879 | 1.000 |
| Up to Eur24999-Eur25000-49999 | -9.506 | 19.774 | -.481 | .631 | 1.000 |
| Up to Eur24999-Eur50000-99999 | -20.330 | 20.564 | -.989 | .323 | 1.000 |
| Up to Eur24999-Prefer Not to Say | -24.495 | 23.645 | -1.036 | .300 | 1.000 |
| Eur100000+-Eur25000-49999 | 5.973 | 18.767 | .318 | .750 | 1.000 |
| Eur100000+-Eur50000-99999 | 16.797 | 19.598 | .857 | .391 | 1.000 |
| Eur100000+-Prefer Not to Say | -20.962 | 22.809 | -.919 | .358 | 1.000 |
| Eur25000-49999-Eur50000-99999 | -10.824 | 15.368 | -.704 | .481 | 1.000 |
| Eur25000-49999-Prefer Not to Say | -14.989 | 19.297 | -.777 | .437 | 1.000 |
| Eur50000-99999-Prefer Not to Say | -4.166 | 20.106 | -.207 | .836 | 1.000 |
| Each row tests the null hypothesis that the Sample 1 and Sample 2 distributions are the same.  Asymptotic significances (2-sided tests) are displayed. The significance level is .050. | | | | | |
| a. Significance values have been adjusted by the Bonferroni correction for multiple tests. | | | | | |

**On the scale shown, how much do you agree the following actions would help address the problem of antibiotic resistance? [Pharmaceutical companies should develop new antibiotics] across HouseholdIncome**

| **Independent-Samples Kruskal-Wallis Test Summary** | |
| --- | --- |
| Total N | 476 |
| Test Statistic | .969^a^ |
| Degree Of Freedom | 4 |
| Asymptotic Sig.(2-sided test) | .915 |
| a. The test statistic is adjusted for ties. | |

| **Pairwise Comparisons of HouseholdIncome** | | | | | |
| --- | --- | --- | --- | --- | --- |
| Sample 1-Sample 2 | Test Statistic | Std. Error | Std. Test Statistic | Sig. | Adj. Sig.^a^ |
| Eur25000-49999-Up to Eur24999 | 4.526 | 19.839 | .228 | .820 | 1.000 |
| Eur25000-49999-Prefer Not to Say | -9.948 | 19.361 | -.514 | .607 | 1.000 |
| Eur25000-49999-Eur100000+ | -10.382 | 18.829 | -.551 | .581 | 1.000 |
| Eur25000-49999-Eur50000-99999 | -14.289 | 15.419 | -.927 | .354 | 1.000 |
| Up to Eur24999-Prefer Not to Say | -5.422 | 23.723 | -.229 | .819 | 1.000 |
| Up to Eur24999-Eur100000+ | -5.855 | 23.291 | -.251 | .802 | 1.000 |
| Up to Eur24999-Eur50000-99999 | -9.762 | 20.632 | -.473 | .636 | 1.000 |
| Prefer Not to Say-Eur100000+ | .434 | 22.885 | .019 | .985 | 1.000 |
| Prefer Not to Say-Eur50000-99999 | 4.341 | 20.172 | .215 | .830 | 1.000 |
| Eur100000+-Eur50000-99999 | 3.907 | 19.663 | .199 | .842 | 1.000 |
| Each row tests the null hypothesis that the Sample 1 and Sample 2 distributions are the same.  Asymptotic significances (2-sided tests) are displayed. The significance level is .050. | | | | | |
| a. Significance values have been adjusted by the Bonferroni correction for multiple tests. | | | | | |

**On the scale shown, how much do you agree with the following statements? [Antibiotic resistance is one of the biggest problems the world faces] across HouseholdIncome**

| **Independent-Samples Kruskal-Wallis Test Summary** | |
| --- | --- |
| Total N | 476 |
| Test Statistic | 6.893^a^ |
| Degree Of Freedom | 4 |
| Asymptotic Sig.(2-sided test) | .142 |
| a. The test statistic is adjusted for ties. | |

| **Pairwise Comparisons of HouseholdIncome** | | | | | |
| --- | --- | --- | --- | --- | --- |
| Sample 1-Sample 2 | Test Statistic | Std. Error | Std. Test Statistic | Sig. | Adj. Sig.^a^ |
| Eur100000+-Prefer Not to Say | -41.529 | 23.325 | -1.781 | .075 | .750 |
| Eur100000+-Up to Eur24999 | 44.807 | 23.739 | 1.888 | .059 | .591 |
| Eur100000+-Eur50000-99999 | 46.350 | 20.041 | 2.313 | .021 | .207 |
| Eur100000+-Eur25000-49999 | 46.711 | 19.191 | 2.434 | .015 | .149 |
| Prefer Not to Say-Up to Eur24999 | 3.277 | 24.179 | .136 | .892 | 1.000 |
| Prefer Not to Say-Eur50000-99999 | 4.821 | 20.560 | .234 | .815 | 1.000 |
| Prefer Not to Say-Eur25000-49999 | 5.181 | 19.733 | .263 | .793 | 1.000 |
| Up to Eur24999-Eur50000-99999 | -1.544 | 21.029 | -.073 | .941 | 1.000 |
| Up to Eur24999-Eur25000-49999 | -1.904 | 20.221 | -.094 | .925 | 1.000 |
| Eur50000-99999-Eur25000-49999 | .360 | 15.715 | .023 | .982 | 1.000 |
| Each row tests the null hypothesis that the Sample 1 and Sample 2 distributions are the same.  Asymptotic significances (2-sided tests) are displayed. The significance level is .050. | | | | | |
| a. Significance values have been adjusted by the Bonferroni correction for multiple tests. | | | | | |

**On the scale shown, how much do you agree with the following statements? [Medical experts will solve the problem of antibiotic resistance before it becomes too serious] across HouseholdIncome**

| **Independent-Samples Kruskal-Wallis Test Summary** | |
| --- | --- |
| Total N | 476 |
| Test Statistic | 15.494^a^ |
| Degree Of Freedom | 4 |
| Asymptotic Sig.(2-sided test) | .004 |
| a. The test statistic is adjusted for ties. | |

| **Pairwise Comparisons of HouseholdIncome** | | | | | |
| --- | --- | --- | --- | --- | --- |
| Sample 1-Sample 2 | Test Statistic | Std. Error | Std. Test Statistic | Sig. | Adj. Sig.^a^ |
| Eur25000-49999-Eur50000-99999 | -14.693 | 15.425 | -.953 | .341 | 1.000 |
| Eur25000-49999-Prefer Not to Say | -26.457 | 19.368 | -1.366 | .172 | 1.000 |
| Eur25000-49999-Eur100000+ | -27.551 | 18.836 | -1.463 | .144 | 1.000 |
| Eur25000-49999-Up to Eur24999 | 76.642 | 19.846 | 3.862 | <.001 | .001 |
| Eur50000-99999-Prefer Not to Say | -11.763 | 20.180 | -.583 | .560 | 1.000 |
| Eur50000-99999-Eur100000+ | -12.858 | 19.670 | -.654 | .513 | 1.000 |
| Eur50000-99999-Up to Eur24999 | 61.949 | 20.639 | 3.001 | .003 | .027 |
| Prefer Not to Say-Eur100000+ | 1.094 | 22.893 | .048 | .962 | 1.000 |
| Prefer Not to Say-Up to Eur24999 | 50.185 | 23.731 | 2.115 | .034 | .345 |
| Eur100000+-Up to Eur24999 | 49.091 | 23.299 | 2.107 | .035 | .351 |
| Each row tests the null hypothesis that the Sample 1 and Sample 2 distributions are the same.  Asymptotic significances (2-sided tests) are displayed. The significance level is .050. | | | | | |
| a. Significance values have been adjusted by the Bonferroni correction for multiple tests. | | | | | |

**On the scale shown, how much do you agree with the following statements? [Everyone needs to take responsibility for using antibiotics responsibly] across HouseholdIncome**

| **Independent-Samples Kruskal-Wallis Test Summary** | |
| --- | --- |
| Total N | 476 |
| Test Statistic | 3.786^a^ |
| Degree Of Freedom | 4 |
| Asymptotic Sig.(2-sided test) | .436 |
| a. The test statistic is adjusted for ties. | |

| **Pairwise Comparisons of HouseholdIncome** | | | | | |
| --- | --- | --- | --- | --- | --- |
| Sample 1-Sample 2 | Test Statistic | Std. Error | Std. Test Statistic | Sig. | Adj. Sig.^a^ |
| Eur100000+-Prefer Not to Say | -7.569 | 15.250 | -.496 | .620 | 1.000 |
| Eur100000+-Eur50000-99999 | 9.461 | 13.103 | .722 | .470 | 1.000 |
| Eur100000+-Up to Eur24999 | 12.735 | 15.521 | .821 | .412 | 1.000 |
| Eur100000+-Eur25000-49999 | 22.137 | 12.548 | 1.764 | .078 | .777 |
| Prefer Not to Say-Eur50000-99999 | 1.893 | 13.443 | .141 | .888 | 1.000 |
| Prefer Not to Say-Up to Eur24999 | 5.167 | 15.809 | .327 | .744 | 1.000 |
| Prefer Not to Say-Eur25000-49999 | 14.568 | 12.902 | 1.129 | .259 | 1.000 |
| Eur50000-99999-Up to Eur24999 | 3.274 | 13.749 | .238 | .812 | 1.000 |
| Eur50000-99999-Eur25000-49999 | 12.676 | 10.275 | 1.234 | .217 | 1.000 |
| Up to Eur24999-Eur25000-49999 | -9.402 | 13.221 | -.711 | .477 | 1.000 |
| Each row tests the null hypothesis that the Sample 1 and Sample 2 distributions are the same.  Asymptotic significances (2-sided tests) are displayed. The significance level is .050. | | | | | |
| a. Significance values have been adjusted by the Bonferroni correction for multiple tests. | | | | | |

**On the scale shown, how much do you agree with the following statements? [There is not much people like me can do to stop antibiotic resistance] across HouseholdIncome**

| **Independent-Samples Kruskal-Wallis Test Summary** | |
| --- | --- |
| Total N | 476 |
| Test Statistic | 8.540^a^ |
| Degree Of Freedom | 4 |
| Asymptotic Sig.(2-sided test) | .074 |
| a. The test statistic is adjusted for ties. | |

| **Pairwise Comparisons of HouseholdIncome** | | | | | |
| --- | --- | --- | --- | --- | --- |
| Sample 1-Sample 2 | Test Statistic | Std. Error | Std. Test Statistic | Sig. | Adj. Sig.^a^ |
| Eur25000-49999-Eur50000-99999 | -5.759 | 15.907 | -.362 | .717 | 1.000 |
| Eur25000-49999-Eur100000+ | -23.163 | 19.425 | -1.192 | .233 | 1.000 |
| Eur25000-49999-Prefer Not to Say | -26.016 | 19.973 | -1.303 | .193 | 1.000 |
| Eur25000-49999-Up to Eur24999 | 55.198 | 20.467 | 2.697 | .007 | .070 |
| Eur50000-99999-Eur100000+ | -17.404 | 20.285 | -.858 | .391 | 1.000 |
| Eur50000-99999-Prefer Not to Say | -20.257 | 20.810 | -.973 | .330 | 1.000 |
| Eur50000-99999-Up to Eur24999 | 49.438 | 21.285 | 2.323 | .020 | .202 |
| Eur100000+-Prefer Not to Say | -2.853 | 23.609 | -.121 | .904 | 1.000 |
| Eur100000+-Up to Eur24999 | 32.034 | 24.028 | 1.333 | .182 | 1.000 |
| Prefer Not to Say-Up to Eur24999 | 29.181 | 24.473 | 1.192 | .233 | 1.000 |
| Each row tests the null hypothesis that the Sample 1 and Sample 2 distributions are the same.  Asymptotic significances (2-sided tests) are displayed. The significance level is .050. | | | | | |
| a. Significance values have been adjusted by the Bonferroni correction for multiple tests. | | | | | |

**On the scale shown, how much do you agree with the following statements? [I am worried about the impact that antibiotic resistance will have on my health, and that of my family] across HouseholdIncome**

| **Independent-Samples Kruskal-Wallis Test Summary** | |
| --- | --- |
| Total N | 476 |
| Test Statistic | 7.302^a^ |
| Degree Of Freedom | 4 |
| Asymptotic Sig.(2-sided test) | .121 |
| a. The test statistic is adjusted for ties. | |

| **Pairwise Comparisons of HouseholdIncome** | | | | | |
| --- | --- | --- | --- | --- | --- |
| Sample 1-Sample 2 | Test Statistic | Std. Error | Std. Test Statistic | Sig. | Adj. Sig.^a^ |
| Eur100000+-Up to Eur24999 | 19.592 | 23.559 | .832 | .406 | 1.000 |
| Eur100000+-Eur50000-99999 | 24.575 | 19.889 | 1.236 | .217 | 1.000 |
| Eur100000+-Prefer Not to Say | -29.270 | 23.149 | -1.264 | .206 | 1.000 |
| Eur100000+-Eur25000-49999 | 48.464 | 19.046 | 2.545 | .011 | .109 |
| Up to Eur24999-Eur50000-99999 | -4.983 | 20.870 | -.239 | .811 | 1.000 |
| Up to Eur24999-Prefer Not to Say | -9.678 | 23.996 | -.403 | .687 | 1.000 |
| Up to Eur24999-Eur25000-49999 | -28.871 | 20.068 | -1.439 | .150 | 1.000 |
| Eur50000-99999-Prefer Not to Say | -4.695 | 20.405 | -.230 | .818 | 1.000 |
| Eur50000-99999-Eur25000-49999 | 23.889 | 15.597 | 1.532 | .126 | 1.000 |
| Prefer Not to Say-Eur25000-49999 | 19.193 | 19.584 | .980 | .327 | 1.000 |
| Each row tests the null hypothesis that the Sample 1 and Sample 2 distributions are the same.  Asymptotic significances (2-sided tests) are displayed. The significance level is .050. | | | | | |
| a. Significance values have been adjusted by the Bonferroni correction for multiple tests. | | | | | |

**On the scale shown, how much do you agree with the following statements? [I am not at risk of getting an antibiotic-resistant infection, as long as I take my antibiotics correctly] across HouseholdIncome**

| **Independent-Samples Kruskal-Wallis Test Summary** | |
| --- | --- |
| Total N | 476 |
| Test Statistic | 12.907^a^ |
| Degree Of Freedom | 4 |
| Asymptotic Sig.(2-sided test) | .012 |
| a. The test statistic is adjusted for ties. | |

| **Pairwise Comparisons of HouseholdIncome** | | | | | |
| --- | --- | --- | --- | --- | --- |
| Sample 1-Sample 2 | Test Statistic | Std. Error | Std. Test Statistic | Sig. | Adj. Sig.^a^ |
| Eur50000-99999-Eur25000-49999 | 2.262 | 15.957 | .142 | .887 | 1.000 |
| Eur50000-99999-Eur100000+ | -8.070 | 20.349 | -.397 | .692 | 1.000 |
| Eur50000-99999-Prefer Not to Say | -19.464 | 20.876 | -.932 | .351 | 1.000 |
| Eur50000-99999-Up to Eur24999 | 70.066 | 21.352 | 3.281 | .001 | .010 |
| Eur25000-49999-Eur100000+ | -5.808 | 19.486 | -.298 | .766 | 1.000 |
| Eur25000-49999-Prefer Not to Say | -17.201 | 20.036 | -.859 | .391 | 1.000 |
| Eur25000-49999-Up to Eur24999 | 67.803 | 20.532 | 3.302 | <.001 | .010 |
| Eur100000+-Prefer Not to Say | -11.394 | 23.684 | -.481 | .630 | 1.000 |
| Eur100000+-Up to Eur24999 | 61.995 | 24.104 | 2.572 | .010 | .101 |
| Prefer Not to Say-Up to Eur24999 | 50.602 | 24.551 | 2.061 | .039 | .393 |
| Each row tests the null hypothesis that the Sample 1 and Sample 2 distributions are the same.  Asymptotic significances (2-sided tests) are displayed. The significance level is .050. | | | | | |
| a. Significance values have been adjusted by the Bonferroni correction for multiple tests. | | | | | |

(12) AGE WITH CATEGORICAL RESPONSES

**Do you think these conditions can be treated with antibiotics? [HIV/AIDS] * AgeGroup**

| **Crosstab** | | | | | |
| --- | --- | --- | --- | --- | --- |
| Count | | | | | |
|  | | AgeGroup | | | Total |
|  |  | 18-34 | 35-54 | 55+ |  |
| Do you think these conditions can be treated with antibiotics? [HIV/AIDS] | Yes | 27 | 9 | 6 | 42 |
|  | No | 132 | 125 | 36 | 293 |
|  | Don't Know | 66 | 53 | 22 | 141 |
| Total | | 225 | 187 | 64 | 476 |

| **Chi-Square Tests** | | | |
| --- | --- | --- | --- |
|  | Value | df | Asymptotic Significance (2-sided) |
| Pearson Chi-Square | 8.050^a^ | 4 | .090 |
| Likelihood Ratio | 8.434 | 4 | .077 |
| Linear-by-Linear Association | 1.371 | 1 | .242 |
| N of Valid Cases | 476 |  |  |
| a. 0 cells (0.0%) have expected count less than 5. The minimum expected count is 5.65. | | | |

**Do you think these conditions can be treated with antibiotics?[Gonorrhoea] * AgeGroup**

| **Crosstab** | | | | | |
| --- | --- | --- | --- | --- | --- |
| Count | | | | | |
|  | | AgeGroup | | | Total |
|  |  | 18-34 | 35-54 | 55+ |  |
| Do you think these conditions can be treated with antibiotics?[Gonorrhoea] | Yes | 86 | 66 | 22 | 174 |
|  | No | 37 | 33 | 10 | 80 |
|  | Don't Know | 102 | 88 | 32 | 222 |
| Total | | 225 | 187 | 64 | 476 |

| **Chi-Square Tests** | | | |
| --- | --- | --- | --- |
|  | Value | df | Asymptotic Significance (2-sided) |
| Pearson Chi-Square | .729^a^ | 4 | .948 |
| Likelihood Ratio | .727 | 4 | .948 |
| Linear-by-Linear Association | .542 | 1 | .461 |
| N of Valid Cases | 476 |  |  |
| a. 0 cells (0.0%) have expected count less than 5. The minimum expected count is 10.76. | | | |

**Do you think these conditions can be treated with antibiotics? [Bladder infection or urinary tract infection (UTI) ] * AgeGroup**

| **Crosstab** | | | | | |
| --- | --- | --- | --- | --- | --- |
| Count | | | | | |
|  | | AgeGroup | | | Total |
|  |  | 18-34 | 35-54 | 55+ |  |
| Do you think these conditions can be treated with antibiotics? [Bladder infection or urinary tract infection (UTI) ] | Yes | 182 | 162 | 54 | 398 |
|  | No | 8 | 7 | 4 | 19 |
|  | Don't Know | 35 | 18 | 6 | 59 |
| Total | | 225 | 187 | 64 | 476 |

| **Chi-Square Tests** | | | |
| --- | --- | --- | --- |
|  | Value | df | Asymptotic Significance (2-sided) |
| Pearson Chi-Square | 4.803^a^ | 4 | .308 |
| Likelihood Ratio | 4.690 | 4 | .321 |
| Linear-by-Linear Association | 2.304 | 1 | .129 |
| N of Valid Cases | 476 |  |  |
| a. 1 cells (11.1%) have expected count less than 5. The minimum expected count is 2.55. | | | |

**Do you think these conditions can be treated with antibiotics? [Diarrhoea] * AgeGroup**

| **Crosstab** | | | | | |
| --- | --- | --- | --- | --- | --- |
| Count | | | | | |
|  | | AgeGroup | | | Total |
|  |  | 18-34 | 35-54 | 55+ |  |
| Do you think these conditions can be treated with antibiotics? [Diarrhoea] | Yes | 52 | 16 | 5 | 73 |
|  | No | 129 | 152 | 54 | 335 |
|  | Don't Know | 44 | 19 | 5 | 68 |
| Total | | 225 | 187 | 64 | 476 |

| **Chi-Square Tests** | | | |
| --- | --- | --- | --- |
|  | Value | df | Asymptotic Significance (2-sided) |
| Pearson Chi-Square | 35.697^a^ | 4 | <.001 |
| Likelihood Ratio | 36.373 | 4 | <.001 |
| Linear-by-Linear Association | .572 | 1 | .450 |
| N of Valid Cases | 476 |  |  |
| a. 0 cells (0.0%) have expected count less than 5. The minimum expected count is 9.14. | | | |

**Do you think these conditions can be treated with antibiotics? [Cold and flu] * AgeGroup**

| **Crosstab** | | | | | |
| --- | --- | --- | --- | --- | --- |
| Count | | | | | |
|  | | AgeGroup | | | Total |
|  |  | 18-34 | 35-54 | 55+ |  |
| Do you think these conditions can be treated with antibiotics? [Cold and flu] | Yes | 56 | 13 | 10 | 79 |
|  | No | 148 | 165 | 52 | 365 |
|  | Don't Know | 21 | 9 | 2 | 32 |
| Total | | 225 | 187 | 64 | 476 |

| **Chi-Square Tests** | | | |
| --- | --- | --- | --- |
|  | Value | df | Asymptotic Significance (2-sided) |
| Pearson Chi-Square | 31.285^a^ | 4 | <.001 |
| Likelihood Ratio | 33.195 | 4 | <.001 |
| Linear-by-Linear Association | 2.347 | 1 | .125 |
| N of Valid Cases | 476 |  |  |
| a. 1 cells (11.1%) have expected count less than 5. The minimum expected count is 4.30. | | | |

**Do you think these conditions can be treated with antibiotics? [Fever] * AgeGroup**

| **Crosstab** | | | | | |
| --- | --- | --- | --- | --- | --- |
| Count | | | | | |
|  | | AgeGroup | | | Total |
|  |  | 18-34 | 35-54 | 55+ |  |
| Do you think these conditions can be treated with antibiotics? [Fever] | Yes | 90 | 55 | 15 | 160 |
|  | No | 117 | 114 | 47 | 278 |
|  | Don't Know | 18 | 18 | 2 | 38 |
| Total | | 225 | 187 | 64 | 476 |

| **Chi-Square Tests** | | | |
| --- | --- | --- | --- |
|  | Value | df | Asymptotic Significance (2-sided) |
| Pearson Chi-Square | 12.474^a^ | 4 | .014 |
| Likelihood Ratio | 13.001 | 4 | .011 |
| Linear-by-Linear Association | 3.821 | 1 | .051 |
| N of Valid Cases | 476 |  |  |
| a. 0 cells (0.0%) have expected count less than 5. The minimum expected count is 5.11. | | | |

**Do you think these conditions can be treated with antibiotics? [Malaria] * AgeGroup**

| **Crosstab** | | | | | |
| --- | --- | --- | --- | --- | --- |
| Count | | | | | |
|  | | AgeGroup | | | Total |
|  |  | 18-34 | 35-54 | 55+ |  |
| Do you think these conditions can be treated with antibiotics? [Malaria] | Yes | 61 | 29 | 14 | 104 |
|  | No | 69 | 77 | 22 | 168 |
|  | Don't Know | 95 | 81 | 28 | 204 |
| Total | | 225 | 187 | 64 | 476 |

| **Chi-Square Tests** | | | |
| --- | --- | --- | --- |
|  | Value | df | Asymptotic Significance (2-sided) |
| Pearson Chi-Square | 9.549^a^ | 4 | .049 |
| Likelihood Ratio | 9.706 | 4 | .046 |
| Linear-by-Linear Association | 1.348 | 1 | .246 |
| N of Valid Cases | 476 |  |  |
| a. 0 cells (0.0%) have expected count less than 5. The minimum expected count is 13.98. | | | |

**Do you think these conditions can be treated with antibiotics? [Measles] * AgeGroup**

| **Crosstab** | | | | | |
| --- | --- | --- | --- | --- | --- |
| Count | | | | | |
|  | | AgeGroup | | | Total |
|  |  | 18-34 | 35-54 | 55+ |  |
| Do you think these conditions can be treated with antibiotics? [Measles] | Yes | 40 | 9 | 5 | 54 |
|  | No | 92 | 124 | 40 | 256 |
|  | Don't Know | 93 | 54 | 19 | 166 |
| Total | | 225 | 187 | 64 | 476 |

| **Chi-Square Tests** | | | |
| --- | --- | --- | --- |
|  | Value | df | Asymptotic Significance (2-sided) |
| Pearson Chi-Square | 34.375^a^ | 4 | <.001 |
| Likelihood Ratio | 35.406 | 4 | <.001 |
| Linear-by-Linear Association | .013 | 1 | .909 |
| N of Valid Cases | 476 |  |  |
| a. 0 cells (0.0%) have expected count less than 5. The minimum expected count is 7.26. | | | |

**Do you think these conditions can be treated with antibiotics? [Skin or wound infection] * AgeGroup**

| **Crosstab** | | | | | |
| --- | --- | --- | --- | --- | --- |
| Count | | | | | |
|  | | AgeGroup | | | Total |
|  |  | 18-34 | 35-54 | 55+ |  |
| Do you think these conditions can be treated with antibiotics? [Skin or wound infection] | Yes | 165 | 147 | 47 | 359 |
|  | No | 33 | 26 | 9 | 68 |
|  | Don't Know | 27 | 14 | 8 | 49 |
| Total | | 225 | 187 | 64 | 476 |

| **Chi-Square Tests** | | | |
| --- | --- | --- | --- |
|  | Value | df | Asymptotic Significance (2-sided) |
| Pearson Chi-Square | 2.830^a^ | 4 | .587 |
| Likelihood Ratio | 2.928 | 4 | .570 |
| Linear-by-Linear Association | .337 | 1 | .562 |
| N of Valid Cases | 476 |  |  |
| a. 0 cells (0.0%) have expected count less than 5. The minimum expected count is 6.59. | | | |

**Do you think these conditions can be treated with antibiotics? [Sore throat] * AgeGroup**

| **Crosstab** | | | | | |
| --- | --- | --- | --- | --- | --- |
| Count | | | | | |
|  | | AgeGroup | | | Total |
|  |  | 18-34 | 35-54 | 55+ |  |
| Do you think these conditions can be treated with antibiotics? [Sore throat] | Yes | 102 | 68 | 20 | 190 |
|  | No | 100 | 104 | 40 | 244 |
|  | Don't Know | 23 | 15 | 4 | 42 |
| Total | | 225 | 187 | 64 | 476 |

| **Chi-Square Tests** | | | |
| --- | --- | --- | --- |
|  | Value | df | Asymptotic Significance (2-sided) |
| Pearson Chi-Square | 8.873^a^ | 4 | .064 |
| Likelihood Ratio | 8.929 | 4 | .063 |
| Linear-by-Linear Association | 1.824 | 1 | .177 |
| N of Valid Cases | 476 |  |  |
| a. 0 cells (0.0%) have expected count less than 5. The minimum expected count is 5.65. | | | |

**Do you think these conditions can be treated with antibiotics? [Body aches] * AgeGroup**

| **Crosstab** | | | | | |
| --- | --- | --- | --- | --- | --- |
| Count | | | | | |
|  | | AgeGroup | | | Total |
|  |  | 18-34 | 35-54 | 55+ |  |
| Do you think these conditions can be treated with antibiotics? [Body aches] | Yes | 38 | 8 | 3 | 49 |
|  | No | 150 | 161 | 55 | 366 |
|  | Don't Know | 37 | 18 | 6 | 61 |
| Total | | 225 | 187 | 64 | 476 |

| **Chi-Square Tests** | | | |
| --- | --- | --- | --- |
|  | Value | df | Asymptotic Significance (2-sided) |
| Pearson Chi-Square | 28.224^a^ | 4 | <.001 |
| Likelihood Ratio | 29.100 | 4 | <.001 |
| Linear-by-Linear Association | 1.199 | 1 | .274 |
| N of Valid Cases | 476 |  |  |
| a. 0 cells (0.0%) have expected count less than 5. The minimum expected count is 6.59. | | | |

**Do you think these conditions can be treated with antibiotics? [Headaches] * AgeGroup**

| **Crosstab** | | | | | |
| --- | --- | --- | --- | --- | --- |
| Count | | | | | |
|  | | AgeGroup | | | Total |
|  |  | 18-34 | 35-54 | 55+ |  |
| Do you think these conditions can be treated with antibiotics? [Headaches] | Yes | 21 | 0 | 1 | 22 |
|  | No | 186 | 181 | 58 | 425 |
|  | Don't Know | 18 | 6 | 5 | 29 |
| Total | | 225 | 187 | 64 | 476 |

| **Chi-Square Tests** | | | |
| --- | --- | --- | --- |
|  | Value | df | Asymptotic Significance (2-sided) |
| Pearson Chi-Square | 27.250^a^ | 4 | <.001 |
| Likelihood Ratio | 34.240 | 4 | <.001 |
| Linear-by-Linear Association | 3.492 | 1 | .062 |
| N of Valid Cases | 476 |  |  |
| a. 2 cells (22.2%) have expected count less than 5. The minimum expected count is 2.96. | | | |

**When do you think you should stop taking antibiotics once you've begun treatment? * AgeGroup**

| **Crosstab** | | | | | |
| --- | --- | --- | --- | --- | --- |
| Count | | | | | |
|  | | AgeGroup | | | Total |
|  |  | 18-34 | 35-54 | 55+ |  |
| When do you think you should stop taking antibiotics once you've begun treatment? | When you have taken all of the antibiotics as directed | 206 | 183 | 64 | 453 |
|  | When you feel better | 18 | 4 | 0 | 22 |
|  | Don't know | 1 | 0 | 0 | 1 |
| Total | | 225 | 187 | 64 | 476 |

| **Chi-Square Tests** | | | |
| --- | --- | --- | --- |
|  | Value | df | Asymptotic Significance (2-sided) |
| Pearson Chi-Square | 12.731^a^ | 4 | .013 |
| Likelihood Ratio | 15.695 | 4 | .003 |
| Linear-by-Linear Association | 11.535 | 1 | <.001 |
| N of Valid Cases | 476 |  |  |
| a. 4 cells (44.4%) have expected count less than 5. The minimum expected count is .13. | | | |

**Do you think this statement is ‘true’ or ‘false’? “It’s okay to use antibiotics that were given to a friend or family member, as long as they were used to treat the same illness” * AgeGroup**

| **Crosstab** | | | | | |
| --- | --- | --- | --- | --- | --- |
| Count | | | | | |
|  | | AgeGroup | | | Total |
|  |  | 18-34 | 35-54 | 55+ |  |
| Do you think this statement is ‘true’ or ‘false’? “It’s okay to use antibiotics that were given to a friend or family member, as long as they were used to treat the same illness” | True | 18 | 10 | 0 | 28 |
|  | False | 197 | 173 | 62 | 432 |
|  | Don't Know | 10 | 4 | 2 | 16 |
| Total | | 225 | 187 | 64 | 476 |

| **Chi-Square Tests** | | | |
| --- | --- | --- | --- |
|  | Value | df | Asymptotic Significance (2-sided) |
| Pearson Chi-Square | 7.780^a^ | 4 | .100 |
| Likelihood Ratio | 11.421 | 4 | .022 |
| Linear-by-Linear Association | 1.636 | 1 | .201 |
| N of Valid Cases | 476 |  |  |
| a. 2 cells (22.2%) have expected count less than 5. The minimum expected count is 2.15. | | | |

**Do you think this statement is ‘true’ or ‘false’? “It’s okay to buy the same antibiotics, or request these from a doctor, if you’re sick and they helped you get better when you had the same symptoms before” * AgeGroup**

| **Crosstab** | | | | | |
| --- | --- | --- | --- | --- | --- |
| Count | | | | | |
|  | | AgeGroup | | | Total |
|  |  | 18-34 | 35-54 | 55+ |  |
| Do you think this statement is ‘true’ or ‘false’? “It’s okay to buy the same antibiotics, or request these from a doctor, if you’re sick and they helped you get better when you had the same symptoms before” | True | 65 | 32 | 16 | 113 |
|  | False | 152 | 149 | 48 | 349 |
|  | Don't Know | 8 | 6 | 0 | 14 |
| Total | | 225 | 187 | 64 | 476 |

| **Chi-Square Tests** | | | |
| --- | --- | --- | --- |
|  | Value | df | Asymptotic Significance (2-sided) |
| Pearson Chi-Square | 10.308^a^ | 4 | .036 |
| Likelihood Ratio | 12.381 | 4 | .015 |
| Linear-by-Linear Association | 1.075 | 1 | .300 |
| N of Valid Cases | 476 |  |  |
| a. 1 cells (11.1%) have expected count less than 5. The minimum expected count is 1.88. | | | |

**Please indicate whether you think the following statements are ‘true’ or ‘false’ [Antibiotic resistance occurs when your body becomes resistant to antibiotics and they no longer work as well.] * AgeGroup**

| **Crosstab** | | | | | |
| --- | --- | --- | --- | --- | --- |
| Count | | | | | |
|  | | AgeGroup | | | Total |
|  |  | 18-34 | 35-54 | 55+ |  |
| Please indicate whether you think the following statements are ‘true’ or ‘false’ [Antibiotic resistance occurs when your body becomes resistant to antibiotics and they no longer work as well.] | True | 162 | 142 | 51 | 355 |
|  | False | 38 | 33 | 5 | 76 |
|  | Don't Know | 25 | 12 | 8 | 45 |
| Total | | 225 | 187 | 64 | 476 |

| **Chi-Square Tests** | | | |
| --- | --- | --- | --- |
|  | Value | df | Asymptotic Significance (2-sided) |
| Pearson Chi-Square | 6.693^a^ | 4 | .153 |
| Likelihood Ratio | 7.442 | 4 | .114 |
| Linear-by-Linear Association | 1.204 | 1 | .273 |
| N of Valid Cases | 476 |  |  |
| a. 0 cells (0.0%) have expected count less than 5. The minimum expected count is 6.05. | | | |

**Please indicate whether you think the following statements are ‘true’ or ‘false’ [Many infections are becoming increasingly resistant to treatment by antibiotics ] * AgeGroup**

| **Crosstab** | | | | | |
| --- | --- | --- | --- | --- | --- |
| Count | | | | | |
|  | | AgeGroup | | | Total |
|  |  | 18-34 | 35-54 | 55+ |  |
| Please indicate whether you think the following statements are ‘true’ or ‘false’ [Many infections are becoming increasingly resistant to treatment by antibiotics ] | True | 153 | 153 | 49 | 355 |
|  | False | 15 | 6 | 6 | 27 |
|  | Don't Know | 57 | 28 | 9 | 94 |
| Total | | 225 | 187 | 64 | 476 |

| **Chi-Square Tests** | | | |
| --- | --- | --- | --- |
|  | Value | df | Asymptotic Significance (2-sided) |
| Pearson Chi-Square | 13.355^a^ | 4 | .010 |
| Likelihood Ratio | 13.474 | 4 | .009 |
| Linear-by-Linear Association | 6.899 | 1 | .009 |
| N of Valid Cases | 476 |  |  |
| a. 1 cells (11.1%) have expected count less than 5. The minimum expected count is 3.63. | | | |

**Please indicate whether you think the following statements are ‘true’ or ‘false’[If bacteria are resistant to antibiotics, it can be very difficult or impossible to treat the infections they cause] * AgeGroup**

| **Crosstab** | | | | | |
| --- | --- | --- | --- | --- | --- |
| Count | | | | | |
|  | | AgeGroup | | | Total |
|  |  | 18-34 | 35-54 | 55+ |  |
| Please indicate whether you think the following statements are ‘true’ or ‘false’[If bacteria are resistant to antibiotics, it can be very difficult or impossible to treat the infections they cause] | True | 172 | 164 | 45 | 381 |
|  | False | 16 | 9 | 9 | 34 |
|  | Don't Know | 37 | 14 | 10 | 61 |
| Total | | 225 | 187 | 64 | 476 |

| **Chi-Square Tests** | | | |
| --- | --- | --- | --- |
|  | Value | df | Asymptotic Significance (2-sided) |
| Pearson Chi-Square | 15.053^a^ | 4 | .005 |
| Likelihood Ratio | 14.799 | 4 | .005 |
| Linear-by-Linear Association | .605 | 1 | .437 |
| N of Valid Cases | 476 |  |  |
| a. 1 cells (11.1%) have expected count less than 5. The minimum expected count is 4.57. | | | |

**Please indicate whether you think the following statements are ‘true’ or ‘false’ [Antibiotic resistance is an issue that could affect me or my family] * AgeGroup**

| **Crosstab** | | | | | |
| --- | --- | --- | --- | --- | --- |
| Count | | | | | |
|  | | AgeGroup | | | Total |
|  |  | 18-34 | 35-54 | 55+ |  |
| Please indicate whether you think the following statements are ‘true’ or ‘false’ [Antibiotic resistance is an issue that could affect me or my family] | True | 177 | 158 | 52 | 387 |
|  | False | 13 | 13 | 2 | 28 |
|  | Don't Know | 35 | 16 | 10 | 61 |
| Total | | 225 | 187 | 64 | 476 |

| **Chi-Square Tests** | | | |
| --- | --- | --- | --- |
|  | Value | df | Asymptotic Significance (2-sided) |
| Pearson Chi-Square | 5.981^a^ | 4 | .201 |
| Likelihood Ratio | 6.375 | 4 | .173 |
| Linear-by-Linear Association | .950 | 1 | .330 |
| N of Valid Cases | 476 |  |  |
| a. 1 cells (11.1%) have expected count less than 5. The minimum expected count is 3.76. | | | |

**Please indicate whether you think the following statements are ‘true’ or ‘false’ [Antibiotic resistance is an issue in other countries but not here] * AgeGroup**

| **Crosstab** | | | | | |
| --- | --- | --- | --- | --- | --- |
| Count | | | | | |
|  | | AgeGroup | | | Total |
|  |  | 18-34 | 35-54 | 55+ |  |
| Please indicate whether you think the following statements are ‘true’ or ‘false’ [Antibiotic resistance is an issue in other countries but not here] | True | 11 | 3 | 3 | 17 |
|  | False | 174 | 161 | 48 | 383 |
|  | Don't Know | 40 | 23 | 13 | 76 |
| Total | | 225 | 187 | 64 | 476 |

| **Chi-Square Tests** | | | |
| --- | --- | --- | --- |
|  | Value | df | Asymptotic Significance (2-sided) |
| Pearson Chi-Square | 7.386^a^ | 4 | .117 |
| Likelihood Ratio | 7.833 | 4 | .098 |
| Linear-by-Linear Association | .022 | 1 | .883 |
| N of Valid Cases | 476 |  |  |
| a. 1 cells (11.1%) have expected count less than 5. The minimum expected count is 2.29. | | | |

**Please indicate whether you think the following statements are ‘true’ or ‘false’ [Antibiotic resistance is only a problem for people who take antibiotics regularly] * AgeGroup**

| **Crosstab** | | | | | |
| --- | --- | --- | --- | --- | --- |
| Count | | | | | |
|  | | AgeGroup | | | Total |
|  |  | 18-34 | 35-54 | 55+ |  |
| Please indicate whether you think the following statements are ‘true’ or ‘false’ [Antibiotic resistance is only a problem for people who take antibiotics regularly] | True | 48 | 38 | 10 | 96 |
|  | False | 141 | 116 | 38 | 295 |
|  | Don't Know | 36 | 33 | 16 | 85 |
| Total | | 225 | 187 | 64 | 476 |

| **Chi-Square Tests** | | | |
| --- | --- | --- | --- |
|  | Value | df | Asymptotic Significance (2-sided) |
| Pearson Chi-Square | 3.164^a^ | 4 | .531 |
| Likelihood Ratio | 3.029 | 4 | .553 |
| Linear-by-Linear Association | 2.285 | 1 | .131 |
| N of Valid Cases | 476 |  |  |
| a. 0 cells (0.0%) have expected count less than 5. The minimum expected count is 11.43. | | | |

**Please indicate whether you think the following statements are ‘true’ or ‘false’ [Bacteria which are resistant to antibiotics can be spread from person to person] * AgeGroup**

| **Crosstab** | | | | | |
| --- | --- | --- | --- | --- | --- |
| Count | | | | | |
|  | | AgeGroup | | | Total |
|  |  | 18-34 | 35-54 | 55+ |  |
| Please indicate whether you think the following statements are ‘true’ or ‘false’ [Bacteria which are resistant to antibiotics can be spread from person to person] | True | 129 | 122 | 39 | 290 |
|  | False | 27 | 20 | 11 | 58 |
|  | Don't Know | 69 | 45 | 14 | 128 |
| Total | | 225 | 187 | 64 | 476 |

| **Chi-Square Tests** | | | |
| --- | --- | --- | --- |
|  | Value | df | Asymptotic Significance (2-sided) |
| Pearson Chi-Square | 5.057^a^ | 4 | .282 |
| Likelihood Ratio | 4.928 | 4 | .295 |
| Linear-by-Linear Association | 2.187 | 1 | .139 |
| N of Valid Cases | 476 |  |  |
| a. 0 cells (0.0%) have expected count less than 5. The minimum expected count is 7.80. | | | |

**Please indicate whether you think the following statements are ‘true’ or ‘false’ [Antibiotic-resistant infections could make medical procedures like surgery, organ transplants and cancer treatment much more dangerous] * AgeGroup**

| **Crosstab** | | | | | |
| --- | --- | --- | --- | --- | --- |
| Count | | | | | |
|  | | AgeGroup | | | Total |
|  |  | 18-34 | 35-54 | 55+ |  |
| Please indicate whether you think the following statements are ‘true’ or ‘false’ [Antibiotic-resistant infections could make medical procedures like surgery, organ transplants and cancer treatment much more dangerous] | True | 177 | 153 | 48 | 378 |
|  | False | 5 | 4 | 7 | 16 |
|  | Don't Know | 43 | 30 | 9 | 82 |
| Total | | 225 | 187 | 64 | 476 |

| **Chi-Square Tests** | | | |
| --- | --- | --- | --- |
|  | Value | df | Asymptotic Significance (2-sided) |
| Pearson Chi-Square | 13.925^a^ | 4 | .008 |
| Likelihood Ratio | 10.075 | 4 | .039 |
| Linear-by-Linear Association | .192 | 1 | .661 |
| N of Valid Cases | 476 |  |  |
| a. 1 cells (11.1%) have expected count less than 5. The minimum expected count is 2.15. | | | |

(13) EDUCATION WITH CATEGORICAL RESPONSES

**Do you think these conditions can be treated with antibiotics? [HIV/AIDS] * EducationLevel**

| **Crosstab** | | | | | |
| --- | --- | --- | --- | --- | --- |
| Count | | | | | |
|  | | EducationLevel | | | Total |
|  |  | Low Education | Medium Education | High Education |  |
| Do you think these conditions can be treated with antibiotics? [HIV/AIDS] | Yes | 12 | 14 | 16 | 42 |
|  | No | 54 | 95 | 144 | 293 |
|  | Don't Know | 33 | 43 | 65 | 141 |
| Total | | 99 | 152 | 225 | 476 |

| **Chi-Square Tests** | | | |
| --- | --- | --- | --- |
|  | Value | df | Asymptotic Significance (2-sided) |
| Pearson Chi-Square | 3.617^a^ | 4 | .460 |
| Likelihood Ratio | 3.554 | 4 | .470 |
| Linear-by-Linear Association | .032 | 1 | .857 |
| N of Valid Cases | 476 |  |  |
| a. 0 cells (0.0%) have expected count less than 5. The minimum expected count is 8.74. | | | |

**Do you think these conditions can be treated with antibiotics?[Gonorrhoea] * EducationLevel**

| **Crosstab** | | | | | |
| --- | --- | --- | --- | --- | --- |
| Count | | | | | |
|  | | EducationLevel | | | Total |
|  |  | Low Education | Medium Education | High Education |  |
| Do you think these conditions can be treated with antibiotics?[Gonorrhoea] | Yes | 31 | 46 | 97 | 174 |
|  | No | 14 | 28 | 38 | 80 |
|  | Don't Know | 54 | 78 | 90 | 222 |
| Total | | 99 | 152 | 225 | 476 |

| **Chi-Square Tests** | | | |
| --- | --- | --- | --- |
|  | Value | df | Asymptotic Significance (2-sided) |
| Pearson Chi-Square | 9.857^a^ | 4 | .043 |
| Likelihood Ratio | 9.887 | 4 | .042 |
| Linear-by-Linear Association | 7.620 | 1 | .006 |
| N of Valid Cases | 476 |  |  |
| a. 0 cells (0.0%) have expected count less than 5. The minimum expected count is 16.64. | | | |

**Do you think these conditions can be treated with antibiotics? [Bladder infection or urinary tract infection (UTI) ] * EducationLevel**

| **Crosstab** | | | | | |
| --- | --- | --- | --- | --- | --- |
| Count | | | | | |
|  | | EducationLevel | | | Total |
|  |  | Low Education | Medium Education | High Education |  |
| Do you think these conditions can be treated with antibiotics? [Bladder infection or urinary tract infection (UTI) ] | Yes | 80 | 122 | 196 | 398 |
|  | No | 7 | 8 | 4 | 19 |
|  | Don't Know | 12 | 22 | 25 | 59 |
| Total | | 99 | 152 | 225 | 476 |

| **Chi-Square Tests** | | | |
| --- | --- | --- | --- |
|  | Value | df | Asymptotic Significance (2-sided) |
| Pearson Chi-Square | 7.191^a^ | 4 | .126 |
| Likelihood Ratio | 7.403 | 4 | .116 |
| Linear-by-Linear Association | 1.334 | 1 | .248 |
| N of Valid Cases | 476 |  |  |
| a. 1 cells (11.1%) have expected count less than 5. The minimum expected count is 3.95. | | | |

**Do you think these conditions can be treated with antibiotics? [Diarrhoea] * EducationLevel**

| **Crosstab** | | | | | |
| --- | --- | --- | --- | --- | --- |
| Count | | | | | |
|  | | EducationLevel | | | Total |
|  |  | Low Education | Medium Education | High Education |  |
| Do you think these conditions can be treated with antibiotics? [Diarrhoea] | Yes | 10 | 20 | 43 | 73 |
|  | No | 76 | 111 | 148 | 335 |
|  | Don't Know | 13 | 21 | 34 | 68 |
| Total | | 99 | 152 | 225 | 476 |

| **Chi-Square Tests** | | | |
| --- | --- | --- | --- |
|  | Value | df | Asymptotic Significance (2-sided) |
| Pearson Chi-Square | 5.956^a^ | 4 | .202 |
| Likelihood Ratio | 6.069 | 4 | .194 |
| Linear-by-Linear Association | 1.322 | 1 | .250 |
| N of Valid Cases | 476 |  |  |
| a. 0 cells (0.0%) have expected count less than 5. The minimum expected count is 14.14. | | | |

**Do you think these conditions can be treated with antibiotics? [Cold and flu] * EducationLevel**

| **Crosstab** | | | | | |
| --- | --- | --- | --- | --- | --- |
| Count | | | | | |
|  | | EducationLevel | | | Total |
|  |  | Low Education | Medium Education | High Education |  |
| Do you think these conditions can be treated with antibiotics? [Cold and flu] | Yes | 20 | 23 | 36 | 79 |
|  | No | 72 | 121 | 172 | 365 |
|  | Don't Know | 7 | 8 | 17 | 32 |
| Total | | 99 | 152 | 225 | 476 |

| **Chi-Square Tests** | | | |
| --- | --- | --- | --- |
|  | Value | df | Asymptotic Significance (2-sided) |
| Pearson Chi-Square | 2.125^a^ | 4 | .713 |
| Likelihood Ratio | 2.112 | 4 | .715 |
| Linear-by-Linear Association | .638 | 1 | .425 |
| N of Valid Cases | 476 |  |  |
| a. 0 cells (0.0%) have expected count less than 5. The minimum expected count is 6.66. | | | |

**Do you think these conditions can be treated with antibiotics? [Fever] * EducationLevel**

| **Crosstab** | | | | | |
| --- | --- | --- | --- | --- | --- |
| Count | | | | | |
|  | | EducationLevel | | | Total |
|  |  | Low Education | Medium Education | High Education |  |
| Do you think these conditions can be treated with antibiotics? [Fever] | Yes | 37 | 42 | 81 | 160 |
|  | No | 55 | 97 | 126 | 278 |
|  | Don't Know | 7 | 13 | 18 | 38 |
| Total | | 99 | 152 | 225 | 476 |

| **Chi-Square Tests** | | | |
| --- | --- | --- | --- |
|  | Value | df | Asymptotic Significance (2-sided) |
| Pearson Chi-Square | 3.703^a^ | 4 | .448 |
| Likelihood Ratio | 3.771 | 4 | .438 |
| Linear-by-Linear Association | .005 | 1 | .944 |
| N of Valid Cases | 476 |  |  |
| a. 0 cells (0.0%) have expected count less than 5. The minimum expected count is 7.90. | | | |

**Do you think these conditions can be treated with antibiotics? [Malaria] * EducationLevel**

| **Crosstab** | | | | | |
| --- | --- | --- | --- | --- | --- |
| Count | | | | | |
|  | | EducationLevel | | | Total |
|  |  | Low Education | Medium Education | High Education |  |
| Do you think these conditions can be treated with antibiotics? [Malaria] | Yes | 27 | 30 | 47 | 104 |
|  | No | 21 | 52 | 95 | 168 |
|  | Don't Know | 51 | 70 | 83 | 204 |
| Total | | 99 | 152 | 225 | 476 |

| **Chi-Square Tests** | | | |
| --- | --- | --- | --- |
|  | Value | df | Asymptotic Significance (2-sided) |
| Pearson Chi-Square | 14.375^a^ | 4 | .006 |
| Likelihood Ratio | 15.021 | 4 | .005 |
| Linear-by-Linear Association | 1.192 | 1 | .275 |
| N of Valid Cases | 476 |  |  |
| a. 0 cells (0.0%) have expected count less than 5. The minimum expected count is 21.63. | | | |

**Do you think these conditions can be treated with antibiotics? [Measles] * EducationLevel**

| **Crosstab** | | | | | |
| --- | --- | --- | --- | --- | --- |
| Count | | | | | |
|  | | EducationLevel | | | Total |
|  |  | Low Education | Medium Education | High Education |  |
| Do you think these conditions can be treated with antibiotics? [Measles] | Yes | 11 | 21 | 22 | 54 |
|  | No | 47 | 84 | 125 | 256 |
|  | Don't Know | 41 | 47 | 78 | 166 |
| Total | | 99 | 152 | 225 | 476 |

| **Chi-Square Tests** | | | |
| --- | --- | --- | --- |
|  | Value | df | Asymptotic Significance (2-sided) |
| Pearson Chi-Square | 4.134^a^ | 4 | .388 |
| Likelihood Ratio | 4.091 | 4 | .394 |
| Linear-by-Linear Association | .112 | 1 | .738 |
| N of Valid Cases | 476 |  |  |
| a. 0 cells (0.0%) have expected count less than 5. The minimum expected count is 11.23. | | | |

**Do you think these conditions can be treated with antibiotics? [Skin or wound infection] * EducationLevel**

| **Crosstab** | | | | | |
| --- | --- | --- | --- | --- | --- |
| Count | | | | | |
|  | | EducationLevel | | | Total |
|  |  | Low Education | Medium Education | High Education |  |
| Do you think these conditions can be treated with antibiotics? [Skin or wound infection] | Yes | 71 | 112 | 176 | 359 |
|  | No | 19 | 24 | 25 | 68 |
|  | Don't Know | 9 | 16 | 24 | 49 |
| Total | | 99 | 152 | 225 | 476 |

| **Chi-Square Tests** | | | |
| --- | --- | --- | --- |
|  | Value | df | Asymptotic Significance (2-sided) |
| Pearson Chi-Square | 4.148^a^ | 4 | .386 |
| Likelihood Ratio | 4.115 | 4 | .391 |
| Linear-by-Linear Association | .500 | 1 | .479 |
| N of Valid Cases | 476 |  |  |
| a. 0 cells (0.0%) have expected count less than 5. The minimum expected count is 10.19. | | | |

**Do you think these conditions can be treated with antibiotics? [Sore throat] * EducationLevel**

| **Crosstab** | | | | | |
| --- | --- | --- | --- | --- | --- |
| Count | | | | | |
|  | | EducationLevel | | | Total |
|  |  | Low Education | Medium Education | High Education |  |
| Do you think these conditions can be treated with antibiotics? [Sore throat] | Yes | 46 | 62 | 82 | 190 |
|  | No | 46 | 79 | 119 | 244 |
|  | Don't Know | 7 | 11 | 24 | 42 |
| Total | | 99 | 152 | 225 | 476 |

| **Chi-Square Tests** | | | |
| --- | --- | --- | --- |
|  | Value | df | Asymptotic Significance (2-sided) |
| Pearson Chi-Square | 3.992^a^ | 4 | .407 |
| Likelihood Ratio | 3.972 | 4 | .410 |
| Linear-by-Linear Association | 3.577 | 1 | .059 |
| N of Valid Cases | 476 |  |  |
| a. 0 cells (0.0%) have expected count less than 5. The minimum expected count is 8.74. | | | |

**Do you think these conditions can be treated with antibiotics? [Body aches] * EducationLevel**

| **Crosstab** | | | | | |
| --- | --- | --- | --- | --- | --- |
| Count | | | | | |
|  | | EducationLevel | | | Total |
|  |  | Low Education | Medium Education | High Education |  |
| Do you think these conditions can be treated with antibiotics? [Body aches] | Yes | 14 | 19 | 16 | 49 |
|  | No | 72 | 117 | 177 | 366 |
|  | Don't Know | 13 | 16 | 32 | 61 |
| Total | | 99 | 152 | 225 | 476 |

| **Chi-Square Tests** | | | |
| --- | --- | --- | --- |
|  | Value | df | Asymptotic Significance (2-sided) |
| Pearson Chi-Square | 5.649^a^ | 4 | .227 |
| Likelihood Ratio | 5.756 | 4 | .218 |
| Linear-by-Linear Association | 2.847 | 1 | .092 |
| N of Valid Cases | 476 |  |  |
| a. 0 cells (0.0%) have expected count less than 5. The minimum expected count is 10.19. | | | |

**Do you think these conditions can be treated with antibiotics? [Headaches] * EducationLevel**

| **Crosstab** | | | | | |
| --- | --- | --- | --- | --- | --- |
| Count | | | | | |
|  | | EducationLevel | | | Total |
|  |  | Low Education | Medium Education | High Education |  |
| Do you think these conditions can be treated with antibiotics? [Headaches] | Yes | 9 | 6 | 7 | 22 |
|  | No | 83 | 139 | 203 | 425 |
|  | Don't Know | 7 | 7 | 15 | 29 |
| Total | | 99 | 152 | 225 | 476 |

| **Chi-Square Tests** | | | |
| --- | --- | --- | --- |
|  | Value | df | Asymptotic Significance (2-sided) |
| Pearson Chi-Square | 6.799^a^ | 4 | .147 |
| Likelihood Ratio | 6.048 | 4 | .196 |
| Linear-by-Linear Association | 2.131 | 1 | .144 |
| N of Valid Cases | 476 |  |  |
| a. 1 cells (11.1%) have expected count less than 5. The minimum expected count is 4.58. | | | |

**When do you think you should stop taking antibiotics once you've begun treatment? * EducationLevel**

| **Crosstab** | | | | | |
| --- | --- | --- | --- | --- | --- |
| Count | | | | | |
|  | | EducationLevel | | | Total |
|  |  | Low Education | Medium Education | High Education |  |
| When do you think you should stop taking antibiotics once you've begun treatment? | When you have taken all of the antibiotics as directed | 91 | 147 | 215 | 453 |
|  | When you feel better | 8 | 5 | 9 | 22 |
|  | Don't know | 0 | 0 | 1 | 1 |
| Total | | 99 | 152 | 225 | 476 |

| **Chi-Square Tests** | | | |
| --- | --- | --- | --- |
|  | Value | df | Asymptotic Significance (2-sided) |
| Pearson Chi-Square | 4.602^a^ | 4 | .331 |
| Likelihood Ratio | 4.589 | 4 | .332 |
| Linear-by-Linear Association | .741 | 1 | .389 |
| N of Valid Cases | 476 |  |  |
| a. 4 cells (44.4%) have expected count less than 5. The minimum expected count is .21. | | | |

**Do you think this statement is ‘true’ or ‘false’? “It’s okay to use antibiotics that were given to a friend or family member, as long as they were used to treat the same illness” * EducationLevel**

| **Crosstab** | | | | | |
| --- | --- | --- | --- | --- | --- |
| Count | | | | | |
|  | | EducationLevel | | | Total |
|  |  | Low Education | Medium Education | High Education |  |
| Do you think this statement is ‘true’ or ‘false’? “It’s okay to use antibiotics that were given to a friend or family member, as long as they were used to treat the same illness” | True | 7 | 12 | 9 | 28 |
|  | False | 84 | 137 | 211 | 432 |
|  | Don't Know | 8 | 3 | 5 | 16 |
| Total | | 99 | 152 | 225 | 476 |

| **Chi-Square Tests** | | | |
| --- | --- | --- | --- |
|  | Value | df | Asymptotic Significance (2-sided) |
| Pearson Chi-Square | 11.552^a^ | 4 | .021 |
| Likelihood Ratio | 10.017 | 4 | .040 |
| Linear-by-Linear Association | .124 | 1 | .724 |
| N of Valid Cases | 476 |  |  |
| a. 1 cells (11.1%) have expected count less than 5. The minimum expected count is 3.33. | | | |

**Do you think this statement is ‘true’ or ‘false’? “It’s okay to buy the same antibiotics, or request these from a doctor, if you’re sick and they helped you get better when you had the same symptoms before” * EducationLevel**

| **Crosstab** | | | | | |
| --- | --- | --- | --- | --- | --- |
| Count | | | | | |
|  | | EducationLevel | | | Total |
|  |  | Low Education | Medium Education | High Education |  |
| Do you think this statement is ‘true’ or ‘false’? “It’s okay to buy the same antibiotics, or request these from a doctor, if you’re sick and they helped you get better when you had the same symptoms before” | True | 39 | 35 | 39 | 113 |
|  | False | 55 | 116 | 178 | 349 |
|  | Don't Know | 5 | 1 | 8 | 14 |
| Total | | 99 | 152 | 225 | 476 |

| **Chi-Square Tests** | | | |
| --- | --- | --- | --- |
|  | Value | df | Asymptotic Significance (2-sided) |
| Pearson Chi-Square | 24.099^a^ | 4 | <.001 |
| Likelihood Ratio | 24.126 | 4 | <.001 |
| Linear-by-Linear Association | 13.100 | 1 | <.001 |
| N of Valid Cases | 476 |  |  |
| a. 2 cells (22.2%) have expected count less than 5. The minimum expected count is 2.91. | | | |

**Please indicate whether you think the following statements are ‘true’ or ‘false’ [Antibiotic resistance occurs when your body becomes resistant to antibiotics and they no longer work as well.] * EducationLevel**

| **Crosstab** | | | | | |
| --- | --- | --- | --- | --- | --- |
| Count | | | | | |
|  | | EducationLevel | | | Total |
|  |  | Low Education | Medium Education | High Education |  |
| Please indicate whether you think the following statements are ‘true’ or ‘false’ [Antibiotic resistance occurs when your body becomes resistant to antibiotics and they no longer work as well.] | True | 85 | 111 | 159 | 355 |
|  | False | 2 | 28 | 46 | 76 |
|  | Don't Know | 12 | 13 | 20 | 45 |
| Total | | 99 | 152 | 225 | 476 |

| **Chi-Square Tests** | | | |
| --- | --- | --- | --- |
|  | Value | df | Asymptotic Significance (2-sided) |
| Pearson Chi-Square | 18.611^a^ | 4 | <.001 |
| Likelihood Ratio | 25.509 | 4 | <.001 |
| Linear-by-Linear Association | 2.127 | 1 | .145 |
| N of Valid Cases | 476 |  |  |
| a. 0 cells (0.0%) have expected count less than 5. The minimum expected count is 9.36. | | | |

**Please indicate whether you think the following statements are ‘true’ or ‘false’ [Many infections are becoming increasingly resistant to treatment by antibiotics ] * EducationLevel**

| **Crosstab** | | | | | |
| --- | --- | --- | --- | --- | --- |
| Count | | | | | |
|  | | EducationLevel | | | Total |
|  |  | Low Education | Medium Education | High Education |  |
| Please indicate whether you think the following statements are ‘true’ or ‘false’ [Many infections are becoming increasingly resistant to treatment by antibiotics ] | True | 62 | 111 | 182 | 355 |
|  | False | 8 | 9 | 10 | 27 |
|  | Don't Know | 29 | 32 | 33 | 94 |
| Total | | 99 | 152 | 225 | 476 |

| **Chi-Square Tests** | | | |
| --- | --- | --- | --- |
|  | Value | df | Asymptotic Significance (2-sided) |
| Pearson Chi-Square | 12.414^a^ | 4 | .015 |
| Likelihood Ratio | 12.112 | 4 | .017 |
| Linear-by-Linear Association | 11.758 | 1 | <.001 |
| N of Valid Cases | 476 |  |  |
| a. 0 cells (0.0%) have expected count less than 5. The minimum expected count is 5.62. | | | |

**Please indicate whether you think the following statements are ‘true’ or ‘false’[If bacteria are resistant to antibiotics, it can be very difficult or impossible to treat the infections they cause] * EducationLevel**

| **Crosstab** | | | | | |
| --- | --- | --- | --- | --- | --- |
| Count | | | | | |
|  | | EducationLevel | | | Total |
|  |  | Low Education | Medium Education | High Education |  |
| Please indicate whether you think the following statements are ‘true’ or ‘false’[If bacteria are resistant to antibiotics, it can be very difficult or impossible to treat the infections they cause] | True | 69 | 123 | 189 | 381 |
|  | False | 10 | 9 | 15 | 34 |
|  | Don't Know | 20 | 20 | 21 | 61 |
| Total | | 99 | 152 | 225 | 476 |

| **Chi-Square Tests** | | | |
| --- | --- | --- | --- |
|  | Value | df | Asymptotic Significance (2-sided) |
| Pearson Chi-Square | 9.738^a^ | 4 | .045 |
| Likelihood Ratio | 9.263 | 4 | .055 |
| Linear-by-Linear Association | 8.472 | 1 | .004 |
| N of Valid Cases | 476 |  |  |
| a. 0 cells (0.0%) have expected count less than 5. The minimum expected count is 7.07. | | | |

**Please indicate whether you think the following statements are ‘true’ or ‘false’ [Antibiotic resistance is an issue that could affect me or my family] * EducationLevel**

| **Crosstab** | | | | | |
| --- | --- | --- | --- | --- | --- |
| Count | | | | | |
|  | | EducationLevel | | | Total |
|  |  | Low Education | Medium Education | High Education |  |
| Please indicate whether you think the following statements are ‘true’ or ‘false’ [Antibiotic resistance is an issue that could affect me or my family] | True | 77 | 121 | 189 | 387 |
|  | False | 6 | 6 | 16 | 28 |
|  | Don't Know | 16 | 25 | 20 | 61 |
| Total | | 99 | 152 | 225 | 476 |

| **Chi-Square Tests** | | | |
| --- | --- | --- | --- |
|  | Value | df | Asymptotic Significance (2-sided) |
| Pearson Chi-Square | 7.093^a^ | 4 | .131 |
| Likelihood Ratio | 7.317 | 4 | .120 |
| Linear-by-Linear Association | 3.426 | 1 | .064 |
| N of Valid Cases | 476 |  |  |
| a. 0 cells (0.0%) have expected count less than 5. The minimum expected count is 5.82. | | | |

**Please indicate whether you think the following statements are ‘true’ or ‘false’ [Antibiotic resistance is an issue in other countries but not here] * EducationLevel**

| **Crosstab** | | | | | |
| --- | --- | --- | --- | --- | --- |
| Count | | | | | |
|  | | EducationLevel | | | Total |
|  |  | Low Education | Medium Education | High Education |  |
| Please indicate whether you think the following statements are ‘true’ or ‘false’ [Antibiotic resistance is an issue in other countries but not here] | True | 7 | 2 | 8 | 17 |
|  | False | 67 | 120 | 196 | 383 |
|  | Don't Know | 25 | 30 | 21 | 76 |
| Total | | 99 | 152 | 225 | 476 |

| **Chi-Square Tests** | | | |
| --- | --- | --- | --- |
|  | Value | df | Asymptotic Significance (2-sided) |
| Pearson Chi-Square | 21.751^a^ | 4 | <.001 |
| Likelihood Ratio | 21.963 | 4 | <.001 |
| Linear-by-Linear Association | 8.108 | 1 | .004 |
| N of Valid Cases | 476 |  |  |
| a. 1 cells (11.1%) have expected count less than 5. The minimum expected count is 3.54. | | | |

**Please indicate whether you think the following statements are ‘true’ or ‘false’ [Antibiotic resistance is only a problem for people who take antibiotics regularly] * EducationLevel**

| **Crosstab** | | | | | |
| --- | --- | --- | --- | --- | --- |
| Count | | | | | |
|  | | EducationLevel | | | Total |
|  |  | Low Education | Medium Education | High Education |  |
| Please indicate whether you think the following statements are ‘true’ or ‘false’ [Antibiotic resistance is only a problem for people who take antibiotics regularly] | True | 27 | 29 | 40 | 96 |
|  | False | 46 | 93 | 156 | 295 |
|  | Don't Know | 26 | 30 | 29 | 85 |
| Total | | 99 | 152 | 225 | 476 |

| **Chi-Square Tests** | | | |
| --- | --- | --- | --- |
|  | Value | df | Asymptotic Significance (2-sided) |
| Pearson Chi-Square | 16.356^a^ | 4 | .003 |
| Likelihood Ratio | 16.256 | 4 | .003 |
| Linear-by-Linear Association | .454 | 1 | .500 |
| N of Valid Cases | 476 |  |  |
| a. 0 cells (0.0%) have expected count less than 5. The minimum expected count is 17.68. | | | |

**Please indicate whether you think the following statements are ‘true’ or ‘false’ [Bacteria which are resistant to antibiotics can be spread from person to person] * EducationLevel**

| **Crosstab** | | | | | |
| --- | --- | --- | --- | --- | --- |
| Count | | | | | |
|  | | EducationLevel | | | Total |
|  |  | Low Education | Medium Education | High Education |  |
| Please indicate whether you think the following statements are ‘true’ or ‘false’ [Bacteria which are resistant to antibiotics can be spread from person to person] | True | 49 | 95 | 146 | 290 |
|  | False | 15 | 17 | 26 | 58 |
|  | Don't Know | 35 | 40 | 53 | 128 |
| Total | | 99 | 152 | 225 | 476 |

| **Chi-Square Tests** | | | |
| --- | --- | --- | --- |
|  | Value | df | Asymptotic Significance (2-sided) |
| Pearson Chi-Square | 7.264^a^ | 4 | .123 |
| Likelihood Ratio | 7.153 | 4 | .128 |
| Linear-by-Linear Association | 5.875 | 1 | .015 |
| N of Valid Cases | 476 |  |  |
| a. 0 cells (0.0%) have expected count less than 5. The minimum expected count is 12.06. | | | |

**Please indicate whether you think the following statements are ‘true’ or ‘false’ [Antibiotic-resistant infections could make medical procedures like surgery, organ transplants and cancer treatment much more dangerous] * EducationLevel**

| **Crosstab** | | | | | |
| --- | --- | --- | --- | --- | --- |
| Count | | | | | |
|  | | EducationLevel | | | Total |
|  |  | Low Education | Medium Education | High Education |  |
| Please indicate whether you think the following statements are ‘true’ or ‘false’ [Antibiotic-resistant infections could make medical procedures like surgery, organ transplants and cancer treatment much more dangerous] | True | 71 | 120 | 187 | 378 |
|  | False | 4 | 7 | 5 | 16 |
|  | Don't Know | 24 | 25 | 33 | 82 |
| Total | | 99 | 152 | 225 | 476 |

| **Chi-Square Tests** | | | |
| --- | --- | --- | --- |
|  | Value | df | Asymptotic Significance (2-sided) |
| Pearson Chi-Square | 6.572^a^ | 4 | .160 |
| Likelihood Ratio | 6.355 | 4 | .174 |
| Linear-by-Linear Association | 4.861 | 1 | .027 |
| N of Valid Cases | 476 |  |  |
| a. 1 cells (11.1%) have expected count less than 5. The minimum expected count is 3.33. | | | |

(14) GENDER WITH CATEGORICAL RESPONSES

**Do you think these conditions can be treated with antibiotics? [HIV/AIDS] * What is your gender?**

| **Crosstab** | | | | |
| --- | --- | --- | --- | --- |
| Count | | | | |
|  | | What is your gender? | | Total |
|  |  | Male | Female |  |
| Do you think these conditions can be treated with antibiotics? [HIV/AIDS] | Yes | 11 | 31 | 42 |
|  | No | 118 | 175 | 293 |
|  | Don't Know | 62 | 79 | 141 |
| Total | | 191 | 285 | 476 |

| **Chi-Square Tests** | | | |
| --- | --- | --- | --- |
|  | Value | df | Asymptotic Significance (2-sided) |
| Pearson Chi-Square | 4.266^a^ | 2 | .119 |
| Likelihood Ratio | 4.450 | 2 | .108 |
| Linear-by-Linear Association | 3.251 | 1 | .071 |
| N of Valid Cases | 476 |  |  |
| a. 0 cells (0.0%) have expected count less than 5. The minimum expected count is 16.85. | | | |

**Do you think these conditions can be treated with antibiotics?[Gonorrhoea] * What is your gender?**

| **Crosstab** | | | | |
| --- | --- | --- | --- | --- |
| Count | | | | |
|  | | What is your gender? | | Total |
|  |  | Male | Female |  |
| Do you think these conditions can be treated with antibiotics?[Gonorrhoea] | Yes | 71 | 103 | 174 |
|  | No | 28 | 52 | 80 |
|  | Don't Know | 92 | 130 | 222 |
| Total | | 191 | 285 | 476 |

| **Chi-Square Tests** | | | |
| --- | --- | --- | --- |
|  | Value | df | Asymptotic Significance (2-sided) |
| Pearson Chi-Square | 1.068^a^ | 2 | .586 |
| Likelihood Ratio | 1.082 | 2 | .582 |
| Linear-by-Linear Association | .032 | 1 | .858 |
| N of Valid Cases | 476 |  |  |
| a. 0 cells (0.0%) have expected count less than 5. The minimum expected count is 32.10. | | | |

**Do you think these conditions can be treated with antibiotics? [Bladder infection or urinary tract infection (UTI) ] * What is your gender?**

| **Crosstab** | | | | |
| --- | --- | --- | --- | --- |
| Count | | | | |
|  | | What is your gender? | | Total |
|  |  | Male | Female |  |
| Do you think these conditions can be treated with antibiotics? [Bladder infection or urinary tract infection (UTI) ] | Yes | 150 | 248 | 398 |
|  | No | 4 | 15 | 19 |
|  | Don't Know | 37 | 22 | 59 |
| Total | | 191 | 285 | 476 |

| **Chi-Square Tests** | | | |
| --- | --- | --- | --- |
|  | Value | df | Asymptotic Significance (2-sided) |
| Pearson Chi-Square | 16.389^a^ | 2 | <.001 |
| Likelihood Ratio | 16.334 | 2 | <.001 |
| Linear-by-Linear Association | 10.217 | 1 | .001 |
| N of Valid Cases | 476 |  |  |
| a. 0 cells (0.0%) have expected count less than 5. The minimum expected count is 7.62. | | | |

**Do you think these conditions can be treated with antibiotics? [Diarrhoea] * What is your gender?**

| **Crosstab** | | | | |
| --- | --- | --- | --- | --- |
| Count | | | | |
|  | | What is your gender? | | Total |
|  |  | Male | Female |  |
| Do you think these conditions can be treated with antibiotics? [Diarrhoea] | Yes | 35 | 38 | 73 |
|  | No | 112 | 223 | 335 |
|  | Don't Know | 44 | 24 | 68 |
| Total | | 191 | 285 | 476 |

| **Chi-Square Tests** | | | |
| --- | --- | --- | --- |
|  | Value | df | Asymptotic Significance (2-sided) |
| Pearson Chi-Square | 25.205^a^ | 2 | <.001 |
| Likelihood Ratio | 24.891 | 2 | <.001 |
| Linear-by-Linear Association | 3.570 | 1 | .059 |
| N of Valid Cases | 476 |  |  |
| a. 0 cells (0.0%) have expected count less than 5. The minimum expected count is 27.29. | | | |

**Do you think these conditions can be treated with antibiotics? [Cold and flu] * What is your gender?**

| **Crosstab** | | | | |
| --- | --- | --- | --- | --- |
| Count | | | | |
|  | | What is your gender? | | Total |
|  |  | Male | Female |  |
| Do you think these conditions can be treated with antibiotics? [Cold and flu] | Yes | 41 | 38 | 79 |
|  | No | 132 | 233 | 365 |
|  | Don't Know | 18 | 14 | 32 |
| Total | | 191 | 285 | 476 |

| **Chi-Square Tests** | | | |
| --- | --- | --- | --- |
|  | Value | df | Asymptotic Significance (2-sided) |
| Pearson Chi-Square | 10.405^a^ | 2 | .006 |
| Likelihood Ratio | 10.246 | 2 | .006 |
| Linear-by-Linear Association | .670 | 1 | .413 |
| N of Valid Cases | 476 |  |  |
| a. 0 cells (0.0%) have expected count less than 5. The minimum expected count is 12.84. | | | |

**Do you think these conditions can be treated with antibiotics? [Fever] * What is your gender?**

| **Crosstab** | | | | |
| --- | --- | --- | --- | --- |
| Count | | | | |
|  | | What is your gender? | | Total |
|  |  | Male | Female |  |
| Do you think these conditions can be treated with antibiotics? [Fever] | Yes | 73 | 87 | 160 |
|  | No | 98 | 180 | 278 |
|  | Don't Know | 20 | 18 | 38 |
| Total | | 191 | 285 | 476 |

| **Chi-Square Tests** | | | |
| --- | --- | --- | --- |
|  | Value | df | Asymptotic Significance (2-sided) |
| Pearson Chi-Square | 7.236^a^ | 2 | .027 |
| Likelihood Ratio | 7.197 | 2 | .027 |
| Linear-by-Linear Association | .408 | 1 | .523 |
| N of Valid Cases | 476 |  |  |
| a. 0 cells (0.0%) have expected count less than 5. The minimum expected count is 15.25. | | | |

**Do you think these conditions can be treated with antibiotics? [Malaria] * What is your gender?**

| **Crosstab** | | | | |
| --- | --- | --- | --- | --- |
| Count | | | | |
|  | | What is your gender? | | Total |
|  |  | Male | Female |  |
| Do you think these conditions can be treated with antibiotics? [Malaria] | Yes | 36 | 68 | 104 |
|  | No | 70 | 98 | 168 |
|  | Don't Know | 85 | 119 | 204 |
| Total | | 191 | 285 | 476 |

| **Chi-Square Tests** | | | |
| --- | --- | --- | --- |
|  | Value | df | Asymptotic Significance (2-sided) |
| Pearson Chi-Square | 1.682^a^ | 2 | .431 |
| Likelihood Ratio | 1.704 | 2 | .427 |
| Linear-by-Linear Association | 1.140 | 1 | .286 |
| N of Valid Cases | 476 |  |  |
| a. 0 cells (0.0%) have expected count less than 5. The minimum expected count is 41.73. | | | |

**Do you think these conditions can be treated with antibiotics? [Measles] * What is your gender?**

| **Crosstab** | | | | |
| --- | --- | --- | --- | --- |
| Count | | | | |
|  | | What is your gender? | | Total |
|  |  | Male | Female |  |
| Do you think these conditions can be treated with antibiotics? [Measles] | Yes | 22 | 32 | 54 |
|  | No | 94 | 162 | 256 |
|  | Don't Know | 75 | 91 | 166 |
| Total | | 191 | 285 | 476 |

| **Chi-Square Tests** | | | |
| --- | --- | --- | --- |
|  | Value | df | Asymptotic Significance (2-sided) |
| Pearson Chi-Square | 3.011^a^ | 2 | .222 |
| Likelihood Ratio | 3.003 | 2 | .223 |
| Linear-by-Linear Association | 1.393 | 1 | .238 |
| N of Valid Cases | 476 |  |  |
| a. 0 cells (0.0%) have expected count less than 5. The minimum expected count is 21.67. | | | |

**Do you think these conditions can be treated with antibiotics? [Skin or wound infection] * What is your gender?**

| **Crosstab** | | | | |
| --- | --- | --- | --- | --- |
| Count | | | | |
|  | | What is your gender? | | Total |
|  |  | Male | Female |  |
| Do you think these conditions can be treated with antibiotics? [Skin or wound infection] | Yes | 132 | 227 | 359 |
|  | No | 32 | 36 | 68 |
|  | Don't Know | 27 | 22 | 49 |
| Total | | 191 | 285 | 476 |

| **Chi-Square Tests** | | | |
| --- | --- | --- | --- |
|  | Value | df | Asymptotic Significance (2-sided) |
| Pearson Chi-Square | 7.619^a^ | 2 | .022 |
| Likelihood Ratio | 7.502 | 2 | .023 |
| Linear-by-Linear Association | 7.577 | 1 | .006 |
| N of Valid Cases | 476 |  |  |
| a. 0 cells (0.0%) have expected count less than 5. The minimum expected count is 19.66. | | | |

**Do you think these conditions can be treated with antibiotics? [Sore throat] * What is your gender?**

| **Crosstab** | | | | |
| --- | --- | --- | --- | --- |
| Count | | | | |
|  | | What is your gender? | | Total |
|  |  | Male | Female |  |
| Do you think these conditions can be treated with antibiotics? [Sore throat] | Yes | 74 | 116 | 190 |
|  | No | 93 | 151 | 244 |
|  | Don't Know | 24 | 18 | 42 |
| Total | | 191 | 285 | 476 |

| **Chi-Square Tests** | | | |
| --- | --- | --- | --- |
|  | Value | df | Asymptotic Significance (2-sided) |
| Pearson Chi-Square | 5.583^a^ | 2 | .061 |
| Likelihood Ratio | 5.456 | 2 | .065 |
| Linear-by-Linear Association | 1.968 | 1 | .161 |
| N of Valid Cases | 476 |  |  |
| a. 0 cells (0.0%) have expected count less than 5. The minimum expected count is 16.85. | | | |

**Do you think these conditions can be treated with antibiotics? [Body aches] * What is your gender?**

| **Crosstab** | | | | |
| --- | --- | --- | --- | --- |
| Count | | | | |
|  | | What is your gender? | | Total |
|  |  | Male | Female |  |
| Do you think these conditions can be treated with antibiotics? [Body aches] | Yes | 25 | 24 | 49 |
|  | No | 133 | 233 | 366 |
|  | Don't Know | 33 | 28 | 61 |
| Total | | 191 | 285 | 476 |

| **Chi-Square Tests** | | | |
| --- | --- | --- | --- |
|  | Value | df | Asymptotic Significance (2-sided) |
| Pearson Chi-Square | 9.563^a^ | 2 | .008 |
| Likelihood Ratio | 9.418 | 2 | .009 |
| Linear-by-Linear Association | .384 | 1 | .535 |
| N of Valid Cases | 476 |  |  |
| a. 0 cells (0.0%) have expected count less than 5. The minimum expected count is 19.66. | | | |

**Do you think these conditions can be treated with antibiotics? [Headaches] * What is your gender?**

| **Crosstab** | | | | |
| --- | --- | --- | --- | --- |
| Count | | | | |
|  | | What is your gender? | | Total |
|  |  | Male | Female |  |
| Do you think these conditions can be treated with antibiotics? [Headaches] | Yes | 14 | 8 | 22 |
|  | No | 156 | 269 | 425 |
|  | Don't Know | 21 | 8 | 29 |
| Total | | 191 | 285 | 476 |

| **Chi-Square Tests** | | | |
| --- | --- | --- | --- |
|  | Value | df | Asymptotic Significance (2-sided) |
| Pearson Chi-Square | 19.714^a^ | 2 | <.001 |
| Likelihood Ratio | 19.421 | 2 | <.001 |
| Linear-by-Linear Association | 1.434 | 1 | .231 |
| N of Valid Cases | 476 |  |  |
| a. 0 cells (0.0%) have expected count less than 5. The minimum expected count is 8.83. | | | |

**When do you think you should stop taking antibiotics once you've begun treatment? * What is your gender?**

| **Crosstab** | | | | |
| --- | --- | --- | --- | --- |
| Count | | | | |
|  | | What is your gender? | | Total |
|  |  | Male | Female |  |
| When do you think you should stop taking antibiotics once you've begun treatment? | When you have taken all of the antibiotics as directed | 175 | 278 | 453 |
|  | When you feel better | 15 | 7 | 22 |
|  | Don't know | 1 | 0 | 1 |
| Total | | 191 | 285 | 476 |

| **Chi-Square Tests** | | | |
| --- | --- | --- | --- |
|  | Value | df | Asymptotic Significance (2-sided) |
| Pearson Chi-Square | 9.121^a^ | 2 | .010 |
| Likelihood Ratio | 9.303 | 2 | .010 |
| Linear-by-Linear Association | 9.100 | 1 | .003 |
| N of Valid Cases | 476 |  |  |
| a. 2 cells (33.3%) have expected count less than 5. The minimum expected count is .40. | | | |

**Do you think this statement is ‘true’ or ‘false’? “It’s okay to use antibiotics that were given to a friend or family member, as long as they were used to treat the same illness” * What is your gender?**

| **Crosstab** | | | | |
| --- | --- | --- | --- | --- |
| Count | | | | |
|  | | What is your gender? | | Total |
|  |  | Male | Female |  |
| Do you think this statement is ‘true’ or ‘false’? “It’s okay to use antibiotics that were given to a friend or family member, as long as they were used to treat the same illness” | True | 15 | 13 | 28 |
|  | False | 163 | 269 | 432 |
|  | Don't Know | 13 | 3 | 16 |
| Total | | 191 | 285 | 476 |

| **Chi-Square Tests** | | | |
| --- | --- | --- | --- |
|  | Value | df | Asymptotic Significance (2-sided) |
| Pearson Chi-Square | 14.401^a^ | 2 | <.001 |
| Likelihood Ratio | 14.472 | 2 | <.001 |
| Linear-by-Linear Association | .753 | 1 | .385 |
| N of Valid Cases | 476 |  |  |
| a. 0 cells (0.0%) have expected count less than 5. The minimum expected count is 6.42. | | | |

**Do you think this statement is ‘true’ or ‘false’? “It’s okay to buy the same antibiotics, or request these from a doctor, if you’re sick and they helped you get better when you had the same symptoms before” * What is your gender?**

| **Crosstab** | | | | |
| --- | --- | --- | --- | --- |
| Count | | | | |
|  | | What is your gender? | | Total |
|  |  | Male | Female |  |
| Do you think this statement is ‘true’ or ‘false’? “It’s okay to buy the same antibiotics, or request these from a doctor, if you’re sick and they helped you get better when you had the same symptoms before” | True | 41 | 72 | 113 |
|  | False | 142 | 207 | 349 |
|  | Don't Know | 8 | 6 | 14 |
| Total | | 191 | 285 | 476 |

| **Chi-Square Tests** | | | |
| --- | --- | --- | --- |
|  | Value | df | Asymptotic Significance (2-sided) |
| Pearson Chi-Square | 2.428^a^ | 2 | .297 |
| Likelihood Ratio | 2.393 | 2 | .302 |
| Linear-by-Linear Association | 1.765 | 1 | .184 |
| N of Valid Cases | 476 |  |  |
| a. 0 cells (0.0%) have expected count less than 5. The minimum expected count is 5.62. | | | |

**Please indicate whether you think the following statements are ‘true’ or ‘false’ [Antibiotic resistance occurs when your body becomes resistant to antibiotics and they no longer work as well.] * What is your gender?**

| **Crosstab** | | | | |
| --- | --- | --- | --- | --- |
| Count | | | | |
|  | | What is your gender? | | Total |
|  |  | Male | Female |  |
| Please indicate whether you think the following statements are ‘true’ or ‘false’ [Antibiotic resistance occurs when your body becomes resistant to antibiotics and they no longer work as well.] | True | 131 | 224 | 355 |
|  | False | 38 | 38 | 76 |
|  | Don't Know | 22 | 23 | 45 |
| Total | | 191 | 285 | 476 |

| **Chi-Square Tests** | | | |
| --- | --- | --- | --- |
|  | Value | df | Asymptotic Significance (2-sided) |
| Pearson Chi-Square | 6.059^a^ | 2 | .048 |
| Likelihood Ratio | 5.987 | 2 | .050 |
| Linear-by-Linear Association | 4.966 | 1 | .026 |
| N of Valid Cases | 476 |  |  |
| a. 0 cells (0.0%) have expected count less than 5. The minimum expected count is 18.06. | | | |

**Please indicate whether you think the following statements are ‘true’ or ‘false’ [Many infections are becoming increasingly resistant to treatment by antibiotics ] * What is your gender?**

| **Crosstab** | | | | |
| --- | --- | --- | --- | --- |
| Count | | | | |
|  | | What is your gender? | | Total |
|  |  | Male | Female |  |
| Please indicate whether you think the following statements are ‘true’ or ‘false’ [Many infections are becoming increasingly resistant to treatment by antibiotics ] | True | 141 | 214 | 355 |
|  | False | 11 | 16 | 27 |
|  | Don't Know | 39 | 55 | 94 |
| Total | | 191 | 285 | 476 |

| **Chi-Square Tests** | | | |
| --- | --- | --- | --- |
|  | Value | df | Asymptotic Significance (2-sided) |
| Pearson Chi-Square | .102^a^ | 2 | .951 |
| Likelihood Ratio | .101 | 2 | .951 |
| Linear-by-Linear Association | .101 | 1 | .750 |
| N of Valid Cases | 476 |  |  |
| a. 0 cells (0.0%) have expected count less than 5. The minimum expected count is 10.83. | | | |

**Please indicate whether you think the following statements are ‘true’ or ‘false’[If bacteria are resistant to antibiotics, it can be very difficult or impossible to treat the infections they cause] * What is your gender?**

| **Crosstab** | | | | |
| --- | --- | --- | --- | --- |
| Count | | | | |
|  | | What is your gender? | | Total |
|  |  | Male | Female |  |
| Please indicate whether you think the following statements are ‘true’ or ‘false’[If bacteria are resistant to antibiotics, it can be very difficult or impossible to treat the infections they cause] | True | 153 | 228 | 381 |
|  | False | 11 | 23 | 34 |
|  | Don't Know | 27 | 34 | 61 |
| Total | | 191 | 285 | 476 |

| **Chi-Square Tests** | | | |
| --- | --- | --- | --- |
|  | Value | df | Asymptotic Significance (2-sided) |
| Pearson Chi-Square | 1.290^a^ | 2 | .525 |
| Likelihood Ratio | 1.308 | 2 | .520 |
| Linear-by-Linear Association | .106 | 1 | .745 |
| N of Valid Cases | 476 |  |  |
| a. 0 cells (0.0%) have expected count less than 5. The minimum expected count is 13.64. | | | |

**Please indicate whether you think the following statements are ‘true’ or ‘false’ [Antibiotic resistance is an issue that could affect me or my family] * What is your gender?**

| **Crosstab** | | | | |
| --- | --- | --- | --- | --- |
| Count | | | | |
|  | | What is your gender? | | Total |
|  |  | Male | Female |  |
| Please indicate whether you think the following statements are ‘true’ or ‘false’ [Antibiotic resistance is an issue that could affect me or my family] | True | 151 | 236 | 387 |
|  | False | 12 | 16 | 28 |
|  | Don't Know | 28 | 33 | 61 |
| Total | | 191 | 285 | 476 |

| **Chi-Square Tests** | | | |
| --- | --- | --- | --- |
|  | Value | df | Asymptotic Significance (2-sided) |
| Pearson Chi-Square | 1.132^a^ | 2 | .568 |
| Likelihood Ratio | 1.120 | 2 | .571 |
| Linear-by-Linear Association | 1.128 | 1 | .288 |
| N of Valid Cases | 476 |  |  |
| a. 0 cells (0.0%) have expected count less than 5. The minimum expected count is 11.24. | | | |

**Please indicate whether you think the following statements are ‘true’ or ‘false’ [Antibiotic resistance is an issue in other countries but not here] * What is your gender?**

| **Crosstab** | | | | |
| --- | --- | --- | --- | --- |
| Count | | | | |
|  | | What is your gender? | | Total |
|  |  | Male | Female |  |
| Please indicate whether you think the following statements are ‘true’ or ‘false’ [Antibiotic resistance is an issue in other countries but not here] | True | 8 | 9 | 17 |
|  | False | 153 | 230 | 383 |
|  | Don't Know | 30 | 46 | 76 |
| Total | | 191 | 285 | 476 |

| **Chi-Square Tests** | | | |
| --- | --- | --- | --- |
|  | Value | df | Asymptotic Significance (2-sided) |
| Pearson Chi-Square | .359^a^ | 2 | .836 |
| Likelihood Ratio | .353 | 2 | .838 |
| Linear-by-Linear Association | .136 | 1 | .712 |
| N of Valid Cases | 476 |  |  |
| a. 0 cells (0.0%) have expected count less than 5. The minimum expected count is 6.82. | | | |

**Please indicate whether you think the following statements are ‘true’ or ‘false’ [Antibiotic resistance is only a problem for people who take antibiotics regularly] * What is your gender?**

| **Crosstab** | | | | |
| --- | --- | --- | --- | --- |
| Count | | | | |
|  | | What is your gender? | | Total |
|  |  | Male | Female |  |
| Please indicate whether you think the following statements are ‘true’ or ‘false’ [Antibiotic resistance is only a problem for people who take antibiotics regularly] | True | 36 | 60 | 96 |
|  | False | 121 | 174 | 295 |
|  | Don't Know | 34 | 51 | 85 |
| Total | | 191 | 285 | 476 |

| **Chi-Square Tests** | | | |
| --- | --- | --- | --- |
|  | Value | df | Asymptotic Significance (2-sided) |
| Pearson Chi-Square | .374^a^ | 2 | .830 |
| Likelihood Ratio | .375 | 2 | .829 |
| Linear-by-Linear Association | .134 | 1 | .714 |
| N of Valid Cases | 476 |  |  |
| a. 0 cells (0.0%) have expected count less than 5. The minimum expected count is 34.11. | | | |

**Please indicate whether you think the following statements are ‘true’ or ‘false’ [Bacteria which are resistant to antibiotics can be spread from person to person] * What is your gender?**

| **Crosstab** | | | | |
| --- | --- | --- | --- | --- |
| Count | | | | |
|  | | What is your gender? | | Total |
|  |  | Male | Female |  |
| Please indicate whether you think the following statements are ‘true’ or ‘false’ [Bacteria which are resistant to antibiotics can be spread from person to person] | True | 120 | 170 | 290 |
|  | False | 15 | 43 | 58 |
|  | Don't Know | 56 | 72 | 128 |
| Total | | 191 | 285 | 476 |

| **Chi-Square Tests** | | | |
| --- | --- | --- | --- |
|  | Value | df | Asymptotic Significance (2-sided) |
| Pearson Chi-Square | 5.801^a^ | 2 | .055 |
| Likelihood Ratio | 6.082 | 2 | .048 |
| Linear-by-Linear Association | .012 | 1 | .914 |
| N of Valid Cases | 476 |  |  |
| a. 0 cells (0.0%) have expected count less than 5. The minimum expected count is 23.27. | | | |

**Please indicate whether you think the following statements are ‘true’ or ‘false’ [Antibiotic-resistant infections could make medical procedures like surgery, organ transplants and cancer treatment much more dangerous] * What is your gender?**

| **Crosstab** | | | | |
| --- | --- | --- | --- | --- |
| Count | | | | |
|  | | What is your gender? | | Total |
|  |  | Male | Female |  |
| Please indicate whether you think the following statements are ‘true’ or ‘false’ [Antibiotic-resistant infections could make medical procedures like surgery, organ transplants and cancer treatment much more dangerous] | True | 145 | 233 | 378 |
|  | False | 10 | 6 | 16 |
|  | Don't Know | 36 | 46 | 82 |
| Total | | 191 | 285 | 476 |

| **Chi-Square Tests** | | | |
| --- | --- | --- | --- |
|  | Value | df | Asymptotic Significance (2-sided) |
| Pearson Chi-Square | 4.311^a^ | 2 | .116 |
| Likelihood Ratio | 4.224 | 2 | .121 |
| Linear-by-Linear Association | 1.438 | 1 | .231 |
| N of Valid Cases | 476 |  |  |
| a. 0 cells (0.0%) have expected count less than 5. The minimum expected count is 6.42. | | | |

(15) HOUSEHOLD INCOME DIFFERENCES ON CATEGORICAL RESPONSES

**Do you think these conditions can be treated with antibiotics? [HIV/AIDS] * HouseholdIncome**

| **Crosstab** | | | | | | | |
| --- | --- | --- | --- | --- | --- | --- | --- |
| Count | | | | | | | |
|  | | HouseholdIncome | | | | | Total |
|  |  | Up to Eur24999 | Eur25000-49999 | Eur50000-99999 | Eur100000+ | Prefer Not to Say |  |
| Do you think these conditions can be treated with antibiotics? [HIV/AIDS] | Yes | 10 | 15 | 8 | 2 | 7 | 42 |
|  | No | 33 | 97 | 82 | 43 | 38 | 293 |
|  | Don't Know | 15 | 52 | 35 | 22 | 17 | 141 |
| Total | | 58 | 164 | 125 | 67 | 62 | 476 |

| **Chi-Square Tests** | | | |
| --- | --- | --- | --- |
|  | Value | df | Asymptotic Significance (2-sided) |
| Pearson Chi-Square | 10.256^a^ | 8 | .248 |
| Likelihood Ratio | 10.188 | 8 | .252 |
| Linear-by-Linear Association | .558 | 1 | .455 |
| N of Valid Cases | 476 |  |  |
| a. 0 cells (0.0%) have expected count less than 5. The minimum expected count is 5.12. | | | |

**Do you think these conditions can be treated with antibiotics?[Gonorrhoea] * HouseholdIncome**

| **Crosstab** | | | | | | | |
| --- | --- | --- | --- | --- | --- | --- | --- |
| Count | | | | | | | |
|  | | HouseholdIncome | | | | | Total |
|  |  | Up to Eur24999 | Eur25000-49999 | Eur50000-99999 | Eur100000+ | Prefer Not to Say |  |
| Do you think these conditions can be treated with antibiotics?[Gonorrhoea] | Yes | 19 | 57 | 53 | 20 | 25 | 174 |
|  | No | 9 | 34 | 16 | 12 | 9 | 80 |
|  | Don't Know | 30 | 73 | 56 | 35 | 28 | 222 |
| Total | | 58 | 164 | 125 | 67 | 62 | 476 |

| **Chi-Square Tests** | | | |
| --- | --- | --- | --- |
|  | Value | df | Asymptotic Significance (2-sided) |
| Pearson Chi-Square | 6.654^a^ | 8 | .574 |
| Likelihood Ratio | 6.644 | 8 | .575 |
| Linear-by-Linear Association | .113 | 1 | .737 |
| N of Valid Cases | 476 |  |  |
| a. 0 cells (0.0%) have expected count less than 5. The minimum expected count is 9.75. | | | |

**Do you think these conditions can be treated with antibiotics? [Bladder infection or urinary tract infection (UTI) ] * HouseholdIncome**

| **Crosstab** | | | | | | | |
| --- | --- | --- | --- | --- | --- | --- | --- |
| Count | | | | | | | |
|  | | HouseholdIncome | | | | | Total |
|  |  | Up to Eur24999 | Eur25000-49999 | Eur50000-99999 | Eur100000+ | Prefer Not to Say |  |
| Do you think these conditions can be treated with antibiotics? [Bladder infection or urinary tract infection (UTI) ] | Yes | 43 | 137 | 110 | 55 | 53 | 398 |
|  | No | 5 | 9 | 2 | 1 | 2 | 19 |
|  | Don't Know | 10 | 18 | 13 | 11 | 7 | 59 |
| Total | | 58 | 164 | 125 | 67 | 62 | 476 |

| **Chi-Square Tests** | | | |
| --- | --- | --- | --- |
|  | Value | df | Asymptotic Significance (2-sided) |
| Pearson Chi-Square | 10.622^a^ | 8 | .224 |
| Likelihood Ratio | 10.465 | 8 | .234 |
| Linear-by-Linear Association | .657 | 1 | .418 |
| N of Valid Cases | 476 |  |  |
| a. 4 cells (26.7%) have expected count less than 5. The minimum expected count is 2.32. | | | |

**Do you think these conditions can be treated with antibiotics? [Diarrhoea] * HouseholdIncome**

| **Crosstab** | | | | | | | |
| --- | --- | --- | --- | --- | --- | --- | --- |
| Count | | | | | | | |
|  | | HouseholdIncome | | | | | Total |
|  |  | Up to Eur24999 | Eur25000-49999 | Eur50000-99999 | Eur100000+ | Prefer Not to Say |  |
| Do you think these conditions can be treated with antibiotics? [Diarrhoea] | Yes | 12 | 20 | 19 | 11 | 11 | 73 |
|  | No | 40 | 115 | 91 | 49 | 40 | 335 |
|  | Don't Know | 6 | 29 | 15 | 7 | 11 | 68 |
| Total | | 58 | 164 | 125 | 67 | 62 | 476 |

| **Chi-Square Tests** | | | |
| --- | --- | --- | --- |
|  | Value | df | Asymptotic Significance (2-sided) |
| Pearson Chi-Square | 6.545^a^ | 8 | .586 |
| Likelihood Ratio | 6.546 | 8 | .586 |
| Linear-by-Linear Association | .018 | 1 | .893 |
| N of Valid Cases | 476 |  |  |
| a. 0 cells (0.0%) have expected count less than 5. The minimum expected count is 8.29. | | | |

**Do you think these conditions can be treated with antibiotics? [Cold and flu] * HouseholdIncome**

| **Crosstab** | | | | | | | |
| --- | --- | --- | --- | --- | --- | --- | --- |
| Count | | | | | | | |
|  | | HouseholdIncome | | | | | Total |
|  |  | Up to Eur24999 | Eur25000-49999 | Eur50000-99999 | Eur100000+ | Prefer Not to Say |  |
| Do you think these conditions can be treated with antibiotics? [Cold and flu] | Yes | 16 | 24 | 14 | 13 | 12 | 79 |
|  | No | 34 | 130 | 107 | 49 | 45 | 365 |
|  | Don't Know | 8 | 10 | 4 | 5 | 5 | 32 |
| Total | | 58 | 164 | 125 | 67 | 62 | 476 |

| **Chi-Square Tests** | | | |
| --- | --- | --- | --- |
|  | Value | df | Asymptotic Significance (2-sided) |
| Pearson Chi-Square | 18.486^a^ | 8 | .018 |
| Likelihood Ratio | 17.760 | 8 | .023 |
| Linear-by-Linear Association | .004 | 1 | .950 |
| N of Valid Cases | 476 |  |  |
| a. 3 cells (20.0%) have expected count less than 5. The minimum expected count is 3.90. | | | |

**Do you think these conditions can be treated with antibiotics? [Fever] * HouseholdIncome**

| **Crosstab** | | | | | | | |
| --- | --- | --- | --- | --- | --- | --- | --- |
| Count | | | | | | | |
|  | | HouseholdIncome | | | | | Total |
|  |  | Up to Eur24999 | Eur25000-49999 | Eur50000-99999 | Eur100000+ | Prefer Not to Say |  |
| Do you think these conditions can be treated with antibiotics? [Fever] | Yes | 24 | 50 | 47 | 22 | 17 | 160 |
|  | No | 30 | 103 | 73 | 36 | 36 | 278 |
|  | Don't Know | 4 | 11 | 5 | 9 | 9 | 38 |
| Total | | 58 | 164 | 125 | 67 | 62 | 476 |

| **Chi-Square Tests** | | | |
| --- | --- | --- | --- |
|  | Value | df | Asymptotic Significance (2-sided) |
| Pearson Chi-Square | 12.778^a^ | 8 | .120 |
| Likelihood Ratio | 12.265 | 8 | .140 |
| Linear-by-Linear Association | 3.033 | 1 | .082 |
| N of Valid Cases | 476 |  |  |
| a. 2 cells (13.3%) have expected count less than 5. The minimum expected count is 4.63. | | | |

**Do you think these conditions can be treated with antibiotics? [Malaria] * HouseholdIncome**

| **Crosstab** | | | | | | | |
| --- | --- | --- | --- | --- | --- | --- | --- |
| Count | | | | | | | |
|  | | HouseholdIncome | | | | | Total |
|  |  | Up to Eur24999 | Eur25000-49999 | Eur50000-99999 | Eur100000+ | Prefer Not to Say |  |
| Do you think these conditions can be treated with antibiotics? [Malaria] | Yes | 23 | 27 | 26 | 13 | 15 | 104 |
|  | No | 11 | 65 | 49 | 22 | 21 | 168 |
|  | Don't Know | 24 | 72 | 50 | 32 | 26 | 204 |
| Total | | 58 | 164 | 125 | 67 | 62 | 476 |

| **Chi-Square Tests** | | | |
| --- | --- | --- | --- |
|  | Value | df | Asymptotic Significance (2-sided) |
| Pearson Chi-Square | 17.641^a^ | 8 | .024 |
| Likelihood Ratio | 17.034 | 8 | .030 |
| Linear-by-Linear Association | .384 | 1 | .535 |
| N of Valid Cases | 476 |  |  |
| a. 0 cells (0.0%) have expected count less than 5. The minimum expected count is 12.67. | | | |

**Do you think these conditions can be treated with antibiotics? [Measles] * HouseholdIncome**

| **Crosstab** | | | | | | | |
| --- | --- | --- | --- | --- | --- | --- | --- |
| Count | | | | | | | |
|  | | HouseholdIncome | | | | | Total |
|  |  | Up to Eur24999 | Eur25000-49999 | Eur50000-99999 | Eur100000+ | Prefer Not to Say |  |
| Do you think these conditions can be treated with antibiotics? [Measles] | Yes | 10 | 18 | 12 | 9 | 5 | 54 |
|  | No | 24 | 88 | 81 | 31 | 32 | 256 |
|  | Don't Know | 24 | 58 | 32 | 27 | 25 | 166 |
| Total | | 58 | 164 | 125 | 67 | 62 | 476 |

| **Chi-Square Tests** | | | |
| --- | --- | --- | --- |
|  | Value | df | Asymptotic Significance (2-sided) |
| Pearson Chi-Square | 13.108^a^ | 8 | .108 |
| Likelihood Ratio | 13.161 | 8 | .106 |
| Linear-by-Linear Association | .423 | 1 | .515 |
| N of Valid Cases | 476 |  |  |
| a. 0 cells (0.0%) have expected count less than 5. The minimum expected count is 6.58. | | | |

**Do you think these conditions can be treated with antibiotics? [Skin or wound infection] * HouseholdIncome**

| **Crosstab** | | | | | | | |
| --- | --- | --- | --- | --- | --- | --- | --- |
| Count | | | | | | | |
|  | | HouseholdIncome | | | | | Total |
|  |  | Up to Eur24999 | Eur25000-49999 | Eur50000-99999 | Eur100000+ | Prefer Not to Say |  |
| Do you think these conditions can be treated with antibiotics? [Skin or wound infection] | Yes | 42 | 122 | 100 | 49 | 46 | 359 |
|  | No | 9 | 26 | 16 | 8 | 9 | 68 |
|  | Don't Know | 7 | 16 | 9 | 10 | 7 | 49 |
| Total | | 58 | 164 | 125 | 67 | 62 | 476 |

| **Chi-Square Tests** | | | |
| --- | --- | --- | --- |
|  | Value | df | Asymptotic Significance (2-sided) |
| Pearson Chi-Square | 4.138^a^ | 8 | .844 |
| Likelihood Ratio | 4.099 | 8 | .848 |
| Linear-by-Linear Association | .004 | 1 | .952 |
| N of Valid Cases | 476 |  |  |
| a. 0 cells (0.0%) have expected count less than 5. The minimum expected count is 5.97. | | | |

**Do you think these conditions can be treated with antibiotics? [Sore throat] * HouseholdIncome**

| **Crosstab** | | | | | | | |
| --- | --- | --- | --- | --- | --- | --- | --- |
| Count | | | | | | | |
|  | | HouseholdIncome | | | | | Total |
|  |  | Up to Eur24999 | Eur25000-49999 | Eur50000-99999 | Eur100000+ | Prefer Not to Say |  |
| Do you think these conditions can be treated with antibiotics? [Sore throat] | Yes | 33 | 60 | 50 | 21 | 26 | 190 |
|  | No | 22 | 91 | 64 | 40 | 27 | 244 |
|  | Don't Know | 3 | 13 | 11 | 6 | 9 | 42 |
| Total | | 58 | 164 | 125 | 67 | 62 | 476 |

| **Chi-Square Tests** | | | |
| --- | --- | --- | --- |
|  | Value | df | Asymptotic Significance (2-sided) |
| Pearson Chi-Square | 13.480^a^ | 8 | .096 |
| Likelihood Ratio | 13.061 | 8 | .110 |
| Linear-by-Linear Association | 3.383 | 1 | .066 |
| N of Valid Cases | 476 |  |  |
| a. 0 cells (0.0%) have expected count less than 5. The minimum expected count is 5.12. | | | |

**Do you think these conditions can be treated with antibiotics? [Body aches] * HouseholdIncome**

| **Crosstab** | | | | | | | |
| --- | --- | --- | --- | --- | --- | --- | --- |
| Count | | | | | | | |
|  | | HouseholdIncome | | | | | Total |
|  |  | Up to Eur24999 | Eur25000-49999 | Eur50000-99999 | Eur100000+ | Prefer Not to Say |  |
| Do you think these conditions can be treated with antibiotics? [Body aches] | Yes | 11 | 16 | 11 | 7 | 4 | 49 |
|  | No | 39 | 131 | 101 | 47 | 48 | 366 |
|  | Don't Know | 8 | 17 | 13 | 13 | 10 | 61 |
| Total | | 58 | 164 | 125 | 67 | 62 | 476 |

| **Chi-Square Tests** | | | |
| --- | --- | --- | --- |
|  | Value | df | Asymptotic Significance (2-sided) |
| Pearson Chi-Square | 11.164^a^ | 8 | .193 |
| Likelihood Ratio | 10.241 | 8 | .248 |
| Linear-by-Linear Association | 3.974 | 1 | .046 |
| N of Valid Cases | 476 |  |  |
| a. 0 cells (0.0%) have expected count less than 5. The minimum expected count is 5.97. | | | |

**Do you think these conditions can be treated with antibiotics? [Headaches] * HouseholdIncome**

| **Crosstab** | | | | | | | |
| --- | --- | --- | --- | --- | --- | --- | --- |
| Count | | | | | | | |
|  | | HouseholdIncome | | | | | Total |
|  |  | Up to Eur24999 | Eur25000-49999 | Eur50000-99999 | Eur100000+ | Prefer Not to Say |  |
| Do you think these conditions can be treated with antibiotics? [Headaches] | Yes | 7 | 8 | 3 | 1 | 3 | 22 |
|  | No | 46 | 146 | 115 | 63 | 55 | 425 |
|  | Don't Know | 5 | 10 | 7 | 3 | 4 | 29 |
| Total | | 58 | 164 | 125 | 67 | 62 | 476 |

| **Chi-Square Tests** | | | |
| --- | --- | --- | --- |
|  | Value | df | Asymptotic Significance (2-sided) |
| Pearson Chi-Square | 11.624^a^ | 8 | .169 |
| Likelihood Ratio | 10.246 | 8 | .248 |
| Linear-by-Linear Association | .718 | 1 | .397 |
| N of Valid Cases | 476 |  |  |
| a. 6 cells (40.0%) have expected count less than 5. The minimum expected count is 2.68. | | | |

**When do you think you should stop taking antibiotics once you've begun treatment? * HouseholdIncome**

| **Crosstab** | | | | | | | |
| --- | --- | --- | --- | --- | --- | --- | --- |
| Count | | | | | | | |
|  | | HouseholdIncome | | | | | Total |
|  |  | Up to Eur24999 | Eur25000-49999 | Eur50000-99999 | Eur100000+ | Prefer Not to Say |  |
| When do you think you should stop taking antibiotics once you've begun treatment? | When you have taken all of the antibiotics as directed | 53 | 154 | 122 | 66 | 58 | 453 |
|  | When you feel better | 5 | 10 | 3 | 0 | 4 | 22 |
|  | Don't know | 0 | 0 | 0 | 1 | 0 | 1 |
| Total | | 58 | 164 | 125 | 67 | 62 | 476 |

| **Chi-Square Tests** | | | |
| --- | --- | --- | --- |
|  | Value | df | Asymptotic Significance (2-sided) |
| Pearson Chi-Square | 14.053^a^ | 8 | .080 |
| Likelihood Ratio | 14.722 | 8 | .065 |
| Linear-by-Linear Association | .839 | 1 | .360 |
| N of Valid Cases | 476 |  |  |
| a. 8 cells (53.3%) have expected count less than 5. The minimum expected count is .12. | | | |

**Do you think this statement is ‘true’ or ‘false’? “It’s okay to use antibiotics that were given to a friend or family member, as long as they were used to treat the same illness” * HouseholdIncome**

| **Crosstab** | | | | | | | |
| --- | --- | --- | --- | --- | --- | --- | --- |
| Count | | | | | | | |
|  | | HouseholdIncome | | | | | Total |
|  |  | Up to Eur24999 | Eur25000-49999 | Eur50000-99999 | Eur100000+ | Prefer Not to Say |  |
| Do you think this statement is ‘true’ or ‘false’? “It’s okay to use antibiotics that were given to a friend or family member, as long as they were used to treat the same illness” | True | 4 | 10 | 6 | 5 | 3 | 28 |
|  | False | 50 | 148 | 116 | 62 | 56 | 432 |
|  | Don't Know | 4 | 6 | 3 | 0 | 3 | 16 |
| Total | | 58 | 164 | 125 | 67 | 62 | 476 |

| **Chi-Square Tests** | | | |
| --- | --- | --- | --- |
|  | Value | df | Asymptotic Significance (2-sided) |
| Pearson Chi-Square | 6.180^a^ | 8 | .627 |
| Likelihood Ratio | 7.910 | 8 | .442 |
| Linear-by-Linear Association | .165 | 1 | .685 |
| N of Valid Cases | 476 |  |  |
| a. 7 cells (46.7%) have expected count less than 5. The minimum expected count is 1.95. | | | |

**Do you think this statement is ‘true’ or ‘false’? “It’s okay to buy the same antibiotics, or request these from a doctor, if you’re sick and they helped you get better when you had the same symptoms before” * HouseholdIncome**

| **Crosstab** | | | | | | | |
| --- | --- | --- | --- | --- | --- | --- | --- |
| Count | | | | | | | |
|  | | HouseholdIncome | | | | | Total |
|  |  | Up to Eur24999 | Eur25000-49999 | Eur50000-99999 | Eur100000+ | Prefer Not to Say |  |
| Do you think this statement is ‘true’ or ‘false’? “It’s okay to buy the same antibiotics, or request these from a doctor, if you’re sick and they helped you get better when you had the same symptoms before” | True | 19 | 35 | 25 | 13 | 21 | 113 |
|  | False | 37 | 124 | 96 | 54 | 38 | 349 |
|  | Don't Know | 2 | 5 | 4 | 0 | 3 | 14 |
| Total | | 58 | 164 | 125 | 67 | 62 | 476 |

| **Chi-Square Tests** | | | |
| --- | --- | --- | --- |
|  | Value | df | Asymptotic Significance (2-sided) |
| Pearson Chi-Square | 11.897^a^ | 8 | .156 |
| Likelihood Ratio | 13.389 | 8 | .099 |
| Linear-by-Linear Association | .079 | 1 | .778 |
| N of Valid Cases | 476 |  |  |
| a. 5 cells (33.3%) have expected count less than 5. The minimum expected count is 1.71. | | | |

**Please indicate whether you think the following statements are ‘true’ or ‘false’ [Antibiotic resistance occurs when your body becomes resistant to antibiotics and they no longer work as well.] * HouseholdIncome**

| **Crosstab** | | | | | | | |
| --- | --- | --- | --- | --- | --- | --- | --- |
| Count | | | | | | | |
|  | | HouseholdIncome | | | | | Total |
|  |  | Up to Eur24999 | Eur25000-49999 | Eur50000-99999 | Eur100000+ | Prefer Not to Say |  |
| Please indicate whether you think the following statements are ‘true’ or ‘false’ [Antibiotic resistance occurs when your body becomes resistant to antibiotics and they no longer work as well.] | True | 46 | 115 | 94 | 56 | 44 | 355 |
|  | False | 4 | 37 | 20 | 4 | 11 | 76 |
|  | Don't Know | 8 | 12 | 11 | 7 | 7 | 45 |
| Total | | 58 | 164 | 125 | 67 | 62 | 476 |

| **Chi-Square Tests** | | | |
| --- | --- | --- | --- |
|  | Value | df | Asymptotic Significance (2-sided) |
| Pearson Chi-Square | 15.520^a^ | 8 | .050 |
| Likelihood Ratio | 17.040 | 8 | .030 |
| Linear-by-Linear Association | .013 | 1 | .908 |
| N of Valid Cases | 476 |  |  |
| a. 0 cells (0.0%) have expected count less than 5. The minimum expected count is 5.48. | | | |

**Please indicate whether you think the following statements are ‘true’ or ‘false’ [Many infections are becoming increasingly resistant to treatment by antibiotics ] * HouseholdIncome**

| **Crosstab** | | | | | | | |
| --- | --- | --- | --- | --- | --- | --- | --- |
| Count | | | | | | | |
|  | | HouseholdIncome | | | | | Total |
|  |  | Up to Eur24999 | Eur25000-49999 | Eur50000-99999 | Eur100000+ | Prefer Not to Say |  |
| Please indicate whether you think the following statements are ‘true’ or ‘false’ [Many infections are becoming increasingly resistant to treatment by antibiotics ] | True | 41 | 124 | 96 | 48 | 46 | 355 |
|  | False | 2 | 13 | 6 | 4 | 2 | 27 |
|  | Don't Know | 15 | 27 | 23 | 15 | 14 | 94 |
| Total | | 58 | 164 | 125 | 67 | 62 | 476 |

| **Chi-Square Tests** | | | |
| --- | --- | --- | --- |
|  | Value | df | Asymptotic Significance (2-sided) |
| Pearson Chi-Square | 5.707^a^ | 8 | .680 |
| Likelihood Ratio | 5.698 | 8 | .681 |
| Linear-by-Linear Association | .023 | 1 | .880 |
| N of Valid Cases | 476 |  |  |
| a. 3 cells (20.0%) have expected count less than 5. The minimum expected count is 3.29. | | | |

**Please indicate whether you think the following statements are ‘true’ or ‘false’[If bacteria are resistant to antibiotics, it can be very difficult or impossible to treat the infections they cause] * HouseholdIncome**

| **Crosstab** | | | | | | | |
| --- | --- | --- | --- | --- | --- | --- | --- |
| Count | | | | | | | |
|  | | HouseholdIncome | | | | | Total |
|  |  | Up to Eur24999 | Eur25000-49999 | Eur50000-99999 | Eur100000+ | Prefer Not to Say |  |
| Please indicate whether you think the following statements are ‘true’ or ‘false’[If bacteria are resistant to antibiotics, it can be very difficult or impossible to treat the infections they cause] | True | 45 | 133 | 101 | 53 | 49 | 381 |
|  | False | 3 | 16 | 7 | 2 | 6 | 34 |
|  | Don't Know | 10 | 15 | 17 | 12 | 7 | 61 |
| Total | | 58 | 164 | 125 | 67 | 62 | 476 |

| **Chi-Square Tests** | | | |
| --- | --- | --- | --- |
|  | Value | df | Asymptotic Significance (2-sided) |
| Pearson Chi-Square | 8.709^a^ | 8 | .367 |
| Likelihood Ratio | 8.976 | 8 | .344 |
| Linear-by-Linear Association | .030 | 1 | .862 |
| N of Valid Cases | 476 |  |  |
| a. 3 cells (20.0%) have expected count less than 5. The minimum expected count is 4.14. | | | |

**Please indicate whether you think the following statements are ‘true’ or ‘false’ [Antibiotic resistance is an issue that could affect me or my family] * HouseholdIncome**

| **Crosstab** | | | | | | | |
| --- | --- | --- | --- | --- | --- | --- | --- |
| Count | | | | | | | |
|  | | HouseholdIncome | | | | | Total |
|  |  | Up to Eur24999 | Eur25000-49999 | Eur50000-99999 | Eur100000+ | Prefer Not to Say |  |
| Please indicate whether you think the following statements are ‘true’ or ‘false’ [Antibiotic resistance is an issue that could affect me or my family] | True | 46 | 135 | 100 | 56 | 50 | 387 |
|  | False | 1 | 10 | 11 | 2 | 4 | 28 |
|  | Don't Know | 11 | 19 | 14 | 9 | 8 | 61 |
| Total | | 58 | 164 | 125 | 67 | 62 | 476 |

| **Chi-Square Tests** | | | |
| --- | --- | --- | --- |
|  | Value | df | Asymptotic Significance (2-sided) |
| Pearson Chi-Square | 6.819^a^ | 8 | .556 |
| Likelihood Ratio | 7.248 | 8 | .510 |
| Linear-by-Linear Association | .108 | 1 | .743 |
| N of Valid Cases | 476 |  |  |
| a. 3 cells (20.0%) have expected count less than 5. The minimum expected count is 3.41. | | | |

**Please indicate whether you think the following statements are ‘true’ or ‘false’ [Antibiotic resistance is an issue in other countries but not here] * HouseholdIncome**

| **Crosstab** | | | | | | | |
| --- | --- | --- | --- | --- | --- | --- | --- |
| Count | | | | | | | |
|  | | HouseholdIncome | | | | | Total |
|  |  | Up to Eur24999 | Eur25000-49999 | Eur50000-99999 | Eur100000+ | Prefer Not to Say |  |
| Please indicate whether you think the following statements are ‘true’ or ‘false’ [Antibiotic resistance is an issue in other countries but not here] | True | 2 | 8 | 3 | 0 | 4 | 17 |
|  | False | 38 | 129 | 108 | 57 | 51 | 383 |
|  | Don't Know | 18 | 27 | 14 | 10 | 7 | 76 |
| Total | | 58 | 164 | 125 | 67 | 62 | 476 |

| **Chi-Square Tests** | | | |
| --- | --- | --- | --- |
|  | Value | df | Asymptotic Significance (2-sided) |
| Pearson Chi-Square | 18.472^a^ | 8 | .018 |
| Likelihood Ratio | 19.168 | 8 | .014 |
| Linear-by-Linear Association | 4.586 | 1 | .032 |
| N of Valid Cases | 476 |  |  |
| a. 4 cells (26.7%) have expected count less than 5. The minimum expected count is 2.07. | | | |

**Please indicate whether you think the following statements are ‘true’ or ‘false’ [Antibiotic resistance is only a problem for people who take antibiotics regularly] * HouseholdIncome**

| **Crosstab** | | | | | | | |
| --- | --- | --- | --- | --- | --- | --- | --- |
| Count | | | | | | | |
|  | | HouseholdIncome | | | | | Total |
|  |  | Up to Eur24999 | Eur25000-49999 | Eur50000-99999 | Eur100000+ | Prefer Not to Say |  |
| Please indicate whether you think the following statements are ‘true’ or ‘false’ [Antibiotic resistance is only a problem for people who take antibiotics regularly] | True | 18 | 33 | 19 | 11 | 15 | 96 |
|  | False | 27 | 103 | 89 | 42 | 34 | 295 |
|  | Don't Know | 13 | 28 | 17 | 14 | 13 | 85 |
| Total | | 58 | 164 | 125 | 67 | 62 | 476 |

| **Chi-Square Tests** | | | |
| --- | --- | --- | --- |
|  | Value | df | Asymptotic Significance (2-sided) |
| Pearson Chi-Square | 13.049^a^ | 8 | .110 |
| Likelihood Ratio | 12.891 | 8 | .116 |
| Linear-by-Linear Association | .540 | 1 | .462 |
| N of Valid Cases | 476 |  |  |
| a. 0 cells (0.0%) have expected count less than 5. The minimum expected count is 10.36. | | | |

**Please indicate whether you think the following statements are ‘true’ or ‘false’ [Bacteria which are resistant to antibiotics can be spread from person to person] * HouseholdIncome**

| **Crosstab** | | | | | | | |
| --- | --- | --- | --- | --- | --- | --- | --- |
| Count | | | | | | | |
|  | | HouseholdIncome | | | | | Total |
|  |  | Up to Eur24999 | Eur25000-49999 | Eur50000-99999 | Eur100000+ | Prefer Not to Say |  |
| Please indicate whether you think the following statements are ‘true’ or ‘false’ [Bacteria which are resistant to antibiotics can be spread from person to person] | True | 33 | 99 | 84 | 38 | 36 | 290 |
|  | False | 11 | 19 | 10 | 9 | 9 | 58 |
|  | Don't Know | 14 | 46 | 31 | 20 | 17 | 128 |
| Total | | 58 | 164 | 125 | 67 | 62 | 476 |

| **Chi-Square Tests** | | | |
| --- | --- | --- | --- |
|  | Value | df | Asymptotic Significance (2-sided) |
| Pearson Chi-Square | 6.318^a^ | 8 | .612 |
| Likelihood Ratio | 6.215 | 8 | .623 |
| Linear-by-Linear Association | .042 | 1 | .838 |
| N of Valid Cases | 476 |  |  |
| a. 0 cells (0.0%) have expected count less than 5. The minimum expected count is 7.07. | | | |

**Please indicate whether you think the following statements are ‘true’ or ‘false’ [Antibiotic-resistant infections could make medical procedures like surgery, organ transplants and cancer treatment much more dangerous] * HouseholdIncome**

| **Crosstab** | | | | | | | |
| --- | --- | --- | --- | --- | --- | --- | --- |
| Count | | | | | | | |
|  | | HouseholdIncome | | | | | Total |
|  |  | Up to Eur24999 | Eur25000-49999 | Eur50000-99999 | Eur100000+ | Prefer Not to Say |  |
| Please indicate whether you think the following statements are ‘true’ or ‘false’ [Antibiotic-resistant infections could make medical procedures like surgery, organ transplants and cancer treatment much more dangerous] | True | 40 | 137 | 100 | 53 | 48 | 378 |
|  | False | 3 | 6 | 2 | 1 | 4 | 16 |
|  | Don't Know | 15 | 21 | 23 | 13 | 10 | 82 |
| Total | | 58 | 164 | 125 | 67 | 62 | 476 |

| **Chi-Square Tests** | | | |
| --- | --- | --- | --- |
|  | Value | df | Asymptotic Significance (2-sided) |
| Pearson Chi-Square | 10.105^a^ | 8 | .258 |
| Likelihood Ratio | 9.979 | 8 | .266 |
| Linear-by-Linear Association | .071 | 1 | .790 |
| N of Valid Cases | 476 |  |  |
| a. 4 cells (26.7%) have expected count less than 5. The minimum expected count is 1.95. | | | |

(16) REGION DIFFERENCES ON CATEGORICAL RESPONSES

**Do you think these conditions can be treated with antibiotics? [HIV/AIDS] * Which region of Malta do you live in?**

| **Crosstab** | | | | | | | | |
| --- | --- | --- | --- | --- | --- | --- | --- | --- |
| Count | | | | | | | | |
|  | | Which region of Malta do you live in? | | | | | | Total |
|  |  | Northern Region | Northern Harbour Region | Southern Harbour Region | South Western Region | Western Region | Gozo and Comino |  |
| Do you think these conditions can be treated with antibiotics? [HIV/AIDS] | Yes | 12 | 12 | 5 | 5 | 6 | 2 | 42 |
|  | No | 54 | 110 | 22 | 39 | 59 | 9 | 293 |
|  | Don't Know | 32 | 49 | 15 | 21 | 22 | 2 | 141 |
| Total | | 98 | 171 | 42 | 65 | 87 | 13 | 476 |

| **Chi-Square Tests** | | | |
| --- | --- | --- | --- |
|  | Value | df | Asymptotic Significance (2-sided) |
| Pearson Chi-Square | 8.117^a^ | 10 | .617 |
| Likelihood Ratio | 8.101 | 10 | .619 |
| Linear-by-Linear Association | .299 | 1 | .585 |
| N of Valid Cases | 476 |  |  |
| a. 3 cells (16.7%) have expected count less than 5. The minimum expected count is 1.15. | | | |

**Do you think these conditions can be treated with antibiotics?[Gonorrhoea] * Which region of Malta do you live in?**

| **Crosstab** | | | | | | | | |
| --- | --- | --- | --- | --- | --- | --- | --- | --- |
| Count | | | | | | | | |
|  | | Which region of Malta do you live in? | | | | | | Total |
|  |  | Northern Region | Northern Harbour Region | Southern Harbour Region | South Western Region | Western Region | Gozo and Comino |  |
| Do you think these conditions can be treated with antibiotics?[Gonorrhoea] | Yes | 32 | 70 | 17 | 18 | 32 | 5 | 174 |
|  | No | 10 | 33 | 7 | 12 | 16 | 2 | 80 |
|  | Don't Know | 56 | 68 | 18 | 35 | 39 | 6 | 222 |
| Total | | 98 | 171 | 42 | 65 | 87 | 13 | 476 |

| **Chi-Square Tests** | | | |
| --- | --- | --- | --- |
|  | Value | df | Asymptotic Significance (2-sided) |
| Pearson Chi-Square | 11.284^a^ | 10 | .336 |
| Likelihood Ratio | 11.676 | 10 | .307 |
| Linear-by-Linear Association | .010 | 1 | .919 |
| N of Valid Cases | 476 |  |  |
| a. 2 cells (11.1%) have expected count less than 5. The minimum expected count is 2.18. | | | |

**Do you think these conditions can be treated with antibiotics? [Bladder infection or urinary tract infection (UTI) ] * Which region of Malta do you live in?**

| **Crosstab** | | | | | | | | |
| --- | --- | --- | --- | --- | --- | --- | --- | --- |
| Count | | | | | | | | |
|  | | Which region of Malta do you live in? | | | | | | Total |
|  |  | Northern Region | Northern Harbour Region | Southern Harbour Region | South Western Region | Western Region | Gozo and Comino |  |
| Do you think these conditions can be treated with antibiotics? [Bladder infection or urinary tract infection (UTI) ] | Yes | 82 | 140 | 35 | 53 | 76 | 12 | 398 |
|  | No | 3 | 7 | 2 | 5 | 2 | 0 | 19 |
|  | Don't Know | 13 | 24 | 5 | 7 | 9 | 1 | 59 |
| Total | | 98 | 171 | 42 | 65 | 87 | 13 | 476 |

| **Chi-Square Tests** | | | |
| --- | --- | --- | --- |
|  | Value | df | Asymptotic Significance (2-sided) |
| Pearson Chi-Square | 5.117^a^ | 10 | .883 |
| Likelihood Ratio | 5.323 | 10 | .869 |
| Linear-by-Linear Association | 1.026 | 1 | .311 |
| N of Valid Cases | 476 |  |  |
| a. 6 cells (33.3%) have expected count less than 5. The minimum expected count is .52. | | | |

**Do you think these conditions can be treated with antibiotics? [Diarrhoea] * Which region of Malta do you live in?**

| **Crosstab** | | | | | | | | |
| --- | --- | --- | --- | --- | --- | --- | --- | --- |
| Count | | | | | | | | |
|  | | Which region of Malta do you live in? | | | | | | Total |
|  |  | Northern Region | Northern Harbour Region | Southern Harbour Region | South Western Region | Western Region | Gozo and Comino |  |
| Do you think these conditions can be treated with antibiotics? [Diarrhoea] | Yes | 13 | 26 | 7 | 11 | 14 | 2 | 73 |
|  | No | 72 | 120 | 27 | 45 | 63 | 8 | 335 |
|  | Don't Know | 13 | 25 | 8 | 9 | 10 | 3 | 68 |
| Total | | 98 | 171 | 42 | 65 | 87 | 13 | 476 |

| **Chi-Square Tests** | | | |
| --- | --- | --- | --- |
|  | Value | df | Asymptotic Significance (2-sided) |
| Pearson Chi-Square | 2.965^a^ | 10 | .982 |
| Likelihood Ratio | 2.844 | 10 | .985 |
| Linear-by-Linear Association | .151 | 1 | .698 |
| N of Valid Cases | 476 |  |  |
| a. 2 cells (11.1%) have expected count less than 5. The minimum expected count is 1.86. | | | |

**Do you think these conditions can be treated with antibiotics? [Cold and flu] * Which region of Malta do you live in?**

| **Crosstab** | | | | | | | | |
| --- | --- | --- | --- | --- | --- | --- | --- | --- |
| Count | | | | | | | | |
|  | | Which region of Malta do you live in? | | | | | | Total |
|  |  | Northern Region | Northern Harbour Region | Southern Harbour Region | South Western Region | Western Region | Gozo and Comino |  |
| Do you think these conditions can be treated with antibiotics? [Cold and flu] | Yes | 13 | 27 | 11 | 11 | 15 | 2 | 79 |
|  | No | 77 | 132 | 29 | 50 | 68 | 9 | 365 |
|  | Don't Know | 8 | 12 | 2 | 4 | 4 | 2 | 32 |
| Total | | 98 | 171 | 42 | 65 | 87 | 13 | 476 |

| **Chi-Square Tests** | | | |
| --- | --- | --- | --- |
|  | Value | df | Asymptotic Significance (2-sided) |
| Pearson Chi-Square | 6.211^a^ | 10 | .797 |
| Likelihood Ratio | 5.613 | 10 | .847 |
| Linear-by-Linear Association | .679 | 1 | .410 |
| N of Valid Cases | 476 |  |  |
| a. 4 cells (22.2%) have expected count less than 5. The minimum expected count is .87. | | | |

**Do you think these conditions can be treated with antibiotics? [Fever] * Which region of Malta do you live in?**

| **Crosstab** | | | | | | | | |
| --- | --- | --- | --- | --- | --- | --- | --- | --- |
| Count | | | | | | | | |
|  | | Which region of Malta do you live in? | | | | | | Total |
|  |  | Northern Region | Northern Harbour Region | Southern Harbour Region | South Western Region | Western Region | Gozo and Comino |  |
| Do you think these conditions can be treated with antibiotics? [Fever] | Yes | 25 | 48 | 16 | 29 | 38 | 4 | 160 |
|  | No | 63 | 107 | 25 | 33 | 42 | 8 | 278 |
|  | Don't Know | 10 | 16 | 1 | 3 | 7 | 1 | 38 |
| Total | | 98 | 171 | 42 | 65 | 87 | 13 | 476 |

| **Chi-Square Tests** | | | |
| --- | --- | --- | --- |
|  | Value | df | Asymptotic Significance (2-sided) |
| Pearson Chi-Square | 15.606^a^ | 10 | .111 |
| Likelihood Ratio | 16.244 | 10 | .093 |
| Linear-by-Linear Association | 8.846 | 1 | .003 |
| N of Valid Cases | 476 |  |  |
| a. 3 cells (16.7%) have expected count less than 5. The minimum expected count is 1.04. | | | |

**Do you think these conditions can be treated with antibiotics? [Malaria] * Which region of Malta do you live in?**

| **Crosstab** | | | | | | | | |
| --- | --- | --- | --- | --- | --- | --- | --- | --- |
| Count | | | | | | | | |
|  | | Which region of Malta do you live in? | | | | | | Total |
|  |  | Northern Region | Northern Harbour Region | Southern Harbour Region | South Western Region | Western Region | Gozo and Comino |  |
| Do you think these conditions can be treated with antibiotics? [Malaria] | Yes | 23 | 42 | 11 | 8 | 16 | 4 | 104 |
|  | No | 25 | 71 | 9 | 20 | 38 | 5 | 168 |
|  | Don't Know | 50 | 58 | 22 | 37 | 33 | 4 | 204 |
| Total | | 98 | 171 | 42 | 65 | 87 | 13 | 476 |

| **Chi-Square Tests** | | | |
| --- | --- | --- | --- |
|  | Value | df | Asymptotic Significance (2-sided) |
| Pearson Chi-Square | 23.222^a^ | 10 | .010 |
| Likelihood Ratio | 24.003 | 10 | .008 |
| Linear-by-Linear Association | .210 | 1 | .646 |
| N of Valid Cases | 476 |  |  |
| a. 2 cells (11.1%) have expected count less than 5. The minimum expected count is 2.84. | | | |

**Do you think these conditions can be treated with antibiotics? [Measles] * Which region of Malta do you live in?**

| **Crosstab** | | | | | | | | |
| --- | --- | --- | --- | --- | --- | --- | --- | --- |
| Count | | | | | | | | |
|  | | Which region of Malta do you live in? | | | | | | Total |
|  |  | Northern Region | Northern Harbour Region | Southern Harbour Region | South Western Region | Western Region | Gozo and Comino |  |
| Do you think these conditions can be treated with antibiotics? [Measles] | Yes | 15 | 12 | 6 | 6 | 11 | 4 | 54 |
|  | No | 42 | 98 | 21 | 34 | 55 | 6 | 256 |
|  | Don't Know | 41 | 61 | 15 | 25 | 21 | 3 | 166 |
| Total | | 98 | 171 | 42 | 65 | 87 | 13 | 476 |

| **Chi-Square Tests** | | | |
| --- | --- | --- | --- |
|  | Value | df | Asymptotic Significance (2-sided) |
| Pearson Chi-Square | 18.533^a^ | 10 | .047 |
| Likelihood Ratio | 17.863 | 10 | .057 |
| Linear-by-Linear Association | 4.424 | 1 | .035 |
| N of Valid Cases | 476 |  |  |
| a. 3 cells (16.7%) have expected count less than 5. The minimum expected count is 1.47. | | | |

**Do you think these conditions can be treated with antibiotics? [Skin or wound infection] * Which region of Malta do you live in?**

| **Crosstab** | | | | | | | | |
| --- | --- | --- | --- | --- | --- | --- | --- | --- |
| Count | | | | | | | | |
|  | | Which region of Malta do you live in? | | | | | | Total |
|  |  | Northern Region | Northern Harbour Region | Southern Harbour Region | South Western Region | Western Region | Gozo and Comino |  |
| Do you think these conditions can be treated with antibiotics? [Skin or wound infection] | Yes | 73 | 129 | 30 | 46 | 71 | 10 | 359 |
|  | No | 11 | 25 | 8 | 11 | 10 | 3 | 68 |
|  | Don't Know | 14 | 17 | 4 | 8 | 6 | 0 | 49 |
| Total | | 98 | 171 | 42 | 65 | 87 | 13 | 476 |

| **Chi-Square Tests** | | | |
| --- | --- | --- | --- |
|  | Value | df | Asymptotic Significance (2-sided) |
| Pearson Chi-Square | 7.681^a^ | 10 | .660 |
| Likelihood Ratio | 8.856 | 10 | .546 |
| Linear-by-Linear Association | 1.443 | 1 | .230 |
| N of Valid Cases | 476 |  |  |
| a. 3 cells (16.7%) have expected count less than 5. The minimum expected count is 1.34. | | | |

**Do you think these conditions can be treated with antibiotics? [Sore throat] * Which region of Malta do you live in?**

| **Crosstab** | | | | | | | | |
| --- | --- | --- | --- | --- | --- | --- | --- | --- |
| Count | | | | | | | | |
|  | | Which region of Malta do you live in? | | | | | | Total |
|  |  | Northern Region | Northern Harbour Region | Southern Harbour Region | South Western Region | Western Region | Gozo and Comino |  |
| Do you think these conditions can be treated with antibiotics? [Sore throat] | Yes | 32 | 62 | 23 | 33 | 36 | 4 | 190 |
|  | No | 53 | 95 | 18 | 26 | 43 | 9 | 244 |
|  | Don't Know | 13 | 14 | 1 | 6 | 8 | 0 | 42 |
| Total | | 98 | 171 | 42 | 65 | 87 | 13 | 476 |

| **Chi-Square Tests** | | | |
| --- | --- | --- | --- |
|  | Value | df | Asymptotic Significance (2-sided) |
| Pearson Chi-Square | 15.673^a^ | 10 | .109 |
| Likelihood Ratio | 17.140 | 10 | .071 |
| Linear-by-Linear Association | 3.191 | 1 | .074 |
| N of Valid Cases | 476 |  |  |
| a. 2 cells (11.1%) have expected count less than 5. The minimum expected count is 1.15. | | | |

**Do you think these conditions can be treated with antibiotics? [Body aches] * Which region of Malta do you live in?**

| **Crosstab** | | | | | | | | |
| --- | --- | --- | --- | --- | --- | --- | --- | --- |
| Count | | | | | | | | |
|  | | Which region of Malta do you live in? | | | | | | Total |
|  |  | Northern Region | Northern Harbour Region | Southern Harbour Region | South Western Region | Western Region | Gozo and Comino |  |
| Do you think these conditions can be treated with antibiotics? [Body aches] | Yes | 8 | 17 | 7 | 8 | 8 | 1 | 49 |
|  | No | 73 | 133 | 30 | 51 | 69 | 10 | 366 |
|  | Don't Know | 17 | 21 | 5 | 6 | 10 | 2 | 61 |
| Total | | 98 | 171 | 42 | 65 | 87 | 13 | 476 |

| **Chi-Square Tests** | | | |
| --- | --- | --- | --- |
|  | Value | df | Asymptotic Significance (2-sided) |
| Pearson Chi-Square | 5.367^a^ | 10 | .865 |
| Likelihood Ratio | 5.052 | 10 | .888 |
| Linear-by-Linear Association | .876 | 1 | .349 |
| N of Valid Cases | 476 |  |  |
| a. 3 cells (16.7%) have expected count less than 5. The minimum expected count is 1.34. | | | |

**Do you think these conditions can be treated with antibiotics? [Headaches] * Which region of Malta do you live in?**

| **Crosstab** | | | | | | | | |
| --- | --- | --- | --- | --- | --- | --- | --- | --- |
| Count | | | | | | | | |
|  | | Which region of Malta do you live in? | | | | | | Total |
|  |  | Northern Region | Northern Harbour Region | Southern Harbour Region | South Western Region | Western Region | Gozo and Comino |  |
| Do you think these conditions can be treated with antibiotics? [Headaches] | Yes | 3 | 9 | 3 | 2 | 4 | 1 | 22 |
|  | No | 93 | 151 | 36 | 57 | 78 | 10 | 425 |
|  | Don't Know | 2 | 11 | 3 | 6 | 5 | 2 | 29 |
| Total | | 98 | 171 | 42 | 65 | 87 | 13 | 476 |

| **Chi-Square Tests** | | | |
| --- | --- | --- | --- |
|  | Value | df | Asymptotic Significance (2-sided) |
| Pearson Chi-Square | 8.173^a^ | 10 | .612 |
| Likelihood Ratio | 8.383 | 10 | .591 |
| Linear-by-Linear Association | .921 | 1 | .337 |
| N of Valid Cases | 476 |  |  |
| a. 8 cells (44.4%) have expected count less than 5. The minimum expected count is .60. | | | |

**When do you think you should stop taking antibiotics once you've begun treatment? * Which region of Malta do you live in?**

| **Crosstab** | | | | | | | | |
| --- | --- | --- | --- | --- | --- | --- | --- | --- |
| Count | | | | | | | | |
|  | | Which region of Malta do you live in? | | | | | | Total |
|  |  | Northern Region | Northern Harbour Region | Southern Harbour Region | South Western Region | Western Region | Gozo and Comino |  |
| When do you think you should stop taking antibiotics once you've begun treatment? | When you have taken all of the antibiotics as directed | 97 | 161 | 37 | 61 | 84 | 13 | 453 |
|  | When you feel better | 1 | 9 | 5 | 4 | 3 | 0 | 22 |
|  | Don't know | 0 | 1 | 0 | 0 | 0 | 0 | 1 |
| Total | | 98 | 171 | 42 | 65 | 87 | 13 | 476 |

| **Chi-Square Tests** | | | |
| --- | --- | --- | --- |
|  | Value | df | Asymptotic Significance (2-sided) |
| Pearson Chi-Square | 11.146^a^ | 10 | .346 |
| Likelihood Ratio | 11.813 | 10 | .298 |
| Linear-by-Linear Association | .039 | 1 | .843 |
| N of Valid Cases | 476 |  |  |
| a. 11 cells (61.1%) have expected count less than 5. The minimum expected count is .03. | | | |

**Do you think this statement is ‘true’ or ‘false’? “It’s okay to use antibiotics that were given to a friend or family member, as long as they were used to treat the same illness” * Which region of Malta do you live in?**

| **Crosstab** | | | | | | | | |
| --- | --- | --- | --- | --- | --- | --- | --- | --- |
| Count | | | | | | | | |
|  | | Which region of Malta do you live in? | | | | | | Total |
|  |  | Northern Region | Northern Harbour Region | Southern Harbour Region | South Western Region | Western Region | Gozo and Comino |  |
| Do you think this statement is ‘true’ or ‘false’? “It’s okay to use antibiotics that were given to a friend or family member, as long as they were used to treat the same illness” | True | 5 | 11 | 5 | 3 | 4 | 0 | 28 |
|  | False | 90 | 155 | 34 | 59 | 81 | 13 | 432 |
|  | Don't Know | 3 | 5 | 3 | 3 | 2 | 0 | 16 |
| Total | | 98 | 171 | 42 | 65 | 87 | 13 | 476 |

| **Chi-Square Tests** | | | |
| --- | --- | --- | --- |
|  | Value | df | Asymptotic Significance (2-sided) |
| Pearson Chi-Square | 7.542^a^ | 10 | .673 |
| Likelihood Ratio | 7.763 | 10 | .652 |
| Linear-by-Linear Association | .142 | 1 | .706 |
| N of Valid Cases | 476 |  |  |
| a. 8 cells (44.4%) have expected count less than 5. The minimum expected count is .44. | | | |

**Do you think this statement is ‘true’ or ‘false’? “It’s okay to buy the same antibiotics, or request these from a doctor, if you’re sick and they helped you get better when you had the same symptoms before” * Which region of Malta do you live in?**

| **Crosstab** | | | | | | | | |
| --- | --- | --- | --- | --- | --- | --- | --- | --- |
| Count | | | | | | | | |
|  | | Which region of Malta do you live in? | | | | | | Total |
|  |  | Northern Region | Northern Harbour Region | Southern Harbour Region | South Western Region | Western Region | Gozo and Comino |  |
| Do you think this statement is ‘true’ or ‘false’? “It’s okay to buy the same antibiotics, or request these from a doctor, if you’re sick and they helped you get better when you had the same symptoms before” | True | 21 | 31 | 14 | 19 | 24 | 4 | 113 |
|  | False | 73 | 136 | 28 | 44 | 61 | 7 | 349 |
|  | Don't Know | 4 | 4 | 0 | 2 | 2 | 2 | 14 |
| Total | | 98 | 171 | 42 | 65 | 87 | 13 | 476 |

| **Chi-Square Tests** | | | |
| --- | --- | --- | --- |
|  | Value | df | Asymptotic Significance (2-sided) |
| Pearson Chi-Square | 16.851^a^ | 10 | .078 |
| Likelihood Ratio | 14.586 | 10 | .148 |
| Linear-by-Linear Association | 2.491 | 1 | .114 |
| N of Valid Cases | 476 |  |  |
| a. 6 cells (33.3%) have expected count less than 5. The minimum expected count is .38. | | | |

**Please indicate whether you think the following statements are ‘true’ or ‘false’ [Antibiotic resistance occurs when your body becomes resistant to antibiotics and they no longer work as well.] * Which region of Malta do you live in?**

| **Crosstab** | | | | | | | | |
| --- | --- | --- | --- | --- | --- | --- | --- | --- |
| Count | | | | | | | | |
|  | | Which region of Malta do you live in? | | | | | | Total |
|  |  | Northern Region | Northern Harbour Region | Southern Harbour Region | South Western Region | Western Region | Gozo and Comino |  |
| Please indicate whether you think the following statements are ‘true’ or ‘false’ [Antibiotic resistance occurs when your body becomes resistant to antibiotics and they no longer work as well.] | True | 69 | 124 | 34 | 53 | 64 | 11 | 355 |
|  | False | 16 | 33 | 3 | 8 | 14 | 2 | 76 |
|  | Don't Know | 13 | 14 | 5 | 4 | 9 | 0 | 45 |
| Total | | 98 | 171 | 42 | 65 | 87 | 13 | 476 |

| **Chi-Square Tests** | | | |
| --- | --- | --- | --- |
|  | Value | df | Asymptotic Significance (2-sided) |
| Pearson Chi-Square | 9.072^a^ | 10 | .525 |
| Likelihood Ratio | 10.718 | 10 | .380 |
| Linear-by-Linear Association | 1.742 | 1 | .187 |
| N of Valid Cases | 476 |  |  |
| a. 3 cells (16.7%) have expected count less than 5. The minimum expected count is 1.23. | | | |

**Please indicate whether you think the following statements are ‘true’ or ‘false’ [Many infections are becoming increasingly resistant to treatment by antibiotics ] * Which region of Malta do you live in?**

| **Crosstab** | | | | | | | | |
| --- | --- | --- | --- | --- | --- | --- | --- | --- |
| Count | | | | | | | | |
|  | | Which region of Malta do you live in? | | | | | | Total |
|  |  | Northern Region | Northern Harbour Region | Southern Harbour Region | South Western Region | Western Region | Gozo and Comino |  |
| Please indicate whether you think the following statements are ‘true’ or ‘false’ [Many infections are becoming increasingly resistant to treatment by antibiotics ] | True | 71 | 130 | 31 | 43 | 71 | 9 | 355 |
|  | False | 2 | 12 | 3 | 4 | 4 | 2 | 27 |
|  | Don't Know | 25 | 29 | 8 | 18 | 12 | 2 | 94 |
| Total | | 98 | 171 | 42 | 65 | 87 | 13 | 476 |

| **Chi-Square Tests** | | | |
| --- | --- | --- | --- |
|  | Value | df | Asymptotic Significance (2-sided) |
| Pearson Chi-Square | 12.802^a^ | 10 | .235 |
| Likelihood Ratio | 12.683 | 10 | .242 |
| Linear-by-Linear Association | .566 | 1 | .452 |
| N of Valid Cases | 476 |  |  |
| a. 5 cells (27.8%) have expected count less than 5. The minimum expected count is .74. | | | |

**Please indicate whether you think the following statements are ‘true’ or ‘false’[If bacteria are resistant to antibiotics, it can be very difficult or impossible to treat the infections they cause] * Which region of Malta do you live in?**

| **Crosstab** | | | | | | | | |
| --- | --- | --- | --- | --- | --- | --- | --- | --- |
| Count | | | | | | | | |
|  | | Which region of Malta do you live in? | | | | | | Total |
|  |  | Northern Region | Northern Harbour Region | Southern Harbour Region | South Western Region | Western Region | Gozo and Comino |  |
| Please indicate whether you think the following statements are ‘true’ or ‘false’[If bacteria are resistant to antibiotics, it can be very difficult or impossible to treat the infections they cause] | True | 82 | 135 | 31 | 47 | 75 | 11 | 381 |
|  | False | 3 | 18 | 5 | 6 | 1 | 1 | 34 |
|  | Don't Know | 13 | 18 | 6 | 12 | 11 | 1 | 61 |
| Total | | 98 | 171 | 42 | 65 | 87 | 13 | 476 |

| **Chi-Square Tests** | | | |
| --- | --- | --- | --- |
|  | Value | df | Asymptotic Significance (2-sided) |
| Pearson Chi-Square | 15.131^a^ | 10 | .127 |
| Likelihood Ratio | 17.451 | 10 | .065 |
| Linear-by-Linear Association | .009 | 1 | .924 |
| N of Valid Cases | 476 |  |  |
| a. 4 cells (22.2%) have expected count less than 5. The minimum expected count is .93. | | | |

**Please indicate whether you think the following statements are ‘true’ or ‘false’ [Antibiotic resistance is an issue that could affect me or my family] * Which region of Malta do you live in?**

| **Crosstab** | | | | | | | | |
| --- | --- | --- | --- | --- | --- | --- | --- | --- |
| Count | | | | | | | | |
|  | | Which region of Malta do you live in? | | | | | | Total |
|  |  | Northern Region | Northern Harbour Region | Southern Harbour Region | South Western Region | Western Region | Gozo and Comino |  |
| Please indicate whether you think the following statements are ‘true’ or ‘false’ [Antibiotic resistance is an issue that could affect me or my family] | True | 81 | 145 | 31 | 54 | 66 | 10 | 387 |
|  | False | 3 | 8 | 4 | 3 | 7 | 3 | 28 |
|  | Don't Know | 14 | 18 | 7 | 8 | 14 | 0 | 61 |
| Total | | 98 | 171 | 42 | 65 | 87 | 13 | 476 |

| **Chi-Square Tests** | | | |
| --- | --- | --- | --- |
|  | Value | df | Asymptotic Significance (2-sided) |
| Pearson Chi-Square | 14.798^a^ | 10 | .140 |
| Likelihood Ratio | 13.724 | 10 | .186 |
| Linear-by-Linear Association | .789 | 1 | .374 |
| N of Valid Cases | 476 |  |  |
| a. 4 cells (22.2%) have expected count less than 5. The minimum expected count is .76. | | | |

**Please indicate whether you think the following statements are ‘true’ or ‘false’ [Antibiotic resistance is an issue in other countries but not here] * Which region of Malta do you live in?**

| **Crosstab** | | | | | | | | |
| --- | --- | --- | --- | --- | --- | --- | --- | --- |
| Count | | | | | | | | |
|  | | Which region of Malta do you live in? | | | | | | Total |
|  |  | Northern Region | Northern Harbour Region | Southern Harbour Region | South Western Region | Western Region | Gozo and Comino |  |
| Please indicate whether you think the following statements are ‘true’ or ‘false’ [Antibiotic resistance is an issue in other countries but not here] | True | 2 | 3 | 3 | 2 | 6 | 1 | 17 |
|  | False | 76 | 146 | 31 | 46 | 72 | 12 | 383 |
|  | Don't Know | 20 | 22 | 8 | 17 | 9 | 0 | 76 |
| Total | | 98 | 171 | 42 | 65 | 87 | 13 | 476 |

| **Chi-Square Tests** | | | |
| --- | --- | --- | --- |
|  | Value | df | Asymptotic Significance (2-sided) |
| Pearson Chi-Square | 19.484^a^ | 10 | .035 |
| Likelihood Ratio | 20.508 | 10 | .025 |
| Linear-by-Linear Association | 3.473 | 1 | .062 |
| N of Valid Cases | 476 |  |  |
| a. 6 cells (33.3%) have expected count less than 5. The minimum expected count is .46. | | | |

**Please indicate whether you think the following statements are ‘true’ or ‘false’ [Antibiotic resistance is only a problem for people who take antibiotics regularly] * Which region of Malta do you live in?**

| **Crosstab** | | | | | | | | |
| --- | --- | --- | --- | --- | --- | --- | --- | --- |
| Count | | | | | | | | |
|  | | Which region of Malta do you live in? | | | | | | Total |
|  |  | Northern Region | Northern Harbour Region | Southern Harbour Region | South Western Region | Western Region | Gozo and Comino |  |
| Please indicate whether you think the following statements are ‘true’ or ‘false’ [Antibiotic resistance is only a problem for people who take antibiotics regularly] | True | 16 | 27 | 18 | 15 | 16 | 4 | 96 |
|  | False | 57 | 118 | 19 | 38 | 55 | 8 | 295 |
|  | Don't Know | 25 | 26 | 5 | 12 | 16 | 1 | 85 |
| Total | | 98 | 171 | 42 | 65 | 87 | 13 | 476 |

| **Chi-Square Tests** | | | |
| --- | --- | --- | --- |
|  | Value | df | Asymptotic Significance (2-sided) |
| Pearson Chi-Square | 23.341^a^ | 10 | .010 |
| Likelihood Ratio | 20.901 | 10 | .022 |
| Linear-by-Linear Association | 2.355 | 1 | .125 |
| N of Valid Cases | 476 |  |  |
| a. 2 cells (11.1%) have expected count less than 5. The minimum expected count is 2.32. | | | |

**Please indicate whether you think the following statements are ‘true’ or ‘false’ [Bacteria which are resistant to antibiotics can be spread from person to person] * Which region of Malta do you live in?**

| **Crosstab** | | | | | | | | |
| --- | --- | --- | --- | --- | --- | --- | --- | --- |
| Count | | | | | | | | |
|  | | Which region of Malta do you live in? | | | | | | Total |
|  |  | Northern Region | Northern Harbour Region | Southern Harbour Region | South Western Region | Western Region | Gozo and Comino |  |
| Please indicate whether you think the following statements are ‘true’ or ‘false’ [Bacteria which are resistant to antibiotics can be spread from person to person] | True | 61 | 107 | 24 | 36 | 54 | 8 | 290 |
|  | False | 10 | 26 | 6 | 6 | 9 | 1 | 58 |
|  | Don't Know | 27 | 38 | 12 | 23 | 24 | 4 | 128 |
| Total | | 98 | 171 | 42 | 65 | 87 | 13 | 476 |

| **Chi-Square Tests** | | | |
| --- | --- | --- | --- |
|  | Value | df | Asymptotic Significance (2-sided) |
| Pearson Chi-Square | 6.498^a^ | 10 | .772 |
| Likelihood Ratio | 6.439 | 10 | .777 |
| Linear-by-Linear Association | .573 | 1 | .449 |
| N of Valid Cases | 476 |  |  |
| a. 2 cells (11.1%) have expected count less than 5. The minimum expected count is 1.58. | | | |

**Please indicate whether you think the following statements are ‘true’ or ‘false’ [Antibiotic-resistant infections could make medical procedures like surgery, organ transplants and cancer treatment much more dangerous] * Which region of Malta do you live in?**

| **Crosstab** | | | | | | | | |
| --- | --- | --- | --- | --- | --- | --- | --- | --- |
| Count | | | | | | | | |
|  | | Which region of Malta do you live in? | | | | | | Total |
|  |  | Northern Region | Northern Harbour Region | Southern Harbour Region | South Western Region | Western Region | Gozo and Comino |  |
| Please indicate whether you think the following statements are ‘true’ or ‘false’ [Antibiotic-resistant infections could make medical procedures like surgery, organ transplants and cancer treatment much more dangerous] | True | 76 | 140 | 30 | 48 | 71 | 13 | 378 |
|  | False | 1 | 7 | 3 | 2 | 3 | 0 | 16 |
|  | Don't Know | 21 | 24 | 9 | 15 | 13 | 0 | 82 |
| Total | | 98 | 171 | 42 | 65 | 87 | 13 | 476 |

| **Chi-Square Tests** | | | |
| --- | --- | --- | --- |
|  | Value | df | Asymptotic Significance (2-sided) |
| Pearson Chi-Square | 11.862^a^ | 10 | .294 |
| Likelihood Ratio | 14.502 | 10 | .151 |
| Linear-by-Linear Association | .481 | 1 | .488 |
| N of Valid Cases | 476 |  |  |
| a. 6 cells (33.3%) have expected count less than 5. The minimum expected count is .44. | | | |
